# Uncovering α-Selectivity for Liver X Receptor Agonists for Lipotoxic Cancer Therapies

**DOI:** 10.1021/acs.jmedchem.4c02712

**Published:** 2025-03-24

**Authors:** Júlia Galvez B. Pedreira, Pascal Woelffing, Moritz Schwarz, Simon Ebner, Ramona Rudalska, Benedikt Masberg, Aylin Esposito, Azam Rashidian, Ekaterina Schevchenko, Lucie Smutna, Petr Pavek, Jenni Kublbeck, Thales Kronenberger, Lars Zender, Michael Lämmerhofer, Daniel Dauch, Stefan A. Laufer

**Affiliations:** 1 Department of Pharmaceutical and Medicinal Chemistry, Institute of Pharmaceutical Sciences, University of Tuebingen, Auf der Morgenstelle 8, Tuebingen 72076, Germany; 2 IFIT Cluster of Excellence EXC 2180 ‘Image-Guided and Functionally Instructed Tumor Therapies’, University of Tuebingen, Tuebingen 72076, Germany; 3 Department of Medical Oncology and Pneumology, University Hospital Tuebingen, Otfried-Mueller-Strasse 14, Tuebingen 72076, Germany; 4 Pharmaceutical (Bio-)Analysis, Institute of Pharmaceutical Sciences, Eberhard-Karls University of Tuebingen, Auf der Morgenstelle 8, Tuebingen 72076, Germany; 5 Department of Pharmacology and Toxicology, Faculty of Pharmacy in Hradec Kralove, Charles University, Akademika Heyrovskeho 1203, Hradec Kralove 500 05, Czech Republic; 6 School of Pharmacy, Faculty of Health Sciences, University of Eastern Finland, P.O. Box 1627, Kuopio FI-70210, Finland; 7 A.I. Virtanen Institute for Molecular Sciences, University of Eastern Finland, P.O. Box 1627, Kuopio FI-70210, Finland; 8 Partner-site Tuebingen, German Center for Infection Research (DZIF), Elfriede-Aulhorn-Str. 6, Tuebingen 72076, Germany; 9 Tuebingen Center for Academic Drug Discovery & Development (TüCAD2), Auf der Morgenstelle 8, Tuebingen 72076, Germany; 10 German Cancer Research Consortium (DKTK), Partner Site Tuebingen, German Cancer Research Center (DKFZ), Im Neuenheimer Feld 280, Heidelberg 69120, Germany

## Abstract

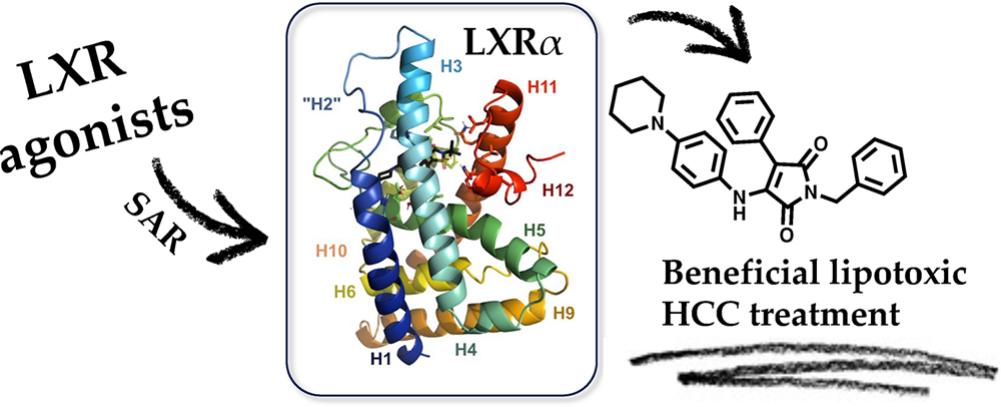

Hepatocellular carcinoma (HCC) is one of the most frequent causes of cancer-related deaths worldwide. We recently showed that pharmacologically induced lipotoxicity represents a promising therapeutic strategy for the treatment of HCC. Synthetic LXRα agonists induce the production of toxic saturated fatty acids in tumor cells. When combined with DFG-out Raf inhibitors, which block fatty acid desaturation by inducing proteasomal degradation of stearoyl-CoA desaturase (SCD1), LXRα activation can trigger lipotoxicity-induced cancer cell death. However, the clinical translation of this therapeutic strategy is limited by the lack of specific LXRα agonists for clinical use. Here, we have developed a series of promising maleimide LXR agonists with increased potency for LXRα and enhanced specificity. Our agonist frontrunner **40** shows high selectivity for LXRα and strong therapeutic efficacy in HCC organoids, therefore illustrating a strong potential for advancing this lipotoxic treatment strategy to clinical application.

## 1 Introduction

Primary liver cancer, mainly consisting of hepatocellular carcinoma (HCC, ∼90% of cases) and intrahepatic cholangiocarcinoma (iCCA), is the third most common cause of cancer-related deaths worldwide.^1,2^ The incidence of liver cancer (more than 800,000 cases in 2022) is expected to continue to rise in the future, as the number of cancer-causing liver diseases such as nonalcoholic steatohepatitis (NASH) increases.^3^ HCCs generally show strong resistance to cytotoxic and targeted therapies,^2^ and only a fraction of tumors respond to immunotherapies.^4^ In particular, the increasing proportion of NASH-related HCCs is largely resistant to checkpoint inhibitors.^4,5^

**Figure 1:**
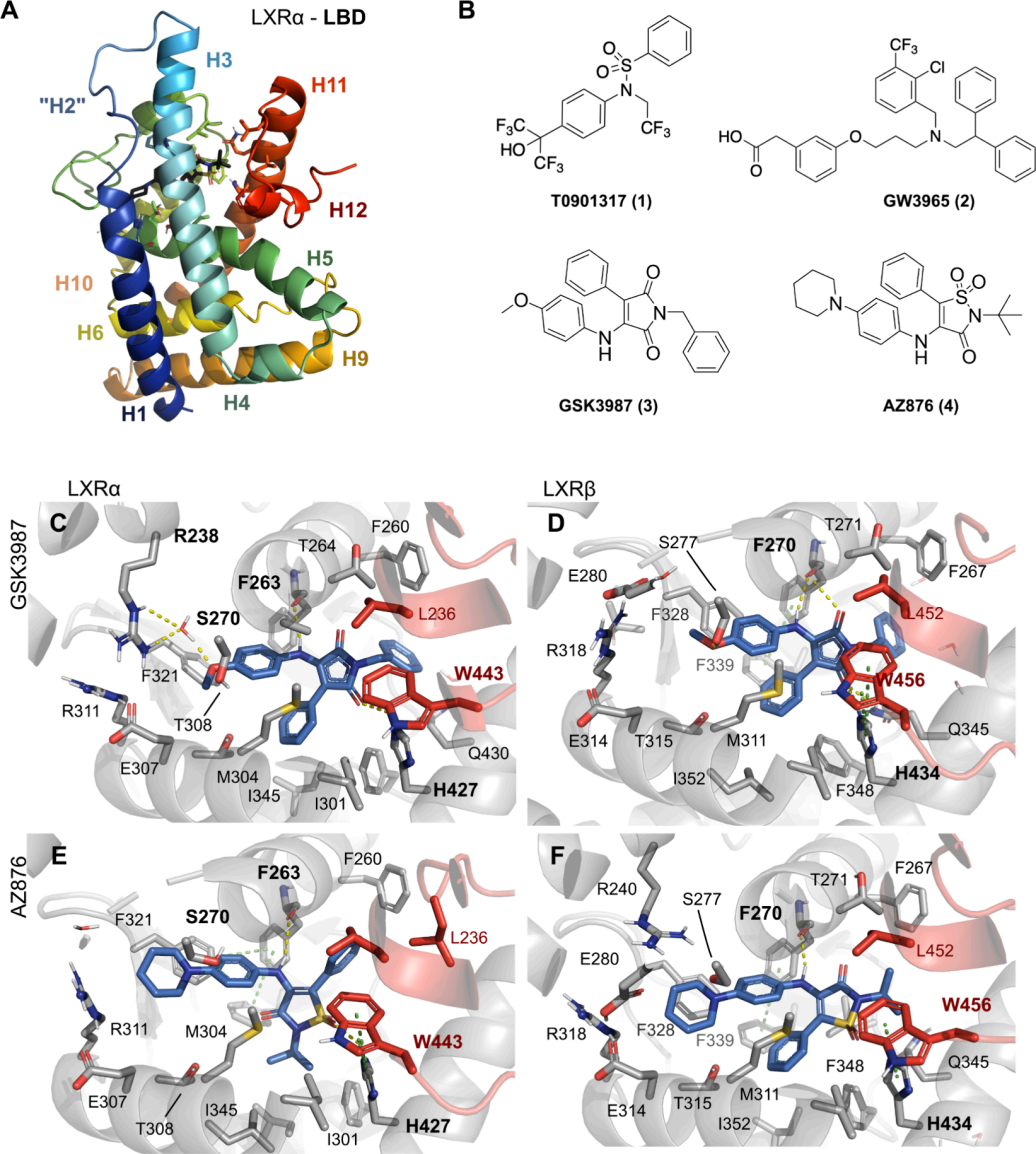
(A) LXRα’s ligand binding domain (LBD) model structure with AZ876 (4) (based on the mice LXRα, PDB ID 2ACL) highlighting the 12 helices (H1–H12). (B) Chemical structure of LXR agonists. (C–F) Close-up view of the ligand binding pocket, highlighting key recognition elements for the maleimide/thiazole binding: hydrogen bond interaction between the carbonyl or sulfonyl and His427 on helix 10/11 promotes an electrostatic interaction between His427 and Trp443 (numbering based on isoform a) on AF-2 which, in turn, leads to stabilization of AF-2 and LXR activation. Figures made using the program PyMOL (v2.5).

We recently showed that pharmacologically induced lipotoxicity represents a promising novel therapeutic strategy for the treatment of HCC.^6^ This therapy is based on the activation of *de novo* fatty acid synthesis in tumor cells via synthetic agonists of the nuclear receptor and transcription factor liver X receptor alpha (LXRα). In combination with an inhibition of the Raf-1 kinase, which blocks fatty acid desaturation, this results in an intracellular accumulation of saturated fatty acids. Such an accumulation of toxic saturated fatty acids induces severe lipotoxicity in the tumor cells, leading to oxidative stress, an endoplasmic reticulum (ER) stress response, and finally apoptosis. We found that Raf-1 directly binds the stearoyl-CoA desaturase (SCD1) and enhances its function by preventing its proteasomal degradation. Conformation changing (DFG-out) Raf inhibitors block Raf-1-mediated SCD1 stabilization and thus reduce SCD1 activity. This results in a toxic accumulation of saturated fatty acids in combination with different LXR agonists such as T0901317 (**1**) or GW3965 (**2**) (Figure 1).^6^

This therapeutic approach, designated as “induced lipotoxicity”, was able to overcome therapy resistance in *in vivo* models of murine and human HCCs and was well tolerated by mice,^6^ indicating a strong potential for the treatment of HCC patients in the future. However, while DFG-out Raf inhibitors such as the multikinase inhibitor sorafenib are already in daily use for the treatment of HCC patients, specific LXRα agonists are not applicable in the clinic so far.^7^ Consequently, the development of specific LXRα activating ligands is a prerequisite for bringing this promising therapeutic approach into clinical use.

Nuclear receptors (NRs) compose a superfamily of transcription factors that regulate various cell processes, mainly upon activation by endogenous lipophilic ligands. Almost all of the 48 known NRs encoded by the human genome share a common overall architecture.^8,9^ The binding of ligands induces conformational changes in the receptor, which in turn, leads to the specific recruitment of cofactors to stimulate or repress transcription.^10^ The LXR isoforms (α and β), important regulators of fatty acid and cholesterol metabolism, function as heterodimers in complex with the retinoid X receptors (RXRs). LXR folding consists of a N-terminal domain, a DNA binding domain (DBD, containing zinc fingers), and a ligand binding domain (LBD) for synthetic agonists or natural ligands such as oxysterols. The LBD is composed of 12 helices (H1–H12, Figure 1A) and contains the C-terminal activation domain. In their apostate (i.e., ligand binding pocket unoccupied), LXR heterodimers bind to the promoter regions of target genes in complex with corepressor proteins.^11−13^ Upon binding of activating ligands in the LBD’s pocket, LXR releases corepressor proteins and changes the conformation of helix 12 (H12) to the active state in which the surface of H3, H5, and H12 can receive coactivator proteins to induce target gene transcription.^14,15^

Due to their important role as transcriptional regulators, much interest has been given to NRs in drug discovery. Activation of LXR has been suggested for the treatment of different kinds of diseases, such as atherosclerosis, Alzheimer’s disease, retinopathy, or brain cancer,^16^ and many different LXR agonists were developed. Some agonists, such as LXR-623^17^ or BMS-779788,^18^ were already tested in phase I clinical trials (NCT00379860, NCT00366522, and NCT00836602), revealing safety and tolerability in humans. However, isoform-specific activation of LXR has proven to be a challenge, as their ligand binding pockets (LBP) are highly similar, and many agonists, such as T0901317 (**1**, Figure 1B), activate both isoforms. Moreover, to avoid potential toxic effects due to LXRα-induced liponeogenesis, recent agonists were rather designed to preferentially induce LXRß instead of LXRα.^16−18^ Therefore, the currently available LXR agonists do not allow for specific activation of LXRα, making them unsuitable for lipotoxic cancer therapy.

For the development of LXRα agonist, in the scope of a therapeutically active lipotoxicity, we analyzed previously described ligands GSK3987 (**3**) and AZ876 (**4**).^19^ The crystal structure of **3** shows that the maleimide core structure allows for all classical interactions promoting nuclear receptor (NR) activation (Figure 1C,D). Specifically, the 2-oxo group forms a strong H-bond interaction with His427 (H10/11), which further stabilizes this amino acid’s interaction with Trp443, an important feature in the active form of the protein. AZ876 (**4**, Figure 1E,F), which is an isothiazole agonist with higher activity for LXRα, suggests a correlation between lipogenic effects and target interaction,^20^ where engaging with the activating H12 while avoiding H3 could enable lipogenesis.

Considering the information regarding α and β selectivity and its correlation with lipogenesis, we were able to design agonists that show strongly improved selectivity for LXRα and demonstrate strong therapeutic efficacy in combination with a DFG-out Raf inhibitor in HCC cells and liver cancer organoid cultures.

## 2 Results and Discussion

### 2.1 Molecular Design

**Figure 2:**
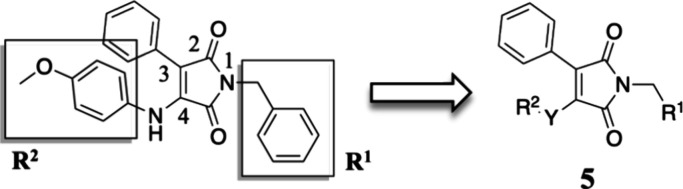
Simplified molecular design of maleimide derivatives inducing LXR activation.

We used the maleimide core as a starting point for molecular design and SAR investigation given its relevant interactions with LXR (Figure 1). By iterative modification of residues R^1^ and R^2^ (Figure 2), we aimed at improving potency and identifying α-selectivity driving interactions.

The initial approach was the variation of R^1^ (accommodated in the hydrophobic pocket of the L:H11 and H12) while maintaining the para-methoxy residue in R^2^ of compound **3** and phenyl at position 3 of the maleimide core (H1–H2’s pocket). Modifications at this residue aimed to explore different steric and electronic profiles.

The second point of modification was the NH bond between the R^2^ residue and the maleimide core. To gain insight into the relevance of the H-bond with Phe263’s main chain, we prepared compounds with an ether linker or no linker, where the aromatic ring is bonded directly to C-4 of maleimide.

The third set of compounds was prepared by variation of the R^2^ residue, where we aimed to explore the depth of the ligand binding pocket and possible new interactions with residues H1 and H2. The comparison between the crystal structures of GSK3987 (**3**) and AZ876 (**4**) showed that, in the latter, the *N*-phenylpiperidine moiety occupies a deep pocket, which could offer new interactions driving selectivity.

### 2.2 Synthesis

Synthesis of most maleimides started from phenylacetic acid (**6**). The carboxylic acid was converted into the substituted amide **7** bearing the intended R^1^ group through classic amide preparation from acyl chloride. The substituted amide (**7**) or unsubstituted amide (**13**) was cyclized to a maleimide by reaction with diethyloxalate and KOtBu.

**Scheme 1:**
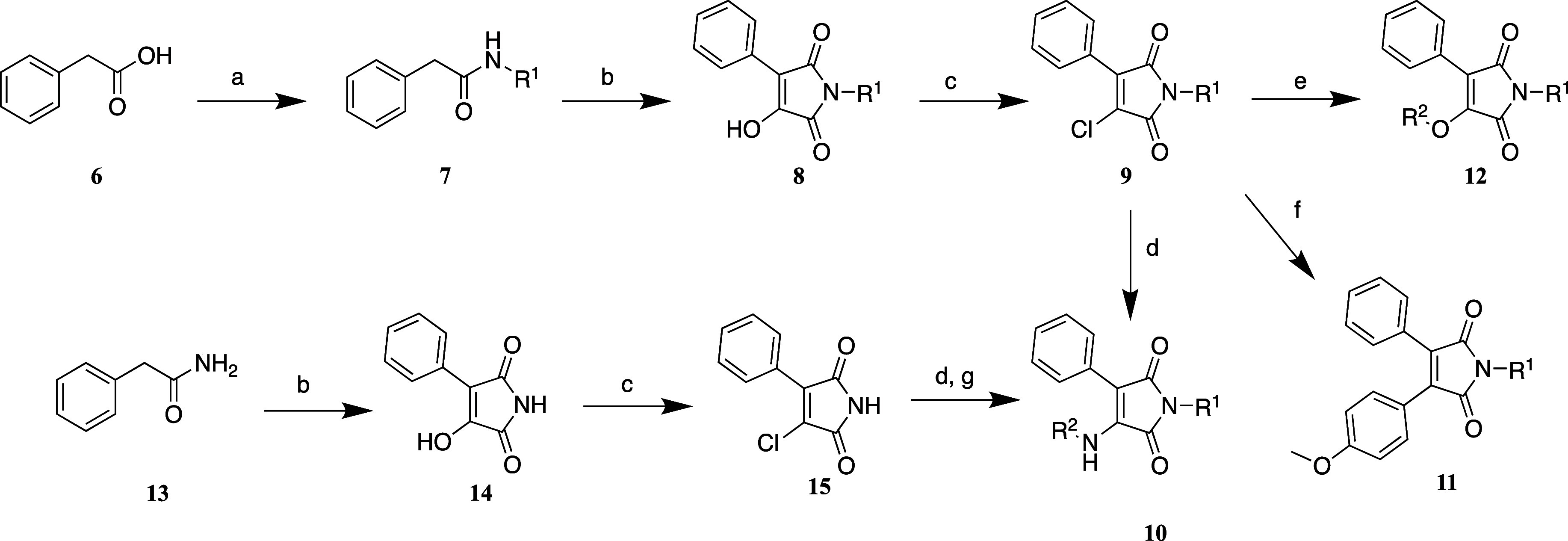
Synthetic Route for Maleimide Derivatives Reagents and conditions: (a) (1) oxalyl chloride, THF, 0 °C, 1 h; (2) R^1^NH_2_, THF, r.t., o.n., 60–98%; (b) diethyloxalate, KOtBu, THF, r.t., 1 h, 86–98%; (c) oxalyl chloride, DMF/DCM, r.t., o.n., 54–61%; (d) R^2^NH_2_, Et_3_N, dioxane, 100 °C, 2 h, 16–93%; (e) R^2^OH, KOtBu, THF, 80 °C, o.n., 38%; (f) R^2^B(OH)_2_, K_2_CO_3_, Pd(dppf)Cl_2_, dioxane, 100 °C, o.n., 85%; (g) R^1^Br, K_2_CO_3_, DMF, 50 °C, 2 h, 11–86%.

The hydroxyl group at the 4-position of the maleimide was converted to chloride derivative **9** or **15** through halogenation with oxalyl chloride. The conversion of **8** to **10** had been previously described directly;^21^ however, the conversion from the hydroxy group to its halogen analog optimized the yields and resulted in less side products. Maleimide derivatives with NH at the 4-position (**10**) were obtained by substitution reaction with the respective amine and triethylamine in dioxane. For the unsubstituted maleimide **15**, the R^1^ group was introduced later by a simple substitution reaction with the respective bromomethyl-substituted residue and potassium carbonate. The O-analogues were synthesized by reaction with phenols and KOtBu. Lastly, the C–C derivative at position 4 was prepared from Suzuki coupling with the appropriate boronic acid and Pd(dppf)Cl_2_ (Scheme 1).

### 2.3 Biochemical Evaluation with TR-FRET Assay

The initial point of modification in our strategy was R^1^, and for comparison in the structure–activity relationship, we used time-resolved fluorescence resonance energy transfer (TR-FRET) assay for both LXRα and LXRβ isoforms, validating the on-target binding and target activation of all of our novel compounds. We introduced point modifications at the para position of the benzyl ring to identify possible electronic and steric effects. The EC_50_ values for both isoforms obtained by the FRET assay are displayed on Table 1 in comparison to GSK3987 (**3**).

**Table 1:**
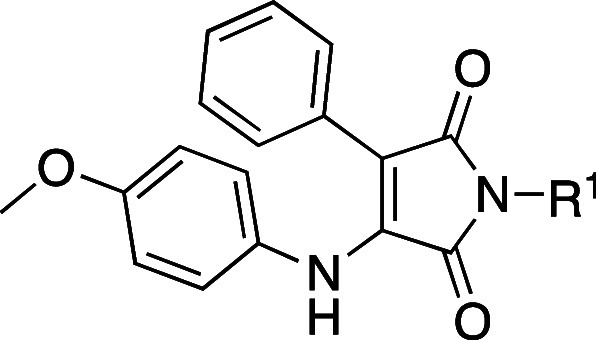

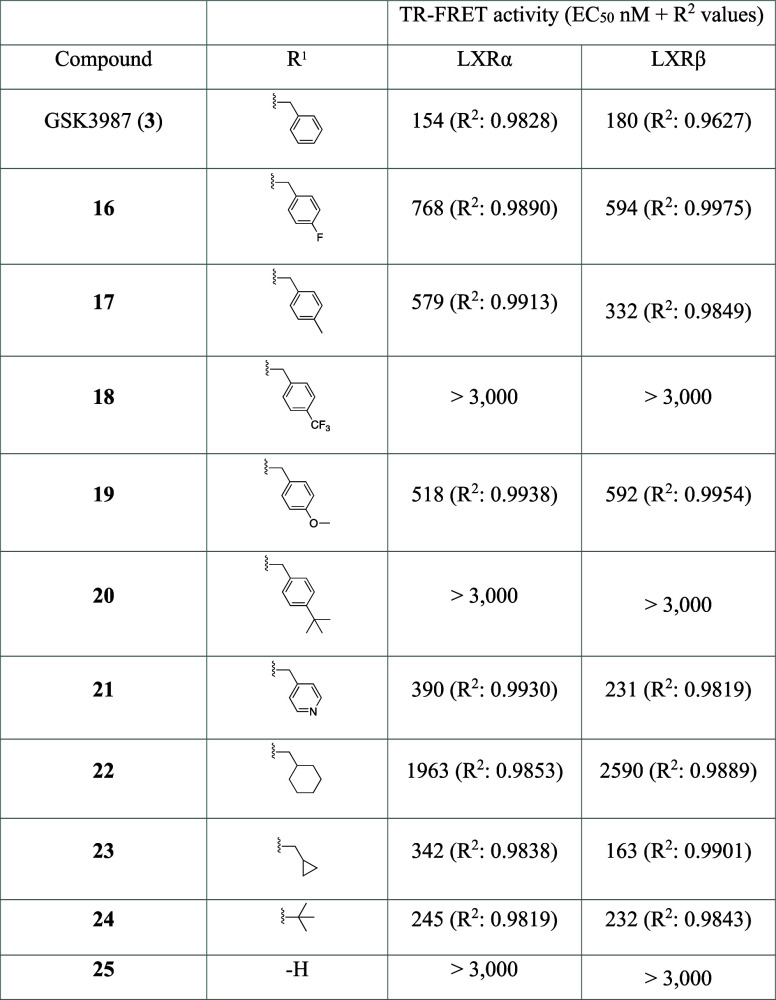
Activity of Maleimide Derivatives on LXRα and LXRβ with Modifications of R^1^

GSK3987 (**3**) displayed mild activity in our essay, however with no selectivity. Introduction of the electron-withdrawing group -F (**16**) led to a loss of activity and showed no selectivity. The bulkier −CH_3_ group (**17**) displayed a slight increase in activity for the β-isoform, and its isostere −CF_3_ (**18**) completely lost potency. We then investigated even bulkier groups, with an inverse electron-donating effect. However, the results for compounds **19** and **20** showed that bigger groups could not be tolerated, and donating electronic effects did not show improvement.

As different substituents at the ring showed no increased activity, we decided to test different ring systems at R^1^. The heteroaromatic system of pyridine in **21** displayed only a mild agonistic effect, and the nonaromatic cyclohexane substituent (**22**) led to a significant loss of activity. Compound **23** however, which bears the phenyl isoster cyclopropane, showed a slight improvement but with preference for the β-isoform. Finally, we tested the tert-butyl system, similarly present in isothiazole AZ876 (**4**). Compound **24** displayed comparable activity to that of reference GSK3987 (**3**), although analogously unselective. These data suggested that activity was influenced mostly by hydrophobic interactions. As a final confirmation of the relevance of the R^1^ group, derivative **25** with no substitution showed no activity.

Before we moved on to the modifications of the R^2^ residue, we decided to investigate the linker between R^2^ and the maleimide core (Table 2). As previously shown, the crystal structure of GSK3987 (**3**) bound to both isoforms (Figure 1C,D) displays a hydrogen bond interaction between the NH group and the backbone of Phe263. As a straightforward way to investigate the relevance of this interaction, we synthesized simple analogs of GSK3987 (**3**), namely, the ether-linker analog **26** and compound **27**, with no linker to the *p*-methoxyphenyl residue. As expected, both compounds completely lost activity. Additionally, compound **28** with methylene elongation of the linker part also displayed no activity. Therefore, we consider that the acidity of the NH group or simple flexibility requirements are important factors for this linker. Nonetheless, there seem to be space constrictions for the pocket. Finally, we moved into the last iteration, i.e. modification of R^2^ to explore potency and selectivity (Table 3).

**Table 2:**
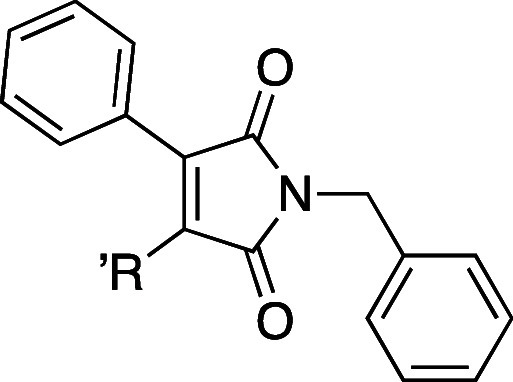

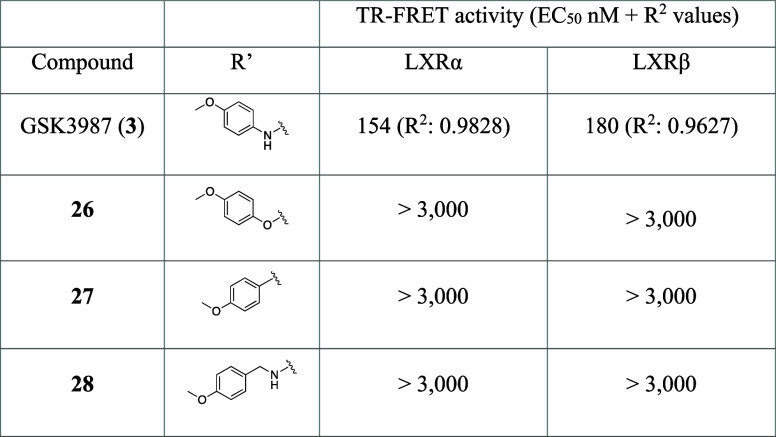
Activity of Maleimide Derivatives on LXRα and LXRβ with Linker Modifications

**Table 3:**
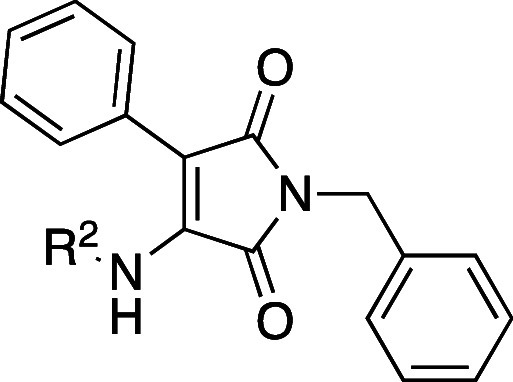

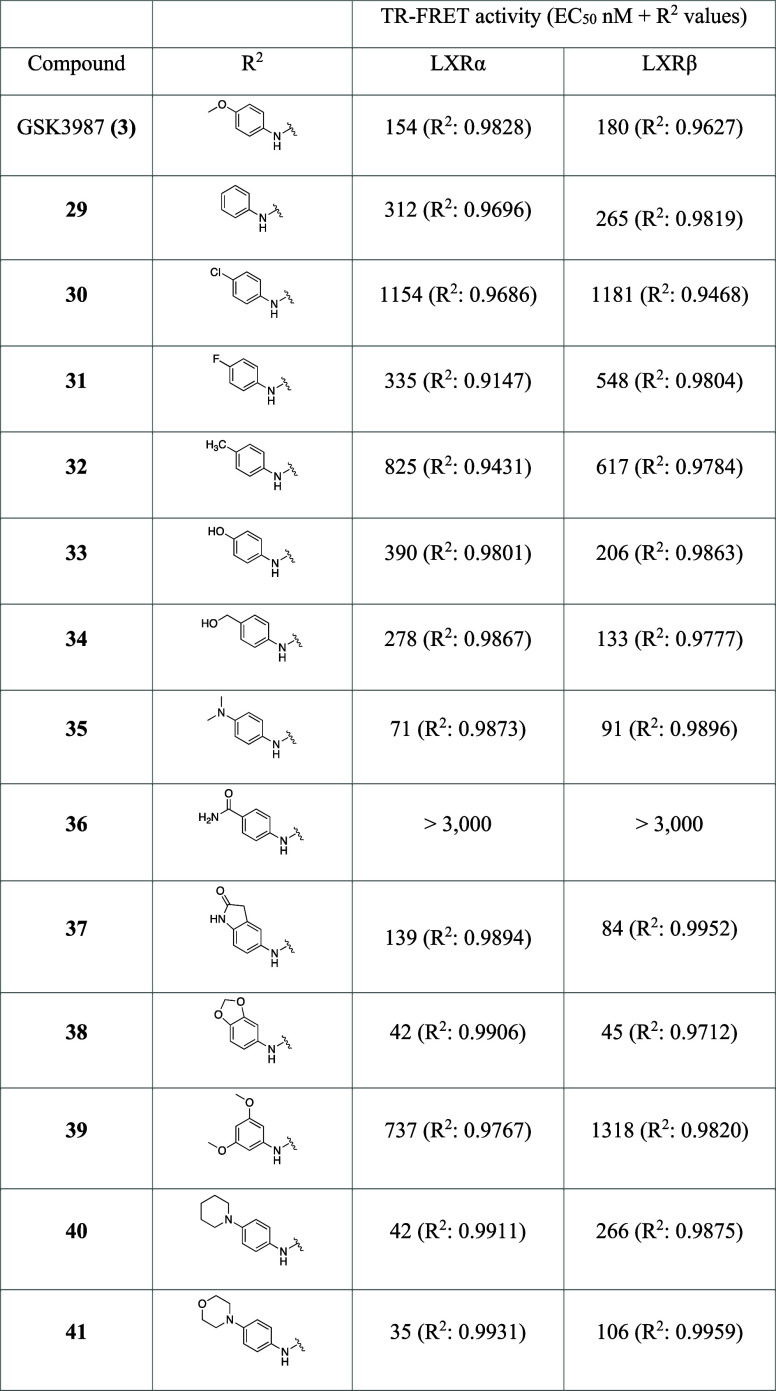
Activation of LXRα and LXRβ of Compounds with Modifications of R^2^

Initially, we tested two previously reported maleimides by Jaye et al.*,*^22^ bearing a phenyl ring (**29**) and *p*-chlorophenyl ring (**30**) at the R^2^ position. We observed that the simplified phenyl derivative **29** already showed a loss of activity, and the halogen-substituted compound (**30**) was almost inactive. To investigate whether the halogen nature was relevant, we tested the fluorine-substituted compound **31**, which had only mild activity. The introduction of a methyl group (**32**) also resulted in high EC_50_ values for both isoforms. The electron-donating hydroxyl group of **33** led to a slight improvement (EC_50_ = 390 and 206 nM), and its elongated *p*-methanol variant **34** also showed promising activity for both isoforms (EC_50_ = 278 and 133 nM). The bulkier dimethylamine group (**35**) was the first compound that showed an improvement related to reference GSK3987 (**3**), reaching lower EC_50_ values for both isoforms, namely, 71 nM for LXRα and 91 nM for LXRβ. We therefore decided to explore bulkier groups.

Our initial attempt with an amide group (**36**) completely abolished activation. Compound **37**, however, an inverted and constrained amide, displayed significant activity, though with a higher LXRβ potency (EC_50_ = 84 nM). The structurally similar benzodioxole group in **38** also displayed good agonistic activity, with almost equal values for both isoforms (EC_50_ of 42 and 45 nM for LXRα and β, respectively). Substitution of different positions of the ring, the 3,5-dimethoxy derivative **39**, led to a significant loss for both isoforms. These results suggested that the pocket could accommodate larger groups, as in **35** or **38**, but there could be a dependency on specific interactions.

When structurally comparing GSK3987 (**3**) to AZ876 (**4**) and their binding mode from the cocrystallized structures (Figure 1), the *N*-phenylpiperidine residue of **4** occupies the same pocket as the 4-methoxyphenyl substituent of **3**. Therefore, we explored groups with similar saturated cyclic substituents at the phenyl ring. Indeed, the *N*-phenylpiperidine substituent (compound **40**) resulted in a remarkable profile, combining high potency for LXRα (42 nM) and more than 6.3x selectivity over LXRβ (266 nM). Closely related groups morpholine and thiomorpholine (**41** and **42**) also displayed good agonistic activity on LXRα (35 and 50 nM) but showed increased activity for LXRβ (106 and 123 nM) and therefore reduced selectivity.

When continuing to explore cyclic aliphatic groups at the end of the phenyl ring, we prepared phenylpiperazine analogue **43**. It lost significant activity on LXRα (1253 nM), but surprisingly retained some potency in LXRβ (283 nM). Methylpiperazine analogue **44** showed no relevant activity or selectivity, and Boc-substitution at the end of the residue (**45**) abolished all activity, possibly due to steric restrictions. Yet, deprotected 4-aminopiperidine compound **46** was over 5 times more potent on LXRβ (120 nM) than on LXRα (630 nM). Overall, selectivity between α and β could be achieved through modulation of the nature of the hydrogen bond groups occupying the deeper parts of the ligand binding pocket.

Finally, simple modifications in the aminopiperidine moiety to explore the size of the group, namely, compounds **47** and **48**, displayed the same selectivity preference for LXRβ. Remaining modifications in the piperazine residue (**49** and **50**), with the introduction of an acyl group, showed good agonistic potency, with high α-selectivity for **50**.

#### 2.3.1 Combination of R^1^ and R^2^ Residues

Based on the information gathered from SAR analysis of both R^1^ and R^2^ residues, we aimed to evaluate a maleimide-based compound, with the *N*-phenylpiperazine at R^2^ and the *tert*-butyl moiety at R^1^, making it an analog of AZ876 (**4**). Indeed, compound **51** displayed a higher potency for LXRα (36 nM), and a 4-fold selectivity over LXRβ (156 nM, Table 4). This confirmed that the maleimide offers a suitable framework for the development of LXR ligands with the desired selectivity profile. As proof of concept, no substitution at R,^1^ even with the potency-inducing *N*-phenylpiperidine residue at **52**, showed no activity on both isoforms.

**Table 4:**
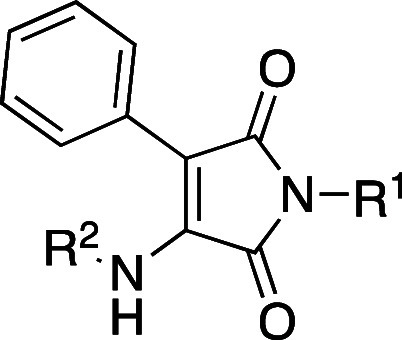

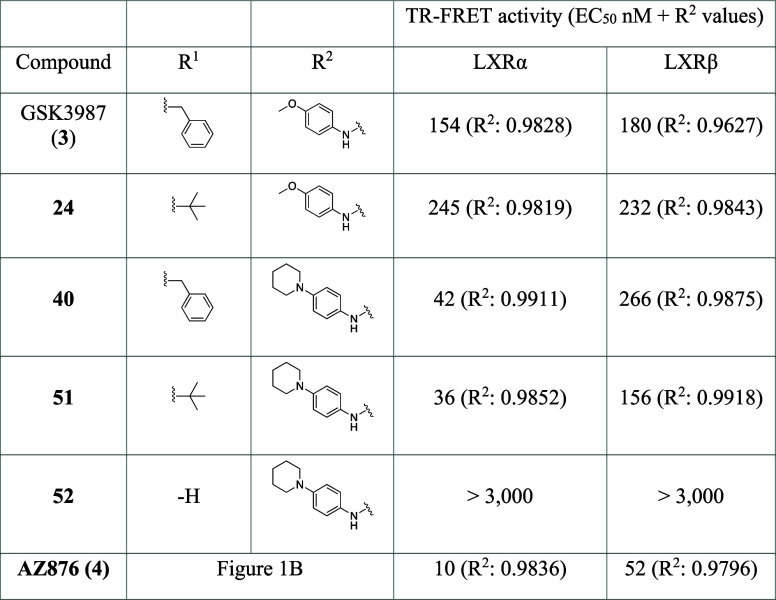
Activity of Maleimide Derivatives Determined with the TR-FRET Assay for LXRα and β Isoforms

### 2.4 Comparison of α and β Selective Compounds

**Figure 3:**
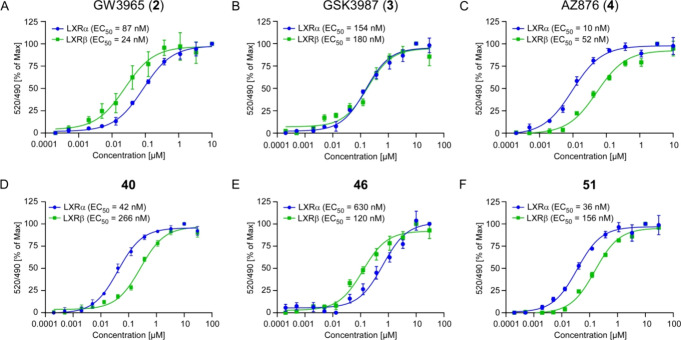
Novel LXRα agonist **40** combines potency for LXRα with selectivity over LXRβ in a biochemical activation assay. (A–F) FRET-based activation assays for LXRα and LXRβ under treatment with GW3965 (**2**) (A), GSK3987 (**3**) (B), AZ876 (**4**) (C), compound **40** (D), compound **46** (E), or compound **51** (F) (*n* = 4 replicates, data are presented as mean values ± SD, the experiments were repeated with similar results).

The dose–response curves and statistical analyses of the FRET assays illustrate the strong α-selectivity of compound **40** (Figure 3 and Supplementary Figure 1). While GW3965 (**2**) showed stronger activation of LXRβ and GSK3987 (**3**) showed no significant difference between both isoforms, compound **40** showed high potency for LXRα and displayed the strongest difference between LXRα and LXRβ activation (6.3*x*, in comparison AZ876 (**4**) = 5.2*x*).

**Figure 4:**
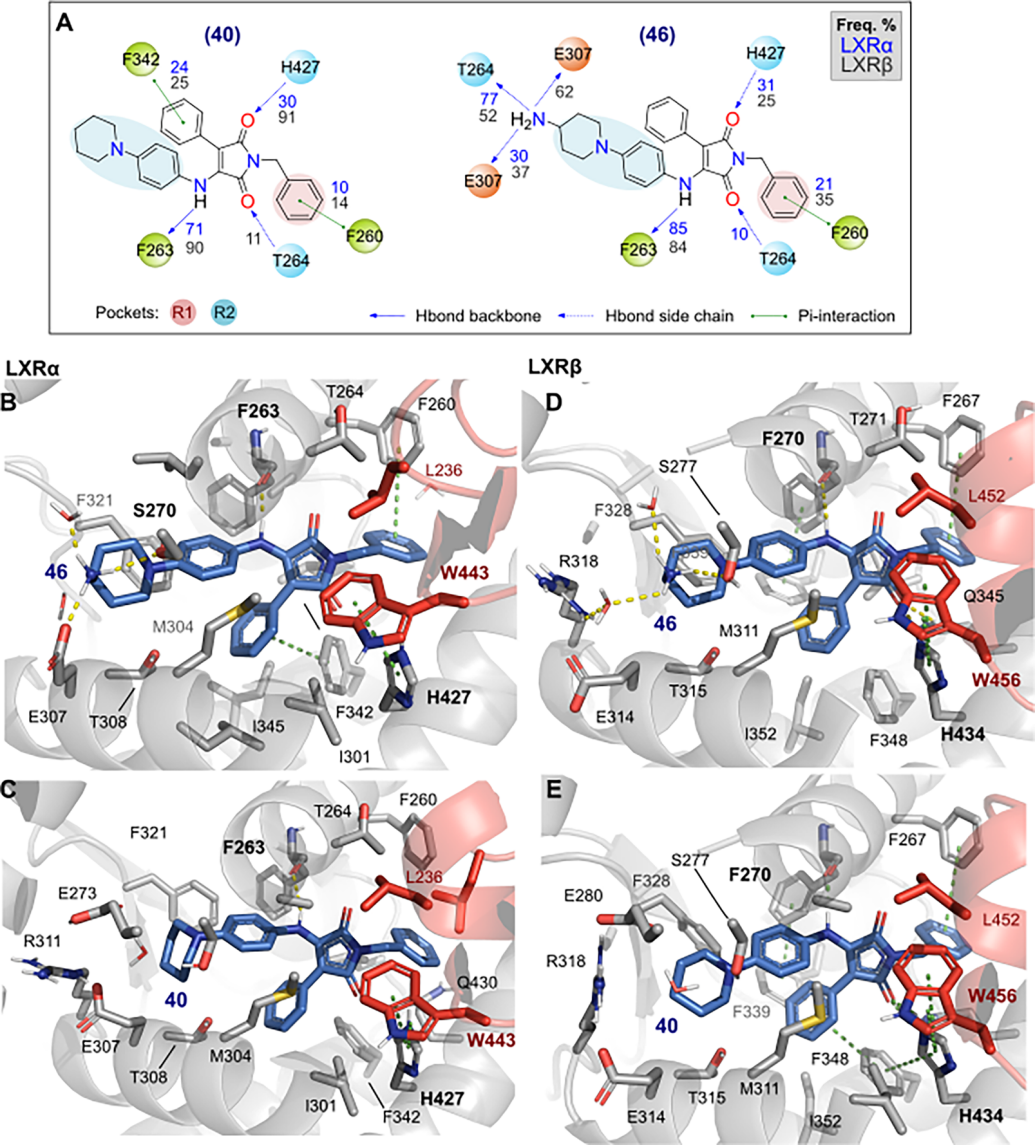
Novel LXR agonists stably interact with key residues in the ligand binding pocket, stabilizing the H12 region. (A) Protein–ligand interactions frequency represented over the calculated MD trajectory (5 × 500 ns) for LXRα (blue numbers) and LXRβ (gray numbers); polar contacts with amino acid side chains are depicted in dashed blue lines, while solid lines show interactions with their backbones, π-mediated interactions are depicted in green. Both the R1 (red) and R2 (light blue) regions of the agonist compounds are highlighted. (B–E) Relevant structures derived from clustering the MD trajectories for LXRα (B, C) and LXRβ (D, E) for both **40** and **46**.

Since the LXRβ-activating compounds **43** and **46**–**47** (Figure 3, Table 3) share a H-bond-donating group (basic group), we looked into possible interactions through MD simulations. The binding mode and most relevant interactions for compounds **40** and **46** are displayed in Figure 4. We found that both compounds occupy the LBD very similar to that of GSK3987 (**3**) in the crystal structure (Figure 1). The phenylpiperidine residue and amino-phenylpiperazine residues of the novel compounds can, however, occupy the back pocket at H1/H2. This analysis suggests that **46** can make specific hydrogen bond interactions, which possibly drive selectivity for the β-isoform.

### 2.5 Analyzing Cellular Activity of Novel LXRα Agonists

**Figure 5:**
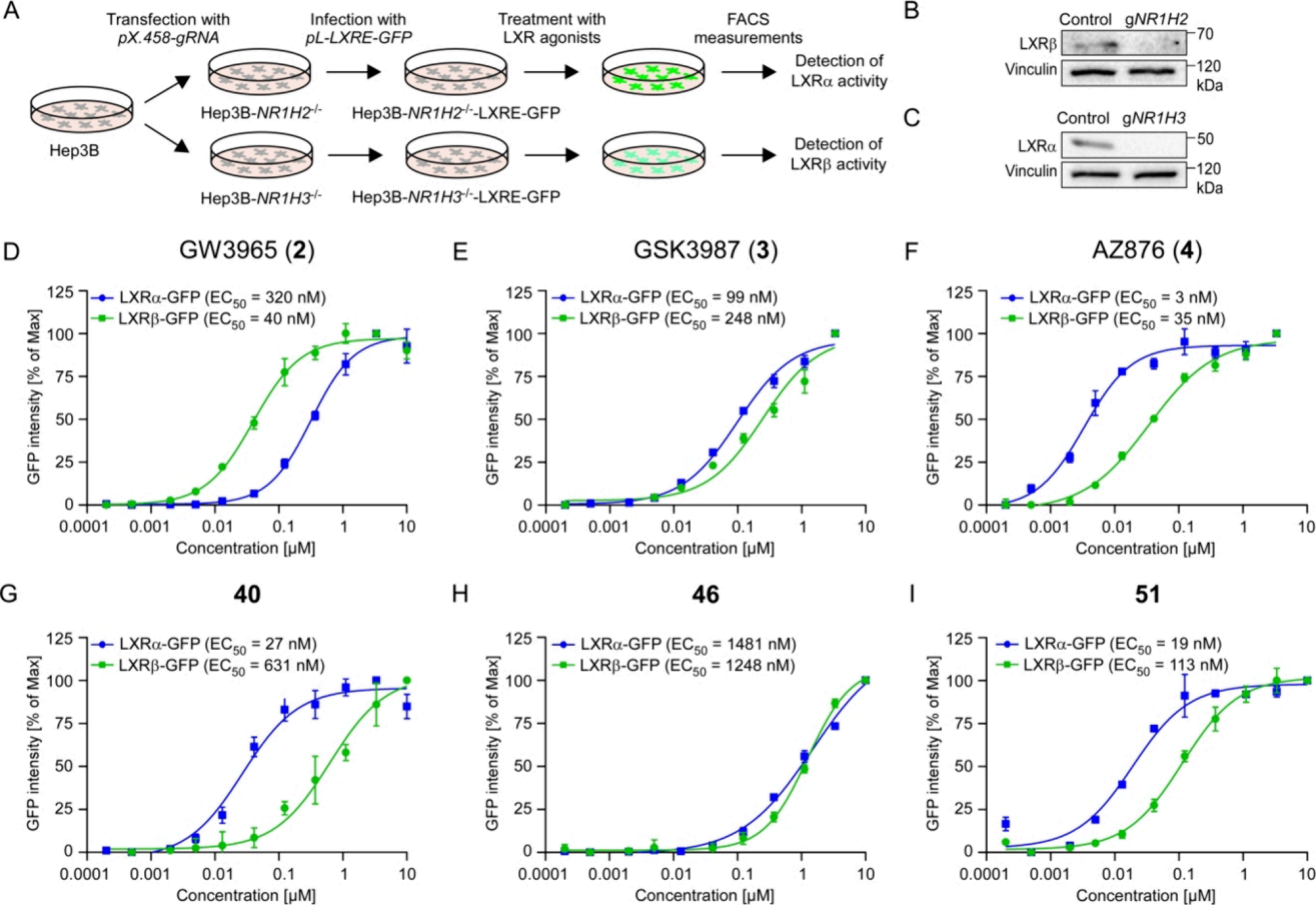
Novel LXRα agonist **40** shows strong cellular activity and selectivity for LXRα. (A) Development of isoform specific LXR reporter assays in Hep3B cells by CRISPR-mediated knockout of the LXRβ gene *NR1H2* or the LXRα gene *NR1H3* and integration of an LXRE-GFP reporter construct. (B, C) Representative Western blot analysis of Hep3B cells upon CRISPR-mediated knockout of *NR1H2* (B) or *NR1H3* (C) (cropped blot images, *n* = 3 independent experiments). Vinculin was used as a loading control. (D–I) LXRα and LXRβ specific reporter assays in Hep3B cells upon 48h of treatment with the LXR agonists GW3965 (**2**) (D), GSK3987 (**3**) (E), AZ876 (**4**) (F), compound **40** (G), compound **46** (H), or compound **51** (I) (quantification of GFP by FACS analysis, *n* = 3 biological replicates, data are presented as mean values ± SD, the experiments were repeated with similar results).

To determine the activity of **40**, **46**, and **51** in HCC cells, we established LXRα- and LXRβ-specific reporter assays in the well-established human HCC cell line Hep3B (Figure 5A). As a first step, we introduced a genetic knockout of the LXRα gene *NR1H3* or the LXRβ gene *NR1H2* in these cells using CRISPR/Cas9-mediated gene editing (Figure 5B,C). To measure the activity of the remaining isoform under treatment, we next stably integrated the LXR response element (LXRE) linked to GFP by lentiviral gene transfer. The quantification of the GFP intensity by FACS analysis thus enables us to define the intracellular activity of both LXR isoforms individually (Figure 5A). As proof-of-principle, GW3965 (**2**) showed a dose-dependent activation of both LXR isoforms; however, a much higher activity for LXRβ was observed (EC_50_ LXRα = 320 nM, EC_50_ LXRβ = 40 nM, Figure 5D).

To gain complete overview of relevant compounds, we tested GSK3987 (**3**) and AZ876 (**4**) in comparison to α- and β-selective novel maleimides **40**, **46**, and **51**. Apart from the β-selective compound **46**, all compounds showed cellular activity (Figure 5E–I).

While GSK3987 (**3**) displayed only a moderate activation of both LXR isoforms (EC_50_ LXRα = 99 nM, EC_50_ LXRβ = 248 nM, Figure 5E), the isothiazole AZ876 (**4**) triggered strong activation of LXRα (EC_50_ LXRα = 3 nM, EC_50_ LXRβ = 35 nM, Figure 5F). Similarly, the maleimide analogue of AZ876 (**4**), compound **51**, activates LXRα. Importantly, compound **40** also showed strong activation of LXRα and displayed the greatest difference between LXRα and LXRβ activation. In fact, the EC_50_ for LXRα (27 nM) was more than 23x lower than that for LXRβ (631 nM, Figure 5G).

To examine the specificity of compound **40** (and as a control compound **46**) toward other nuclear receptors, we assessed their activity in activating eight critical nuclear receptors with cellular reporter assays at saturating concentrations (10 μM). We chose nuclear receptors that are either known to directly or indirectly regulate metabolic processes in the liver, including vitamin D receptor (VDR), peroxisome proliferator-activated receptors α and γ (PPAR α and γ), estrogen receptor α and β (ERα and β) and farnesoid X receptor (FXR) or are relevant in drug metabolism such as aryl hydrocarbon receptor (AhR) and the mice constitutive androstane receptor (mCAR). Indeed, we found no relevant activation of these receptors by compound **40** (Supplementary Figure 2).

In summary, we showed that compound **40** enables a specific and strong activation of LXRα in HCC cells.

### 2.6 Treatment of Human HCC Cells

**Figure 6:**
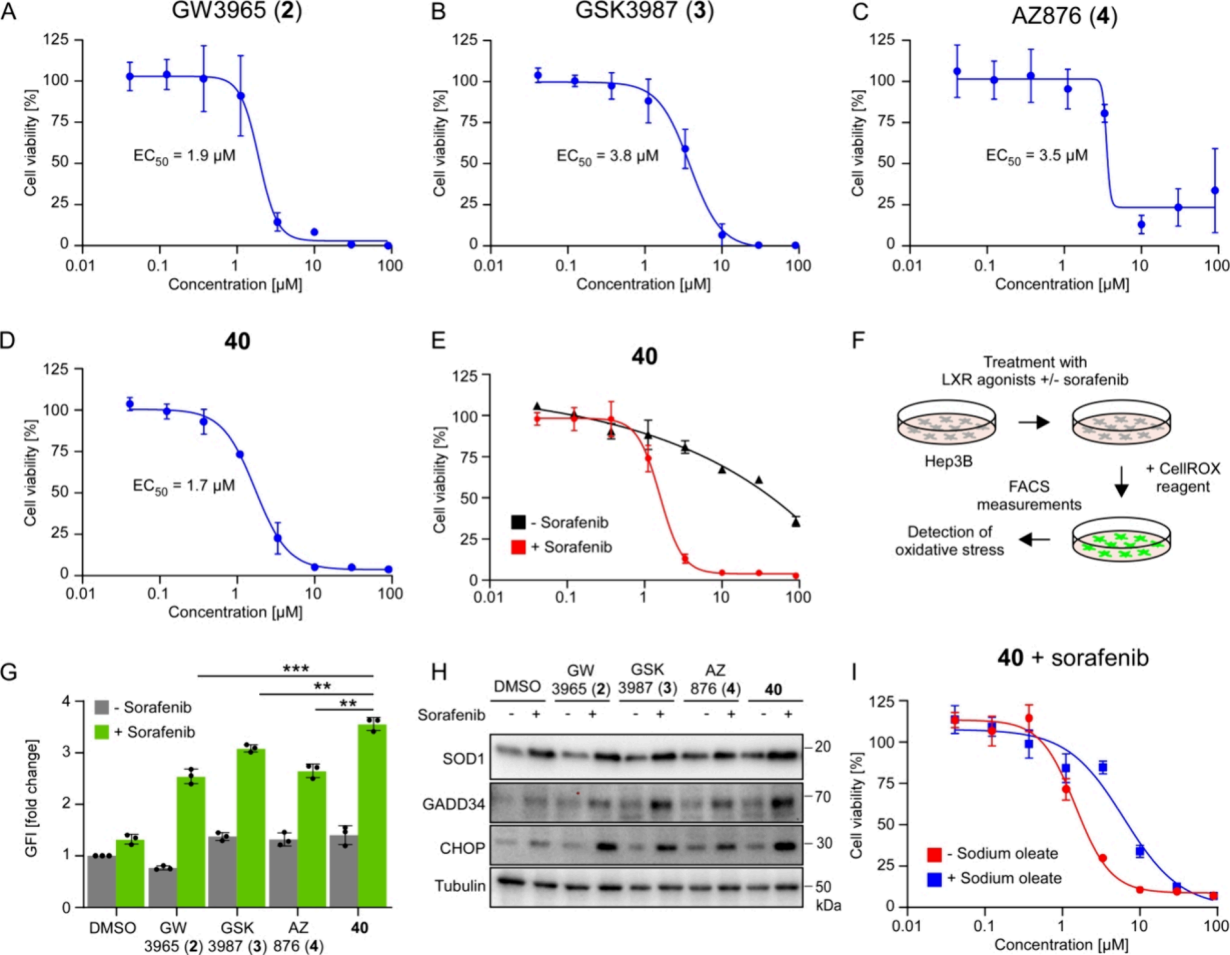
Novel LXRα agonist **40** triggers cell death, oxidative stress, and an ER stress response in HCC cells in combination with an DFG-out Raf inhibitor. (A–D) Cell viability analysis in Hep3B cells upon 5d of treatment with GW3965 (**2**) (A), GSK3987 (**3**) (B), AZ876 (**4**) (C), or compound **40** (D) with 2 μM sorafenib (quantification of viable CellTiter-Glo glow, *n* = 3 independent experiments, data are presented as mean values ± SD, the experiments were repeated with similar results). (E) Cell viability analysis in Hep3B cells upon 5d of treatment with compound **40** ± 2 μM sorafenib (quantification of viable cells by CellTiter-Glo, *n* = 3 biological replicates, data are presented as mean values ± SD, the experiments were repeated with similar results). (F) Detection of oxidative stress in human HCC cells using CellROX reagent. (G) Quantification of oxidative stress in Hep3B cells after 3 days of treatment with 3 μM of LXR agonists ± 1 μM sorafenib (data are presented as mean values ± SD of fold change GFI (green fluorescence intensity). *n* = 3 biological replicates, statistical significance was calculated using two-tailed Student′s *t* test, *** = *P* < 0.001, ** = *P* < 0.01, the experiment was repeated with similar results). (H) Representative Western blot analysis in Hep3B cells after 3 days of treatment with different LXR agonists ± 2 μM sorafenib (cropped blot images, *n* = 3 independent experiments). Tubulin was used as a loading control. (I) Cell viability analysis in Hep3B cells upon 5d of treatment with compound **40** and 2 μM sorafenib ± 200 μM sodium oleate (quantification of viable cells by CellTiter-Glo, *n* = 3 biological replicates, data are presented as mean values ± SD, the experiments were repeated with similar results).

To determine the therapeutic efficacy of compound **40** in HCC cells, we next tested its effect on Hep3B cell viability in combination with the DFG-out Raf inhibitor sorafenib (2 μM). As a comparison, we also tested the cell viability effects of GW3965 (**2**), GSK3987 (**3**), and AZ876 (**4**) during sorafenib therapy. In accordance with our previously described study,^6^ a 2 μM sorafenib monotherapy showed only a low therapeutic effect in Hep3B cells (Supplementary Figure 3) while a combination of GW3965 (**2**) and sorafenib results in a strong, dose-dependent reduction of cell viability (EC_50_ = 1.9 μM, Figure 6A).

Interestingly, GSK3987 (**3**) and AZ876 (**4**) showed lower therapeutic effects than GW3965 (**2**). In strong contrast, compound **40** was even more efficient than GW3965 (**2**) and strongly reduced the cell viability of Hep3B cells in combination with sorafenib (EC_50_ = 1.7 μM) (Figure 6B–D).

To see whether the therapeutic effect of compound **40** is dependent on Raf inhibition, we also performed cell viability assays without sorafenib therapy. Importantly, we found that monotherapy of compound **40** did not show a significant therapeutic effect (Figure 6E).

We previously described that our established lipotoxic therapy triggers oxidative stress and an ER stress response, which finally leads to apoptosis in tumor cells.^6^ Therefore, we next tested oxidative stress induction of compound **40** with or without sorafenib in comparison to GW3965 (**2**), GSK3987 (**3**), and AZ876 (**4**) in Hep3B cells using the established CellROX reagent (Figure 6F). While the agonists do not significantly induce oxidative stress as a monotherapy, we found strongly increased oxidative stress induction by all tested compounds in combination with sorafenib. Particularly, in a side-by-side comparison, compound **40** was more efficient in inducing oxidative stress than GW3965 (**2**), GSK3987 (**3**), and AZ876 (**4**) (Figure 6G).

We next analyzed the activity of the oxidative stress response factor superoxide dismutase 1 (SOD1) and the ER stress response factors GADD34 and CHOP (GADD153) upon treatment with compound **40**, GW3965 (**2**), GSK3987 (**3**), and AZ876 (**4**) with or without sorafenib. We found that activation of oxidative stress and ER stress response factors was dependent on the agonist, while compound **40** triggers a strong induction of all factors in combination with sorafenib (Figure 6H).

To determine whether the therapeutic effect of compound **40** is indeed due to an accumulation of saturated fatty acids and, as such, due to an altered ratio of saturated fatty acids to monounsaturated fatty acids, we replenished Hep3B cells with the monounsaturated fatty acid salt sodium oleate during treatment. Indeed, this high amount of monounsaturated fatty acids strongly diminished the therapeutic efficacy of the compound **40**-sorafenib combination (Figure 6I).

In summary, compound **40** shows, in combination with Raf inhibition, a strong lipotoxic effect and promising therapeutic efficacy in HCC cells.

### 2.7 Analyzing the Therapeutic Efficacy of Novel LXRα Agonists in Untransformed Cells and Liver Cancer Organoids

**Figure 7:**
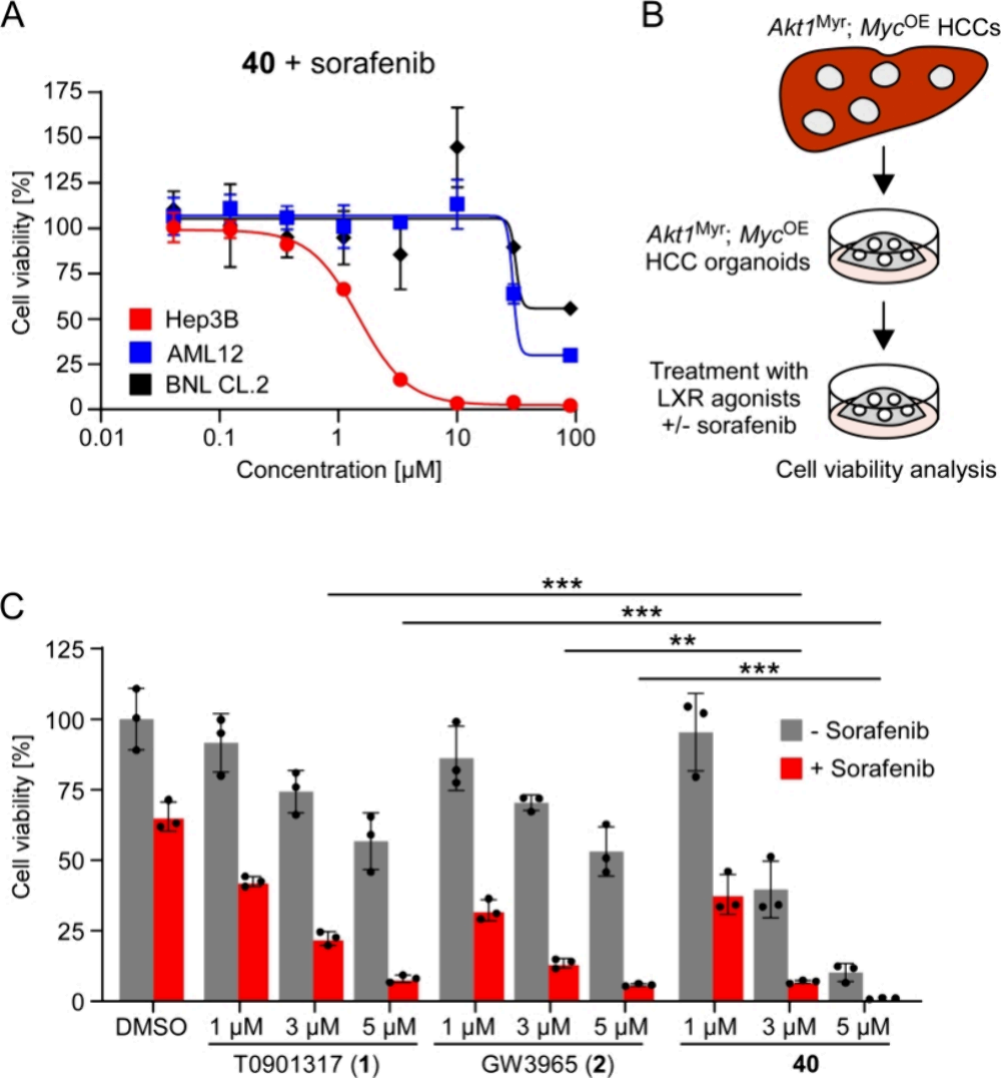
Novel LXRα agonist **40** is highly efficient in HCC organoids in combination with an DFG-out Raf inhibitor but not in healthy liver cells. (A) Cell viability analysis in Hep3B, AML12, and BNL CL.2 cells upon 5d of treatment with compound **40** and 2 μM sorafenib (quantification of viable cells by CellTiter-Glo, *n* = 3 biological replicates, data are presented as mean values ± SD, the experiments were repeated with similar results). (B) Generation and treatment of murine *Akt1*^Myr^; *Myc*^OE^ HCC organoids. (C) Cell viability analysis in *Akt1*^Myr^; *Myc*^OE^ organoids upon 5d of treatment with T0901317 (**1**), GW3965 (**2**) or compound **40** with and without 4 μM sorafenib (quantification of viable cells by CellTiter-Glo, *n* = 3 biological replicates, data are presented as mean values ± SD, statistical significance was calculated using two-tailed Student′s *t* test, *** = *P* < 0.001, ** = *P* < 0.01, the experiment was repeated with similar results).

To further determine the therapeutic potential of compound **40**, we analyzed the effect of a combinatorial lipotoxic therapy with this agonist in untransformed liver cells. We applied the well-established untransformed liver cell lines AML12 and BNL CL.2 and subjected them to treatment with **40** in combination with sorafenib. In contrast to Hep3B cells, untransformed liver cells show only very low sensitivity toward this combinatorial therapy (Figure 7A), indicating a strong therapeutic window for this therapy.

Finally, we analyzed the efficacy of compound **40** in preclinical treatment studies in three-dimensional liver cancer organoid cultures. HCC organoids driven by myristoylated *Akt1* and overexpressed *Myc* (*Akt1*^Myr^; *Myc*^OE^) were isolated from murine liver tumors and subjected to monotherapies of sorafenib, compound **40**, or a combination thereof (Figure 7C). The therapeutic outcome was side-by-side compared with combinatorial therapies comprising the established agonists T0901317 (**1**) and GW3965 (**2**). While both established LXR agonists revealed a dose-dependent response in combination with sorafenib, GW3965 (**2**) showed in general a higher efficacy than T0901317 (Figure 7C). However, this effect was outperformed by compound **40**, which showed further improved therapeutic efficacy in combination with sorafenib (Figure 7B). Overall, these data show a strong therapeutic potential of compound **40** for the treatment of liver cancer and corroborate the strategy of using LXRα agonists for pharmacologically induced lipotoxicity.

## 3 Conclusions

In this work, we described the design, synthesis, and evaluation of a series of novel maleimide derivatives and generated potent and selective LXRα agonists for lipotoxic cancer therapies. All compounds were biochemically evaluated with TR-FRET assays, which allowed us to determine structure–activity relationships. Through iterative modifications of different substituents in the maleimide core, we found important structural features driving the potency and α/β selectivity.

We identified the key compounds **40** and **51** as α-selective and **46** as β-selective. The isoform selectivity of these compounds was investigated by molecular dynamics studies, indicating the importance of interactions at the back of the H1/H2 pocket. Intracellular activity for LXR was confirmed by isoform-specific reporter assays in Hep3B cells, and compound **40** displayed the highest distinction between LXRα and LXRβ, even when compared to reference compound AZ876 (**4**). To determine the selectivity of compound **40** with regard to other nuclear receptors, the activity of **40** was measured in reporter assays for eight closely related nuclear receptors and displayed no relevant activation on any of them. Importantly, compound **40** also showed strong therapeutic efficacy in combination with the DFG-out Raf-inhibitor sorafenib in HCC cells and displayed an increased ability to induce oxidative stress and an ER stress response with concomitant Raf inhibition. Finally, **40** showed further improved therapeutic efficacy on liver cancer organoids when combined with sorafenib and low cytotoxic effects in normal liver cells.

Overall, the results obtained by this study demonstrate not only the potential for lipotoxic cancer treatment but also the advantages of α-selective LXR agonists. We identified important features for protein interaction to obtain selectivity within isoforms and were able to identify highly potent compounds, and our frontrunner compound **40** displayed improved cellular activity when compared to reference compounds. We believe that the structural information acquired through the novel maleimides can contribute to the development of LXRα agonists that can be applicable to the treatment of HCC in the clinic.

## 4 Experimental Section

### 4.1 Chemistry

All starting materials, reagents, and (anhydrous) solvents were commercially available and were used without further purification. ^1^H and ^13^C NMR spectra were obtained with a Bruker Avance 400 spectrometer. The spectra were calibrated against the residual proton peak of the used deuterated solvent. Chemical shifts (δ) are reported in parts per million. HPLC analysis was carried out on an Agilent 1100 Series LC with Phenomenex Luna C8 column (150 × 4.6 mm, 5 μm) and detection was performed with a UV DAD at 254 and 230 nm wavelength. Elution was carried out with the following gradient: 0.01 M KH_2_PO_4_, pH 2.30 (solvent A), MeOH (solvent B), 40% B to 85% B in 8 min, 85% B for 5 min, 85% to 40% B in 1 min, 40% B for 2 min, stop time 16 min, flow 1.5 mL/min. Thin-layer-chromatography (TLC) analyses were performed on fluorescent silica gel 60 F254 plates (Merck) and visualized under UV illumination at 254 and 366 nm. HR-MS measurements were performed at the mass spectrometry department, Institute of Organic Chemistry, Eberhard-Karls-University Tübingen. Column chromatography was performed on Davisil LC60A 20–45 μm silica from Grace Davison and Geduran Si60 63–200 μm silica from Merck for the precolumn using an Interchim PuriFlash 430 automated flash chromatography system. All synthesized final compounds had a purity of at least 95% as determined by HPLC.

#### 4.1.1 General Procedure for Synthesis of Maleimides

The functionalized amide (1 equiv) and diethyloxalate (1.1 equiv) were added to THF. Potassium *tert*-butoxide (2 equiv) was added slowly to the reaction mixture, leading to a change of color to bright yellow. The reaction was stirred at room temperature overnight. Work up was made by adding 1 M solution of HCl followed by extraction with EtOAc. The organic phase was dried over Na_2_SO_4_ and evaporated to afford hydroxylated maleimide as a yellow solid.

The hydroxylated maleimide (1 equiv) was dissolved in a mixture of DCM and DMF (80:20), the flask was put on an ice bath, and oxalyl chloride was added slowly (2.5 equiv), leading to strong bubbling. The reaction mixture was stirred at room temperature overnight and monitored via TLC (1:5 EtOAc/Hexane). The reaction mixture was directly adsorbed in Celite, and the product was purified by flash column chromatography (0–20% EA/PE). The fractions were evaporated to afford the chlorinated product as a white, crystalline solid.

#### 4.1.2 General Procedure for Substitution Reaction with Anilines

To a round-bottom flask, the chlorinated maleimide (1 equiv) and the aniline (1.2 equiv) were added to dioxane. To this reaction mixture, triethylamine (2 equiv) was added, and the reaction was stirred at reflux temperature until all starting materials had been consumed. After the reaction had finished, the remaining dioxane was evaporated and the crude product was directly adsorbed into Celite and purified on flash chromatography (0–50% EA/PE).

##### 1-Benzyl-3-((4-methoxyphenyl)amino)-4-phenyl-1*H*-pyrrole-2,5-dione (**3**)

Yield: 81%, yellow powder. ^1^H NMR (400 MHz, DMSO) δ 9.57 (s, 1H), 7.38–7.27 (m, 5H), 7.11–7.02 (m, 3H), 6.87 (d, 2H, *J* = 6.9 Hz), 6.67 (d, 2H *J* = 9.0 Hz), 6.52 (d, 2H. *J* = 9.0 Hz), 4.69 (s, 2H), and 3.60 (s, 3H). 13C NMR (100 MHz, DMSO) δ 172.0, 168.0, 156.4, 138.9, 137.7, 130.8, 130.3, 129.7, 129.0, 127.8, 127.3, 126.9, 124.0, 113.5, 100.3, 55.7, 41.2. IR (ATR) (cm^–1^) ν_max_: 3309, 1699, 1641, 1545. ESI-HRMS [M + H]^+^ calculated, 385,15467, found, 385,15539 HPLC: *t*_*ret*_ 12.980 min, purity (254 nm) 100%, (230 nm) 98.9%.

##### 1-(4-Fluorobenzyl)-3-((4-methoxyphenyl)amino)-4-phenyl-1*H*-pyrrole-2,5-dione (**16**)

Yield: 84%, orange powder. ^1^H NMR (400 MHz, DMSO) δ 9.58 (s, 1H), 7.42–7.30 (m, 2H), 7.27–7.13 (m, 2H), 7.11 – 6.99 (m, 3H), 6.87 (d, 2H, *J* = 6.9 Hz), 6.67 (d, 2H, *J* = 9.6 Hz), 6.52 (d, 2H. *J* = 9.0 Hz), 4.69 (s, 2H), 3.60 (s, 3H). ^13^C NMR (100 MHz, DMSO) δ 171.5, 167.5, 161.5 (d, *J*_*CF*_ = 243 Hz), 156.0, 138.5, 133.4, 130.4, 130.0, 129.6, 129.3, 127.0, 126.5, 124.0, 115.5, 115.3, 113.1, 100.0, 55.3, 55.0. IR (ATR) (cm^–1^) ν_max_: 3304, 2921, 1689, 1627, 1509. ESI-HRMS [M + H]^+^ calculated 403,14525, found, 403,14688. HPLC: *t*_*ret*_ 9.246 min, purity (254 nm) 98.2%, (230 nm) 98.0%.

##### 3-((4-Methoxyphenyl)amino)-1-(4-methylbenzyl)-4-phenyl-1*H*-pyrrole-2,5-dione (**17**)

Yield: 11%, orange powder. ^1^H NMR (400 MHz, CDCl_3_) δ 7.35, (d, 2H, *J* = 7.9 Hz), 7.15–7.06 (m, 6H), 6.97 (d, 2H, *J* = 6.6 Hz) 6.58 (d, 2H, *J* = 9.2 Hz), 6.54 (d, 2H, *J* = 9.2 Hz), 4.73 (s, 2H), 3.69 (s, 3H), 2.33 (s, 3H). ^13^C NMR (100 MHz, CDCl_3_) δ: 172.1, 168.3, 157.0, 137.7, 136.9, 133.8, 129.8, 129.5, 129.5, 129.4, 128.9, 127.3, 127.2, 123.4, 113.6, 101.4, 55.6, 41.6, 21.3. IR (ATR) (cm^–1^) ν_max_: 3299, 2921, 1754, 1689, 1626. ESI-HRMS [M + H]^+^ calculated 399,17032, found, 399,17285. HPLC *t*_*ret*_ 9.569 min, purity (254 nm) 98.6%, (230 nm) 97.0%.

##### 3-((4-Methoxyphenyl)amino)-4-phenyl-1-(4-(trifluoromethyl)benzyl)-1*H*-pyrrole-2,5-dione (**18**)

Yield: 48%, orange powder. ^1^H NMR (400 MHz, DMSO) δ 9.62 (s, 1H), 7.73 (d, 2H, *J* = 8.1 Hz), 7.55 (d, 2H, *J* = 8.0 Hz), 7.10–7.00 (m, 4H), 6.89 (d, 2H, *J* = 7.0 Hz), 6.68 (d, 2H, *J* = 8.8 Hz), 6.53 (d, 2H, *J* = 8.8 Hz), 4.79 (s, 2H), 3.61 (s, 3H). ^13^C NMR (100 MHz, DMSO) δ 171.4, 167.5, 155.9, 141.9, 138.5, 130.3, 129.8, 129.2, 128.1, 128.1, 126.8, 126.4, 125.5 (q, *J*_*CF*_ = 3.8 Hz), 123.5, 122.9, 113.0, 99.9, 55.2, 40.3. IR (ATR) (cm^–1^) ν_max_: 3311, 1695, 1634, 1509. ESI-HRMS [M + H]^+^ calculated 453,14205, found, 453,1425. HPLC: *t*_*ret*_ 9.801 min, purity (254 nm) 99.45%, (230 nm) 97.8%.

##### 1-(4-Methoxybenzyl)-3-((4-methoxyphenyl)amino)-4-phenyl-1*H*-pyrrole-2,5-dione (**19**)

Yield: 67%, orange powder. ^1^H NMR (400 MHz, DMSO) δ 9.54 (s, 1H), 7.25 (d, 2H, *J* = 8.7 Hz), 7.11–7.02 (m, 3H), 6.92–6.86 (m, 4H), 6.67 (d, 2H, *J* = 9.0 Hz), 6.5 (d, 2H, *J* = 9.0 Hz), 4.61 (s, 2H), 3.73 (s, 3H), 3.6 (s, 3H). ^13^C NMR (100 MHz, DMSO) δ: 171.5, 167.4, 158.6, 155.9, 138.4, 130.3, 129.8, 129.2, 129.0, 126.8, 126.4, 123.5, 113.9, 113.0, 99.7, 55.2, 55.01. IR (ATR) (cm^–1^) ν_max_: 3300, 2967, 1755, 1690, 1626. ESI-HRMS [M + H]^+^ calculated 415,16523, found, 415,1689. HPLC: *t*_*ret*_ 9.005 min, purity (254 nm) 99.8%, (230 nm) 98.6%.

##### 1-(4-(*tert*-Butyl)benzyl)-3-((4-methoxyphenyl)amino)-4-phenyl-1*H*-pyrrole-2,5-dione *(***20***)*

Yield: 86%, orange powder. ^1^H NMR (400 MHz, DMSO) δ 9.56 (s, 1H), 7.37 (d, 2H, *J* = 8.3 Hz), 7.24 (d, 2H, *J* = 8.3 Hz), 7.11–7.02 (m, 3H), 6.88 (d, 2H, *J* = 7.0 Hz), 6.67 (d, 2H, *J* = 8.9 Hz), 6.53 (d, 2H, *J* = 8.9 Hz), 4.65 (s, 2H), 3.60 (s, 3H), 1.26 (s, 9H). ^13^C NMR (100 MHz, DMSO) δ: 171.5, 167.5, 155.9, 149.7, 138.4, 134.2, 130.4, 129.8, 129.2, 127.3, 126.8, 126.3, 125.3, 123.5, 113.0, 99.7, 55.2, 34.2, 31.1. IR (ATR) (cm^–1^) ν_max_: 3288, 2952, 1759, 1692, 1627. ESI-HRMS [M + H]^+^ calculated 441,21727, found, 441,22135. HPLC: *t*_*ret*_ 10.850 min, purity (254 nm) 99.8%, (230 nm) 99.7%.

##### 3-((4-Methoxyphenyl)amino)-4-phenyl-1-(pyridin-4-ylmethyl)-1*H*-pyrrole-2,5-dione (**21**)

Yield: 43%, light brown powder. ^1^H NMR (400 MHz, DMSO) δ 9.63 (s, 1H), 8.55 (d, 2H, *J* = 6.0 Hz), 7.32 (d, 2H, *J* = 6.0 Hz), 7.10–7.03 (m, 3H), 6.90 (d, 2H, *J* = 6.8 Hz), 6.70 (d, 2H, *J* = 8.9 Hz), 6.54 (d, 2H, *J* = 8.9 Hz), 4.73 (s, 2H), 3.61 (s, 3H). ^13^C NMR (100 MHz, DMSO) δ: 171.3, 167.5, 155.9, 149.8, 146.0, 138.6, 130.3, 129.8, 129.2, 126.8, 126.4, 123.6, 122.0, 113.0, 99.9, 55.2. IR (ATR) (cm^–1^) ν_max_: 3101, 2918, 1763, 1699, 1634. ESI-HRMS [M + H]^+^ calculated, 386,14992, found, 386,1508. HPLC: *t*_*ret*_ 7.881 min, purity (254 nm) 96.5%, (230 nm) 98.6%.

##### 1-(Cyclohexylmethyl)-3-((4-methoxyphenyl)amino)-4-phenyl-1*H*-pyrrole-2,5-dione (**22**)

Yield: 77%. orange powder. ^1^H NMR (400 MHz, CDCl3) δ 7.13 (s, 1H), 7.06–7.00 (m, 3H), 6.94–6.92 (d, 2H, *J* = 6.8 Hz), 6.54–6.47 (m, 4H), 3.63 (s, 3H), 3.36 (d, 2H, *J* = 7.0 Hz), 1.66–1.58 (m, 5H), 1.22 – 1.11 (m, 4H), 0.97–0.89 (m, 2H). ^13^C NMR (100 MHz, CDCl3) δ: 172.7, 168.9, 156.9, 136.7, 129.85, 129.6, 129.5, 127.3, 127.1, 123.3, 113.6, 101.0, 55.6, 44.4, 37.3, 30.9, 26.4, 25.8. IR (ATR) (cm^–1^) ν_max_: 3295, 2925, 1754, 1684, 1632. ESI-HRMS [M + H]^+^ calculated 391,20162, found, 391,20335. HPLC: *t*_*ret*_ 10.728 min, purity (254 nm) 99.7%, (230 nm) 99.1%.

##### 1-(Cyclopropylmethyl)-3-((4-methoxyphenyl)amino)-4-phenyl-1*H*-pyrrole-2,5-dione *(***23**)

Yield: 60%, yellow powder. ^1^H NMR (400 MHz, CDCl_3_) δ 7.12 (s, 1H), 7.06–7.01 (m, 3H), 6.93 (d, 2H, *J* = 6.8 Hz), 6.55–6.47 (m, 4H), 3.63 (s, 3H), 3.39 (d, 2H, *J* = 7.1 Hz), 1.15–1.11 (m, 1H), 0.44 (d, 2H, *J* = 7.3 Hz), 0.30 (d, 2H, *J* = 4.6 Hz). ^13^C NMR (100 MHz, CDCl_3_) δ: 172.5, 168.7, 157.0, 136.9, 129.8, 129.6, 129.5, 127.3, 127.1, 123.3, 113.7, 101.3, 55.6, 43.0, 10.7, 4.0. IR (ATR) (cm^–1^) ν_max_: 3289, 2930, 1755, 1692, 1630. ESI-HRMS [M + H]^+^ calculated 349,15467, found, 349,15582. HPLC *t*_*ret*_ 9.235 min, purity (245 nm) 98.5%, (230 nm) 97.5%.

##### 1-(*tert*-Butyl)-3-((4-methoxyphenyl)amino)-4-phenyl-1*H*-pyrrole-2,5-dione (**24**)

Yield: 38%, yellow powder. ^1^H NMR (400 MHz, CDCl3) δ 7.05–7.00 (m, 4H), 6.91–6.89 (m, 2H), 6.52–6.45 (m, 4H), 3.62 (s, 3H), 1.60 (s, 9H). ^13^C NMR (100 MHz, CDCl3) δ: 173.8, 169.3, 156.8, 136.5, 129.9, 129.7, 129.6, 127.3, 127.0, 123.3, 113.6, 101.0, 57.6, 55.6, 29.3. IR (ATR) (cm^–1^) ν_max_: 3289, 2930, 1755, 1692, 1630. ESI-HRMS [M + H]^+^ calculated 351,17032, found, 351,1718. HPLC: *t*_*ret*_ 9.905 min, purity (254 nm) 99.9%, (230 nm) 99.8%.

##### 3-((4-Methoxyphenyl)amino)-4-phenyl-1*H*-pyrrole-2,5-dione (**25**)

Yield: 70%, red powder. ^1^H NMR (400 MHz, DMSO) δ 10.66 (s, 1H), 9.3 (s, 1H), 7.09–7.02 (m, 3H), 6.86 (d, 2H, *J* = 7.0 Hz), 6.64 (d, 2H, *J* = 8.7 Hz), 6.51 (d, 2H, *J* = 8.7 Hz), 3.60 (s, 3H). ^13^C NMR (100 MHz, DMSO) δ: 172.9, 169.0, 155.7, 138.4, 130.5, 130.0, 129.3, 126.8, 126.3, 123.3, 113.0, 100.8, 55.2. IR (ATR) (cm^–1^) ν_max_: 3278, 3048, 1755, 1688, 1624. ESI-HRMS [M + H]^+^ calculated, 295,10772, found, 295,10936. HPLC *t*_*ret*_ 7.184 min, purity (254 nm) 98.9%, (230 nm) 98.6%.

##### 1-Benzyl-3-(4-methoxyphenoxy)-4-phenyl-1*H*-pyrrole-2,5-dione (**26**)

Yield: 38%, yellow powder. ^1^H NMR (400 MHz, CDCl_3_) δ 7.82 – 7.80 (m, 2H), 7.32–7.21 (m, 8H), 6.90 (d, 2H, *J* = 9.0 Hz), 6.73 (d, 2H, *J* = 9.0 Hz), 4.63 (s, 2H), 3.70 (s, 3H). ^13^C NMR (10 MHz, CDCl_3_) δ: 169.4, 165.1, 156.8, 149.1, 148.6, 136.4, 129.6, 129.2, 128.8, 128.6, 128.1, 127.7, 119.1, 114.7, 77.4, 55.8, 41.7. IR (ATR) (cm^–1^) ν_max_: 2950, 2851, 1698, 1636. ESI-HRMS [M + H]^+^ calculated 386,13868, found, 386,14167. HPLC *t*_*ret*_ 10.118 min, purity (254 nm) 99.6%, (230 nm) 98.9%.

##### 1-Benzyl-3,4-diphenyl-1*H*-pyrrole-2,5-dione (**27**)

^1^H NMR (400 MHz, CDCl_3_) Yield: 85%, yellow powder. δ 7.41–7.40 (m, 6H), 7.31–7.25 (m, 9H), 6.76 (d, 2H, *J* = 8.9 Hz), 4.75 (s, 2H). ^13^C NMR (100 MHz, CDCl_3_) δ: 170.5, 136.6, 136.3, 130.1, 123.0, 129.0, 128.8, 128.7, 128.7, 128.0, 42.2. IR (ATR) (cm^–1^) ν_max_: 2924, 1695, 1596. ESI-HRMS [M + H]^+^ calculated 385,15467, found, 385,15483. HPLC: *t*_*ret*_ 10.252 min, purity (254 nm) 100%, (230 nm) 93.13%.

##### 1-Benzyl-3-((4-methoxybenzyl)amino)-4-phenyl-1*H*-pyrrole-2,5-dione (**28)**

Yield: 45%, yellow powder. ^1^H NMR (400 MHz, CDCl3) δ 7.34–7.19 (m, 10H), 6.90 (d, 2H, *J* = 8.4 Hz), 6.73 (d, 2H, *J* = 8.4 Hz), 5.43 (s, 1H), 4.12 (d, 2H, *J* = 5.9 Hz), 3.71 (s, 3H), 3.28 (d, 2H), 1.62–1.53 (m, 6H), 1.19–1.05 (m, 4H), 0.93–0.84 (m, 2H). ^13^C NMR (100 MHz, CDCl3) δ: 172.0, 167.7, 159.5, 141.8, 136.9, 130.6, 130.1, 129.2, 128.9, 128.8, 128.7, 128.1, 127.8, 127.6, 114.3, 100.3, 55.4, 48.1, 41.8. IR (ATR) (cm^–1^) ν_max_: 3321, 1760, 1701, 1636. ESI-HRMS [M + H]^+^ calculated, 399,17032, found, 399,17257. HPLC: *t*_*ret*_ 9.482 min, (254 nm), 99.7%; (230 nm) 98.8%.

##### 1-Benzyl-3-phenyl-4-(phenylamino)-1*H*-pyrrole-2,5-dione (**29**)

Yield: 75%, yellow powder. ^1^H NMR (400 MHz, DMSO) δ 9.69 (s, 1H), 7.38–7.28 (m, 5H), 7.12–7.04 (m, 3H), 6.98–6.86 (m, 5H), 6.73 (d, 2H, *J* = 7.5 Hz), 4.71(s, 2H). ^13^C NMR (100 MHz, DMSO) δ: 171.4, 167.6, 137.7, 137.4, 137.1, 129.8, 129.2, 128.6, 127.7, 127.4, 126.9, 126.7, 123.6, 121.7, 101.6, 40.8. IR (ATR) (cm^–1^) ν_max_: 3282, 1763, 1697, 1632. ESI-HRMS [M + Na]^+^ calculated, 377,12605, found, 377,12663. HPLC: *t*_*ret*_ 9.604 min, purity (254 nm) 99.9%, (230 nm) 97.6%

##### 1-Benzyl-3-((4-chlorophenyl)amino)-4-phenyl-1*H*-pyrrole-2,5-dione (**30**)

Yield: 79%, orange powder. ^1^H NMR (400 MHz, DMSO) δ 9.75 (s, 1H), 7.34–7.28 (m, 5H), 7.13–7.12 (m, 3H), 7.01–6.95 (m, 4H), 6.73 (d, 2H, *J* = 7.5 Hz), 4.70 (s, 2H). ^13^C NMR (100 MHz, DMSO) δ: 171.3, 167.5, 137.5, 137.0, 136.5, 129.6, 129.2, 128.6, 127.5, 127.4, 127.1, 123.1, 102.7, 40.8. IR (ATR) (cm^–1^) ν_max_: 3296, 1758, 1696, 1643. ESI-HRMS [M + H]^+^ calculated 389,10513, found, 389,10886. HPLC *t*_*ret*_ 9.604 min, purity: (254 nm) 98.9%, (230 nm) 97.6%.

##### 1-Benzyl-3-((4-fluorophenyl)amino)-4-phenyl-1*H*-pyrrole-2,5-dione (**31**)

Yield: 75%. ^1^H NMR (400 MHz, CDCl_3_) δ 7.37 (d, 2H, *J* = 7.1 Hz), 7.29–7.22 (m, 3H), 7.12 (s, 1H), 7.09–7.02 (m, 3H), 6.91 (d, 2H, *J* = 7.2 Hz), 6.64 (t, 3H, *J* = 8.6 Hz), 6.55–6.52 (m, 2H), 4.71(s, 2H). ^13^C NMR (100 MHz, CDCl_3_) δ 171.8, 168.2, 159,8 (d, *J*_*CF*_ = 245 Hz), 136.6, 136.6, 132.5, 129.8, 129.2, 128.8, 128.0, 127.6, 127.5, 123.5, 123.4, 115.4, 115.1, 102.6, 77.5, 42.0. IR (ATR) (cm^–1^) ν_max_: 3277, 2922, 1762, 1688, 1628. ESI-HRMS [M + H]^+^ calculated, 373,13468, found, 373,13836. HPLC: *t*_*ret*_ 9.714 min, purity (254 nm) 98.7%, (230 nm) 98.7%.

##### 1-Benzyl-3-phenyl-4-(p-tolylamino)-1*H*-pyrrole-2,5-dione (**32**)

Yield: 63%, yellow powder. ^1^H NMR (400 MHz, CDCl_3_) δ 7.37 (d, 2H, *J* = 7.0 Hz), 7.29–7.21 (m, 3H), 7.15 (s, 1H), 7.07–7.02 (m, 3H), 6.94 (dd, 2H, *J*_*1*_ = 8.1 Hz, *J*_*2*_ = 1.5 Hz), 6.74 (d, 2H, *J* = 8.2 Hz), 6.44 (d, 2H, *J* = 8.2 Hz), 4.71 (s, 2H), 2.14 (s, 3H). ^13^C NMR (100 MHz, CDCl_3_) δ: 172.0, 168.4, 136.7, 136.4, 134.6, 133.8, 129.9, 129.5, 128.9, 128.8, 127.9, 127.3, 121.6, 102.1, 41.9, 21.0. IR (ATR) (cm^–1^) ν_max_: 3302, 1758, 1692, 1636. ESI-HRMS [M + H]^+^ calculated, 369,15975, found, 369,16051. HPLC: *t*_*ret*_ 9.963 min, purity (254 nm) 98.9%, (230 nm) 96.3%.

##### 1-Benzyl-3-((4-hydroxyphenyl)amino)-4-phenyl-1*H*-pyrrole-2,5-dione (**33**)

Yield: 85%, orange powder. ^1^H NMR (400 MHz, DMSO) δ 9.49 (s, 1H), 9.19 (s, 1H), 7.37–7.26 (m, 5H), 7.08–7.03 (m, 3H), 6.88 (d, 2H, *J* = 6.6 Hz), 6.56 (d, 2H, *J* = 8.7 Hz), 6.34 (d, 2H, *J* = 8.7 Hz), 4.68 (s, 2H). ^13^C NMR (100 MHz, DMSO) δ: 171.5, 167.5, 154.1, 138.6, 137.2, 129.9, 129.3, 128.8, 128.6, 127.4, 127.3, 126.8, 126.3, 123.7, 114.3, 99.1, 40.7. IR (ATR) (cm^–1^) ν_max_: 3438, 3308, 1753, 1684, 1636. ESI-HRMS [M + H]^+^ calculated, 371,13902, found, 371,14241. HPLC: tret 8.396 min, purity (254 nm) 99.4%, (230 nm) 99.2%.

##### 1-Benzyl-3-((4-(hydroxymethyl)phenyl)amino)-4-phenyl-1*H*-pyrrole-2,5-dione (**34**)

Yield: 76%, yellow powder. ^1^H NMR (400 MHz, DMSO) δ 9.65 (s, 1H), 7.38–7.28 (m, 5H), 7.11–7.05 (m, 3H) 6.95–6.89 (m, 4H), 6.68 (d, 2H, *J* = 8.5 Hz), 5.04 (t, 1H, *J* = 5. Seven Hz), 4.70 (s, 2H), 4.31 (d, 2H, *J* = 5.7 Hz). ^13^C NMR (100 MHz, DMSO) δ: 171.5, 167.6, 137.9, 137.7, 137.1, 135.9, 129.9, 129.2, 128.6, 127.4, 127.0, 126.7, 125.8, 121.3, 101.4, 62.3, 40.8. IR (ATR) (cm^–1^) ν_max_: 3311, 1760, 1694, 1634. ESI-HRMS [M + H]^+^ calculated 385,15467, found, 385,15727. HPLC: *t*_*ret*_ 8.511 min, purity (254 nm) 98.8%, (230 nm) 98.4%.

##### 1-Benzyl-3-((4-(dimethylamino)phenyl)amino)-4-phenyl-1*H*-pyrrole-2,5-dione (**35**)

Yield: 79% red powder. ^1^H NMR (400 MHz, CDCl_3_) δ 7.37 (d, 2H, *J* = 7.2 Hz), 7.28–7.20 (m, 4H), 7.14 (s, 1H), 7.03- 6.98 (m, 3H), 6.90 (d, 2H, *J* = 6.9 Hz), 6.45 (d, 2H, *J* = 8.6 Hz), 6.27 (d, 2H, *J* = 8.6 Hz), 4.69 (s, 2H), (s, 6H). ^13^C NMR (100 MHz, CDCl_3_) δ: 172.2, 168.4, 148.4, 137.1, 136.9, 129.8, 129.6, 128.8, 128.8, 127.8, 127.2, 126.8, 126.0, 123.3, 100.3, 41.8, 40.9. IR (ATR) (cm^–1^) ν_max_: 3300, 2920, 1751, 1689, 1627. ESI-HRMS [M + H]^+^ calculated, 398,1863, found, 398,18523. HPLC: *t*_*ret*_ 8.782 min, purity (254 nm) 97.0%, (230 nm) 96.2%.

##### 4-((1-Benzyl-2,5-dioxo-4-phenyl-2,5-dihydro-1*H*-pyrrol-3-yl)amino)benzamide (**36**)

Yield: 18%. yellow powder. ^1^H NMR (400 MHz, CDCl_3_) δ 8.15 (s, 1H), 7.59 (d, 2H, *J =* 8.6 Hz), 7.39–7.21 (m, 10H), 6.58 (d, 2H, *J =* 8.6 Hz), 4.70 (s, 2H), 4.09 (s, 2H). ^13^C NMR (100 MHz, CDCl_3_) δ: 171.2, 168.6, 163.1, 151.2, 136.2, 131.3, 130.2, 130.2, 129.8, 129.0, 128.9, 128.7, 128.1, 127.7, 42.2. IR (ATR) (cm^–1^) ν_max_: 3473, 3369, 1700, 1669. ESI-HRMS [M + H]^+^ calculated 398,14992, found, 398,14967. HPLC: *t*_*ret*_ 7.685 min, purity (254 nm) 98.4%, (230 nm) 95.8%.

##### 1-Benzyl-3-((2-oxoindolin-5-yl)amino)-4-phenyl-1*H*-pyrrole-2,5-dione (**37**)

Yield: 93%, orange powder. ^1^H NMR (400 MHz, DMSO) δ 10.22 (s, 1H), 9.62 (s, 1H), 7.38–7.30 (m, 5H), 7.09–7.03 (m, 3H), 6.86 (d, 2H, *J =* 8.0 Hz), 6.60 (d, 1H, *J =* 8.3 Hz), 6.56 (s, 1H), 6.41 (d, 1H, *J =* 8.3 Hz), 4.69 (s, 2H), 3.08 (s, 2H). ^13^C NMR (100 MHz, DMSO) δ: 176.0, 171.40, 167.5, 140.0, 138.5, 137.2, 131.2, 130.1, 129.4, 128.6, 127.4, 126.8, 126.5, 125.0, 121.7, 119.5, 108.0, 99.8, 40.7, 35.4. IR (ATR) (cm^–1^) ν_max_: 3283, 2929, 2848, 1757, 1695, 1628. ESI-HRMS [M + H]^+^ calculated, 410,14992, found, 410,15026. HPLC: *t*_*ret*_ 7.421 min, purity (254 nm) 97.9%, (230 nm) 95.3%.

##### 3-(Benzo[d][1,3]dioxol-5-ylamino)-1-benzyl-4-phenyl-1*H*-pyrrole-2,5-dione (**38**)

Yield: 46%, orange powder. ^1^H NMR (400 MHz, DMSO) δ 9.57 (s, 1H), 7.37–7.30 (m, 5H), 7.12–7.07 (m, 3H), 6.91 (dd, 2H, *J*_*1*_*=* 7.7 Hz, *J*_*2*_*=* 1.7 Hz), 6.48 (d, 1H, *J =* 8.3 Hz), 6.38 (d, 1H, *J =* 2.0 Hz), 6.18 (dd, 1H, *J*_*1*_*=* 8.3 Hz, *J*_*2*_*=* 2.1 Hz), 5.85 (s, 2H), 4.69 (s, 2H). ^13^C NMR (100 MHz, DMSO) δ: 171.4, 167.5, 146.6, 143.7, 138.4, 137.1, 131.7, 129.9, 129.1, 128.6, 127.4, 127.3, 126.9, 126.5, 115.7, 106.9, 104.2, 101.0, 100.5, 40.7. IR (ATR) (cm^–1^) ν_max_: 3287, 2931, 1759, 1695, 1627. ESI-HRMS [M + H]^+^ calculated, 399,13393, found, 399,13614. HPLC: *t*_*ret*_ 9.367 min, purity (254 nm) 99.6%, (230 nm) 99.8%.

##### 1-Benzyl-3-((3,5-dimethoxyphenyl)amino)-4-phenyl-1*H*-pyrrole-2,5-dione (**39**)

Yield: 37%, orange powder. ^1^H NMR (400 MHz, CDCl_3_) δ 7.38 (d, 2H, *J =* 7.1 Hz), 7.29–7.22 (m, 3H), 7.07–7.05 (m, 3H), 6.96 (d, 2H, *J =* 5.7 Hz), 6.59 (d, 2H, *J =* 8.5 Hz), 6.39 (d, 2H, *J =* 8.5 Hz), 5.88 (s, 1H), 4.71 (s, 2H), 3.73 (s, 3H), 3.12 (s, 3H). ^13^C NMR (101 MHz, CDCl_3_) δ: 172.0, 168.3, 148.4, 146.3, 136.7, 136.3, 130.1, 129.8, 129.6, 128.9, 128.8, 128.0, 127.4, 113.5, 110.97, 106.7, 101.4, 56.2, 55.3, 42.0. IR (ATR) (cm^–1^) ν_max_: 3300, 1699, 1644. ESI-HRMS [M + H]^+^ calculated 415,16523, found, 415,17132. HPLC: *t*_*ret*_ 8.982 min, purity (254 nm) 99.3%, (230 nm) 98,3%.

##### 1-Benzyl-3-phenyl-4-((4-(piperidin-1-yl)phenyl)amino)-1*H*-pyrrole-2,5-dione (**40**)

Yield: 16%, red powder. ^1^H NMR (400 MHz, CDCl_3_) δ 7.44 (d, 2H, *J* = 6.9 Hz), 7.33 (t, 2H, *J* = 7.2 Hz), 7.29 (d, 1H, *J* = 7.1 Hz), 7.20 (s, 1H), 7.14–7.05 (m, 3H), 6.98 (dd, 2H, *J*_*1*_ = 8.1 Hz, *J*_*2*_ = 1.5 Hz), 6.58–6.51 (m, 4H), 4.77 (s, 2H), 3.03 – 3.00 (m, 4H), 1.67–1.562 (m, 4H), 1.59–1.53 (m, 2H). ^13^C NMR (100 MHz, CDCl_3_) δ: 172.11, 168.4, 149.8, 137.0, 129.8, 129.6, 128.8, 128.0, 127.9, 127.2, 127.0, 123.0, 116.1, 100.9, 77.4, 76.8, 50.9, 41.9, 25.7, 24.3. IR (ATR) (cm^–1^) ν_max_: 3308, 1755, 1692, 1630. ESI-HRMS [M + H]^+^ calculated 438,21760, found, 438,21793. HPLC: *t*_*ret*_ 7.844 min, purity (254 nm) 96,8%, (230 nm) 95.0%.

##### 1-Benzyl-3-((4-morpholinophenyl)amino)-4-phenyl-1*H*-pyrrole-2,5-dione (**41**)

Yield: 84%, yellow powder. ^1^H NMR (400 MHz, CDCl_3_) δ 7.44 (d, 2H, *J* = 7.1 Hz), 7.34 (t, 2H, *J* = 7.3 Hz), 7.29 (d, 1H, *J* = 7.1 Hz), 7.20 (s, 1H), 7.12–7.06 (m, 3H), 6.97 (d, 2H, *J* = 7.0 Hz), 6.58–6.54 (m, 4H), 4.77 (s, 2H), 3.83 – 3.81 (m, 4H), 3.02 – 3.00 (m, 4H). ^13^C NMR (100 MHz, CDCl_3_) δ: 172.0, 168.3, 136.8, 129.8, 129.5, 127.9, 127.1, 123.1, 115.6, 77.4, 66.8, 4.7, 41.9. IR (ATR) (cm^–1^) ν_max_: 3292, 1695, 1628, 1525, 1506. ESI-HRMS [M + H]^+^ calculated, 440,19687, found, 440,19795. HPLC *t*_*ret*_ 9.216 min, (254 nm) 99.2%, (230 nm) 99.47%.

##### 1-Benzyl-3-phenyl-4-((4-thiomorpholinophenyl)amino)-1*H*-pyrrole-2,5-dione (**42**)

Yield: 56%, orange powder. ^1^H NMR (400 MHz, CDCl_3_) δ 7.44 (d, 2H, *J* = 7.1 Hz), 7.35–7.30 (m, 3H), 7.17 (s, 1H), 7.12–7.06 (m, 3H), 6.97 (d, 2H, *J* = 7.0 Hz), 6.59–6.55 (m, 4H), 4.77 (s, 2H), 3.43 – 3.41 (m, 4H), 2.69–2.67 (m, 4H). ^13^C NMR (100 MHz, CDCl_3_) δ: 172.0, 168.3, 137.0, 136.8, 129.8, 129.5, 128.8, 127.9, 127.3, 127.1, 123.3, 116.8, 77.4, 52.3, 41.9, 26.4. IR (ATR) (cm^–1^) ν_max_: 3311, 2914, 2808, 1695, 1640. ESI-HRMS [M + H]^+^ calculated, 456,17403, found, 456,17653. HPLC: tret 9.485 min, purity: (254 nm) 98.0%, (230 nm) 96.6%).

##### 1-Benzyl-3-phenyl-4-((4-(piperazin-1-yl)phenyl)amino)-1*H*-pyrrole-2,5-dione (**43**)

Yield: 80%, green powder. ^1^H NMR (400 MHz, DMSO) δ 9.60 (s, 1H), 9.38 (s, 1H), 7.37–7.28 (m, 5H), 7.11 (t, 1H, *J* = 7.3 Hz), 7.04 (t, 2H, *J* = 7.7 Hz), 6.86 (d, 2H, *J* = 8.5 Hz), 6.64 (d, 2H, *J* = 9.1 Hz), 6.59 (d, 2H, *J* = 9.1 Hz), 4.69 (s, 2H), 3.24, - 3.21 (m, 4H), 3.15 – 3.08 (m, 4H). ^13^C NMR (101 MHz, DMSO) δ: 171.5, 167.5, 146.3, 138.3, 137.2, 130.2, 129.9, 129.2, 128.6, 127.4, 126.8, 126.4, 123.1, 115.5, 99.9, 48.6, 45.9, 42.1, 40.7. IR (ATR) (cm^–1^) ν_max_: 3285, 2961, 1761, 1695, 1641. ESI-HRMS [M + H]^+^ calculated, 439,21285, found, 439,21155. HPLC: *t*_*ret*_ 6.228 min, purity (254 nm) 97.4%, (230 nm) 97.1%.

##### 1-Benzyl-3-((4-(4-methylpiperazin-1-yl)phenyl)amino)-4-phenyl-1*H*-pyrrole-2,5-dione (**44**)

Yield: 53%, red powder. ^1^H NMR (400 MHz, CDCl_3_) δ 7.43 (d, 2H, *J* = 7.0 Hz), 7.33 (t, 2H, *J* = 7.2 Hz), 7.29–7.24 (m, 2H), 7.11–7.04 (m, 3H), 6.96 (d, 2H, *J* = 6.7 Hz), 6.56 – 6.52 (m, 4H), 4.77 (s, 2H), 3.08 – 3.06 (m, 4H), 2.56 – 2.54 (m, 4H), 2.34 (s, 3H). ^13^C NMR (101 MHz, CDCl_3_) δ: 172.0, 168.3, 148.8, 136.9, 136.8, 129.8, 129.5, 128.8, 128.6, 127.8, 127.2, 127.0, 123.1, 101.1, 55.0, 49.4, 46.1, 41.9. IR (ATR) (cm^–1^) ν_max_: 3296, 2935, 2841, 2904, 1756, 1960, 1627. ESI-HRMS [M + H]^+^ calculated 453,2285, found, 453,23008. HPLC: *t*_*ret*_ 6.206 min, purity (254 nm) 97.5%, (230 nm) 95.3%.

##### *tert*-Butyl-(1-(4-((1-benzyl-2,5-dioxo-4-phenyl-2,5-dihydro-1*H*-pyrrol-3-yl)amino)phenyl)piperidin-4-yl)carbamate (**45**)

Yield: 98% orange powder. ^1^H NMR (400 MHz, DMSO) δ 9.53 (s, 1H), 7.37–7.27 (m, 5H), 7.11–7.02 (m, 3H), 6.86 – 6.83 (m, 3H), 6.57 (d, 2H, *J* = 9.0 Hz), 6.49 (d, 2H, *J* = 9.0 Hz), 4.69 (s, 2H), 3.42 (d, 2H, *J* = 11.2 Hz), 3.33 (m, 1H), 2.57 (t, 2H, *J* = 11.2 Hz), 1.72 (d, 2H, *J* = 10.6 Hz), 1.38 (m, 11H). ^13^C NMR (100 MHz, DMSO) δ: 171.5, 167.5, 154.8, 147.7, 138.3, 137.2, 129.9, 129.2, 128.6, 127.4, 126.8, 126.2, 123.1, 114.9, 99.2, 77.5, 48.0, 47.4, 40.7, 31.0, 28.3. IR (ATR) (cm^–1^) ν_max_: 3371, 3292, 1694, 1678, 1636. ESI-HRMS [M + H]^+^ calculated, 539,26528, found, 539,26531. HPLC: *t*_*ret*_ 9.949 min, purity (254 nm) 98.6%, (230 nm) 97.41%.

##### 3-((4-(4-Aminopiperidin-1-yl)phenyl)amino)-1-benzyl-4-phenyl-1*H*-pyrrole-2,5-dione (**46**)

Yield: 73%, yellow powder. ^1^H NMR (400 MHz, DMSO) δ 9.82 (s, 1H), 8.62 (bs, 2H), 7.37–7.28 (m, 7H), 7.14–7.06 (m, 3H), 6.89 (d, 2H, *J* = 7.9 Hz), 6.78 (d, 2H, *J* = 7.9 Hz), 4.7 (s, 2H), 3.66–3.26 (m, 4H), 2.22–1.93 (m, 4H). ^13^C NMR (100 MHz, DMSO) δ: 171.8, 168.0, 138.3, 137.5, 130.1, 129.6, 127.9, 127.9, 127.5, 127.4, 123.3, 55.4, 49.0, 41.3, 27.9. IR (ATR) (cm^–1^) ν_max_: 3054, 2901, 1705, 1636, 1609. ESI-HRMS [M + H]^+^ calculated 453,2285, found, 453,23044. HPLC: *t_ret_* 6.470 min, purity (254 nm) 100%, (230 nm) 99.3%.

##### 1-Benzyl-3-phenyl-4-((4-(piperidin-4-ylamino)phenyl)amino)-1H-pyrrole-2,5-dione (**47**)

Yield: 82%, yellow powder. ^1^H NMR (400 MHz, DMSO) δ 9.73 (s, 1H), 9.25 (s, 1H), 8.95 (s, 1H), 7.37–7.28 (m, 5H), 7.09–7.05 (m, 3H), 6.91 (d, 2H, *J* = 6.8 Hz), 6.85 (s, 1H), 6.79–6.73 (m, 2H), 3.53 – 3.41 (m, 1H), 3.31 (d, 2H, *J* = 11.0 Hz), 2.94 – 2.82 (m, 2H), 1.93 (d, 2H, *J* = 12.0 Hz), 1.81 – 1.68 (m, 2H). ^13^C NMR (100 MHz, DMSO) δ: 171.3, 167.5, 138.0, 137.0, 129.8, 129.3, 128.5, 127.4, 127.0, 126.6, 123.0, 41.5, 40.7, 38.9. IR (ATR) (cm^–1^) ν_max_: 3286, 1757, 1695, 1641. ESI-HRMS [M + H]^+^ calculated 453,2285, found, 453,22823. HPLC: *t*_*ret*_ 6.078 min, purity (254 nm) 96.9%, (230 nm) 99.4%.

##### 1-Benzyl-3-((4-((1-methylpiperidin-4-yl)amino)phenyl)amino)-4-phenyl-1*H*-pyrrole-2,5-dione (**48**)

Yield: 66%, orange powder. ^1^H NMR (400 MHz, DMSO) δ 9.42 (s, 1H), 7.37–7.27 (m, 5H), 7.02 (m, H, *J* = 5.2 Hz), 6.96 – 6.83 (m, 2H), 6.48 (d, 2H, *J* = 8.7 Hz), 6.18 (d, 2H, *J* = 6.19 Hz), 5.20 (s, 1H, *J* = 7.6 Hz), 4.67 (s, 2H), 3.01–2.99 (m, 1H), 2.66 (d, 2H, *J* = 11.6 Hz), 2.13 (s, 3H), 1.92 (t, 2H, *J* = 10.6 Hz), 1.76 (d, 2H, *J* = 12.2 Hz), 1.30–1.23 (m, 2H). ^13^C NMR (100 MHz, CDCl_3_) δ: 172.1, 168.3, 144.8, 137.3, 137.0, 129.9, 129.6, 128.8, 127.9, 127.2, 126.8, 126.5, 123.8, 112.9, 100.3, 77.4, 54.7, 46.4, 41.8, 32.5. IR (ATR) (cm^–1^) ν_max_: 3294, 1753, 1695, 1619. ESI-HRMS [M + H]^+^ calculated, 467,24415, found, 467,2436. HPLC: *t*_*ret*_ 6.052 min, purity (254 nm) 98.0%, (230 nm) 97.5%.

##### 1-Benzyl-3-((4-(4-oxopiperidin-1-yl)phenyl)amino)-4-phenyl-1*H*-pyrrole-2,5-dione (**49**)

Yield: 49%, orange powder. ^1^H NMR (400 MHz, CDCl_3_) δ 7.45 (d, 2H, *J* = 6.8 Hz), 7.36 – 7.28 (m, 3H), 7.16 (s, 1H), 7.1 – 7.04 (m, 3H), 6.99 (d, 2H, *J* = 9.6 Hz), 6.64 – 6.58 (m, 4H), 4,77 (s, 2H) 3.48 (t, 4H, *J* = 6.1 Hz), 2.49 (t, 2H, 4H). ^13^C NMR (101 MHz, CDCl_3_) δ: 171.9, 168.3, 137.0, 136.8, 130.2, 129.9, 129.2, 128.8, 128.8, 127.9, 127.3, 127.1, 123.5, 115.8, 77.4, 49.4, 41.9, 40.4. IR (ATR) (cm^–1^) ν_max_: 3356, 1757, 1695, 1634. ESI-HRMS [M + H]^+^ calculated 452,19687, found, 452,19682. HPLC *t*_*ret*_ 9.489 min, purity: (254 nm) 96.2%, (230 nm) 100%.

##### 1-Benzyl-3-phenyl-4-((4-(piperidine-1-carbonyl)phenyl)amino)-1*H*-pyrrole-2,5-dione (**50**)

Yield: 50%, orange powder. ^1^H NMR (400 MHz, CDCl_3_) δ 7.40 (d, 2H, *J* = 7.1 Hz), 7.34–7.26 (m, 4H), 7.08–7.01 (m, 7H), 6.59 (d, 2H, *J* = 8.2 Hz), 4.75 (s, 2H), 3.70–3.50 (m, 2H), 3.27–3.07 (m, 2H), 1.63–1.44 (m, 6H). ^13^C NMR (101 MHz, CDCl_3_) δ: 171.7, 169.7, 168.2, 137.4, 136.6, 136.0, 132.31, 130.0, 129.4, 128.8, 128.8, 128.0, 127.6, 127.4, 127.3, 121.3, 103.8, 77.4, 49.0, 43.5, 42.0, 26.5, 24.7. IR (ATR) (cm^–1^) ν_max_: 3275, 2928, 2852, 1763, 1694, 1617. ESI-HRMS [M + H]^+^ calculated, 466,21252, found, 466,21351. HPLC: *t*_*ret*_ 8.630 min, purity (254 nm) 100%, (230 nm) 100%.

##### 1-(*tert*-Butyl)-3-phenyl-4-((4-(piperidin-1-yl)phenyl)amino)-1*H*-pyrrole-2,5-dione (**51**)

Yield: 75%, yellow powder. ^1^H NMR (400 MHz, CDCl_3_) δ 7.12–7.05 (m, 4H), 6.98–6.96 (m, 2H), 6.54 (d, 2H, *J* = 9.1 Hz), 6.52 (d, 2H, *J* = 9.1 Hz), 3.02–2.99 (m, 4H), 1.67–1.62 (m, 12H), 1.57–1.51 (m, 3H). ^13^C NMR (100 MHz, CDCl_3_) δ: 173.9, 169.3, 149.6, 136.5, 128.0, 128.5, 127.2, 122.9, 116.2, 100.6, 77.4, 57.5, 51.1, 29.3, 25.7, 24.3. IR (ATR) (cm^–1^) ν_max_: 3296, 2933, 2784, 1749, 1695, 1646. ESI-HRMS [M + H]^+^ calculated, 404,23325, found, 404,23388. HPLC: *t*_*ret*_ 7.801 min, purity (254 nm) 98.9%, (230 nm) 98.8%.

##### 3-Phenyl-4-((4-(piperidin-1-yl)phenyl)amino)-1*H*-pyrrole-2,5-dione (**52**)

Yield: 79%, red powder. ^1^H NMR (400 MHz, DMSO) δ 10.61 (s, 1H), 9.26 (s, 1H), 7.09–6.99 (m, 3H), 6.85 (d, 2H, *J* = 6.9 Hz), 6.55 (d, 2H, *J* = 9.0 Hz), 6.48 (d, 2H, *J* = 9.0 Hz), 2.96–2.93 (m, 4H), 1.54–1.48 (m, 6H). ^13^C NMR (100 MHz, DMSO) δ: 172.9, 169.0, 130.1, 128.6, 126.0, 100.2, 49.9, 39.7, 24.9, 23.9. IR (ATR) (cm^–1^) ν_max_: 3285, 2935, 1755, 1688, 1624, 1608. ESI-HRMS [M + H]^+^ calculated, 3481,17065, found, 348,17138. HPLC: *t*_*ret*_ 3.116 min, purity (254 nm) 100%, (230 nm) 99.0%.

### 4.2 HR-MS Measurements

The LC–MS measurements were carried out using a 1290 Infinity I LC-System (Agilent Technologies, Waldbronn, Germany) consisting of a binary pump (G4220A) and a thermostated column compartment (G1316A) coupled to a SCIEX Triple-TOF 5600+ (Sciex, Darmstadt, Germany) mass spectrometer equipped with a DuoSpray ESI ion source (Sciex) using the SWATH-MS acquisition mode. Sample injections were performed with an HTC-xt PAL (CTC Analytics, Zwingen, Switzerland) autosampler. To prevent salts and buffers from entering the mass spectrometer, a Cheminert 6-port valve (Valco Instruments Company Inc., TX, USA) was connected between the column compartment and the ion source. Data acquisition was performed with Analyst 1.7.1 software and data analysis using PeakView 2.2 (both Sciex). Prior to LC–MS analysis, stock solutions (100 μM in DMSO) were prepared. Liquid chromatography was performed using a CORTECS C18+ (2.1 × 50 mm, 2.7 μm; Waters, MA, USA) separation column and a linear gradient (*t*[min] – % B) 0–10, 0.5–10, 5.5–95, 6–95, 6.01–10, 6.5–10 at a flow rate of 0.5 mL/min. Mobile phase A consisted of Water +0.1% FA and mobile phase B of ACN + 0.1% FA. The temperature of the column compartment was set to 60 °C and the injection volume was 5 μL.^23^ Compounds **3**, **29**, **40**, and **51** were analyzed at the Mass Spectrometry Department, Institute of Organic Chemistry, Eberhard-Karls-University Tübingen.

### 4.3 Time-Resolved LXR FRET Assay

The biochemical TR-FRET assays were performed using the LanthaScreen TR-FRET LXRα Coactivator Assay Kit, goat (ThermoFisher Scientific, PV4655), and the LanthaScreen TR-FRET LXRβ assay kit, goat (ThermoFisher Scientific, PV4658), according to the manufacturer’s instructions using the following concentrations: 5 nM LXRα LBD (GST); 10 nM LXRβ LBD (GST); 500 nM fluorescein-TRAP220/DRIP-2; 200 nM fluorescein-D22; 10 nM Tb anti-GST antibody. The TR-FRET signals were measured after 6 h using a TECAN SPARK multimode microplate reader with the following settings: first excitation wavelength: 360 nm (bandwidth 35 nm), first emission wavelength: 485 nm (bandwidth 20 nm); second excitation wavelength: 360 nm (bandwidth 35 nm), second emission wavelength: 535 nm (bandwidth 25 nm); integration time: 200 μs; lag time: 100 μs; settle time: 0 ms.

### 4.4 LXR Reporter Assay

LXR reporter assays were performed in Hep3B cells that were obtained from the American Type Culture Collection. The cells were cultured with DMEM (Gibco) medium, supplemented with 10% FCS, at 37 °C and 7% CO_2_. Mycoplasma contamination was excluded via a PCR-based method. For the generation of human *NR1H3* and *NR1H2* knockout lines, CRISPRdirect was used to design gRNAs against both human genes, and complementary oligonucleotides were ordered from Sigma-Aldrich. The gRNAs (Table 5) were cloned into *pX.458* using the restriction side BbSI. CRISPRCas9-mediated knockout was performed by transient transfection using Lipofectamine 3000 reagent from Thermo Fischer Scientific. After generating single-cell clones, the knockouts were confirmed by Western blot analysis (see 4.8). The knockout reporter cell lines were generated via lentiviral infection using the *pL-LXRE-GFP* (*pGF-LXRE-GFP*) plasmid obtained from System Bioscience, and cells were selected using 3 μg/mL Puromycin. The reporter assays were performed after 2 days of treatment with a dilution series of LXR agonists by measuring GFP using the LSRFortessa cell analyzer (BD Bioscience, Flow Cytometry Core Facility Tuebingen). Data were collected using DIVA software (v9.0.1).

**Table 5:** gRNAs Used for Knockout of Human *NR1H3* and *NR1H2*

gene	position	20mer + PAM
human *NR1H3*	E4 (134 – 156)	CCGCCGCAGCGTCATCAAGGGAG
human *NR1H2*	E5 (212 – 234)	CCAGATGGACGCTTTCATGCGGC

### 4.5 Nuclear Receptor Panel Screening

For nuclear receptor panel screening, the HepG2 cell line (obtained by American Type Culture Collection) was maintained in DMEM (Gibco) supplemented with 10% FBS+ 1% nonessential amino acids medium and incubated at 37 °C. Plasmids for mouse constitutive androstane receptor (mCAR) and its luciferase gene reporter construct p2B6-luc have been described in Mejdrová et al.^24^ Other luciferase reporter constructs have been described in Stefela et al.^25^ The transient transfection experiments were performed on 48-well format plates 24 h after cell seeding (1.1 × 10^5^ per well). The Lipofectamine 3000 transfection reagent was used according to the manufacturer’s instructions. Each well was transfected by 150 ng of the reporter construct, 100 ng of the receptor expression construct, and 30 ng of *Renilla reniformis* luciferase construct (pRL-TK) to normalize the transfection. The treatment was carried out 24 h later. The cells were treated with tested substances at 10 μM concentration or 0.1% DMSO as control normalized to this volume of solvent in Opti-MEM (Gibco) containing 5% FBS for 24 h. The dual-luciferase reporter assay system (Promega) was used for the cell lysis and luciferase measurements.

### 4.6 Organoids

Isolation of murine HCC organoid cultures was performed from liver tumors derived from C57BL/6 mice using established protocols.^26^ The animal experiments were approved by committees of the regional authority of the state of Baden-Wuerttemberg (Regierungspraesidium Tuebingen, authorization number M15/18G) as appropriate. After digestion, the organoids were embedded in 100% Growth Factor Reduced (GFR) MatrigelTM (Corning) and cultured at 37 °C and 5% CO_2_ in HCC organoid medium supplemented with nicotinamide (10 mM), *N*-acetylcystein (1.25 mM), human HGF (50 ng/mL), human FGF-10 (100 ng/mL), human R-Spondin (0.5 μg/mL), forskolin (10 μM), A83- 01 (0.5 μM), murine Noggin (50 ng/mL), gastrin I (10 nM), human BMP-7 (25 ng/mL), human Wnt3a (100 ng/mL), B27 supplement (1×) and N2 supplement (1×). The cultures were split by mechanical dissociation every 3 to 4 days.

### 4.7 Treatment Studies and Measurements of Oxidative Stress

For organoid treatment studies, digestion of organoids was performed using TrypLE Express (Gibco) supplemented with 100 μg/mL DNaseI and 10.5 μM Y-27632 dihydrochloride (Invitrogen) at 37 °C, and afterward, single-cells were plated in white 96 well plates (Sarstedt) in a mixture of 10% Matrigel (Growth Factor Reduced (GFR) Matrigel, Corning) in culture medium. Cell viability was determined after 5 days of treatment using the CellTiter-Glo 3D Cell Viability Assay Kit (Promega). Cell viability of Hep3B, AML12, and BNL CL.2 cells was determined after 5 days of treatment in white 96 well plates (Sarstedt) using the CellTiter-Glo 2.0 Cell Viability Assay Kit (Promega). AML12 and BNL CL.2 cells were also obtained from the American Type Culture Collection. The luminescence signal was measured using an INFINITE M PLEX plate reader (Tecan) and the Tecan i-control software (v3.9.1). Sodium oleate (Selleckchem) was dissolved in methanol and added to cells at a final concentration of 200 μM.

The determination of oxidative stress levels was performed using CellROX Green Reagent from Thermo Fischer Scientific in Hep3B cells. Measurement of green fluorescence was performed using the FACS LSRFortessa cell analyzer (BD Bioscience, Flow Cytometry Core Facility Tuebingen) after 3 days of treatment. Data were collected using DIVA software (v9.0.1).

### 4.8 Protein Expression Analyses

Proteins were extracted from cells using RIPA buffer, separated by SDS-PAGE, and transferred to PVDF membranes (Amersham Hybond ECL). For protein detection, membranes were incubated with antibodies against LXRα (Novus Biologicals, NBP2–17186, dilution: 1:1000), LXRβ (Cell Signaling Technology, 13519S, clone: D6M9D, dilution: 1:1000), CHOP (Cell Signaling, 2895 (L63F7), dilution: 1:1000), GADD34 (Proteintech, 10449–1-AP, dilution: 1:1000), SOD1 (ThermoFisher Scientific, MA1–105, clone: 8B10, dilution: 1:1000), α-tubulin (Cell Signaling, 2125 clone: 11H10, dilution: 1:5000), and vinculin (Sigma-Aldrich V9131, clone: hVIN-1, dilution: 1:5000) and visualized using the ChemiDoc MP imaging system (Bio-Rad, ImageLab v5.2.1).

### 4.9 Molecular Modeling and Proposed Binding Mode

The proposed binding mode of our compounds within the ligand binding pocket from LXR was generated to rationalize their interactions and support the SAR discussion. We generated LBD models using LXRα crystal (PDB ID: 2ACL) and LXRβ (PDB ID: 1PQC) structures as templates, due to their high resolution and similarity between cocrystallized ligands and our analog series. Structures were prepared using a Protein preparation wizard and had their H1–H2 gaps filled using Prime. His218 (LXRα) and His219 (LXRβ) were set to HIE configuration, with the NH pointing toward the carbonyl groups of the ligands. To obtain the starting configuration for the systems without crystals, Glide docking was conducted (Glide v. 7.7).^27,28^ For docking, we used default settings and defined residues with 10 Å around CITCO in the crystallographic structure for the binding site. Docking was conducted by using standard precision (SP). Redocking of GSK3987 was conducted as the reference pose and reproduced the standard binding mode. The docking in LXR-LBDs resulted in poses mainly accommodated in hydrophobic regions, and the representative pose was selected based on the Glide docking score and energy.

### 4.10 Molecular Dynamics Simulations

We simulated the monomeric LXR without the coactivator peptide with a similar protocol as described.^24^ We used the Desmond MD simulation engine^29^ and the OPLS4 force field.^30^ The prepared systems were solvated in a cubic box with the size of the box set as a 13 Å minimum distance from the box edges to any atom of the protein with periodic bound conditions. TIP3P water model^31^ was used to describe the solvent and the net charge was neutralized using Na^+^ ion with a final salt concentration of 150 mM. RESPA integrator timesteps of 2 fs for bonded and near and 6 fs for far were applied. The short-range Coulombic interactions were treated using a cutoff value of 9.0 Å, whereas long-range Coulombic interactions were estimated using the Smooth Particle Mesh Ewald method.^32^ Before the production simulations, the systems were relaxed by using the default Desmond relaxation protocol. Simulations were run in an NPT ensemble, with a temperature of 310 K (using the Nosé–Hoover thermostat^33,34^) and a pressure of 1.01325 bar (Martyna–Tobias–Klein barostat^35^). For each LXR-ligand combination, five independent simulations of 500 ns were carried out, resulting in at least 2.5 μs of simulation data for each system. Maestro simulation interaction analysis tool (Schrödinger, LLC) was used for the analysis of RMSD and interaction analysis. All molecular dynamics trajectories and raw data related to the protein–ligand interactions within the simulations are available in the repository: 10.5281/zenodo.12925395

### 4.11 MM/GBSA Binding Energy Calculations

To gain deeper insight into the interactions between LXR isoforms-ligands, we explored their predicted binding energies, employing the molecular mechanics-generalized Born surface area (MM/GBSA) approach as outlined.^36^ MM/GBSA predicts the binding free energy of protein–ligand complexes.^36,37^ The ligands’ ranking based on the free energy could be correlated to the experimental binding affinities, especially in a congeneric series. Every 50^th^ frame from the simulations was considered for the calculations. These were used as input files for the MM/GBSA calculations with the thermal_mmgbsa.py script from the Schrödinger package. Calculated free-binding energies (kcal/mol) are represented by MM/GBSA and normalized by the number of heavy atoms (HAC), according to the following formula: ligand efficiency = ln(binding energy)/(1 + ln(HAC)).

### 4.12 Statistical Analysis

All the statistical analyses were carried out by using GraphPad Prism v9.41 or v10.1.2 software. Statistical analyses were performed by using the two-tailed Student′s *t* test or one-way ANOVA with Dunnett’s post hoc test. A *P* value of <0.05 was considered statistically significant. Drug response curves and EC_50_ values were calculated by using GraphPad Prism v9.41 software. The results are presented as mean ± standard deviation from independent biological measurements.

## Supporting Information Available

Further synthetic methodology of key intermediates, HPLC data, HR-MS values, all NMR spectra, nuclear receptor panel, and sorafenib cell effect; generated homology models, molecular dynamics trajectories, and raw data related to the protein–ligand interactions and predicted binding energy within the simulations; relevant PDBs representing clustered structures from the MD trajectories; initial modeled structures also in the Zenodo link; atomic coordinates (PDF)
Molecular formula strings (CSV)

## Abbreviations

mCAR: androstane receptor
AhR: arylhydrocarbon receptor
DNA: deoxyribonucleic acid
DCM: dichloromethane
DMF: dimethylformamide
DBD: DNA binding domain
EtOAc or EA: ethyl acetate
ER: endoplasmic reticulum
ER: estrogen receptor
FBS: fasting blood sugar
FXR: farsenoid receptor
FCS: fetal calf serum
FACS: fluorescent-activated cell sorting
GFP: green fluorescent protein
EC_50_: half-maximun effective concentration
HCC: hepatocellular carcinoma
iCCA: intrahepatic cholangiocarcinoma
LBD: ligand binding domain
LBP: ligand binding pocket
LXR: liver X receptor
LXRE: liver X receptor response element
MeOH: methanol
MD: molecular dynamics
NASH: nonalcoholic steatohepatitis
NR: nuclear receptor
PE: petrolether
PPAR: peroxisome proliferator-activated receptor
tBuOK: potassium tert-butoxide
Raf: rapidly accelerated fibrosarcoma
RXR: retinoid-X receptor
SD: standard deviation
SCD1: stearoyl-CoA desaturase
THF: tetrahydrofuran
TR-FRET: time-resolved fluorescence resonance energy transfer
TLC: thin-layer chromatography
Et_3_N: triethylamine
VDR: vitamin D receptor
