# Supplementary material for: Uncovering α-Selectivity for Liver X Receptor Agonists for Lipotoxic Cancer Therapies
Source: J Med Chem. 2025 Mar 24;68(7):7180–96. doi: 10.1021/acs.jmedchem.4c02712 (PMC11997999; doi:10.1021/acs.jmedchem.4c02712)
Supplement: Supplementary file 1 — jm4c02712_si_001.pdf [file jm4c02712_si_001.pdf]

Supporting Information

Uncovering α-selectivity for LXR agonists for lipotoxic cancer therapies

Júlia Galvez B. Pedreira[1,2#], Pascal Woelffing[2,3#], Moritz Schwarz[1], Simon Ebner[1], Ramona Rudalska[2,3], Benedikt Masberg[4], Aylin Esposito[2,3], Azam Rashidian[1], Ekaterina Schevchenko[1], Lucie Smutna[5], Petr Pavek[5], Jenni Kublbeck[6,7], Thales Kronenberger[1,6,7,8], Lars Zender[2,3,9,10], Michael Lämmerhofer[4], Daniel Dauch[2,3,9], Stefan Laufer[1,2,8,9*]*

[1]Department of Pharmaceutical and Medicinal Chemistry, Institute of Pharmaceutical Sciences, University of Tuebingen, Auf der Morgenstelle 8, 72076 Tuebingen, Germany

[2]IFIT Cluster of Excellence EXC 2180 'Image-Guided and Functionally Instructed Tumor Therapies', University of Tuebingen, 72076 Tuebingen, Germany

[3]Department of Medical Oncology and Pneumology, University Hospital Tuebingen, Otfried-Mueller-Strasse 14, 72076 Tuebingen, Germany

[4]Pharmaceutical (Bio-)Analysis, Institute of Pharmaceutical Sciences, Eberhard-Karls University of Tuebingen, Auf der Morgenstelle 8, Tuebingen 72076, Germany

[5]Department of Pharmacology and Toxicology, Faculty of Pharmacy in Hradec Kralove, Charles University, Akademika Heyrovskeho 1203, 500 05 Hradec Kralove, Czech Republic

[6]School of Pharmacy, Faculty of Health Sciences, University of Eastern Finland, P.O. Box 1627, FI-70210 Kuopio, Finland

[7]A.I. Virtanen Institute for Molecular Sciences, University of Eastern Finland, P.O. Box 1627, FI-70210 Kuopio, Finland

[8]Partner-site Tuebingen, German Center for Infection Research (DZIF), Elfriede-Aulhorn-Str. 6, 72076, Tuebingen, Germany.

[9]Tuebingen Center for Academic Drug Discovery & Development (TüCAD$_2$), Auf der Morgenstelle 8, 72076 Tuebingen, Germany

[10]German Cancer Research Consortium (DKTK), Partner Site Tuebingen, German Cancer Research Center (DKFZ), Im Neuenheimer Feld 280, 69120 Heidelberg, Germany

Corresponding Authors:
Stefan Laufer - Telephone +49 7071 29 72459, stefan.laufer@uni-tuebingen.de
Daniel Dauch - Telephone +49 7071 29 83180, daniel.dauch@med.uni-tuebingen.de

Table of Contents

1 List of compounds 2
2 Statistical analysis of FRET-based biochemical LXR activation assays 4
3 Nuclear Receptor panel evaluation 5
4 Effect of sorafenib monotherapy on cell viability 5
5 Further synthetic description 6
5.1 Preparation of amides 6
5.2 Preparation of maleimide 6
5.3 Preparation of Chloro-maleimide 6
5.4 Preparation of compounds 16 – 21 7
5.5 Preparation anilines 8
5.5.1 Aromatic substitution 8
5.5.2 Reduction of nitro group 9
5.6 Preparation of compound 21 9
5.7 Preparation of compound 26 9
5.8 Preparation. of compound 27 10
5.9 Preparation of compound 46 10
5.10 Preparation of compound 44 10
6 NMR spectra of final compounds 11
7 HR-MS measurements 52
8 HPLC traces of compounds 3, 16 – 53 53

1 List of compounds

	**InChl**	**FRET activity**	
		α	β
**3**	O=C1N(CC2=CC=CC=C2)C(C(NC3=CC=C(OC)C=C3)=C1C4=CC=CC=C4)=O	154	180
**16**	O=C1N(CC2=CC=C(F)C=C2)C(C(NC3=CC=C(OC)C=C3)=C1C4=CC=CC=C4)=O	768	594
**17**	O=C1N(CC2=CC=C(C)C=C2)C(C(NC3=CC=C(OC)C=C3)=C1C4=CC=CC=C4)=O	579	332
**18**	O=C1N(CC2=CC=C(C(F)(F)F)C=C2)C(C(NC3=CC=C(OC)C=C3)=C1C4=CC=CC=C4)=O	> 3,000	> 3,000

**19**	O=C1N(CC2=CC=C(OC)C=C2)C(C(NC3=CC=C(OC)C=C3)=C1C4=CC=CC=C4)=O	518	592
**20**	O=C1N(CC2=CC=C(C(C)(C)C)C=C2)C(C(NC3=CC=C(OC)C=C3)=C1C4=CC=CC=C4)=O	> 3,000	> 3,000
**21**	O=C1N(CC2=CC=NC=C2)C(C(NC3=CC=C(OC)C=C3)=C1C4=CC=CC=C4)=O	390	231
**22**	O=C1N(CC2CCCCC2)C(C(NC3=CC=C(OC)C=C3)=C1C4=CC=CC=C4)=O	1963	2590
**23**	O=C1N(CC2CC2)C(C(NC3=CC=C(OC)C=C3)=C1C4=CC=CC=C4)=O	342	163
**24**	O=C1N(C(C)(C)C)C(C(NC2=CC=C(OC)C=C2)=C1C3=CC=CC=C3)=O	245	232
**25**	O=C1NC(C(NC2=CC=C(OC)C=C2)=C1C3=CC=CC=C3)=O	> 3,000	> 3,000
**26**	O=C1N(CC2=CC=CC=C2)C(C(OC3=CC=C(OC)C=C3)=C1C4=CC=CC=C4)=O	> 3,000	> 3,000
**27**	O=C1N(CC2=CC=CC=C2)C(C(C3=CC=C(OC)C=C3)=C1C4=CC=CC=C4)=O	> 3,000	> 3,000
**28**	O=C1N(CC2=CC=CC=C2)C(C(NCC3=CC=C(OC)C=C3)=C1C4=CC=CC=C4)=O	> 3,000	> 3,000
**29**	O=C1N(CC2=CC=CC=C2)C(C(NC3=CC=CC=C3)=C1C4=CC=CC=C4)=O	312	265
**30**	O=C1N(CC2=CC=CC=C2)C(C(NC3=CC=C(Cl)C=C3)=C1C4=CC=CC=C4)=O	1154	1181
**31**	O=C1N(CC2=CC=CC=C2)C(C(NC3=CC=C(F)C=C3)=C1C4=CC=CC=C4)=O	335	548
**32**	O=C1N(CC2=CC=CC=C2)C(C(NC3=CC=C(C)C=C3)=C1C4=CC=CC=C4)=O	825	617
**33**	O=C1N(CC2=CC=CC=C2)C(C(NC3=CC=C(O)C=C3)=C1C4=CC=CC=C4)=O	390	206
**34**	O=C1N(CC2=CC=CC=C2)C(C(NC3=CC=C(CO)C=C3)=C1C4=CC=CC=C4)=O	278	133
**35**	O=C1N(CC2=CC=CC=C2)C(C(NC3=CC=C(N(C)C)C=C3)=C1C4=CC=CC=C4)=O	71	91
**36**	O=C1N(CC2=CC=CC=C2)C(C(NC3=CC=C(C(N)=O)C=C3)=C1C4=CC=CC=C4)=O	> 3,000	> 3,000
**37**	O=C1N(CC2=CC=CC=C2)C(C(NC3=CC=C(C(N)=O)C=C3)=C1C4=CC=CC=C4)=O	139	84
**38**	O=C1N(CC2=CC=CC=C2)C(C(NC3=CC(OCO4)=C4C=C3)=C1C5=CC=CC=C5)=O	42	45
**39**	O=C1N(CC2=CC=CC=C2)C(C(NC3=CC(OC)=CC(OC)=C3)=C1C4=CC=CC=C4)=O	737	1318
**40**	O=C1N(CC2=CC=CC=C2)C(C(NC3=CC=C(N4CCCCC4)C=C3)=C1C5=CC=CC=C5)=O	42	266

**41**	O=C1N(CC2=CC=CC=C2)C(C(NC3=CC=C(N4CCOCC4)C=C3)=C1C5=CC=CC=C5)=O	35	106
**42**	O=C1N(CC2=CC=CC=C2)C(C(NC3=CC=C(N4CCSCC4)C=C3)=C1C5=CC=CC=C5)=O	50	123
**43**	O=C1N(CC2=CC=CC=C2)C(C(NC3=CC=C(N4CCNCC4)C=C3)=C1C5=CC=CC=C5)=O	1253	283
**44**	O=C1N(CC2=CC=CC=C2)C(C(NC3=CC=C(N4CCN(C)CC4)C=C3)=C1C5=CC=CC=C5)=O	379	657
**45**	O=C1N(CC2=CC=CC=C2)C(C(NC3=CC=C(N4CCC(NC(OC(C)(C)C)=O)CC4)C=C3)=C1C5=CC=C C=C5)=O	> 3,000	> 3,000
**46**	O=C1N(CC2=CC=CC=C2)C(C(NC3=CC=C(N4CCC(N)CC4)C=C3)=C1C5=CC=CC=C5)=O	630	120
**47**	O=C1N(CC2=CC=CC=C2)C(C(NC3=CC=C(NC4CCNCC4)C=C3)=C1C5=CC=CC=C5)=O	618	116
**48**	O=C1N(CC2=CC=CC=C2)C(C(NC3=CC=C(NC4CCN(C)CC4)C=C3)=C1C5=CC=CC=C5)=O	786	150
**49**	O=C1N(CC2=CC=CC=C2)C(C(NC3=CC=C(N4CCC(CC4)=O)C=C3)=C1C5=CC=CC=C5)=O	103	130
**50**	O=C1N(CC2=CC=CC=C2)C(C(NC3=CC=C(C(N4CCCCC4)=O)C=C3)=C1C5=CC=CC=C5)=O	74	225
**51**	O=C1N(C(C)(C)C)C(C(NC2=CC=C(N3CCCCC3)C=C2)=C1C4=CC=CC=C4)=O	36	156
**52**	O=C1NC(C(NC2=CC=C(N3CCCCC3)C=C2)=C1C4=CC=CC=C4)=O	> 3,000	> 3,000

2 Statistical analysis of FRET-based biochemical LXR activation assays

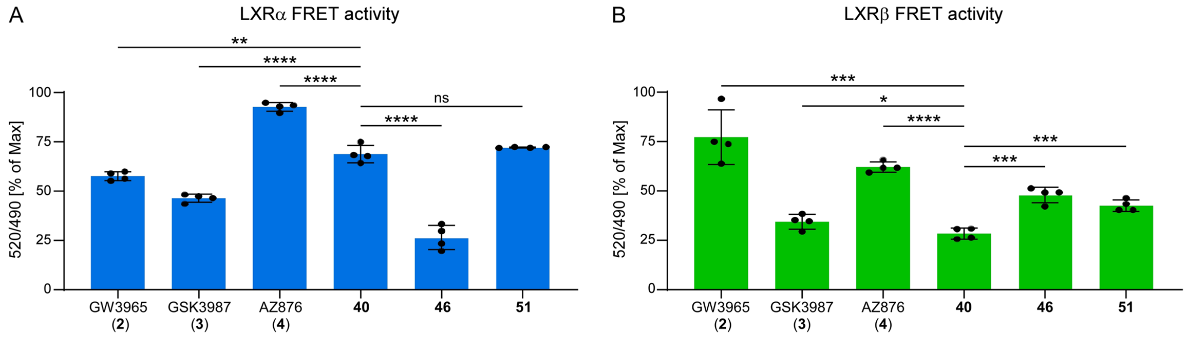

Supplementary Figure 1. Statistical analysis of FRET-based biochemical LXR activation assays (A,B) Comparison of FRET activity of GW3965 (2), GSK3987 (3), AZ876 (4) and novel compounds 40, 46 and 51 at a concentration of 123 nM for LXRα (A) and LXRβ (B) (n = 4 replicates, data are presented as mean values +/- SD, statistical significance was calculated using two-tailed Student´s t-test, **** = $P < 0.0001$, *** = $P < 0.001$, ** = $P < 0.01$, * = $P < 0.05$).

3 Nuclear Receptor panel evaluation

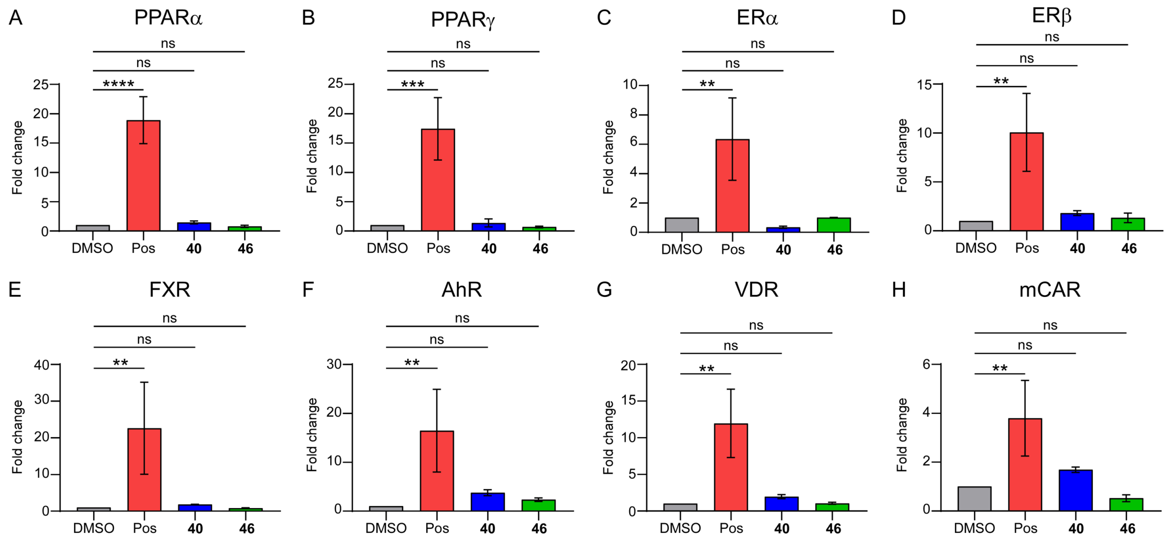

Supplementary Figure 2. The novel LXRα agonist **40** does not significantly activate other nuclear receptors. (**A-H**) Luciferase reporter assays in HepG2 cells to measure the activity of PPARα (**A**), PPARγ (**B**), ERα (**C**), ERβ (**D**), FXR (**E**), AhR (**F**), VDR (**G**) and mCAR (**H**) upon 24 h of treatment with 10 µM of compound **40** or compound **46** (Quantification of luciferase, n = 3 biological replicates, data are presented as mean values +/- SD, *** = $P < 0.001$, ** = $P < 0.01$, * = $P < 0.05$, the experiments were repeated with similar results). The following compounds were used as model ligands: VDR agonist calcitriol (100 nM), mice CAR agonist TCPOBOP (10 µM), PPARα agonist fenofibrate (10 µM), PPARγ agonist rosiglitazone (10 µM), estrogen receptor α and β agonist (estradiol 10 µM), farnesoid X receptor's agonist 6-OCA (1 µM) and aryl hydrocarbon receptor agonist 3-MC (5 µM).

4 Effect of sorafenib monotherapy on cell viability

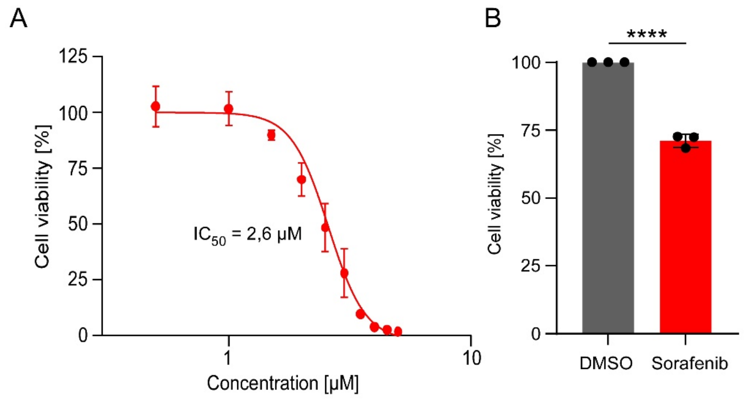

Supplementary Figure 3. Effect of sorafenib monotherapy on cell viability. (**A,B**) Cell viability analysis in Hep3B cells upon 5 d of treatment with different concentrations (**A**) or 2 µM sorafenib (**B**) (quantification of viable cells by cell titer glow, n = 3 independent experiments, data are presented as mean values +/- SD, statistical significance was calculated using two-tailed Student´s t-test, **** = $P < 0.0001$, the experiment was repeated with similar results).

5 Further synthetic description

The following procedure (5.1, 5.2 and 5.3) was used for key compound **40** and similarly for compounds **3**, **22-52.**

5.1 Preparation of amides

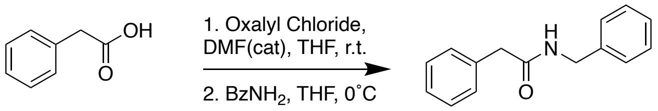

2-Phenylacetic acid (1 eq.) was solubilized in THF and placed in an ice bath. A few drops of dimethylformamide were added, followed by the slow addition of oxalyl chloride (3 eq.), which prompted strong bubbling. The reaction was left stirring until room temperature was achieved. When the carboxylic acid had been completely consumed, the flask was placed in a rotary evaporator, until the only a light ambar oil was left on the flask. The remaining oil was resolubilized in THF and the mixture added dropwise to a flask placed in an ice bath containing the benzylamine (4 eq.) in THF, which led to precipitation of a white solid. The organic solvent was evaporated and water was added. The solid was filtrated under pressure and washed with water to afford a white solid (yield: 60%). ^{1}H NMR (400 MHz, DMSO) δ 8.56 (t, 1H, *J* = 5.5 Hz), 7.32 - 7.22 (m, 10H), 4.27 (d, 2H, *J*= 5.5 Hz), 3.48 (s, 2H). ^{13}C NMR (101 MHz, DMSO) δ 170.1, 139.5, 136.4, 129.0, 128.3, 128.2, 127.2, 126.8, 126.4, 42.4, 42.2. TLC-MS m/z (ESI): 226 [M+H]. HPLC: t_{ret} 6.431 min.

5.2 Preparation of maleimide

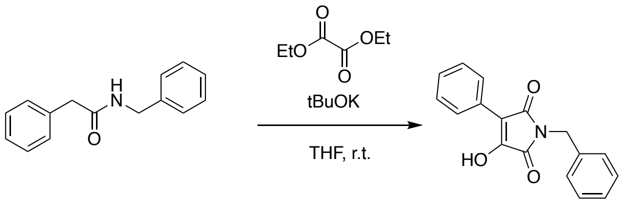

Acetamide (500 mg, 2.2 mmol) and diethyloxalate (330 µL, 2.4 mmol, 1.1 eq) were added to 10 mL of THF. Potassium tert-butoxide (492 mg, 4.4 mmol, 2 eq) was added slowly to the reaction mixture, leading to a change of colour to bright yellow. The reaction was stirred at room-temperature overnight. Work up was made by adding 1M solution of HCl followed by extraction with EtOAc. The organic phase was dried over Na_2SO_4 and evaporated to afford the maleimide as a yellow solid (560 mg, 91%). ^{1}H NMR (400 MHz, DMSO) δ 7.96 (d, 2H, *J* = 8.5 Hz), 7.41 (t, 2H, *J* = 7.7 Hz), 7.34 (t, 2H, *J* = 8.5 Hz), 7.30 - 7.27 (m, 4H), 4.64 (s, 2H). ^{13}C NMR (100 MHz, DMSO) δ 171.0, 166.4, 153.7, 137.0, 129.8, 129.0, 128.6, 128.3, 128.2, 127.4, 127.3, 127.2, 105.6, 40.4.TLC-MS m/z (ESI): 278 [M-H], HPLC: t_{ret} 8.789 min.

5.3 Preparation of Chloro-maleimide

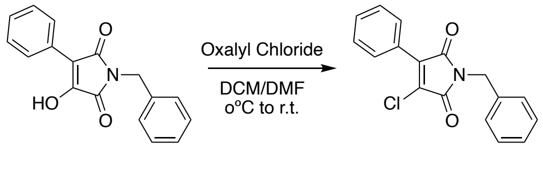

The maleimide (550 mg, 1.97 mmol) was dissolved in a mixture of DCM and DMF (80:20), the flask put on an ice-bath and oxalyl chloride was added slowly (346 µL, 2 eq), leading to strong bubbling. The reaction was stirred at room temperature overnight and monitored via TLC (1:5 EA/Hexane). The reaction mixture was directly adsorbed in Celite and the product purified on flash column chromatography (0-20% EA/PE). The fractions were evaporated to afford the chlorinated product as a white crystalline solid. ^{1}H NMR (400 MHz, DMSO) δ 7.85 - 7.82 (m, 2H), 7.58 – 7.55 (m, 3H), 7.35 - 7.29 (m, 5H), 4.72 (s, 1H). ^{13}C NMR (101 MHz, DMSO) δ 168.0, 164.9, 136.2, 135.0, 130.7, 129.4, 128.6, 128.5, 127.5, 127.4, 126.9, 41.6. HPLC: t_{ret}: 9.102 min.

5.4 Preparation of compounds 16 – 21

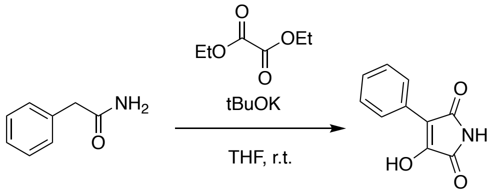

Acetamide (5 g, 37 mmol) and diethyloxalate (5.5 mL, 40.7 mmol, 1.1 eq) were added to 200 mL of THF. Potassium tert-butoxide (8.3 g, 74 mmol, 2 eq) was added slowly to the reaction mixture, leading to a change of colour to bright yellow. The reaction was stirred at room-temperature overnight. Work up was made by adding 1M solution of HCl followed by extraction with EtOAc. The organic phase was dried over Na_2SO_4 and evaporated to afford the maleimide as a yellow solid (6.2 g, 32 mmol, 86%). ^{1}H NMR (400 MHz, DMSO-*d6*) δ 10.66 (s, 1H), 7.93 (d, 2H, *J* = 7.2 Hz,), 7.40 (t, 2H, *J* = 7.7 Hz), 7.28 (t, *J* = 13.2 Hz, 1H). ^{13}C NMR (100 MHz, DMSO-*d6*) δ 172.3, 167.9, 153.2, 129.1, 128.2, 127.4, 106.4. TLC-MS m/z (ESI): 212 [M+Na]. HPLC: t_{ret} 5.021 min.

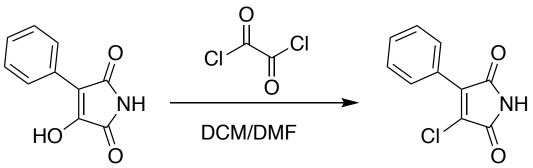

The maleimide (2.0 g, 10.5 mmol) was dissolved in a mixture of DCM and DMF (80:20), the flask put on an ice-bath and oxalyl chloride was added slowly (21.1 mmol, 2 eq), leading to strong bubbling. The reaction was stirred at room temperature overnight and monitored via TLC (1:5 EtOAc/Hexane). The reaction mixture was directly adsorbed in Celite and the product purified on flash column chromatography (0-20% EA/PE). The fractions were evaporated to afford the chlorinated product as a white crystalline solid (1.18 g, 5.7 mmol mmol, 54%). ^{1}H NMR (400 MHz, DMSO-*d6*) δ 11.52 (s, 1H), 7.80 (d, 2H, *J* = 9.8 Hz,), 7.66 – 7.42 (m, 3H). ^{13}C NMR (100 MHz, DMSO-*d6*) δ 169.3, 166.0, 135.4, 131.0, 130.5, 129.4, 128.6, 127.1. HPLC: t_{ret} 6.281 min.

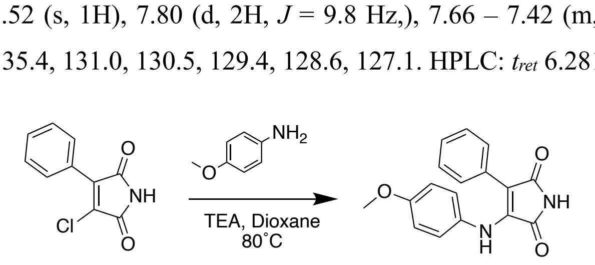

The maleimide (70 mg, 0.338 mmol) was solubilized in dioxane, and then p-methoxyaniline (50 mg, 0.409 mmol, 1.2 eq) was added, followed by triethylamine (0.676, 2 eq.). The reaction mixture was heated at 80˚C overnight. The dioxane was evaporated, and the crude product directly adsorbed into celite. The product was purified by flash column chromatography (0-100% EA/PE). The product was obtained as red powder (70 mg, 70% . ^{1}H NMR (400 MHz, DMSO) δ 10.66 (s, 1H), 9.3 (s, 1H), 7.09 - 7.02 (m, 3H), 6.86 (d, 2H, *J* = 7.0 Hz),

6.64 (d, 2H, J = 8.7 Hz), 6.51 (d, 2H, J = 8.7 Hz), 3.60 (s, 3H). ^{13}C NMR (100 MHz, DMSO) δ 172.9, 169.0, 155.7, 138.4, 130.5, 130.0, 129.3, 126.8, 126.3, 123.3, 113.0, 100.8, 55.2. ESI-HRMS $[M+H]^+$ calculated, 295,10772, found, 295,10936. HPLC t_{ret} 7.184 min.

5.5 Preparation anilines

The intermediates used in the synthesis of compounds **40-52** were prepared according to the two step procedure of 4.2.1 and 4.2.2, from commercial anilines. Exceptions are described below for preparation of compounds **49** and **50**.

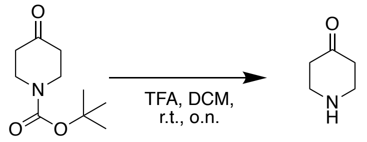

The Boc-protected piperidone was (500 mg, 2.5 mmol) was suspended in 5 mL of DCM. 2.5 mL of trifluoracetic acid were added and the reaction stirred at r.t. overnight. The solvent was then evaporated and the product precipitated with HCl in Dioxane, to afford beige solid (300 mg, 88%). The product was used in the subsequent reactions in 4.2.1 and 4.2.2.

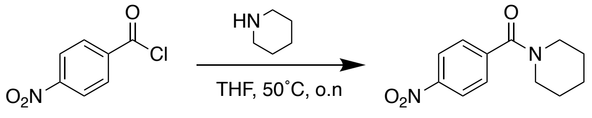

4-nitrobenzoyl chloride (228 mg, 1.23 mmol, 1.0 eq.) was solubilized in dry THF. The reaction mixture was flushed with nitrogen and piperidine (1.1 eq) was added. The reaction was heated to 50˚C and stirred overnight. The reaction mixture was evaporated and the product used further step (4.2.2) without any purification (200 mg, 79%).

5.5.1 Aromatic substitution

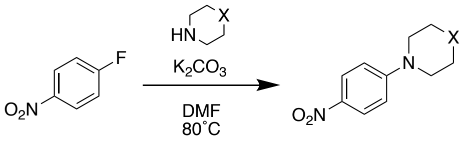

1-fluoro-4-nitrobenzene (1eq.) was added to DMF, followed by the corresponding piperidine or amine (1.2 eq.) and K_2CO_3 (2 eq.). The reaction and heated to 80˚C and stirred overnight. Upon completion, the reaction mixture was cooled down to room temperature and water was added. When precipitation occurred, the solid was filtrated under vacuum. Otherwise, the product was extracted with EtOAc, the organic solution dried over Na_2SO_4, and the crude product adsorbed into celite. Purification was made by flash column chromatography (0 – 50% EtOAc/PE).

5.5.2 Reduction of nitro group

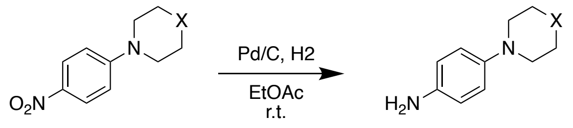

The corresponding nitrophenyl intermediates obtained previously (1 eq.) were solubilized in EtOAc and 10% m/m of Pd/C was added. The reaction mixture was initially flushed with nitrogen, and then with hydrogen and left stirring at room temperature from 2h – 18h. The reaction mixture was filtrated over celite to remove palladium residues and washed with EtOAc. If needed, the product was adsorbed into celite and purified over flash column chromatography (0 – 80% EtOAc/PE). The desired aniline was subsequently used in the preparation of compound **40-52**.

5.6 Preparation of compound 21

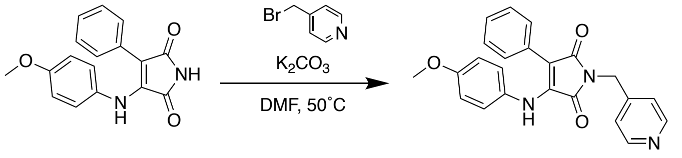

The non-substituted maleimide intermediate **15** was reacted with *p*-methoxyaniline according to the method previously described to afford 25. Compound 25 was solubilized in DMF in a round-bottom flask, and 4-(bromomethyl)pyridine (1.2 eq.) and K_2CO_3 (2 eq.) were combined The solution was heated at 50˚C for 2h. Reaction control was performed via TLC and HPLC. After the reaction finished, the reaction mixture was quenched with water and extracted with EA and brine. The organic phase was dried over sodium sulfate, and the solvent evaporated. The crude oil obtained was adsorbed into Celite, and purification was performed with flash chromatography (20-80% EA/PE). The product was obtained as a light brown powder (yield: 43%).

5.7 **Preparation of compound 26**

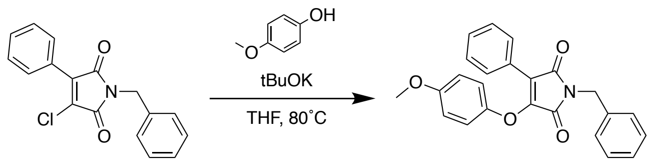

The chlorinated maleimide 9 was solubilized in THF, and to the solution, *p*-methoxyphenol (1.2 eq) and tBuOK (2 eq) was added. The solution was heated to 80˚C and stirred overnight. Reaction control was performed via TLC and HPLC. After the reaction finished, THF was evaporated and the slurry crude mixture solubilized with water, and the extracted with EA. The organic phase was dried over sodium sulfate, and the solvent evaporated. The crude oil obtained was adsorbed into Celite, and purification was performed with flash chromatography (20-80% EA/PE). The product was obtained was a yellow solid (yield: 38%)

5.8 Preparation. of compound 27

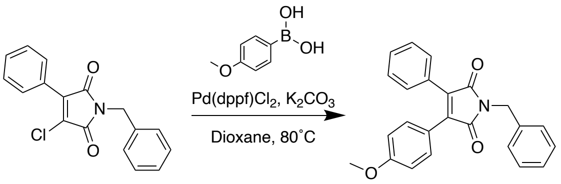

The chlorinated intermediate 9 was solubilized in dioxane, and (*p*-methoxy)phenylboronic acid (2 eq.) and K_2CO_3 (3 eq.) were solubilized in dioxane. The reaction mixture was put under inert atmosphere, and then Pd(dppf)Cl_2 (0.05 eq.) was added. The reaction. Mixture was heated to 100˚C overnight. The reaction was quenched by addition of NH_4Cl saturated solution, followed by extraction with EA. The organic phase was dried over sodium sulfate, and the solvent evaporated. The crude oil obtained was adsorbed into Celite, and purification was performed with flash chromatography (20-50% EA/PE). The product was obtained was a yellow solid (yield: 85%).

5.9 Preparation of compound 46

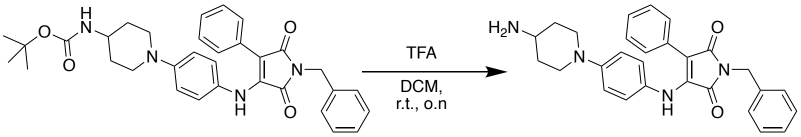

Compound 45 (100 mg, 0.18 mmol) was suspended in 10 mL of DCM, and 5 mL of TFA were added. The reaction was stirred overnight at room temperature. The solvent was evaporated and the product precipitated with HCl in dioxane as a yellow solid (130 mg, 73%).

5.10 Preparation of compound 44

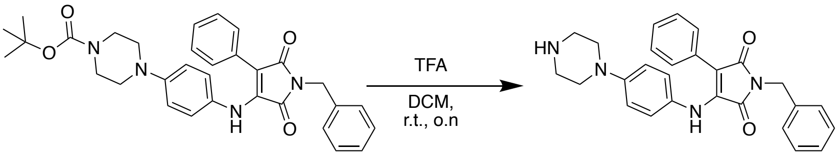

The Boc-protected intermediate (180 mg, 0.33 mmol) was suspended in 10 mL of DCM, and 5 mL of TFA were added. The reaction was stirred overnight at room temperature. The solvent was evaporated and the product precipitated with HCl in dioxane as a green solid (120 mg, 83%).

6 NMR spectra of final compounds

Compound 3

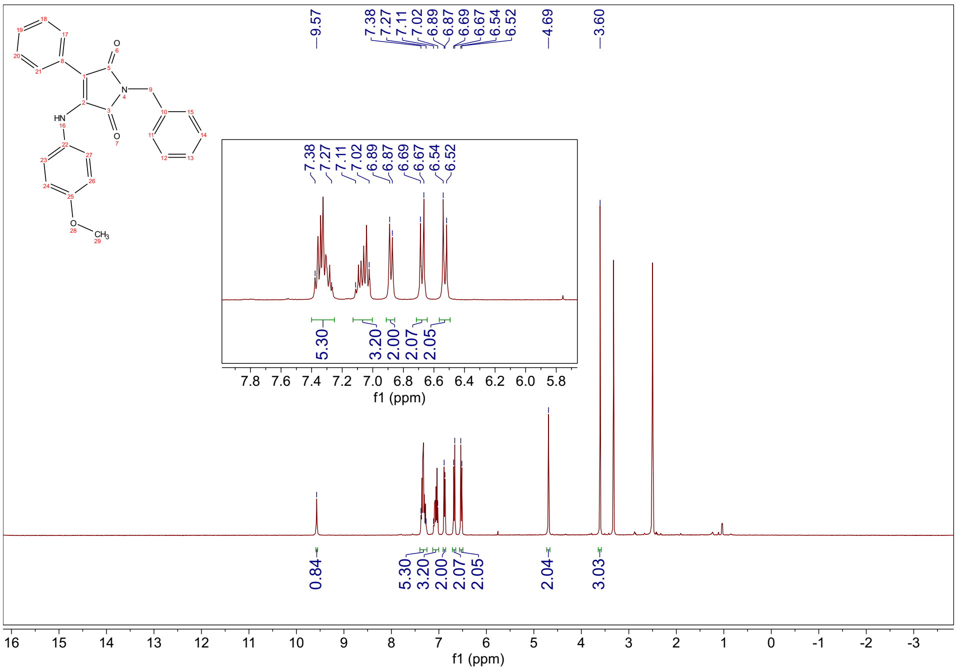

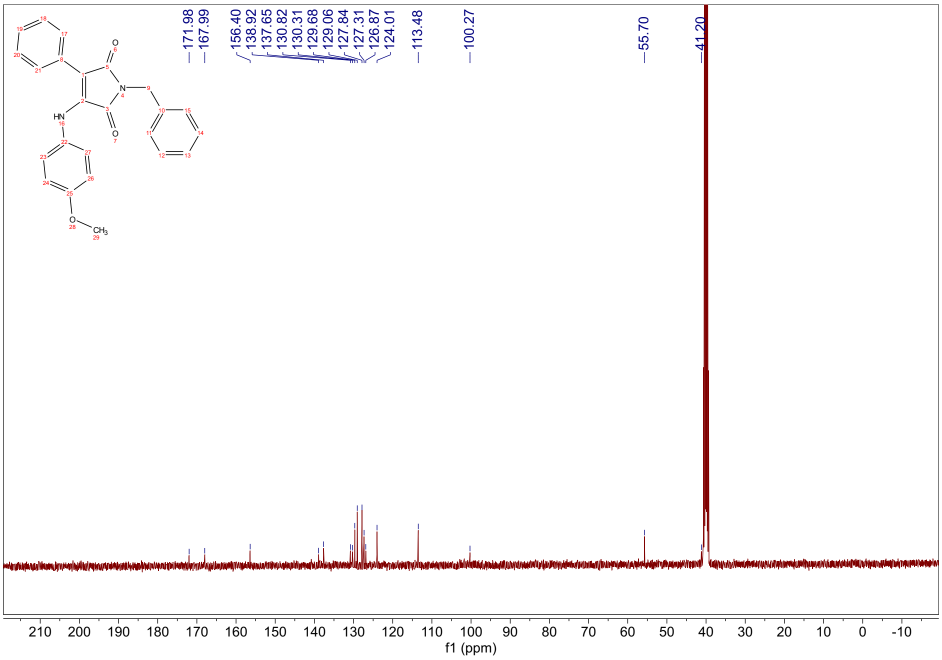

Compound 16

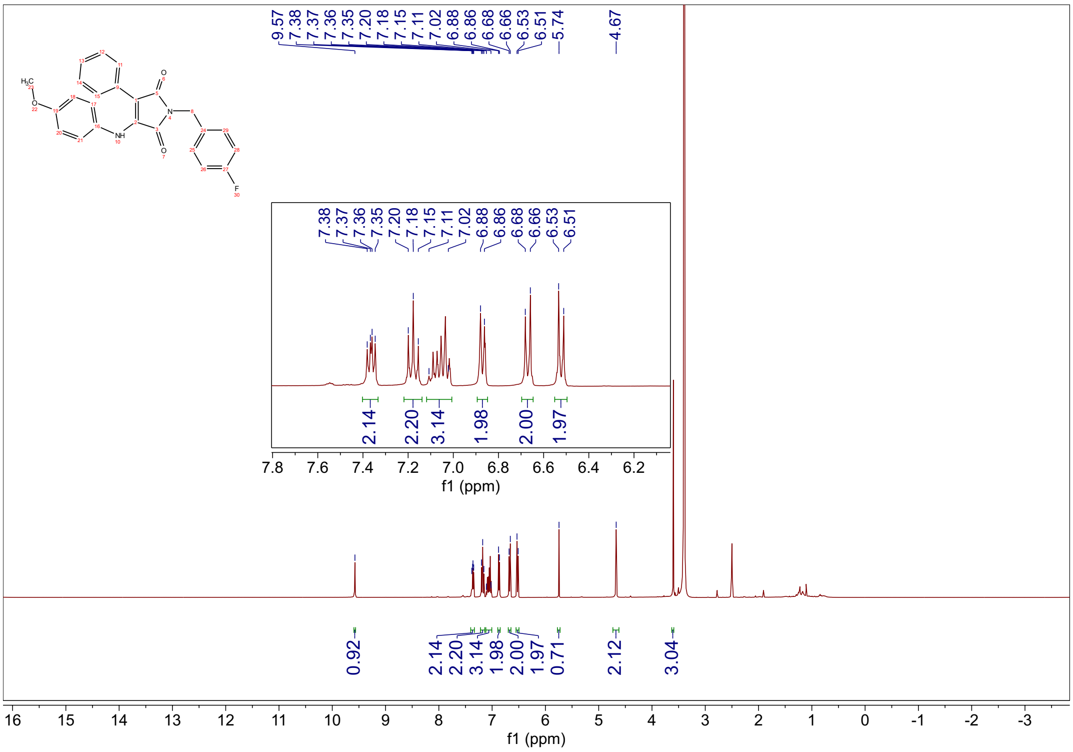

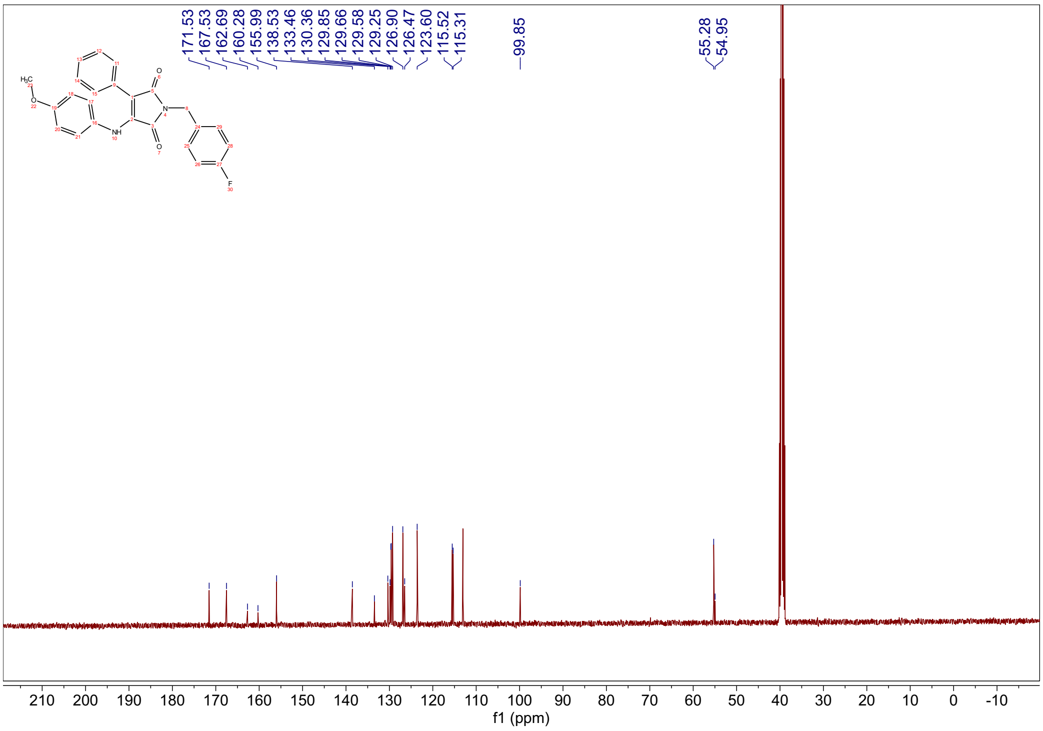

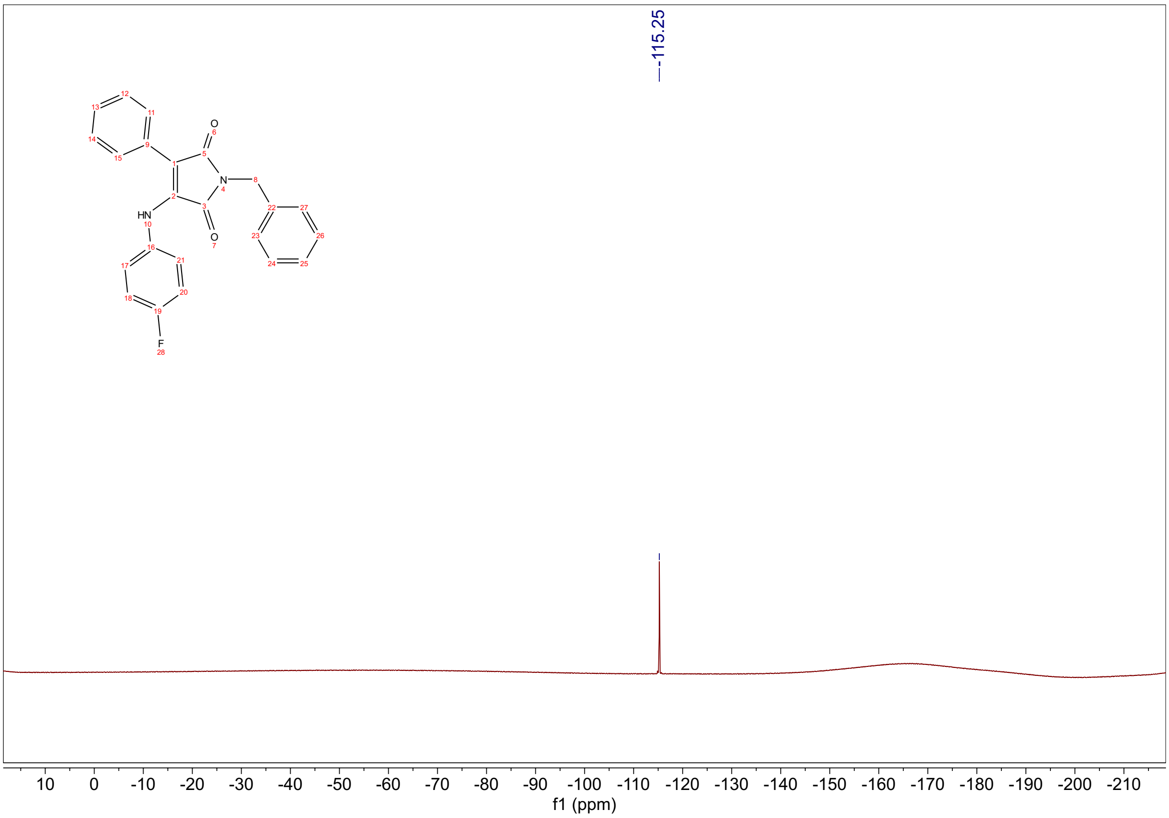

-115.25
HN
F
O
N
10 0 -10 -20 -30 -40 -50 -60 -70 -80 -90 -100 -110 -120 -130 -140 -150 -160 -170 -180 -190 -200 -210
f1 (ppm)

Compound 17

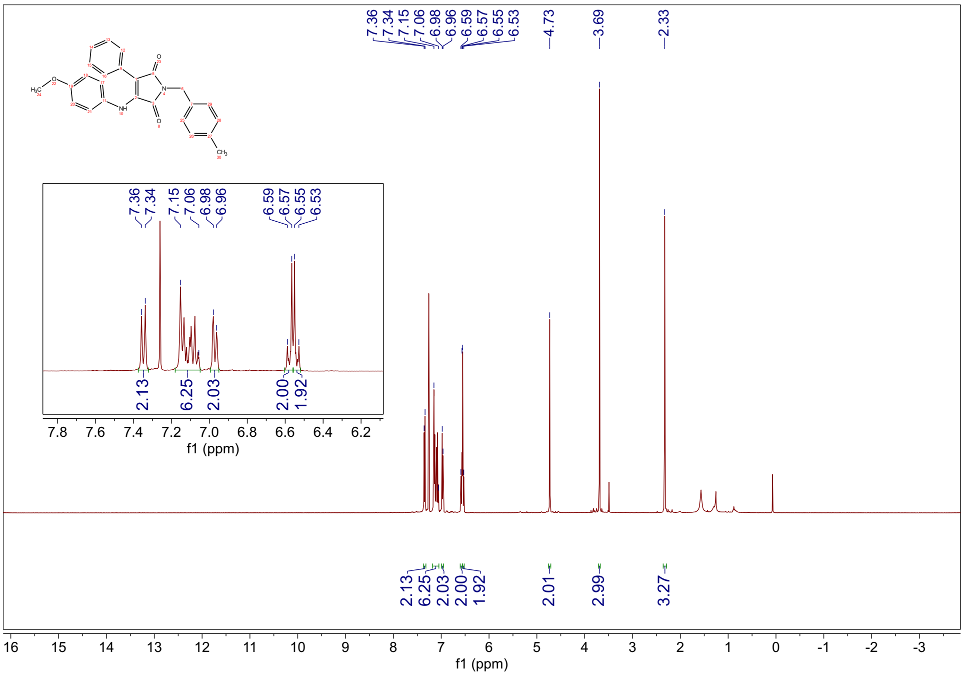
7.36
7.34
7.15
7.06
6.98
6.96
6.59
6.57
6.55
6.53
4.73
3.69
2.33
2.13
6.25
2.03
2.00
1.92
2.01
2.99
3.27
f1 (ppm)

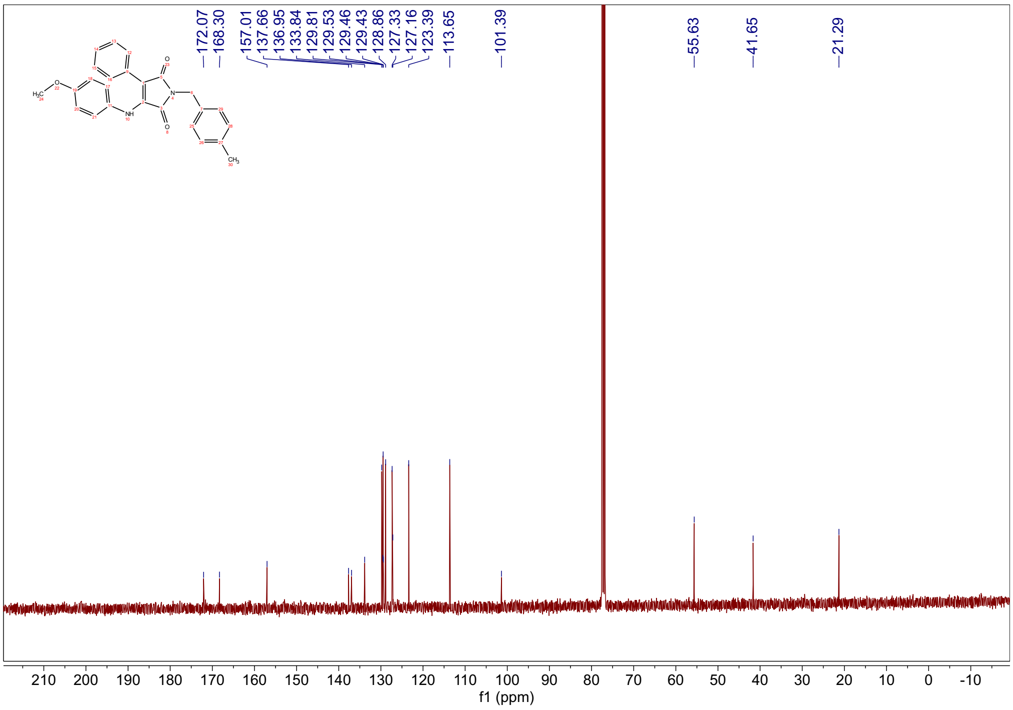
172.07
168.30
157.01
137.66
136.95
133.84
129.81
129.53
129.46
129.43
128.86
127.33
127.16
123.39
113.65
101.39
55.63
41.65
21.29
f1 (ppm)

Compound 18

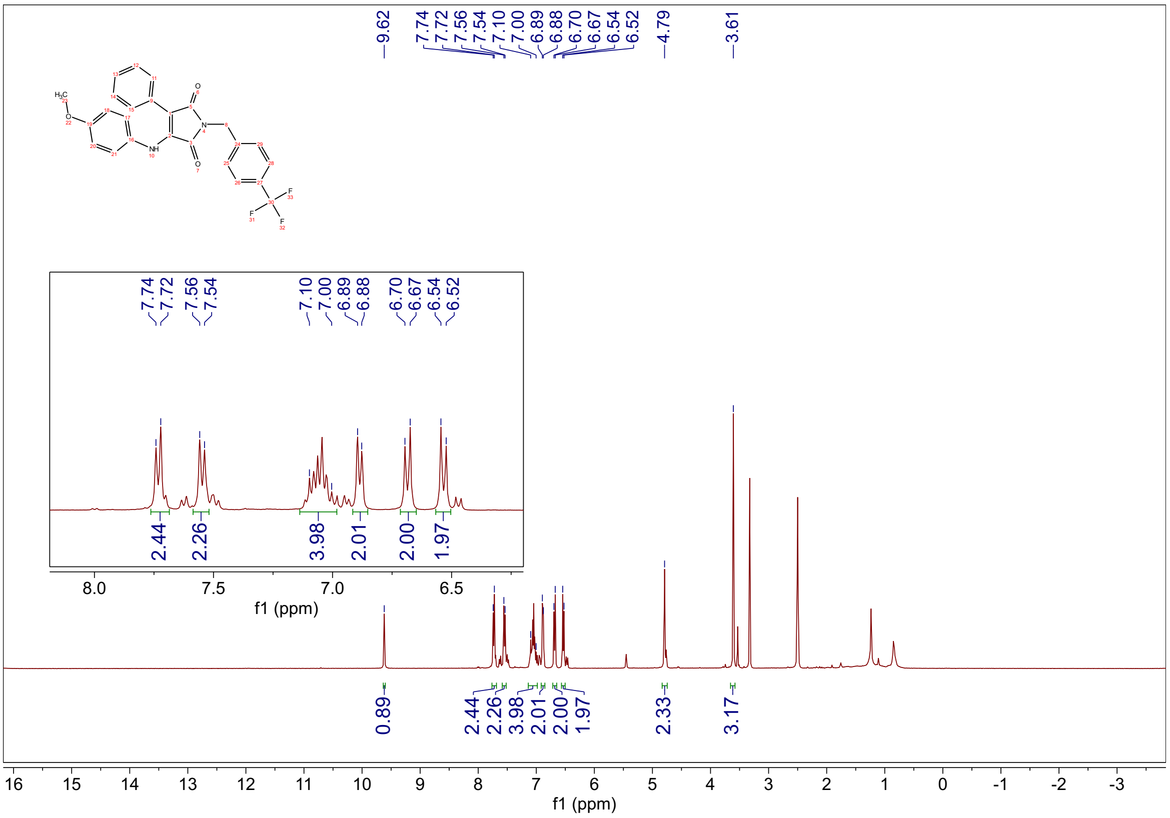
9.62
7.74
7.72
7.56
7.54
7.10
7.00
6.89
6.88
6.70
6.67
6.54
6.52
4.79
3.61
0.89
2.44
2.26
3.98
2.01
2.00
1.97
2.33
3.17
f1 (ppm)

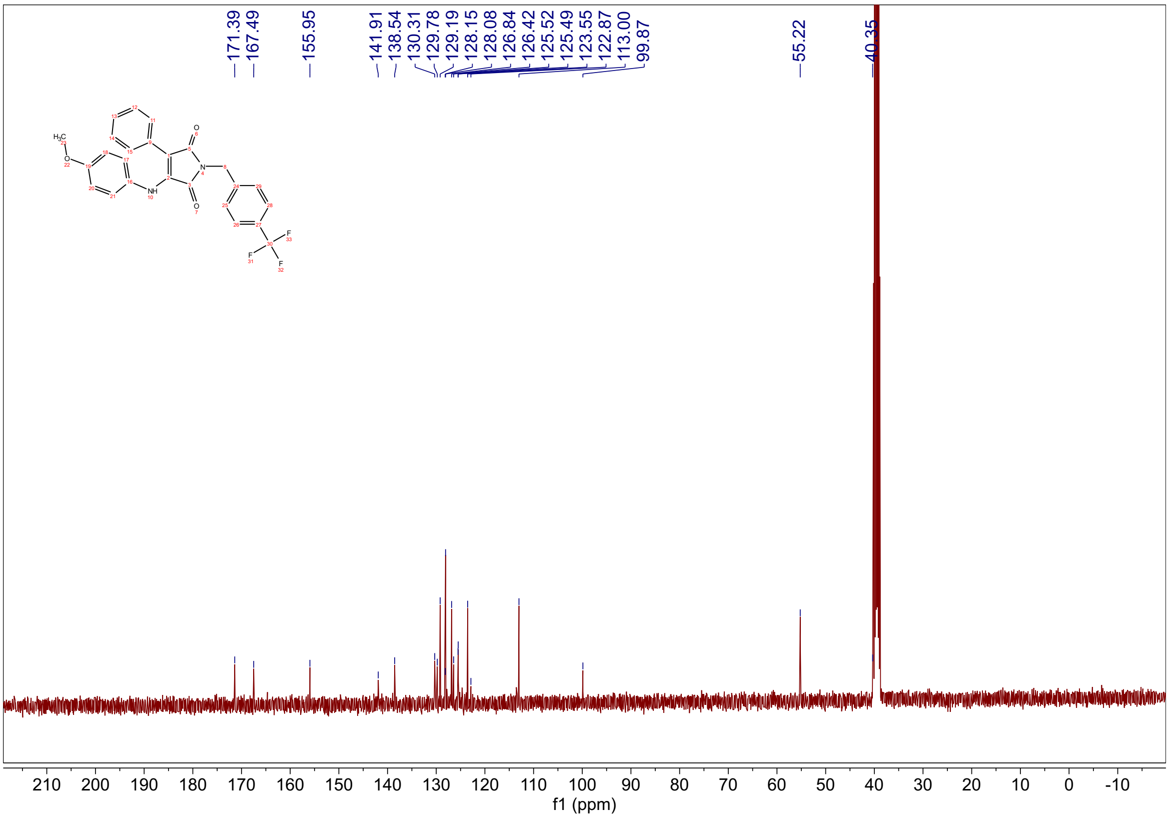
171.39
167.49
155.95
141.91
138.54
130.31
129.78
129.19
128.15
128.08
126.84
126.42
125.52
125.49
123.55
122.87
113.00
99.87
55.22
40.35
f1 (ppm)

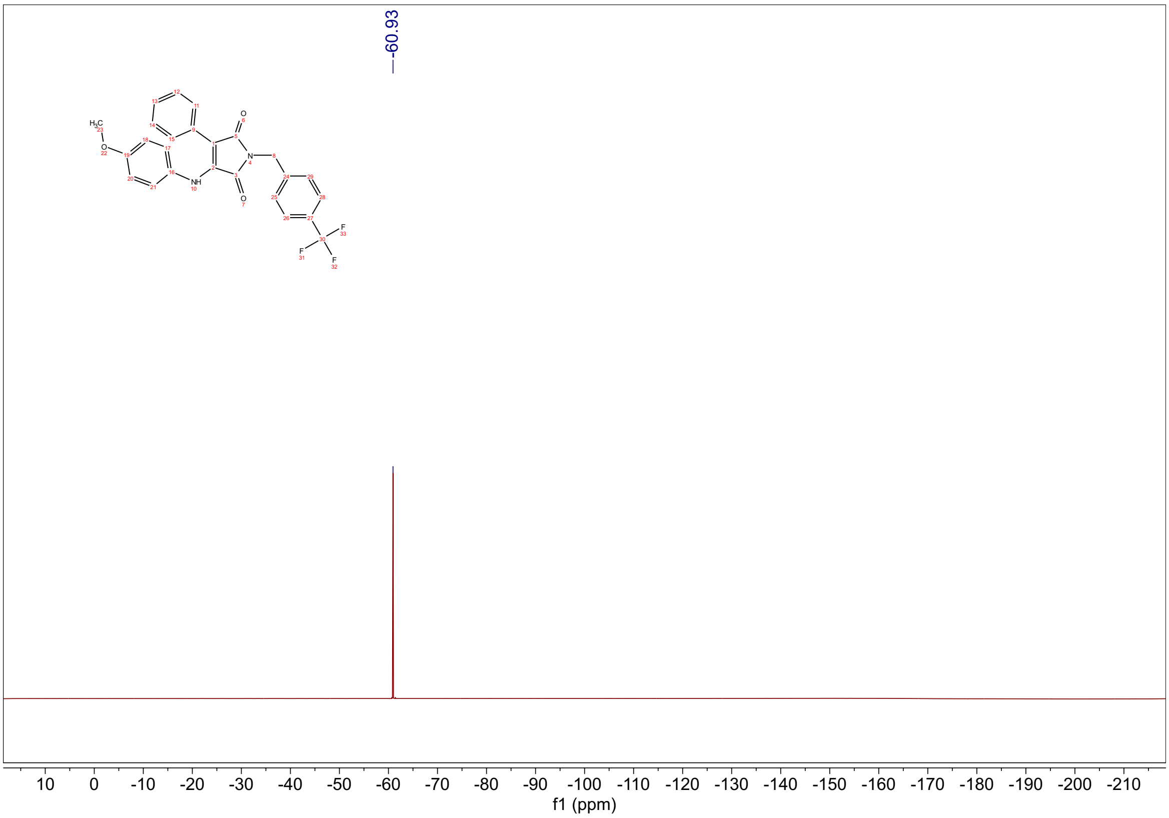

--60.93
10
0
-10
-20
-30
-40
-50
-60
-70
-80
-90
-100
-110
-120
-130
-140
-150
-160
-170
-180
-190
-200
-210
f1 (ppm)

Compound 19

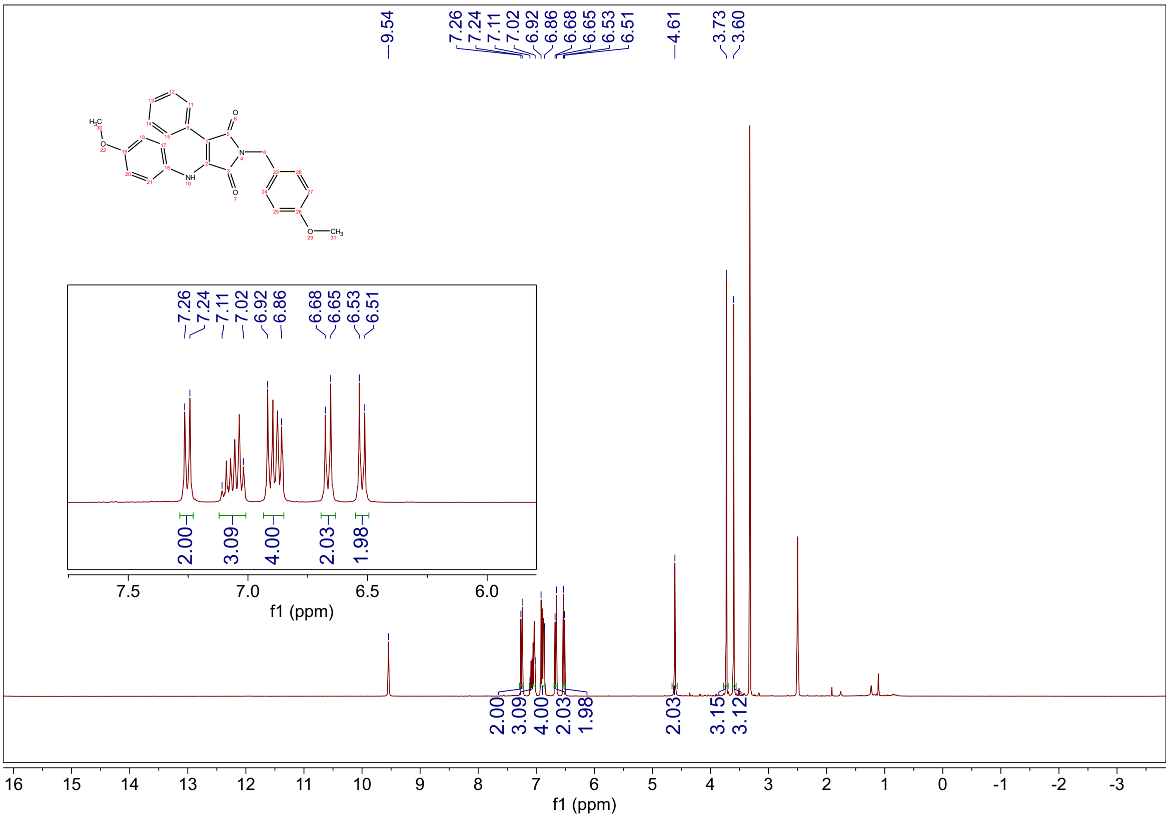

9.54
7.26
7.24
7.11
7.02
6.92
6.86
6.68
6.65
6.53
6.51
4.61
3.73
3.60
2.00
3.09
4.00
2.03
1.98
2.03
3.15
3.12
f1 (ppm)

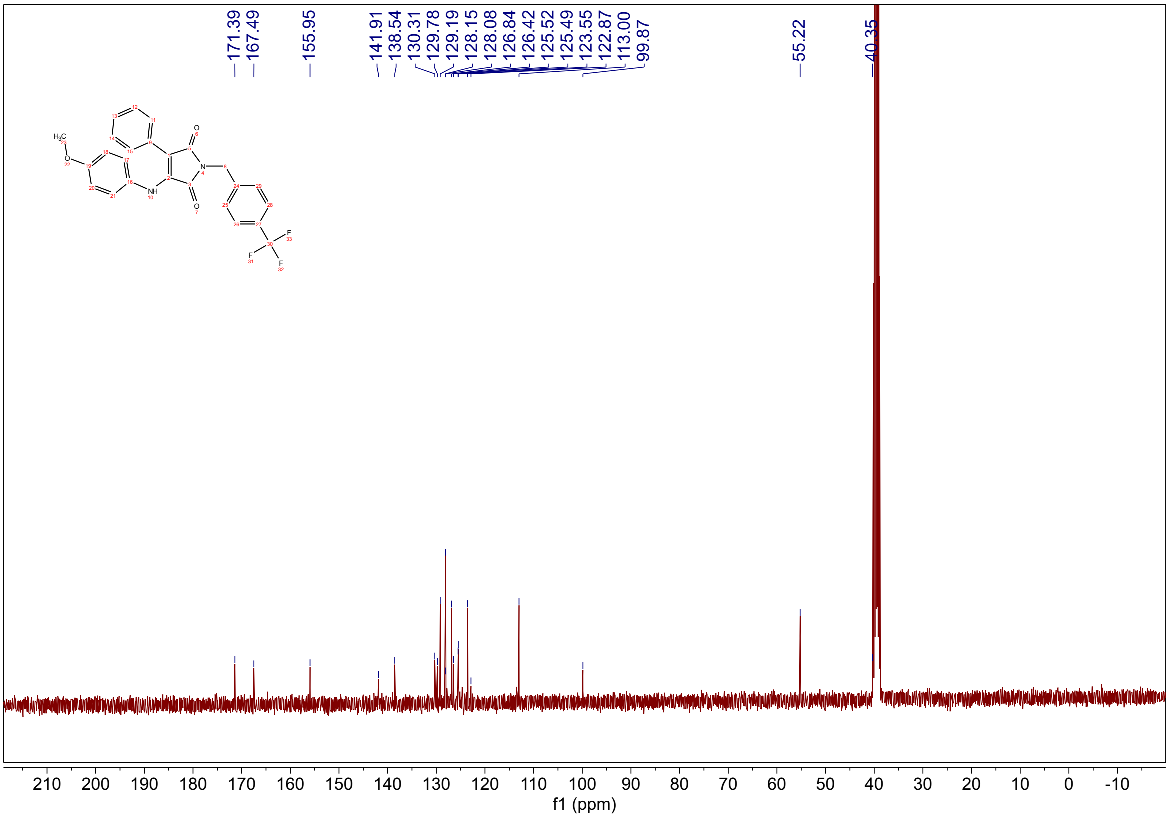

171.39
167.49
155.95
141.91
138.54
130.31
129.78
129.19
128.15
128.08
126.84
126.42
125.52
125.49
123.55
122.87
113.00
99.87
55.22
40.35
f1 (ppm)

Compound 20

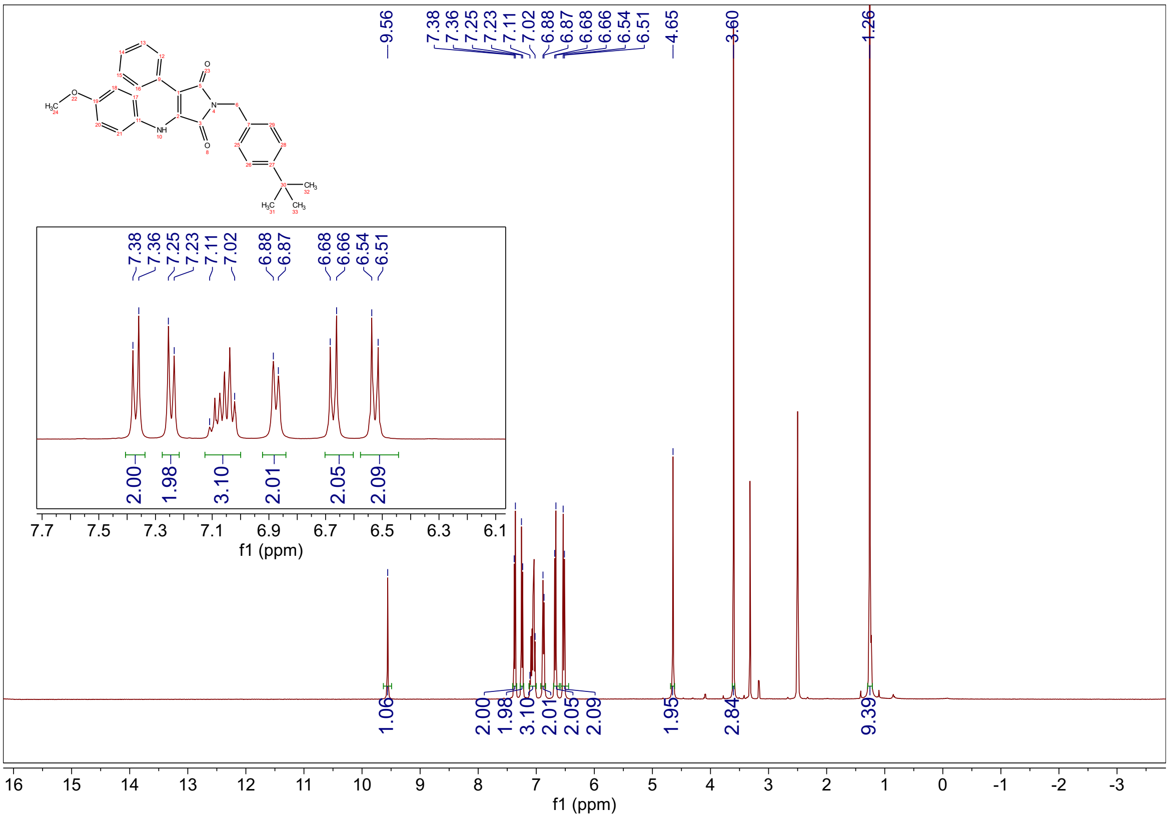
9.56
7.38
7.36
7.25
7.23
7.11
7.02
6.88
6.87
6.68
6.66
6.54
6.51
4.65
3.60
1.26
1.06
2.00
1.98
3.10
2.01
2.05
2.09
1.95
2.84
9.39
f1 (ppm)

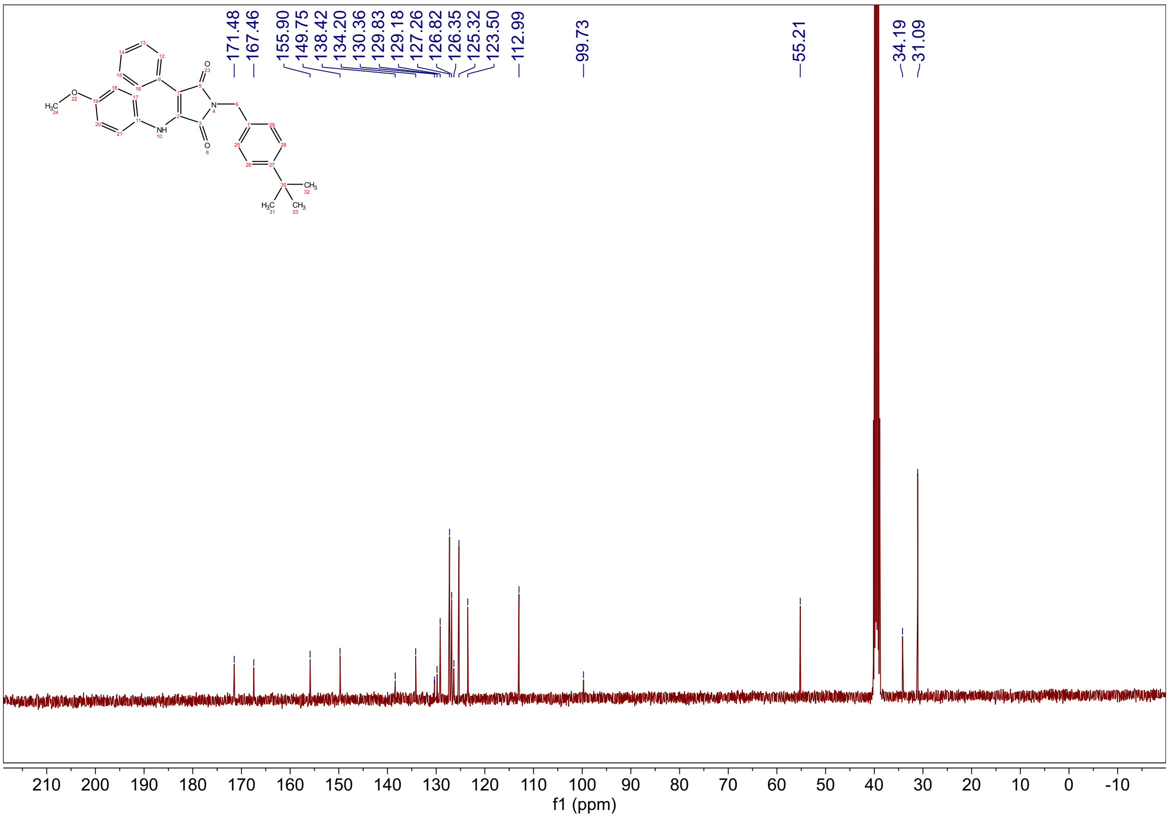
171.48
167.46
155.90
149.75
138.42
134.20
130.36
129.83
129.18
127.26
126.82
126.35
125.32
123.50
112.99
99.73
55.21
34.19
31.09
f1 (ppm)

Compound 21

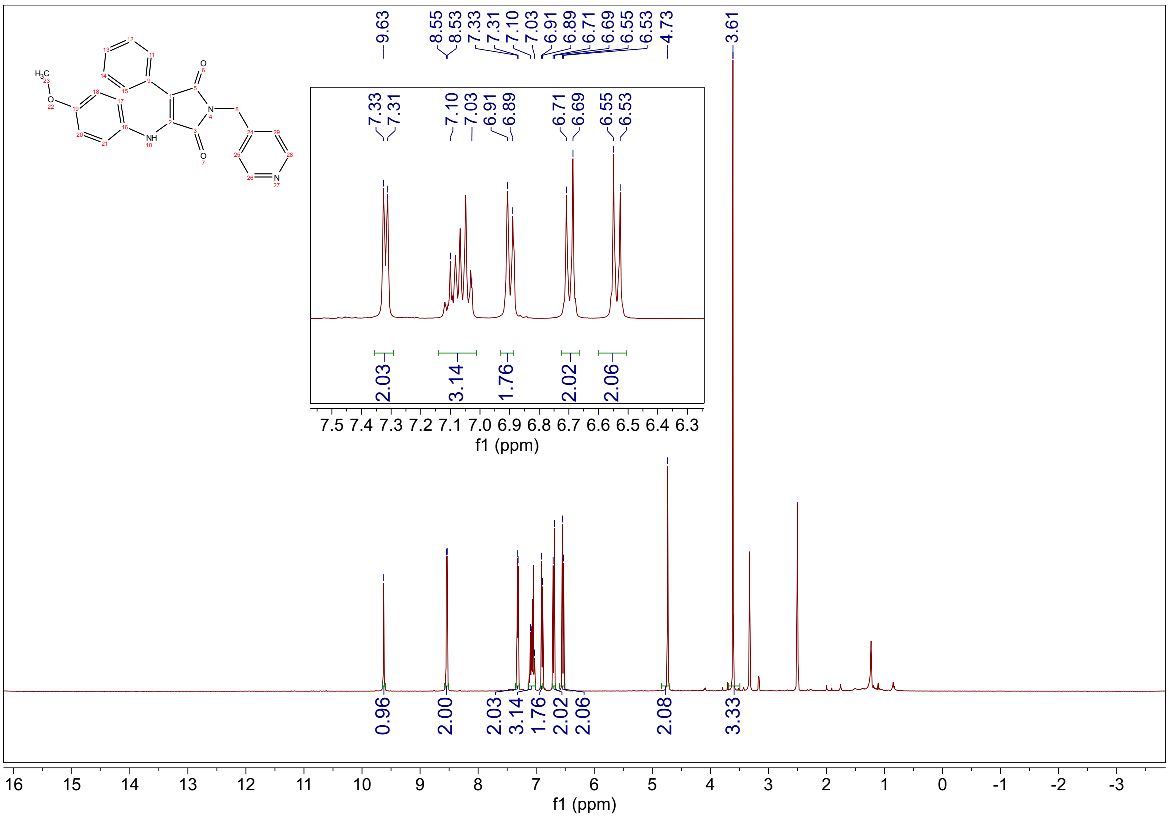

9.63
8.55
8.53
7.33
7.31
7.10
7.03
6.91
6.89
6.71
6.69
6.55
6.53
4.73
3.61
0.96
2.00
2.03
3.14
1.76
2.02
2.06
2.08
3.33
f1 (ppm)

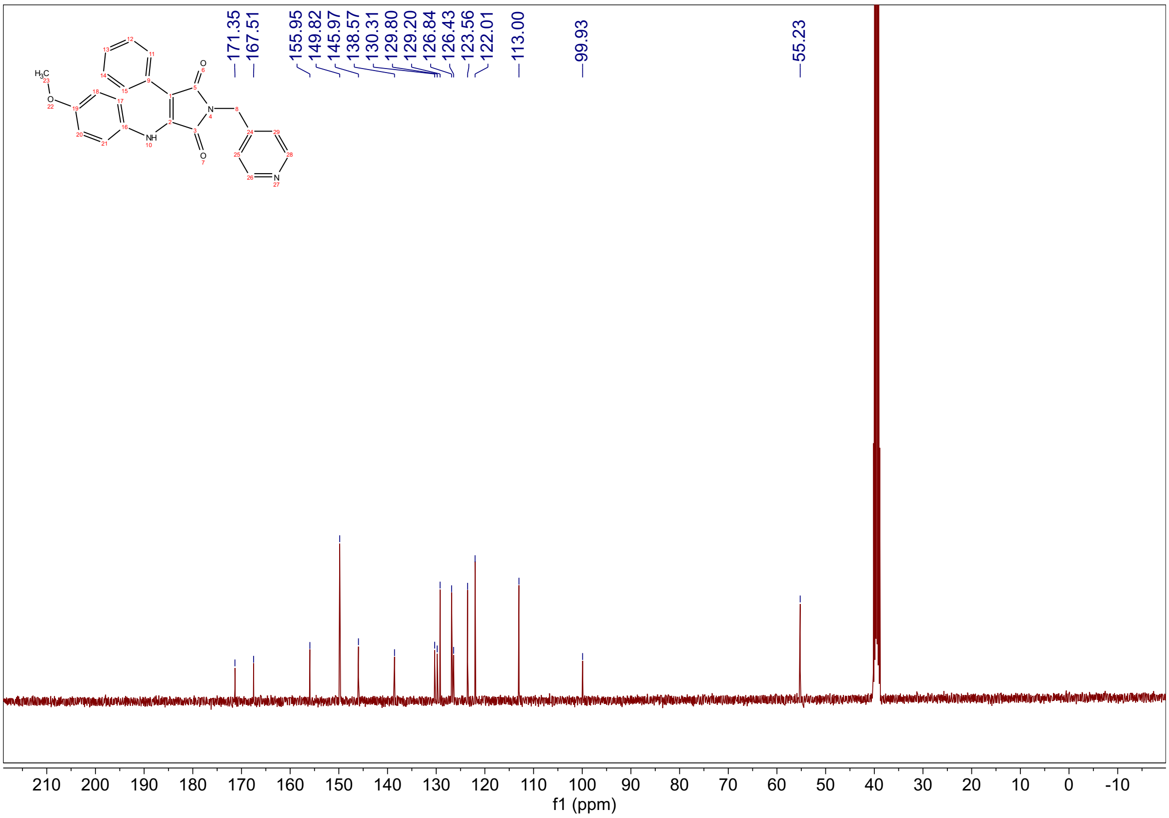

171.35
167.51
155.95
149.82
145.97
138.57
130.31
129.80
129.20
126.84
126.43
123.56
122.01
113.00
99.93
55.23
f1 (ppm)

Compound 22

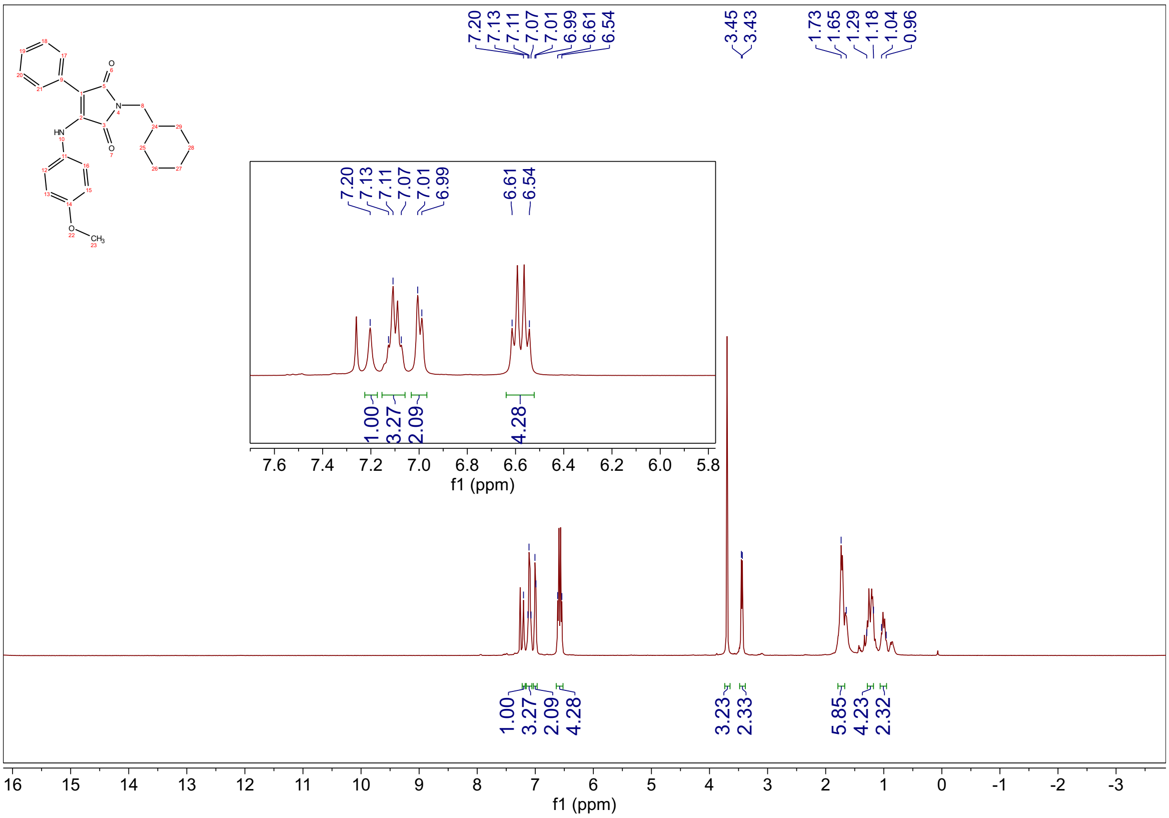
7.20
7.13
7.11
7.07
7.01
6.99
6.61
6.54
3.45
3.43
1.73
1.65
1.29
1.18
1.04
0.96
1.00
3.27
2.09
4.28
3.23
2.33
5.85
4.23
2.32
f1 (ppm)

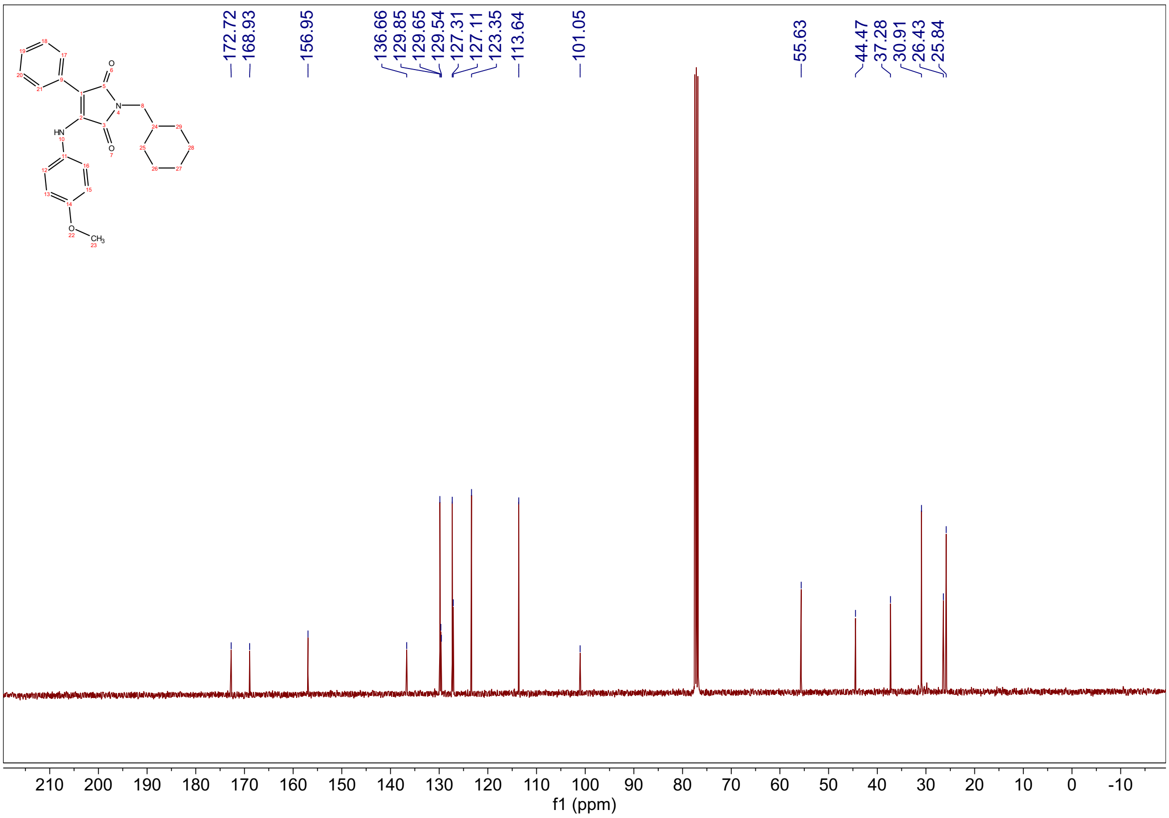
172.72
168.93
156.95
136.66
129.85
129.65
129.54
127.31
127.11
123.35
113.64
101.05
55.63
44.47
37.28
30.91
26.43
25.84
f1 (ppm)

Compound 23

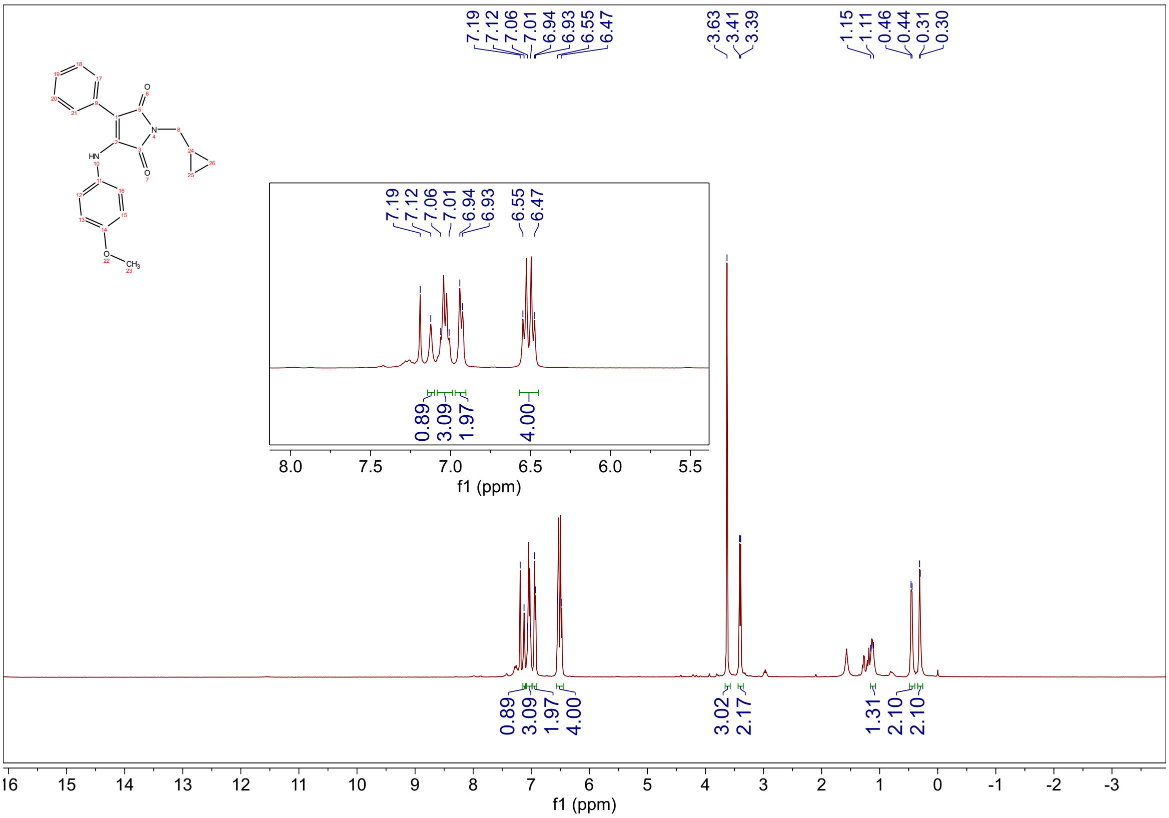
7.19
7.12
7.06
7.01
6.94
6.93
6.55
6.47
3.63
3.41
3.39
1.15
1.11
0.46
0.44
0.31
0.30
0.89
3.09
1.97
4.00
3.02
2.17
1.31
2.10
2.10
f1 (ppm)

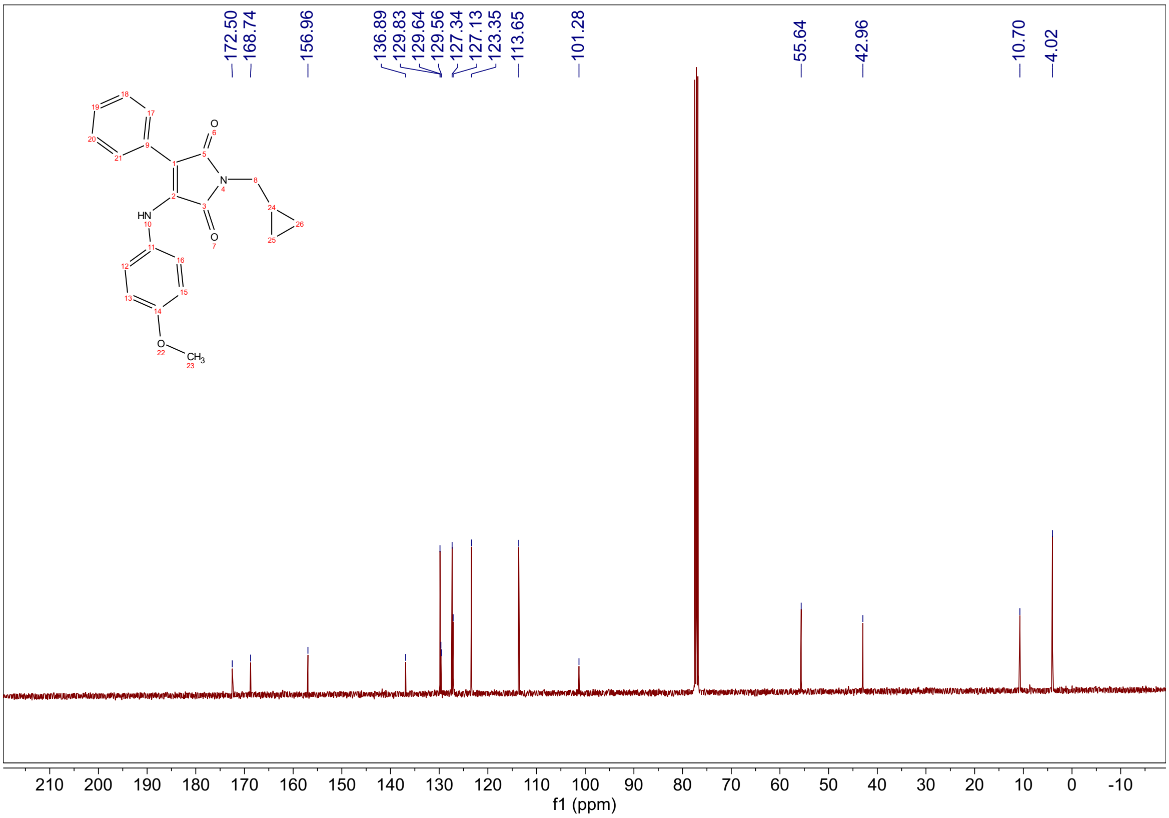
172.50
168.74
156.96
136.89
129.83
129.64
129.56
127.34
127.13
123.35
113.65
101.28
55.64
42.96
10.70
4.02
f1 (ppm)

Compound 24

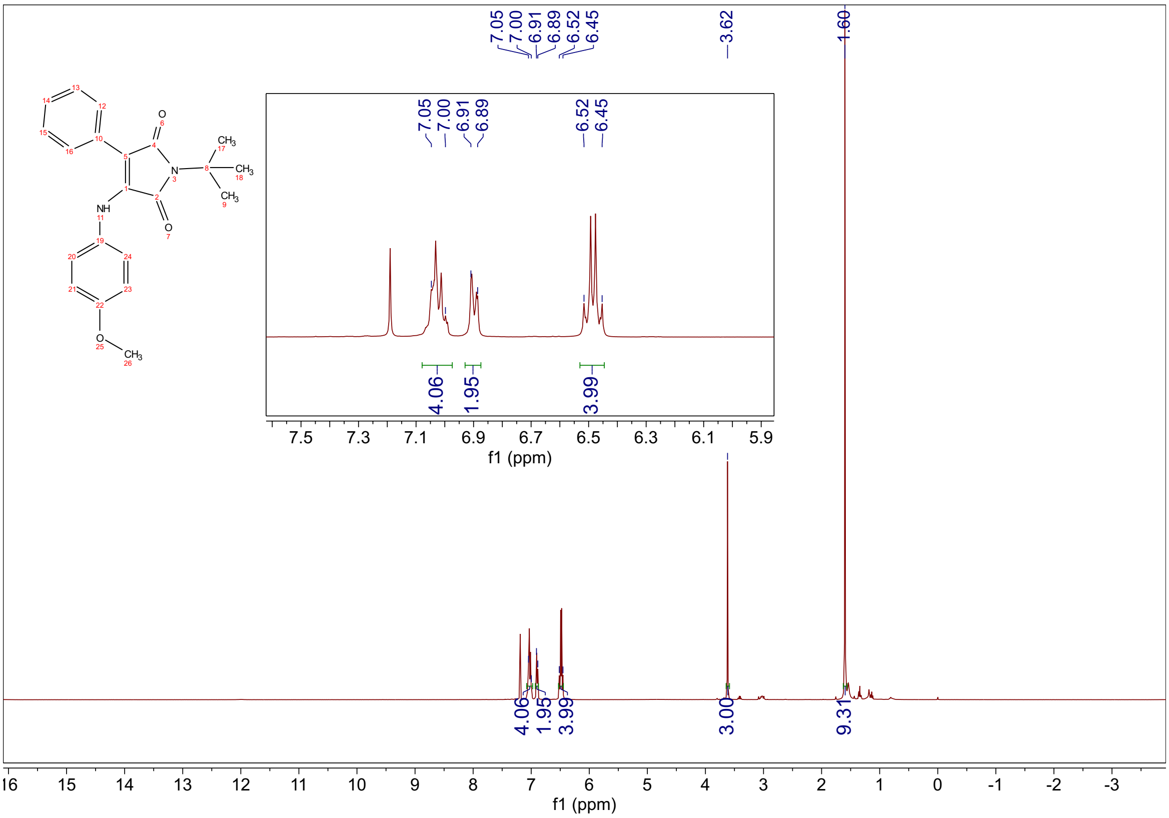

7.05
7.00
6.91
6.89
6.52
6.45
3.62
1.60
4.06
1.95
3.99
3.00
9.31
f1 (ppm)

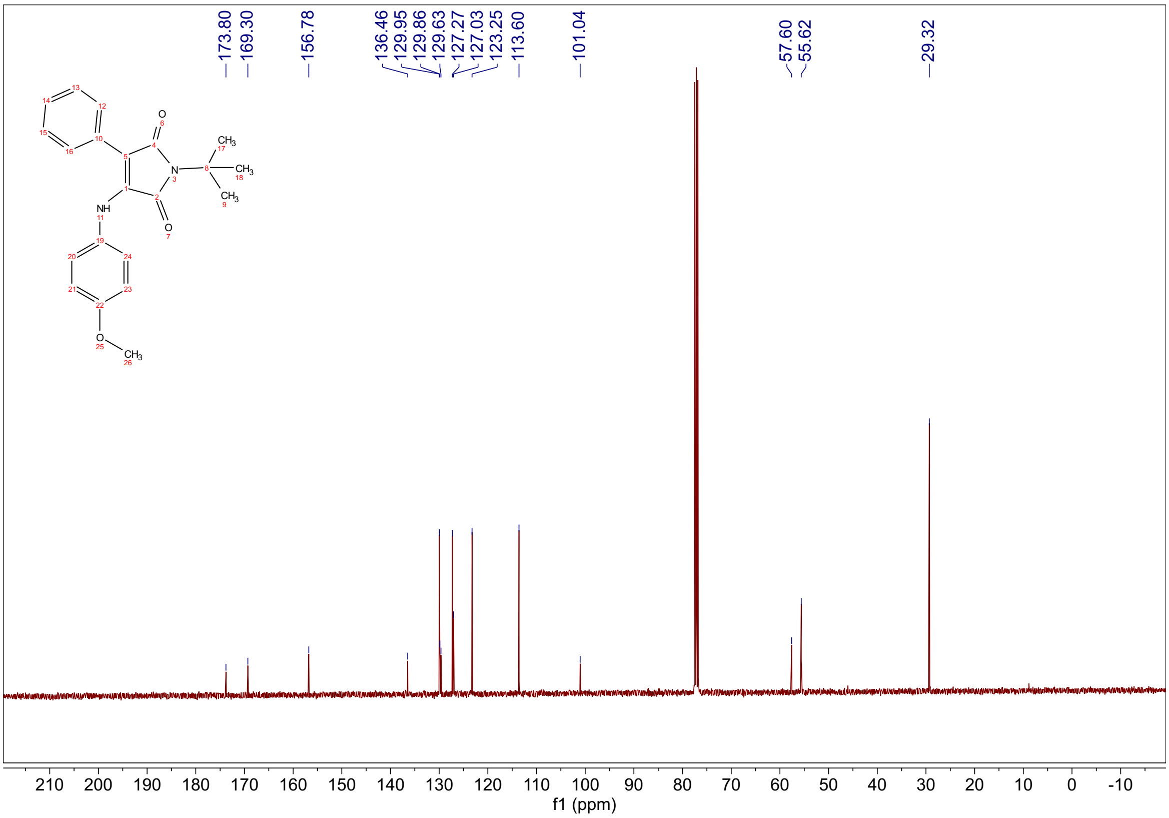

173.80
169.30
156.78
136.46
129.95
129.86
129.63
127.27
127.03
123.25
113.60
101.04
57.60
55.62
29.32
f1 (ppm)

Compound 25

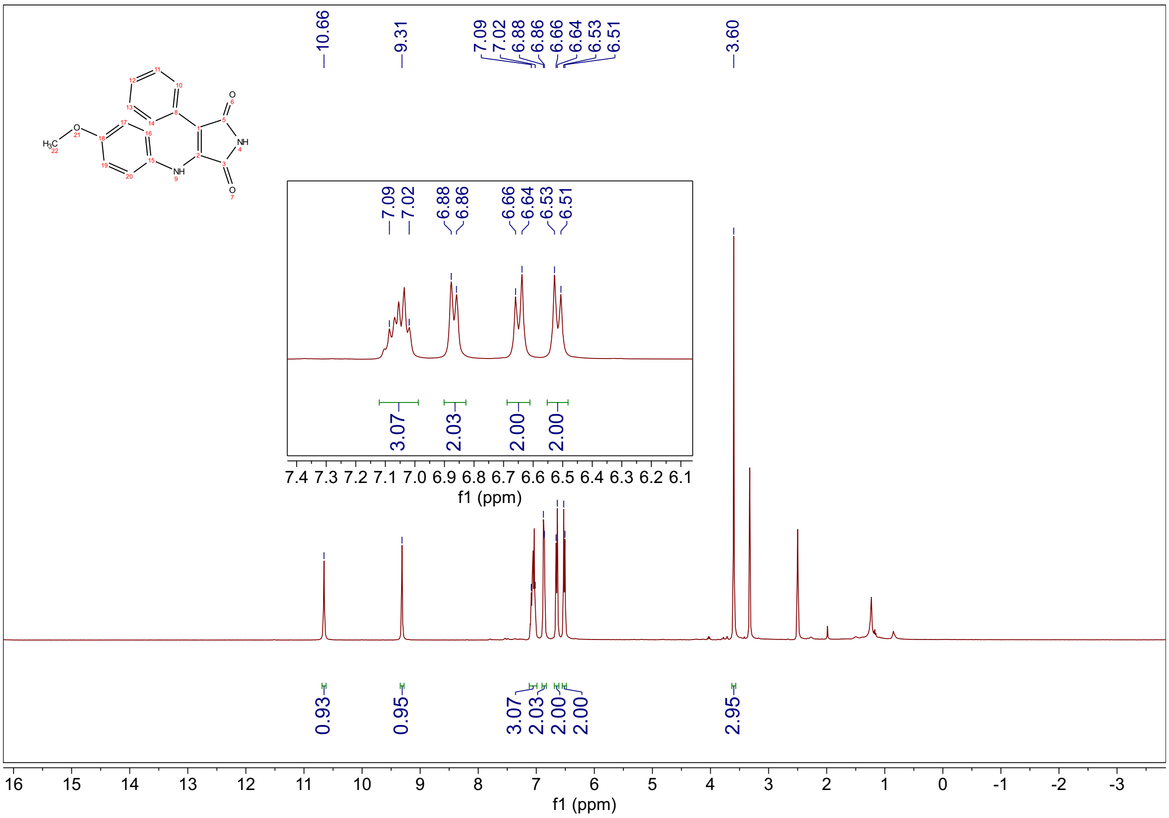
10.66
9.31
7.09
7.02
6.88
6.86
6.66
6.64
6.53
6.51
3.60
0.93
0.95
3.07
2.03
2.00
2.00
2.95
f1 (ppm)

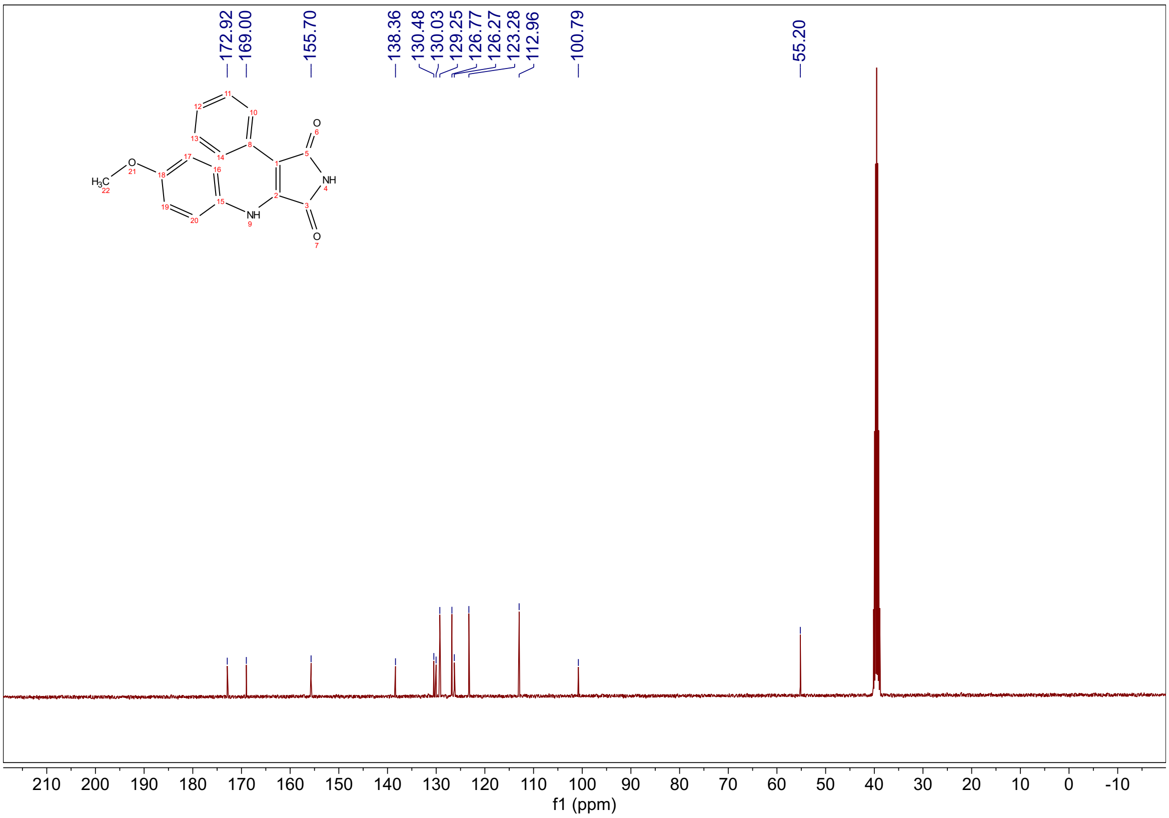
172.92
169.00
155.70
138.36
130.48
130.03
129.25
126.77
126.27
123.28
112.96
100.79
55.20
f1 (ppm)

Compound 26

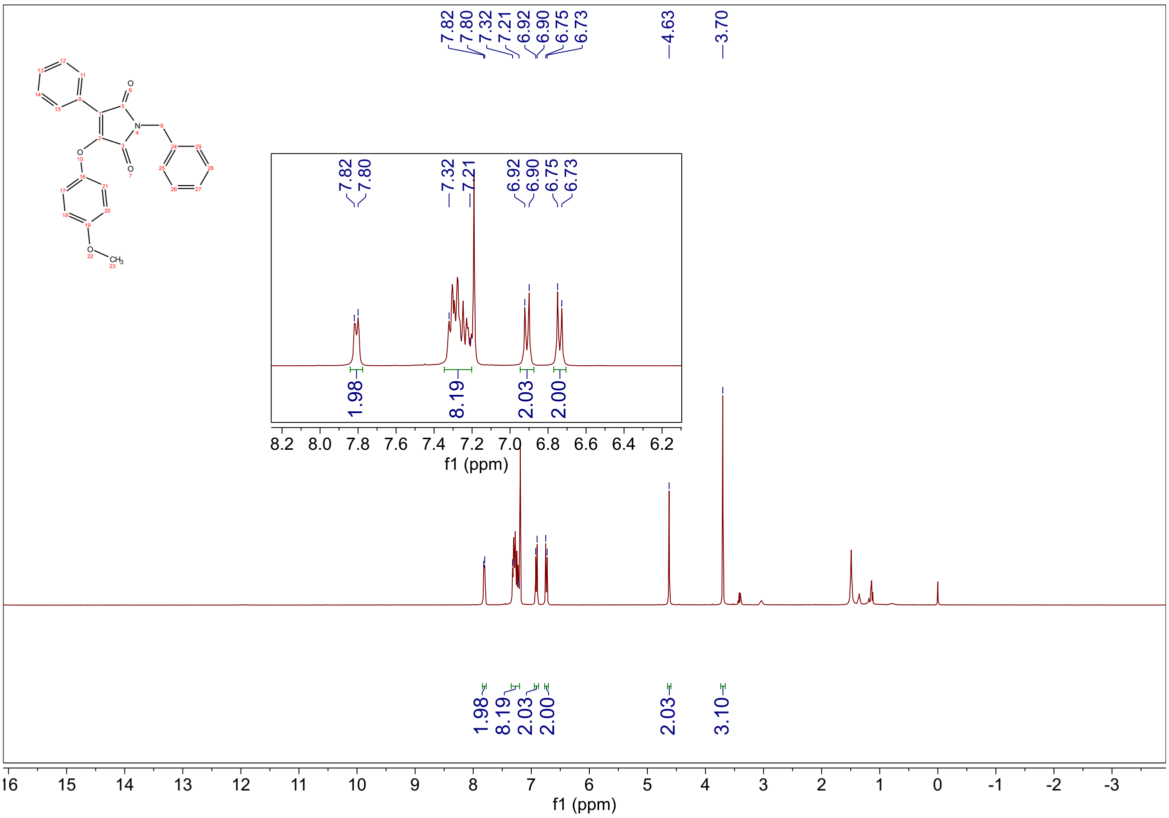
7.82
7.80
7.32
7.21
6.92
6.90
6.75
6.73
4.63
3.70
1.98
8.19
2.03
2.00
2.03
3.10
f1 (ppm)

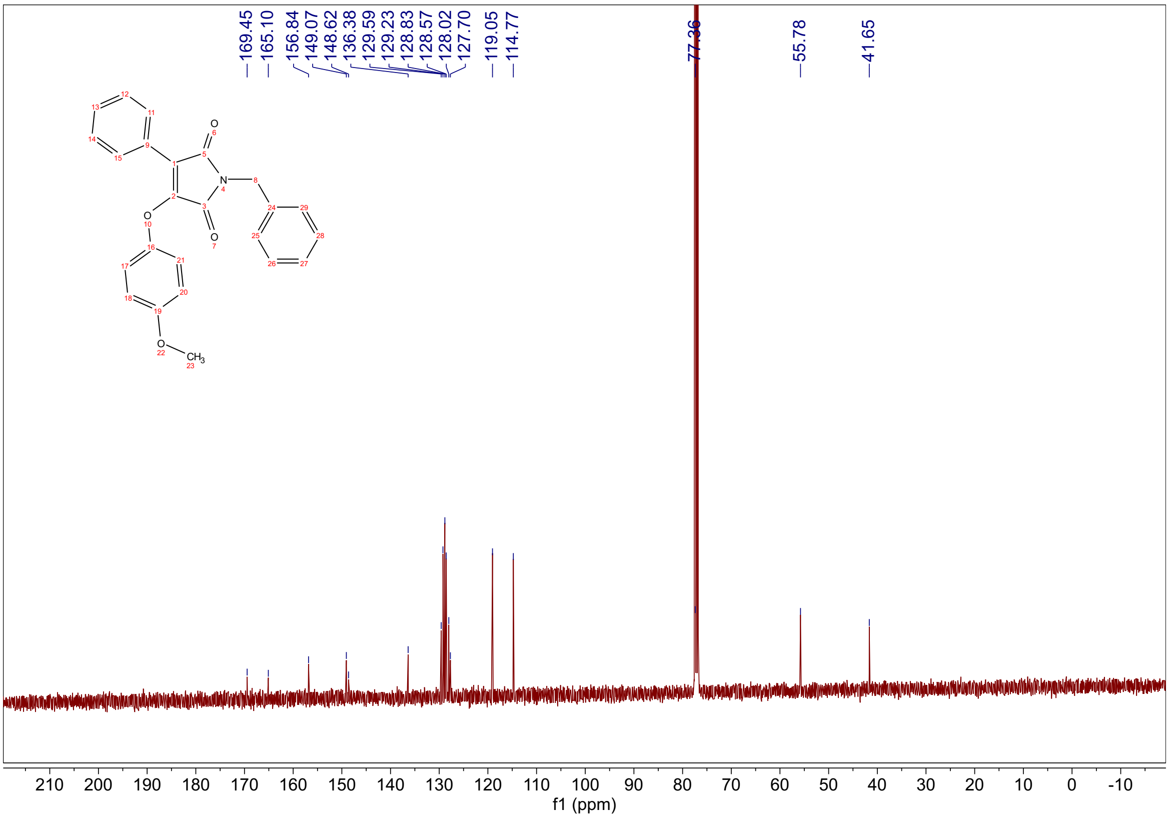
169.45
165.10
156.84
149.07
148.62
136.38
129.59
129.23
128.83
128.57
128.02
127.70
119.05
114.77
77.36
55.78
41.65
f1 (ppm)

Compound 27

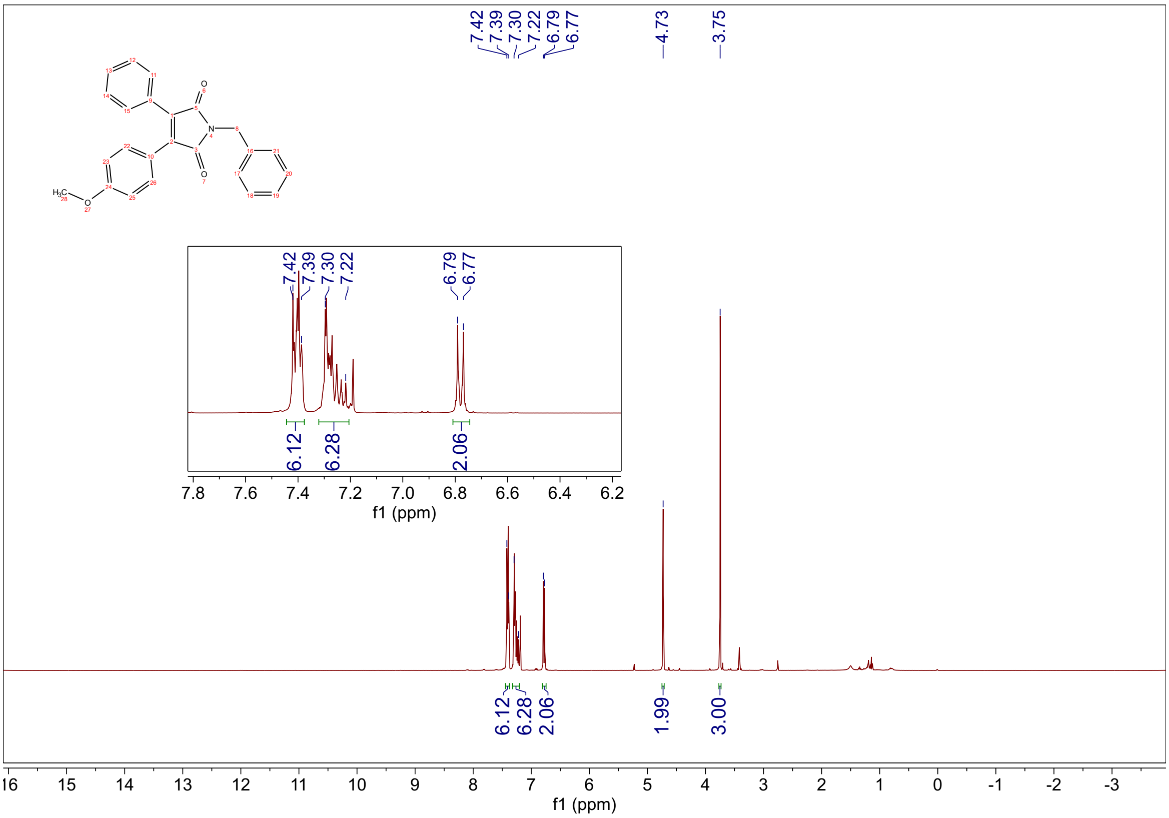

7.42
7.39
7.30
7.22
6.79
6.77
4.73
3.75
6.12
6.28
2.06
1.99
3.00
f1 (ppm)

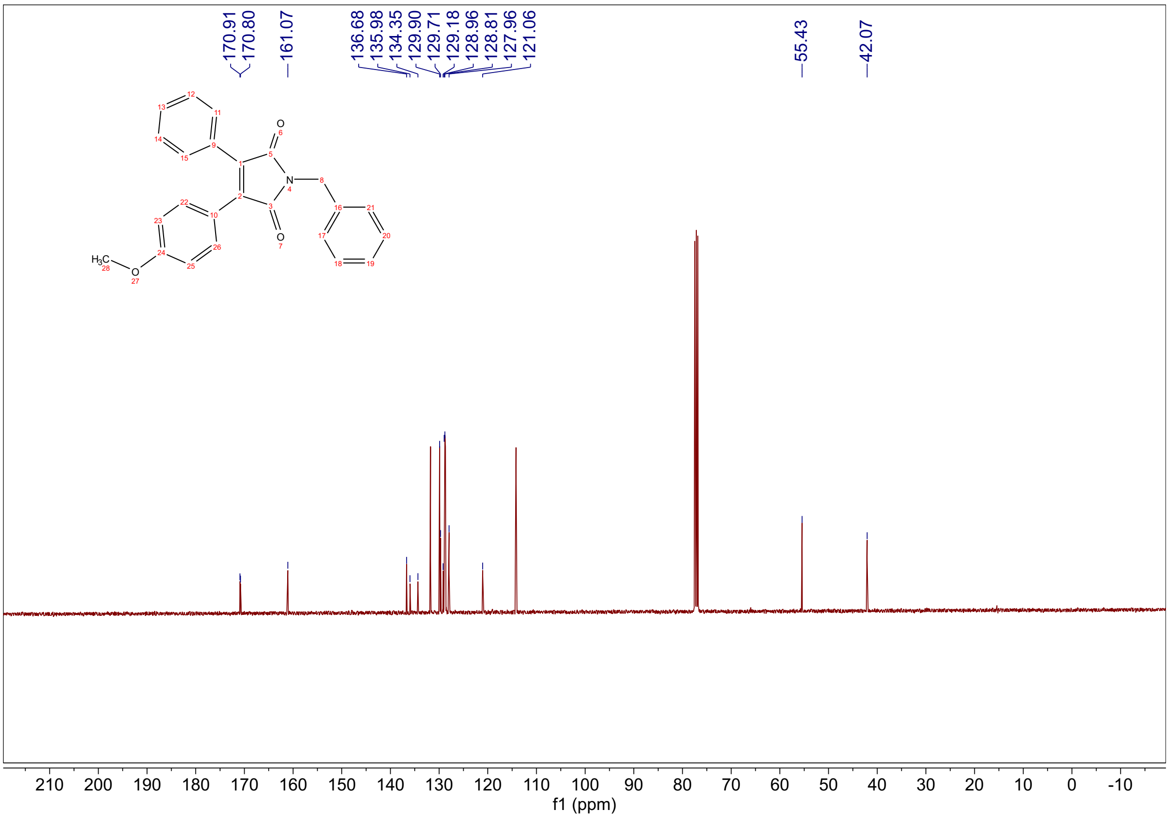

170.91
170.80
161.07
136.68
135.98
134.35
129.90
129.71
129.18
128.96
128.81
127.96
121.06
55.43
42.07
f1 (ppm)

Compound 28

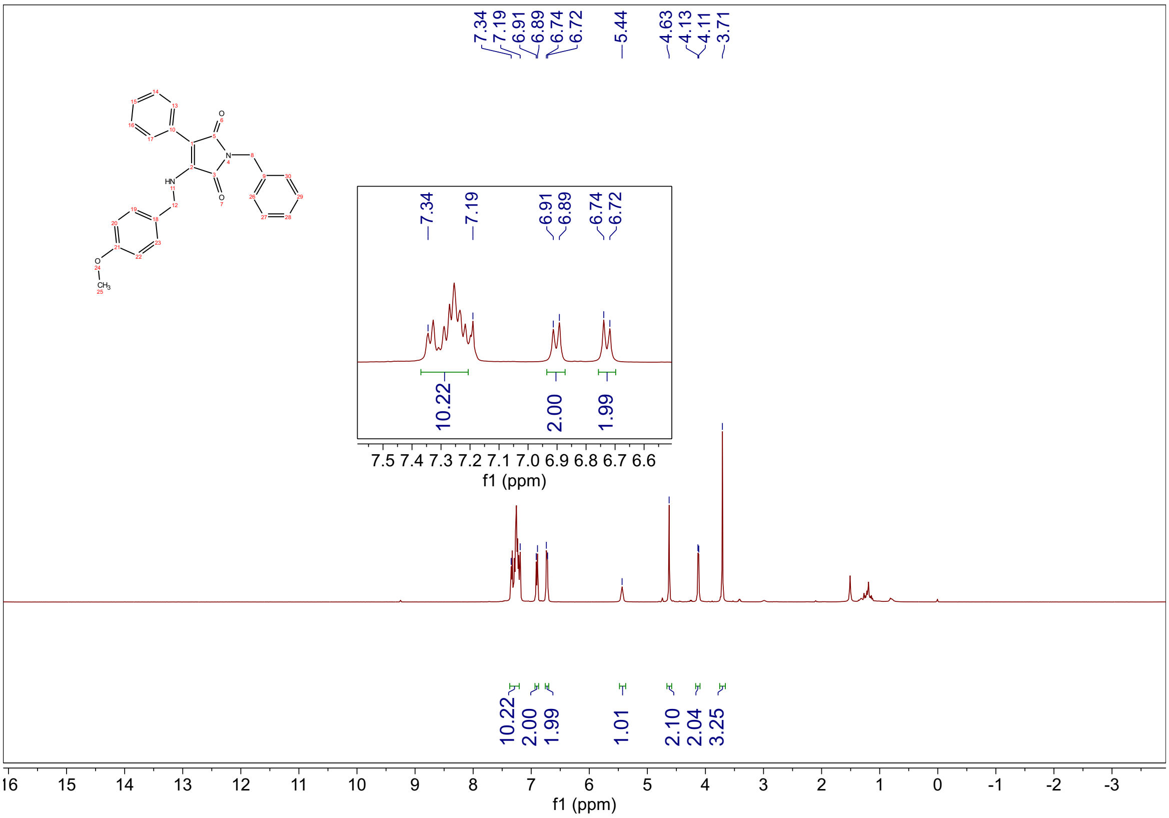
7.34
7.19
6.91
6.89
6.74
6.72
5.44
4.63
4.13
4.11
3.71
10.22
2.00
1.99
7.5 7.4 7.3 7.2 7.1 7.0 6.9 6.8 6.7 6.6
f1 (ppm)
10.22
2.00
1.99
1.01
2.10
2.04
3.25
16 15 14 13 12 11 10 9 8 7 6 5 4 3 2 1 0 -1 -2 -3
f1 (ppm)

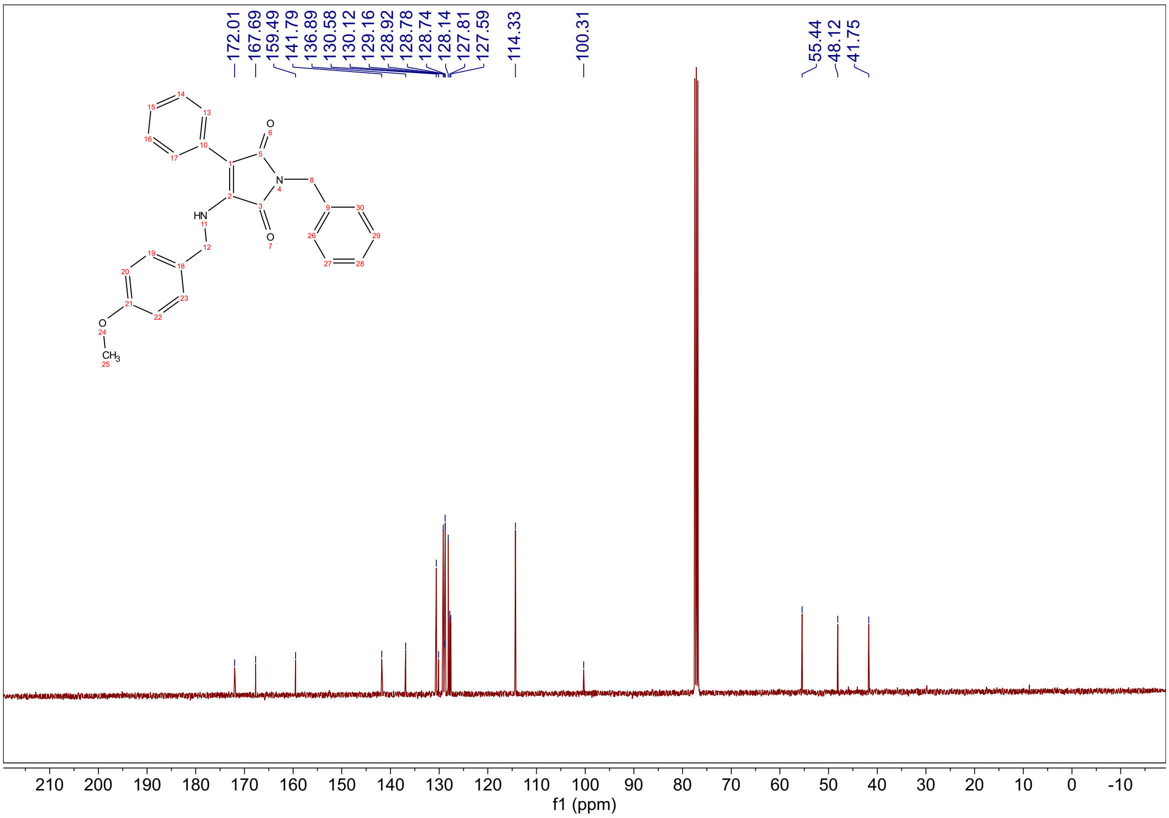
172.01
167.69
159.49
141.79
136.89
130.58
130.12
129.16
128.92
128.78
128.74
128.14
127.81
127.59
114.33
100.31
55.44
48.12
41.75
210 200 190 180 170 160 150 140 130 120 110 100 90 80 70 60 50 40 30 20 10 0 -10
f1 (ppm)

Compound 29

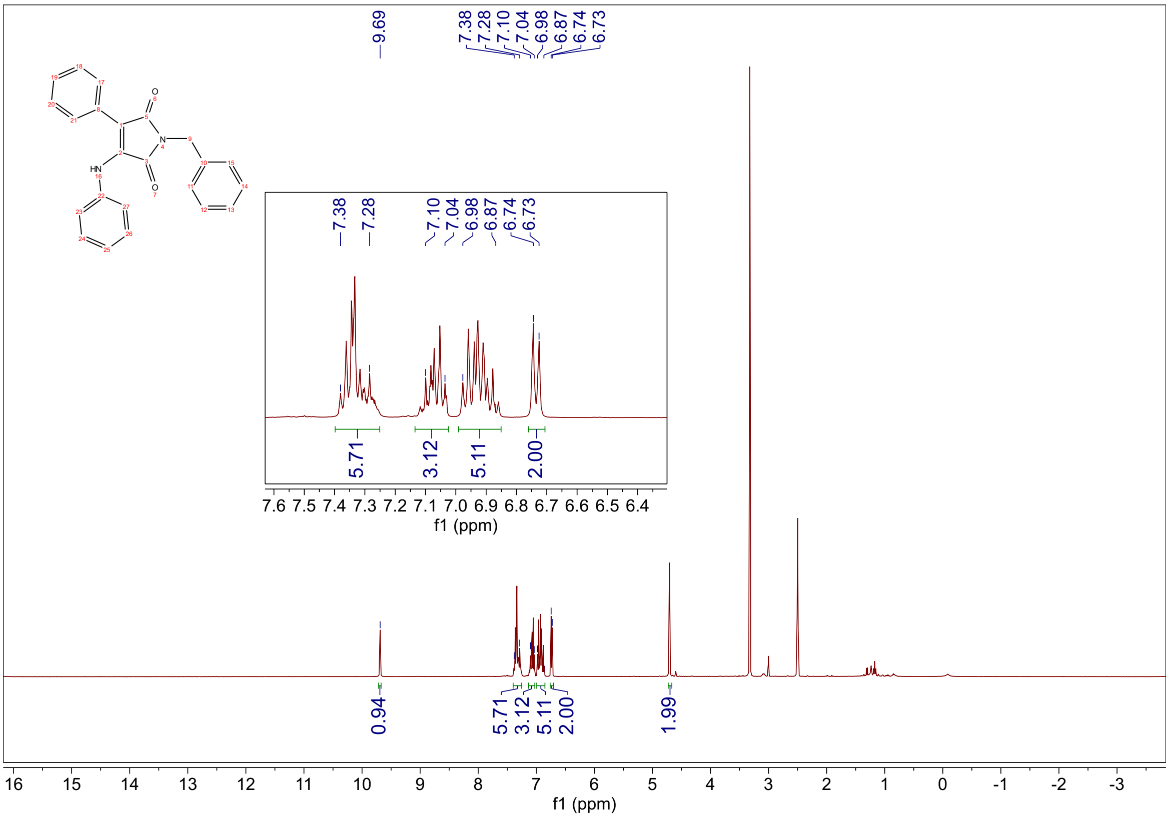
9.69
7.38
7.28
7.10
7.04
6.98
6.87
6.74
6.73
0.94
5.71
3.12
5.11
2.00
1.99
f1 (ppm)

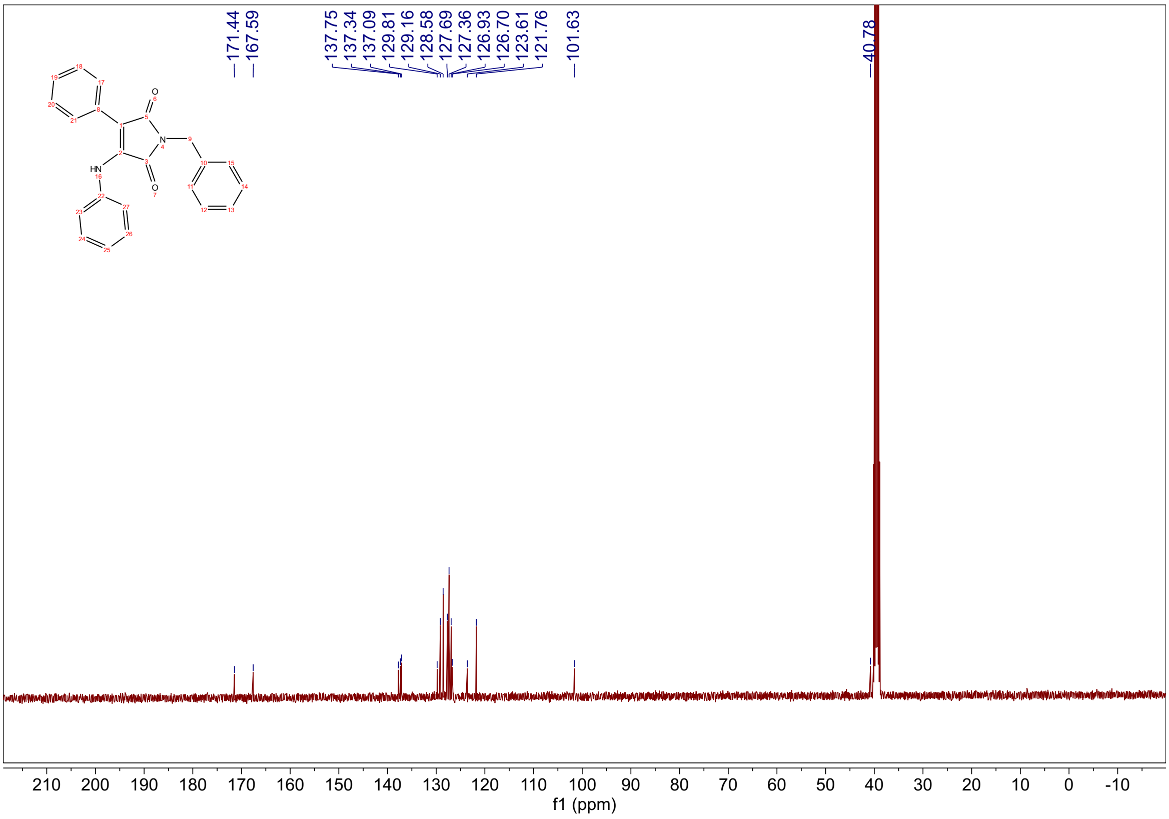
171.44
167.59
137.75
137.34
137.09
129.81
129.16
128.58
127.69
127.36
126.93
126.70
123.61
121.76
101.63
40.78
f1 (ppm)

Compound 30

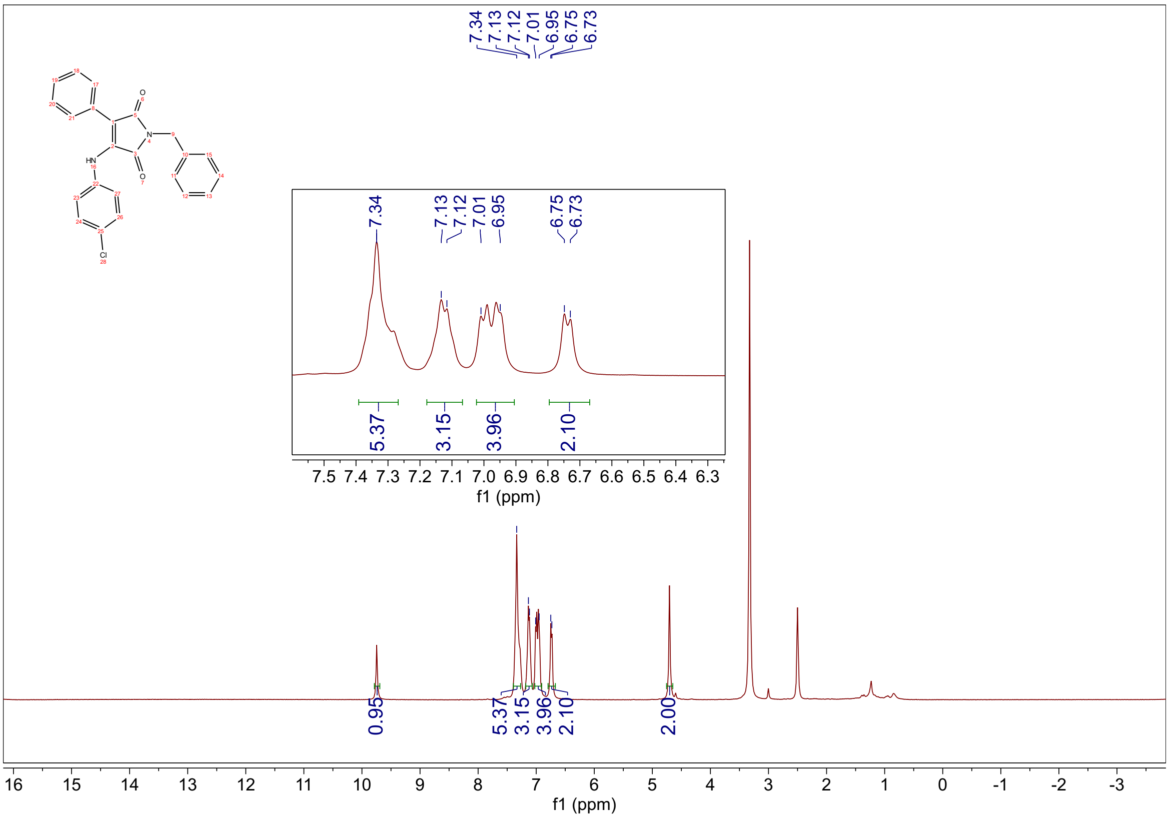
7.34
7.13
7.12
7.01
6.95
6.75
6.73
5.37
3.15
3.96
2.10
0.95
2.00
f1 (ppm)

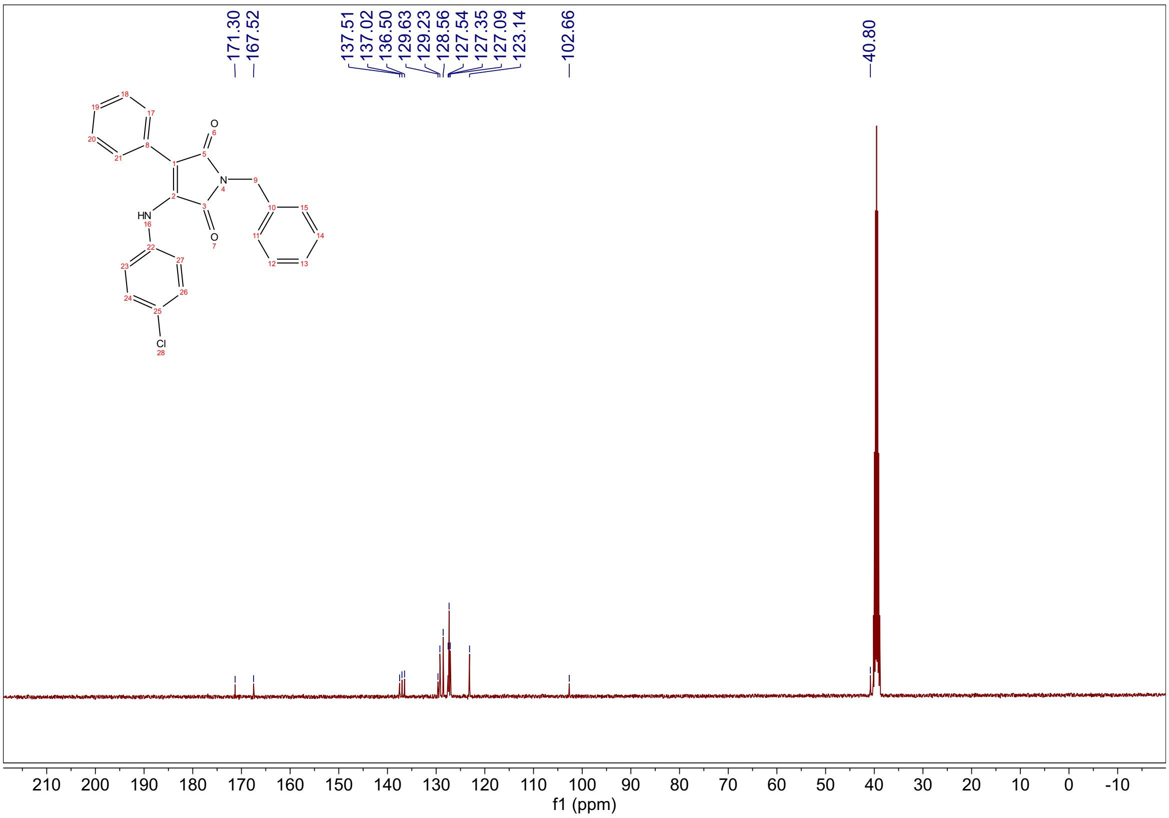
171.30
167.52
137.51
137.02
136.50
129.63
129.23
128.56
127.54
127.35
127.09
123.14
102.66
40.80
f1 (ppm)

Compound 31

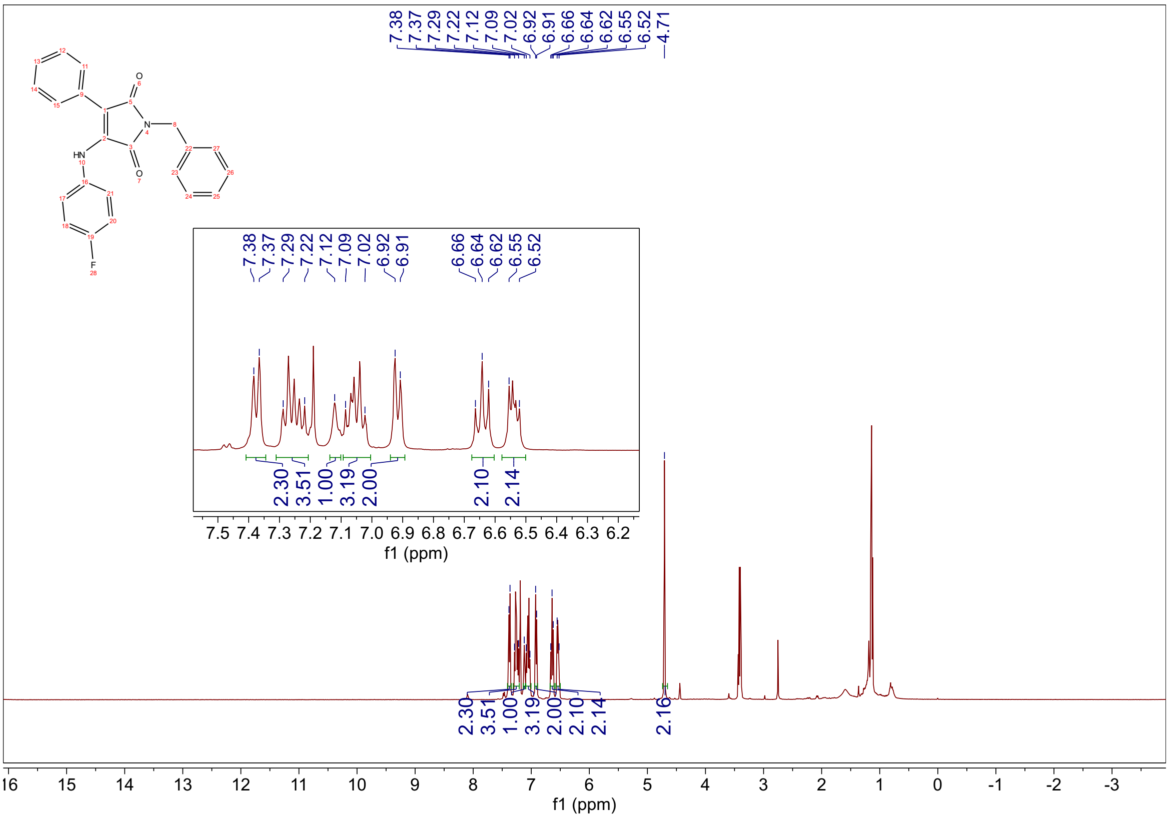
7.38
7.37
7.29
7.22
7.12
7.09
7.02
6.92
6.91
6.66
6.64
6.62
6.55
6.52
4.71
2.30
3.51
1.00
3.19
2.00
2.10
2.14
2.16
f1 (ppm)

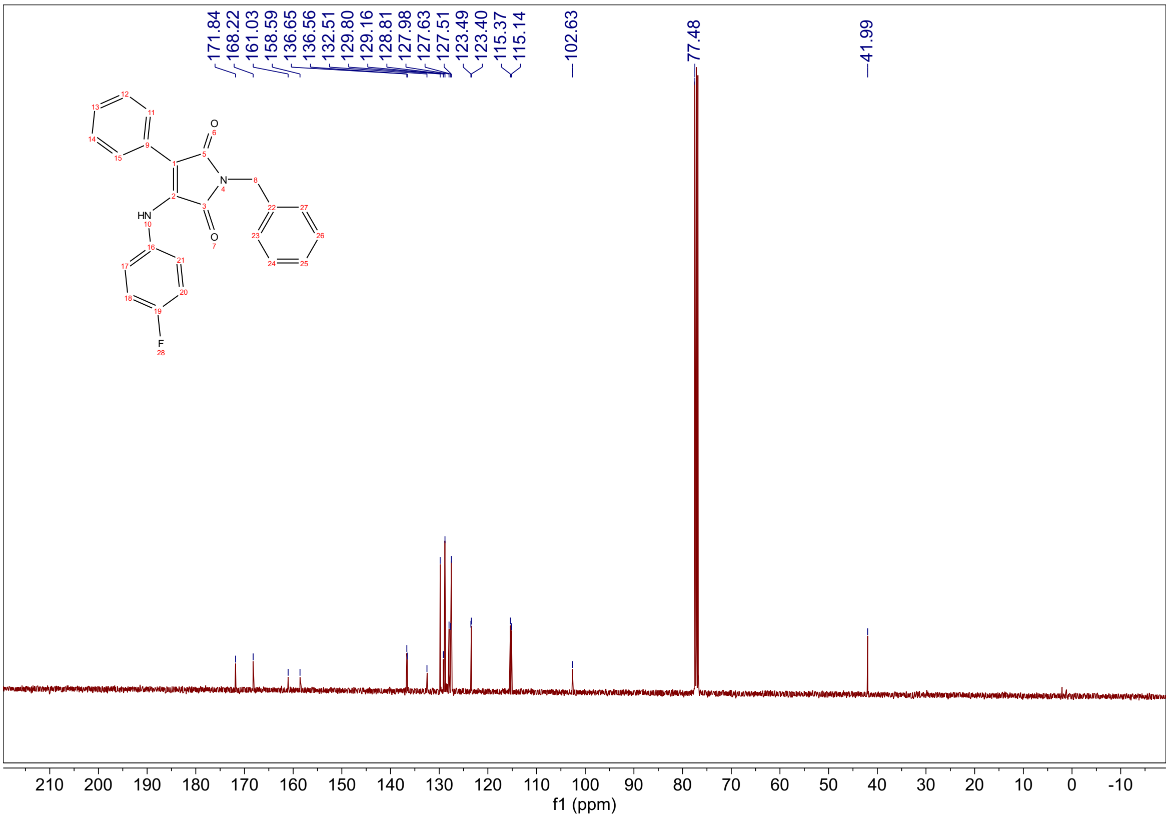
171.84
168.22
161.03
158.59
136.65
136.56
132.51
129.80
129.16
128.81
127.98
127.63
127.51
123.49
123.40
115.37
115.14
102.63
77.48
41.99
f1 (ppm)

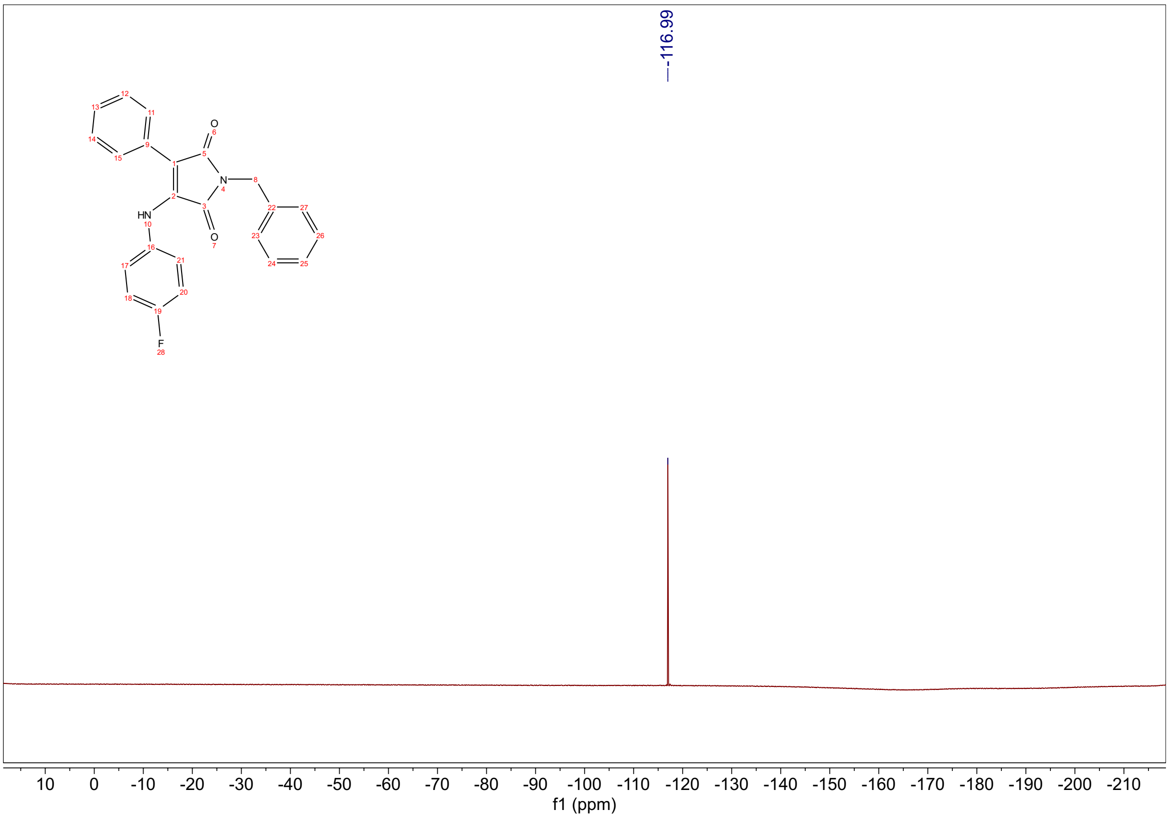
-116.99
HN
F
10 0 -10 -20 -30 -40 -50 -60 -70 -80 -90 -100 -110 -120 -130 -140 -150 -160 -170 -180 -190 -200 -210
f1 (ppm)

Compound 32

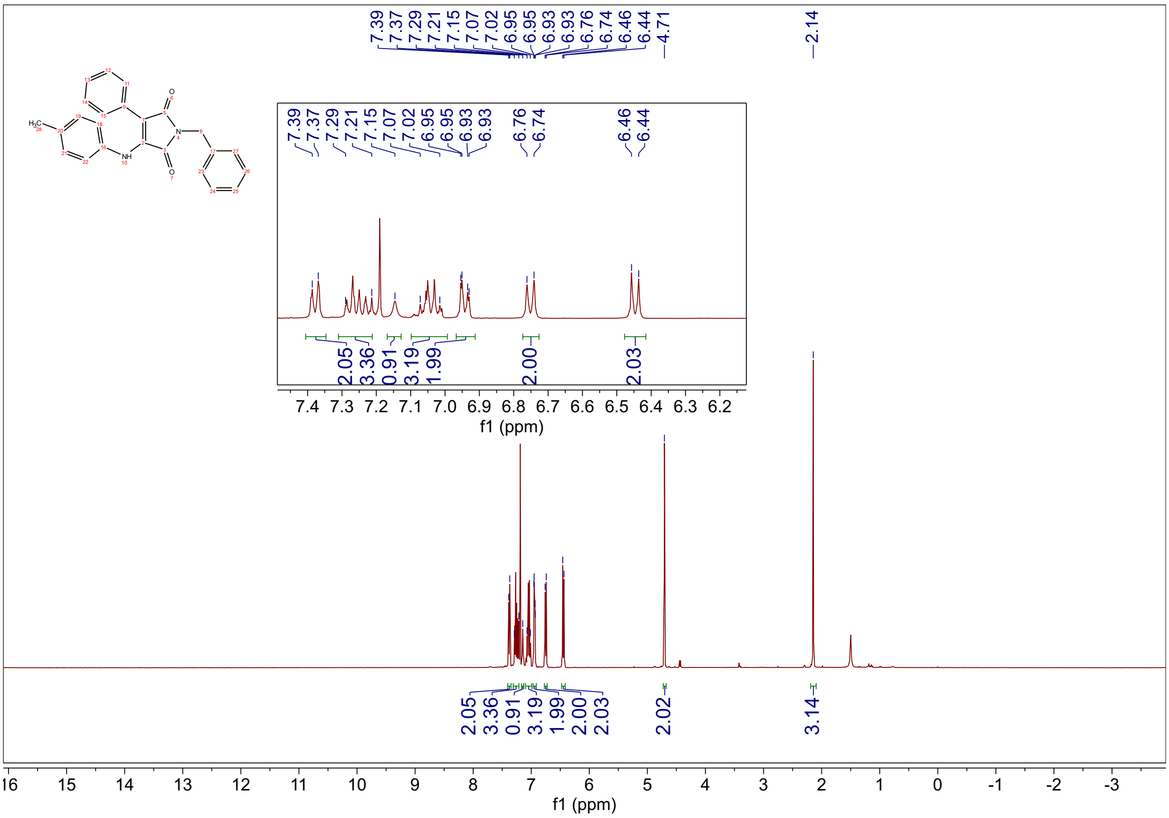

7.39 7.37 7.29 7.21 7.15 7.07 7.02 6.95 6.95 6.93 6.93 6.76 6.74 6.46 6.44 4.71 2.14
2.05 3.36 0.91 3.19 1.99 2.00 2.03 2.02 3.14
f1 (ppm)

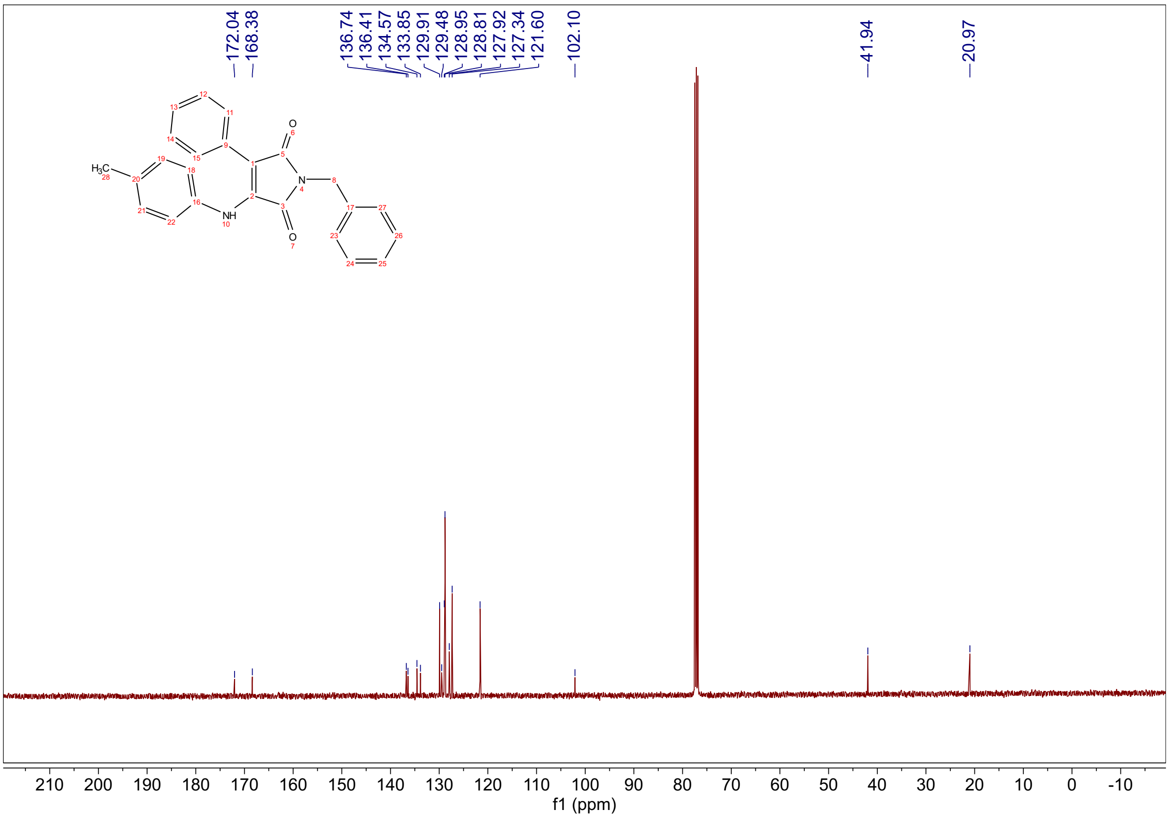

172.04 168.38 136.74 136.41 134.57 133.85 129.91 129.48 128.95 128.81 127.92 127.34 121.60 102.10 41.94 20.97
f1 (ppm)

Compound 33

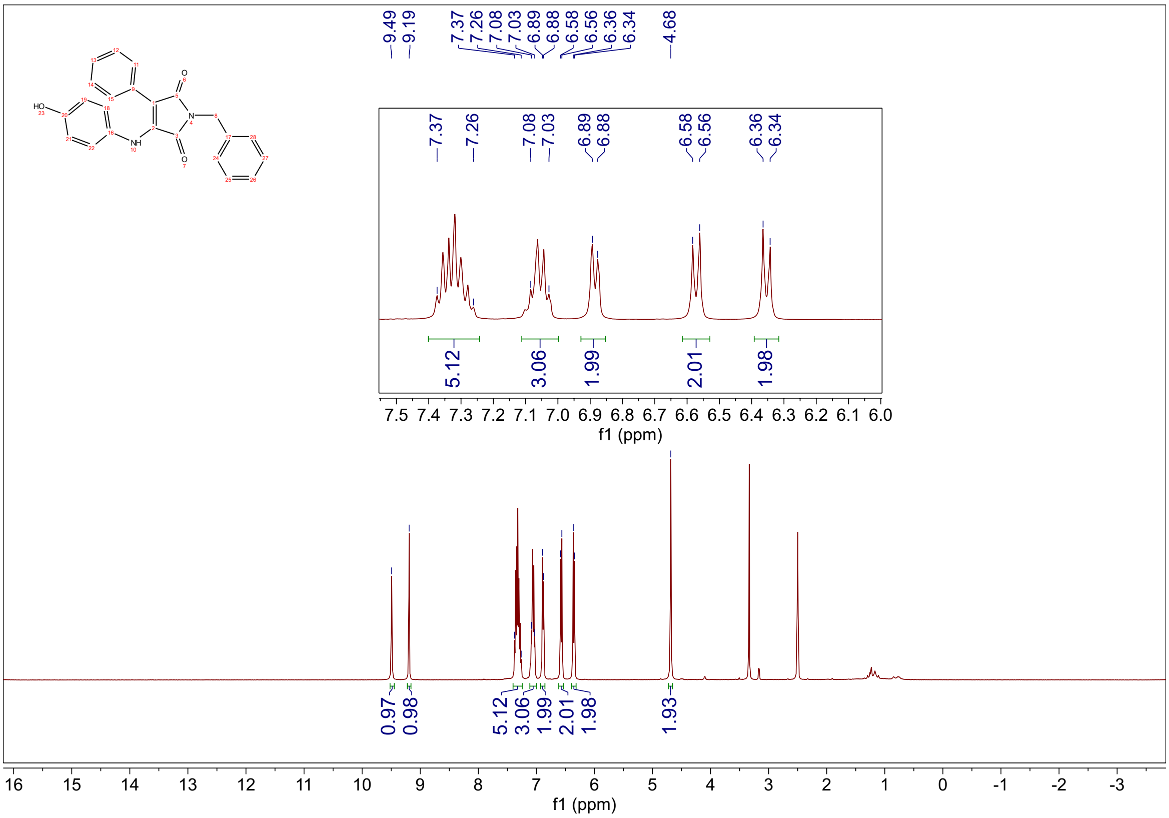
9.49
9.19
7.37
7.26
7.08
7.03
6.89
6.88
6.58
6.56
6.36
6.34
4.68
5.12
3.06
1.99
2.01
1.98
7.5 7.4 7.3 7.2 7.1 7.0 6.9 6.8 6.7 6.6 6.5 6.4 6.3 6.2 6.1 6.0
f1 (ppm)
0.97
0.98
1.93
16 15 14 13 12 11 10 9 8 7 6 5 4 3 2 1 0 -1 -2 -3
f1 (ppm)

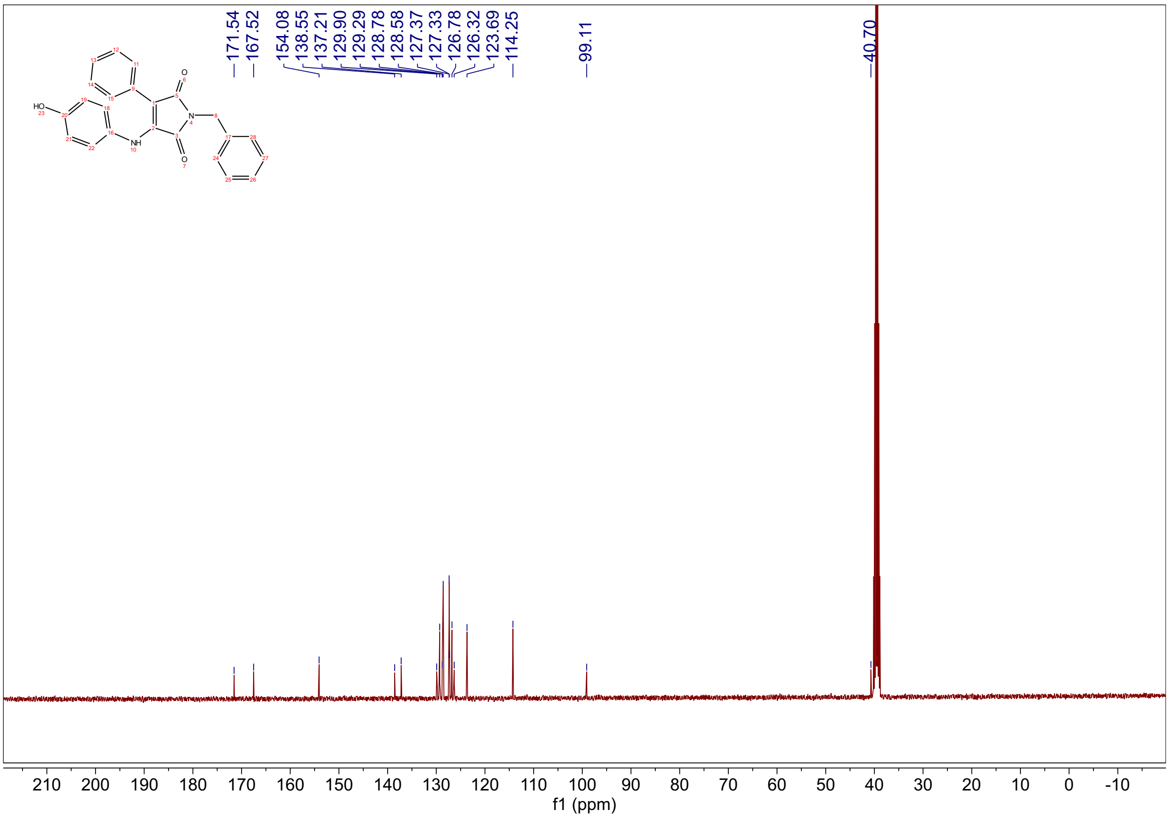
171.54
167.52
154.08
138.55
137.21
129.90
129.29
128.78
128.58
127.37
127.33
126.78
126.32
123.69
114.25
99.11
40.70
210 200 190 180 170 160 150 140 130 120 110 100 90 80 70 60 50 40 30 20 10 0 -10
f1 (ppm)

Compound 34

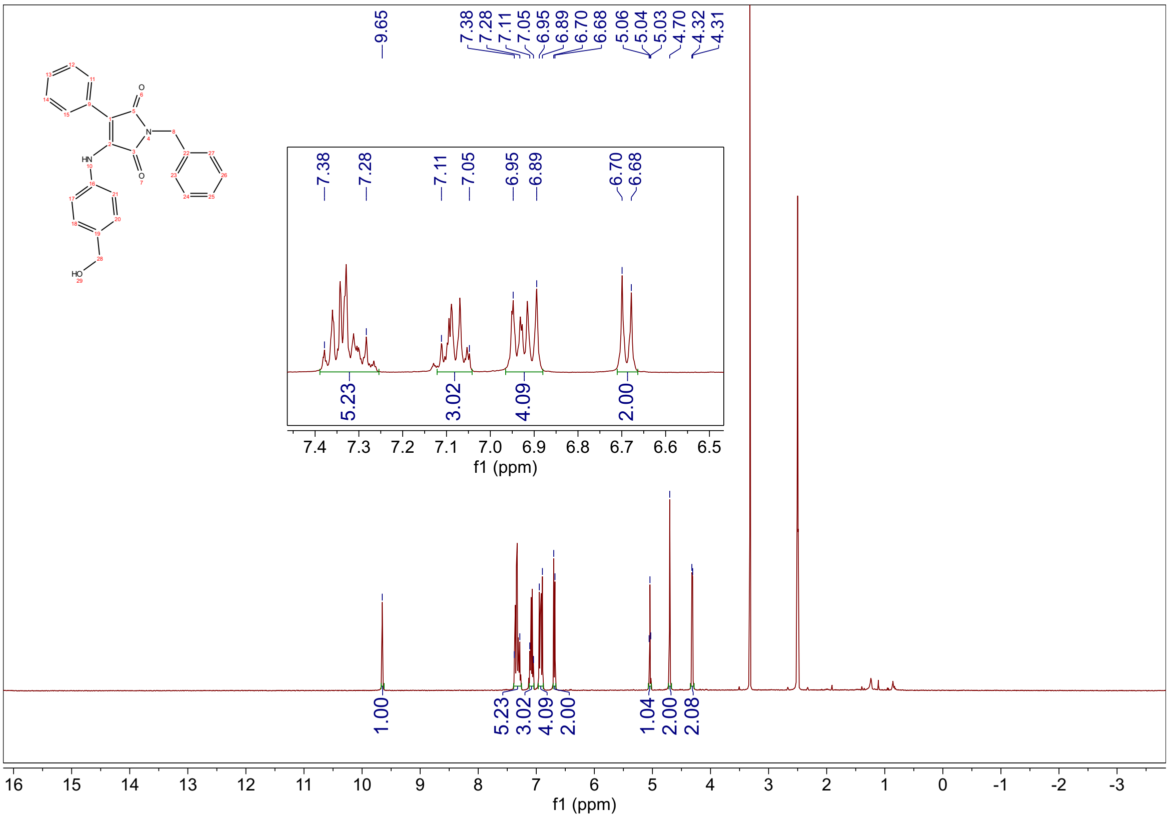

9.65
7.38 7.28 7.11 7.05 6.95 6.89 6.70 6.68 5.06 5.04 5.03 4.70 4.32 4.31
1.00
5.23 3.02 4.09 2.00
1.04 2.00 2.08
f1 (ppm)

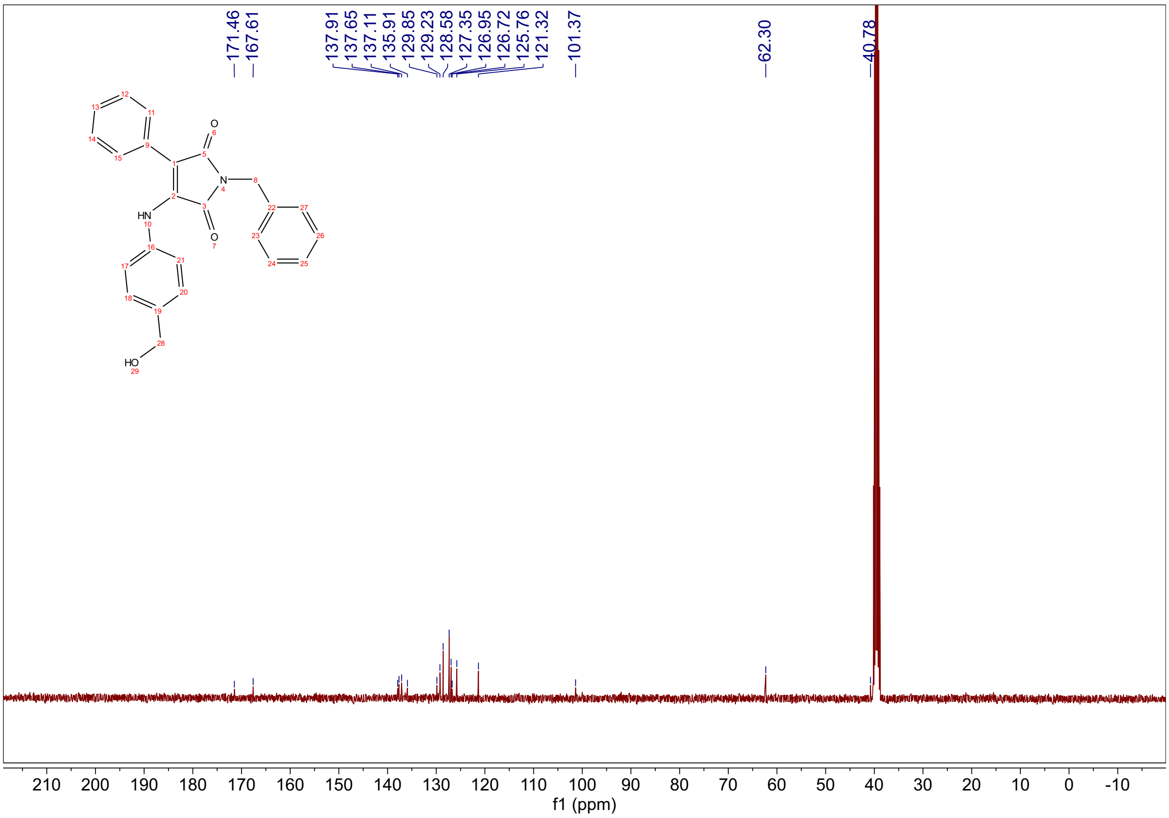

171.46 167.61
137.91 137.65 137.11 135.91 129.85 129.23 128.58 127.35 126.95 126.72 125.76 121.32
101.37
62.30
40.78
f1 (ppm)

Compound 35

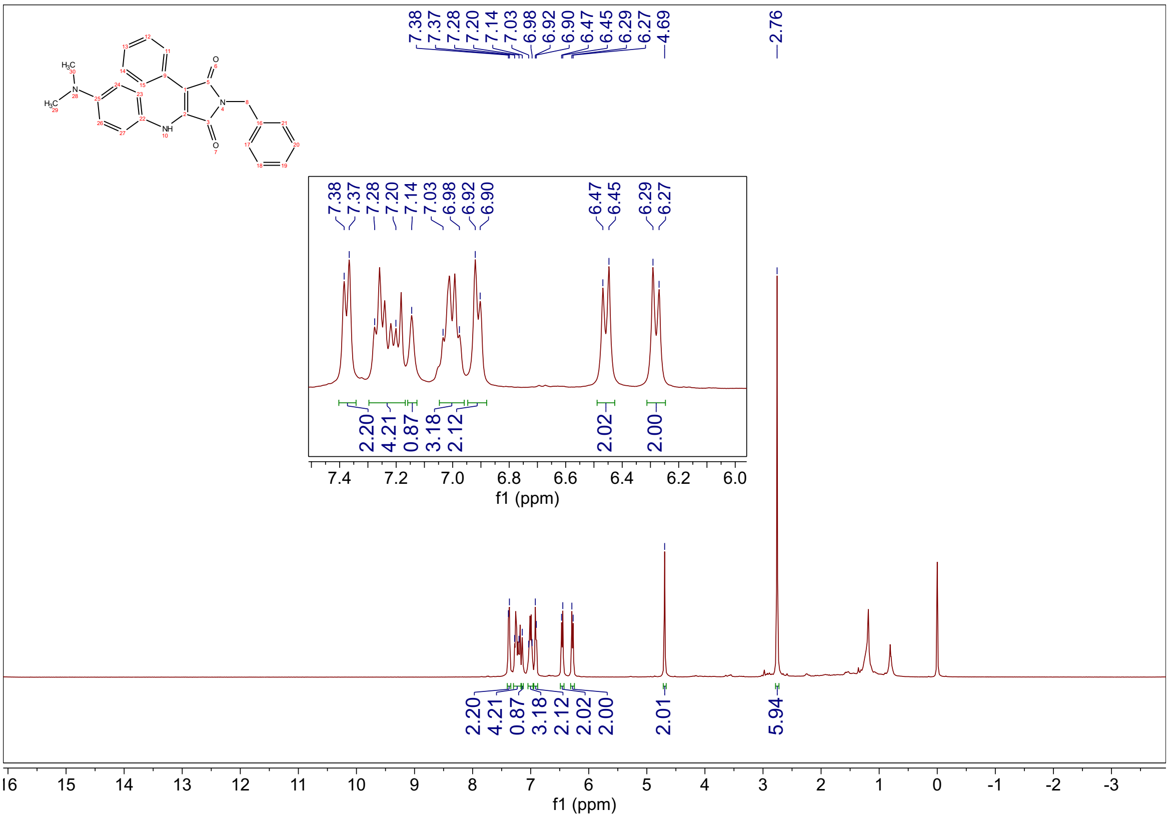

7.38
7.37
7.28
7.20
7.14
7.03
6.98
6.92
6.90
6.47
6.45
6.29
6.27
4.69
2.76
2.20
4.21
0.87
3.18
2.12
2.02
2.00
2.01
5.94
f1 (ppm)

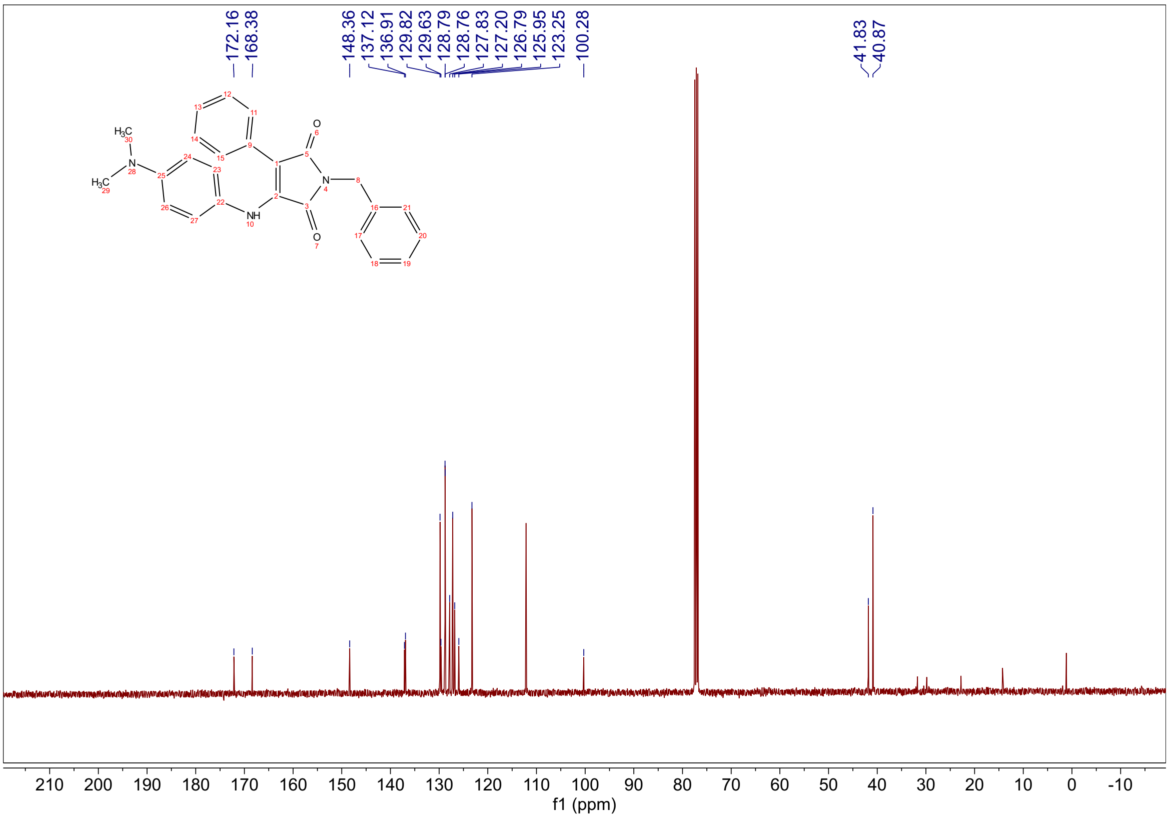

172.16
168.38
148.36
137.12
136.91
129.82
129.63
128.79
128.76
127.83
127.20
126.79
125.95
123.25
100.28
41.83
40.87
f1 (ppm)

Compound 36

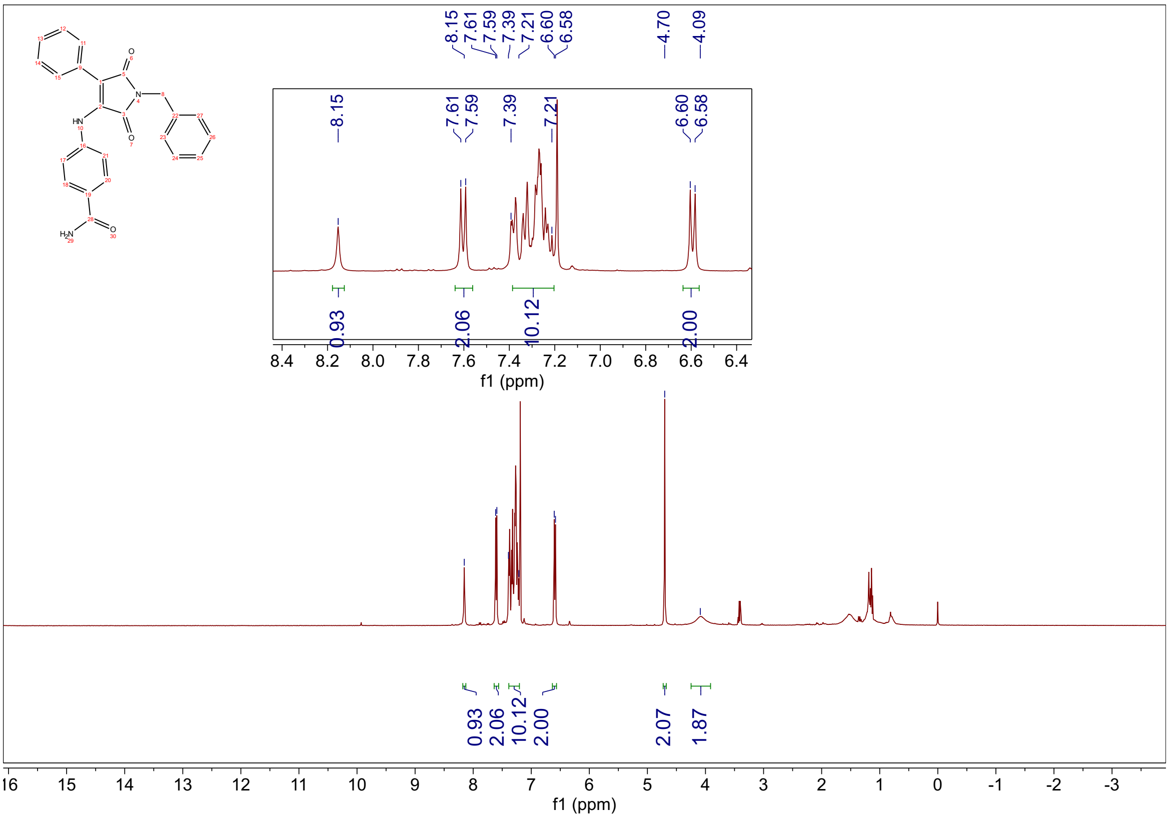
8.15
7.61
7.59
7.39
7.21
6.60
6.58
4.70
4.09
0.93
2.06
10.12
2.00
2.07
1.87
f1 (ppm)

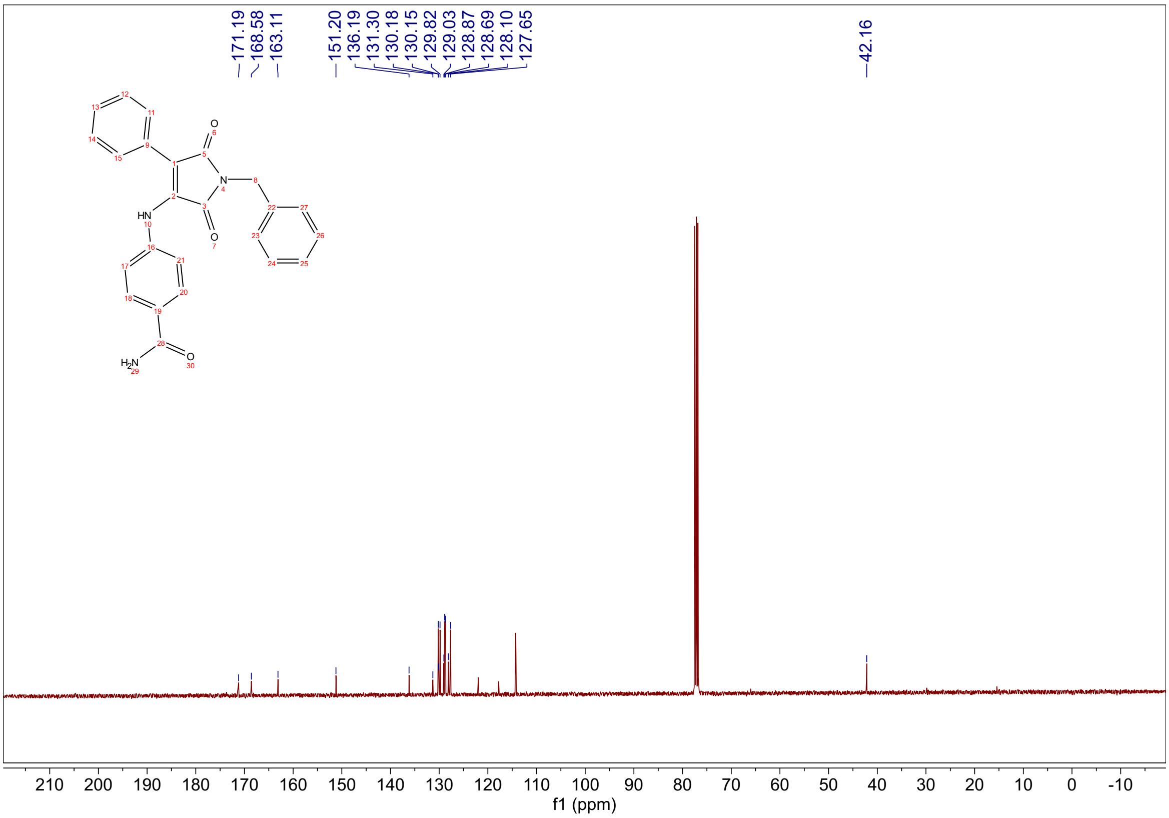
171.19
168.58
163.11
151.20
136.19
131.30
130.18
130.15
129.82
129.03
128.87
128.69
128.10
127.65
42.16
f1 (ppm)

Compound 37

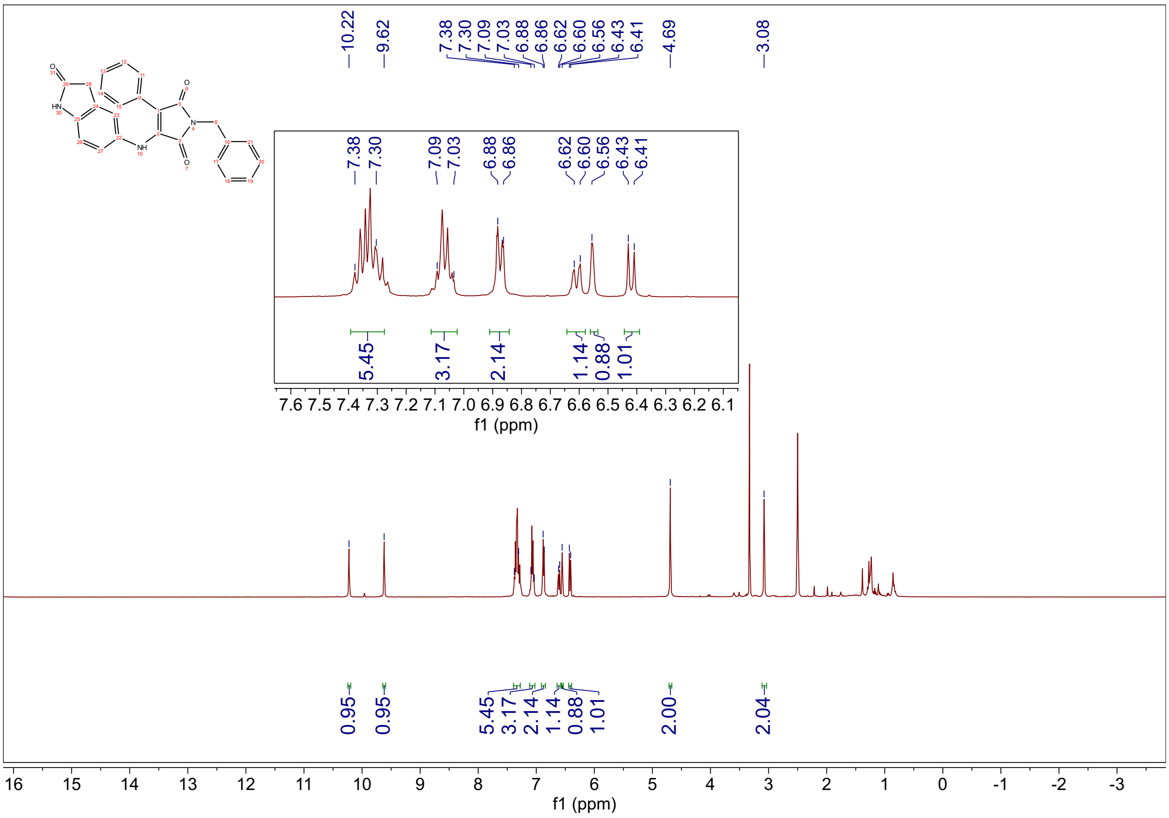

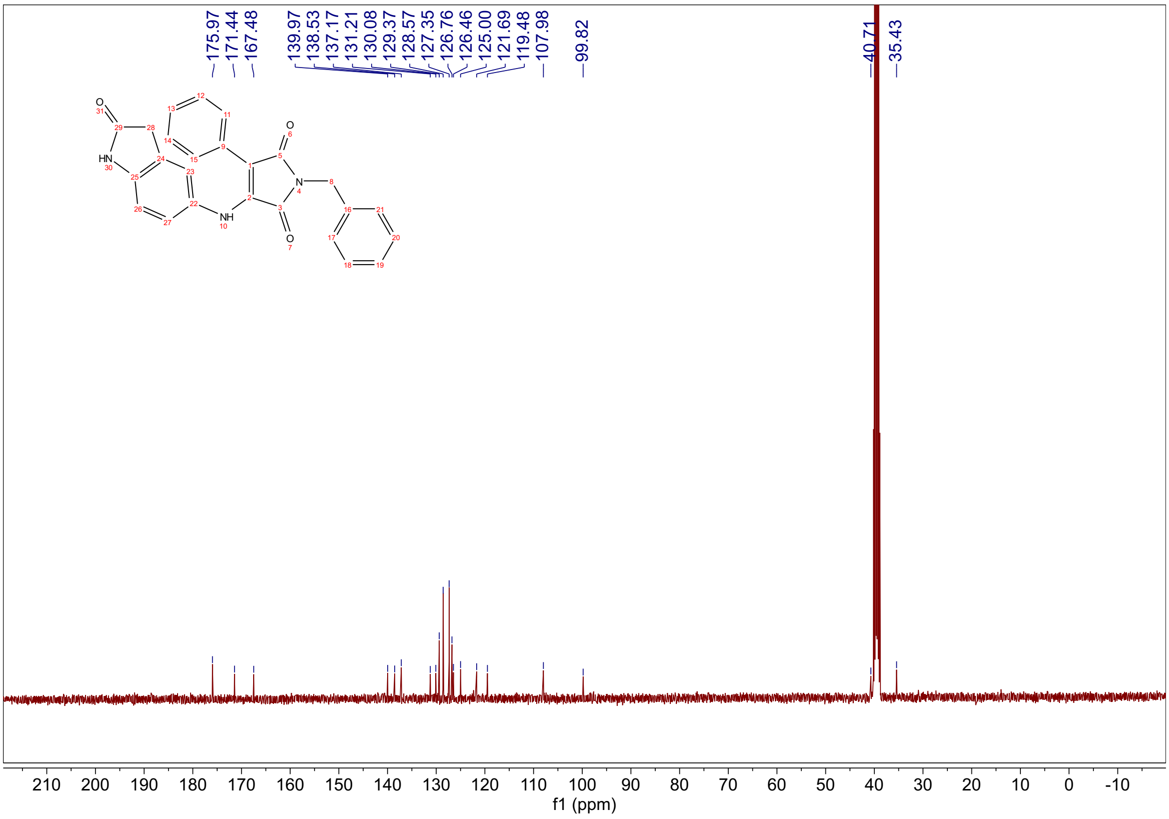

Compound 38

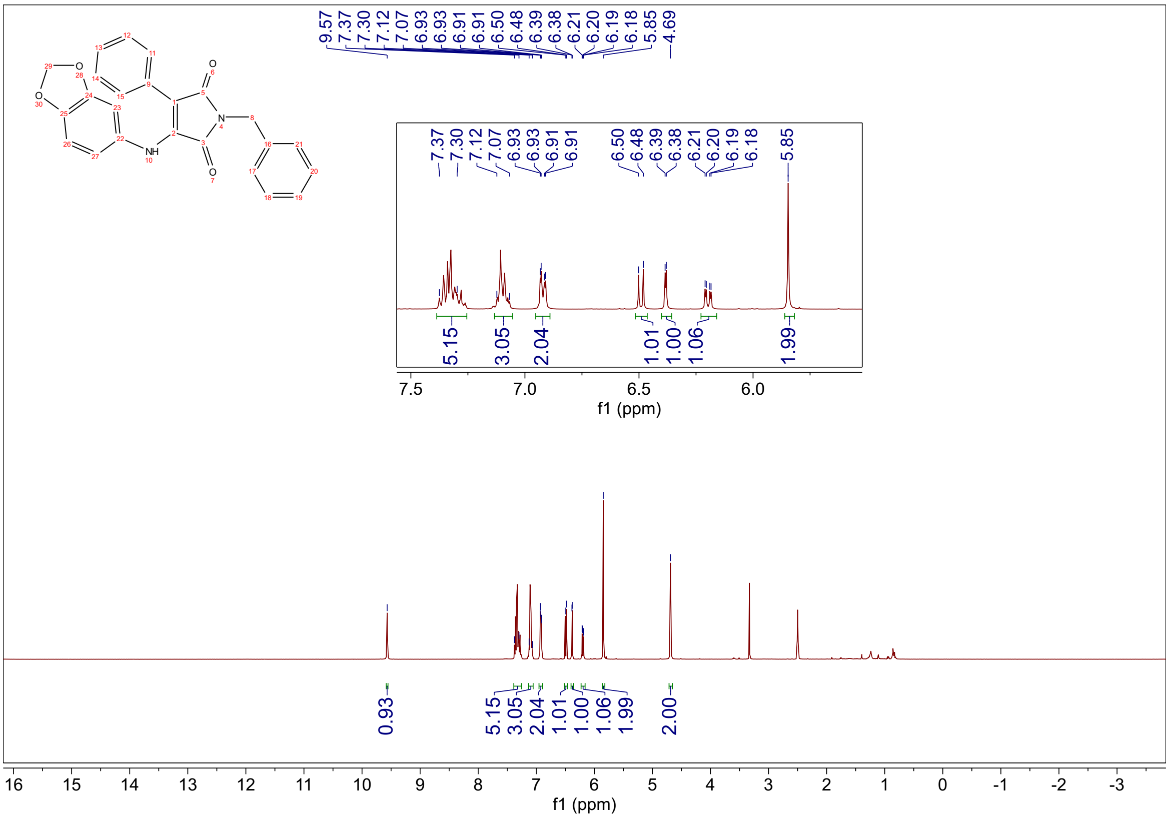

9.57
7.37
7.30
7.12
7.07
6.93
6.93
6.91
6.91
6.50
6.48
6.39
6.38
6.21
6.20
6.19
6.18
5.85
4.69
0.93
5.15
3.05
2.04
1.01
1.00
1.06
1.99
2.00
f1 (ppm)

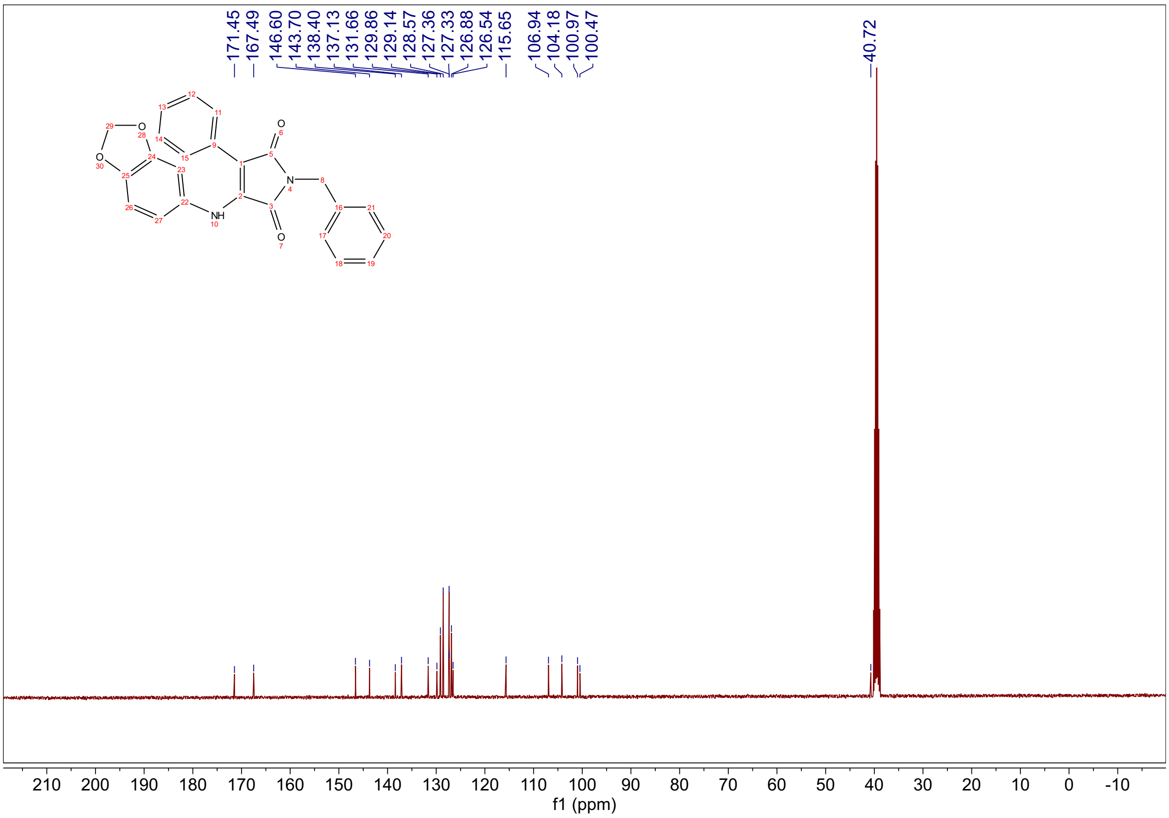

171.45
167.49
146.60
143.70
138.40
137.13
131.66
129.86
129.14
128.57
127.36
127.33
126.88
126.54
115.65
106.94
104.18
100.97
100.47
40.72
f1 (ppm)

Compound 39

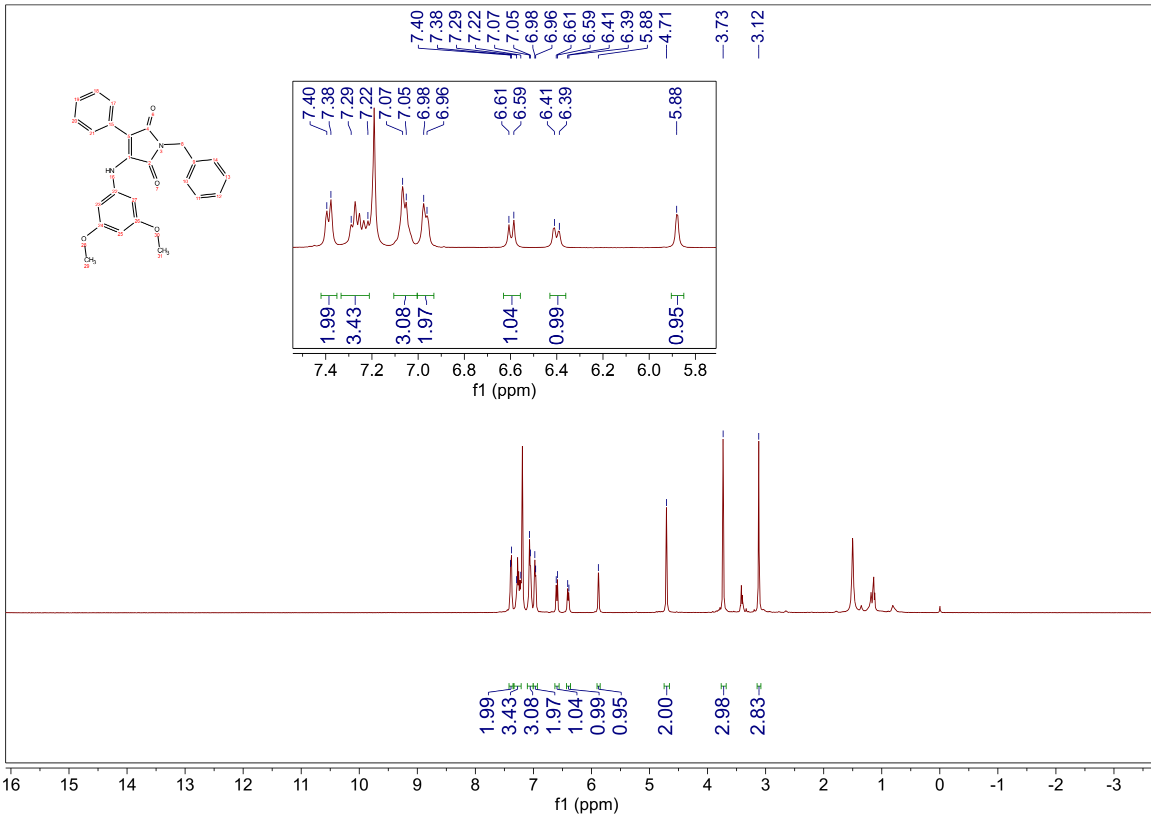
7.40
7.38
7.29
7.22
7.07
7.05
6.98
6.96
6.61
6.59
6.41
6.39
5.88
4.71
3.73
3.12
1.99
3.43
3.08
1.97
1.04
0.99
0.95
2.00
2.98
2.83
f1 (ppm)

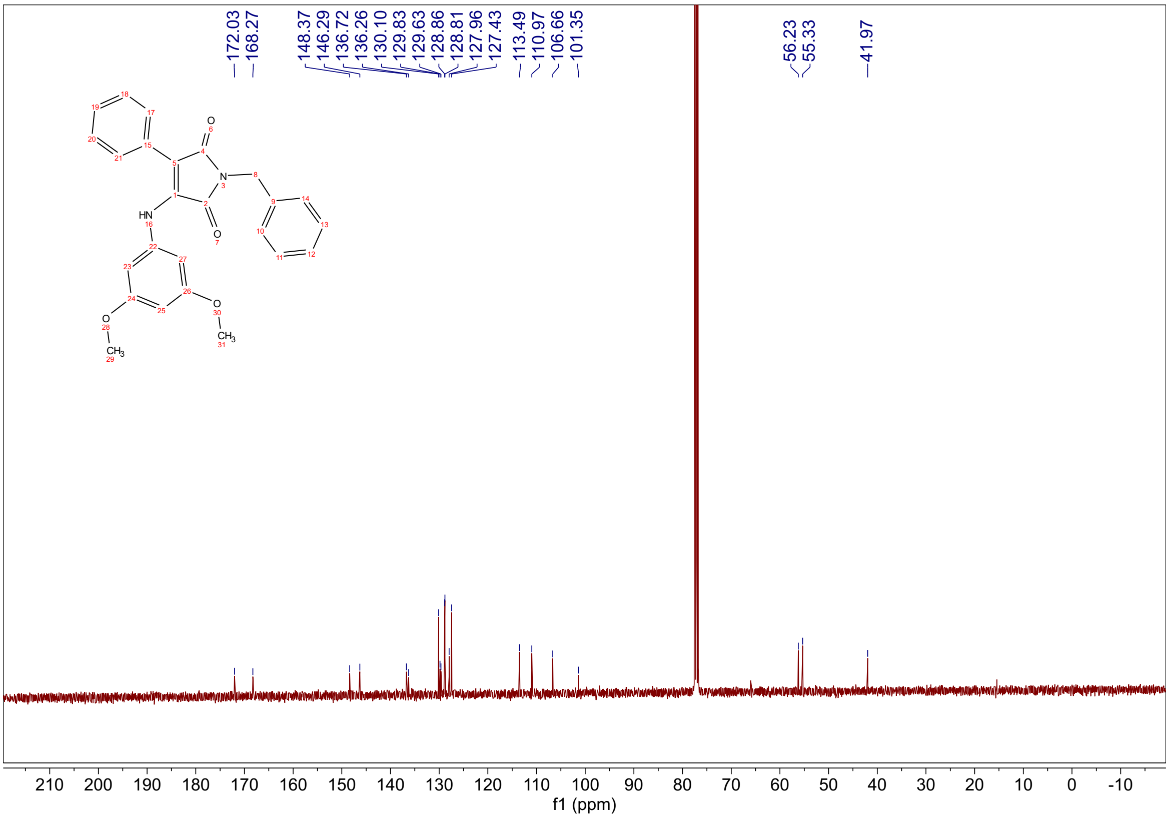
172.03
168.27
148.37
146.29
136.72
136.26
130.10
129.83
129.63
128.86
128.81
127.96
127.43
113.49
110.97
106.66
101.35
56.23
55.33
41.97
f1 (ppm)

Compound 40

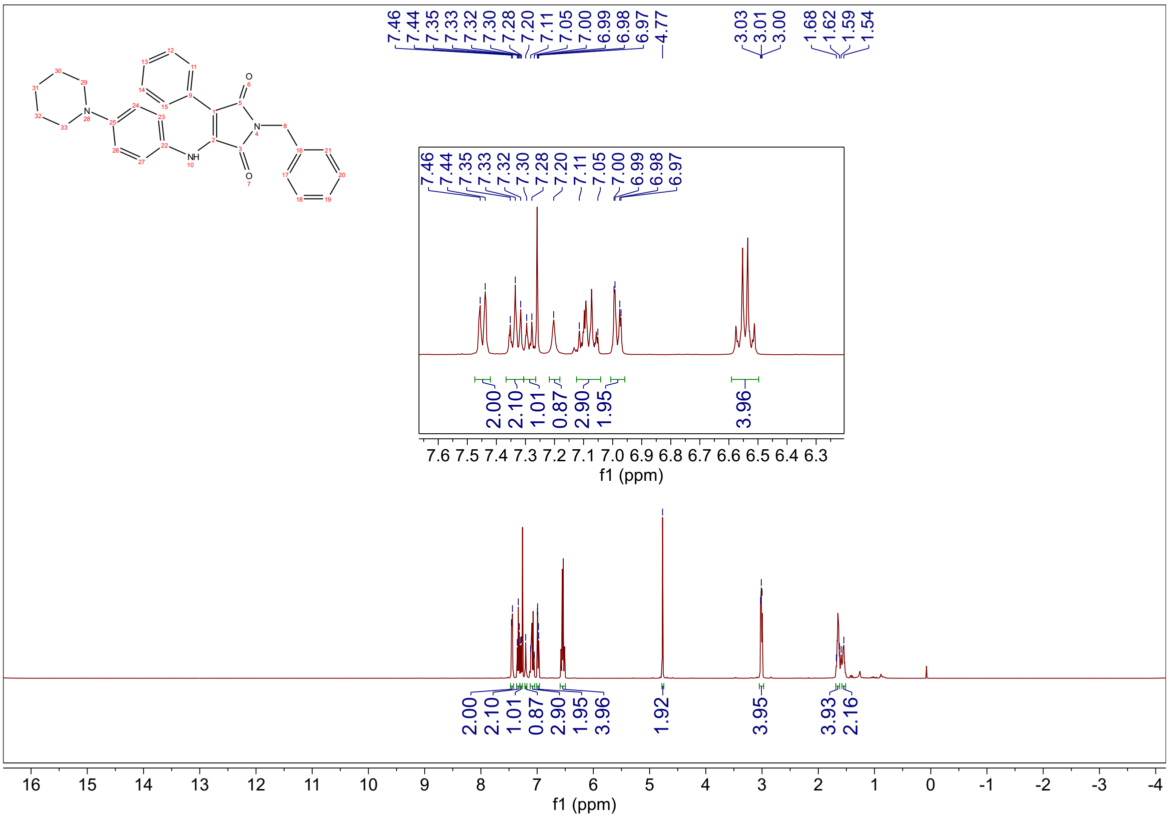
7.46
7.44
7.35
7.33
7.32
7.30
7.28
7.20
7.11
7.05
7.00
6.99
6.98
6.97
4.77
3.03
3.01
3.00
1.68
1.62
1.59
1.54
2.00
2.10
1.01
0.87
2.90
1.95
3.96
1.92
3.95
3.93
2.16
f1 (ppm)

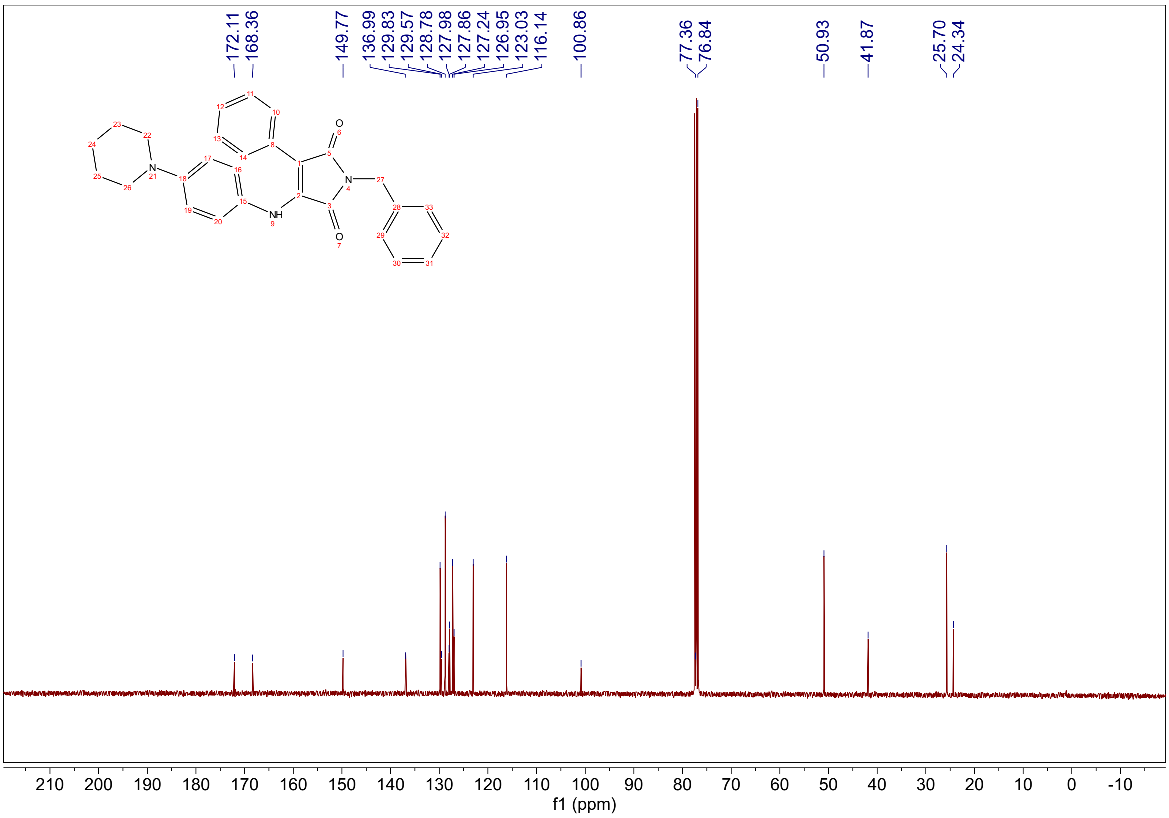
172.11
168.36
149.77
136.99
129.83
129.57
128.78
127.98
127.86
127.24
126.95
123.03
116.14
100.86
77.36
76.84
50.93
41.87
25.70
24.34
f1 (ppm)

Compound 41

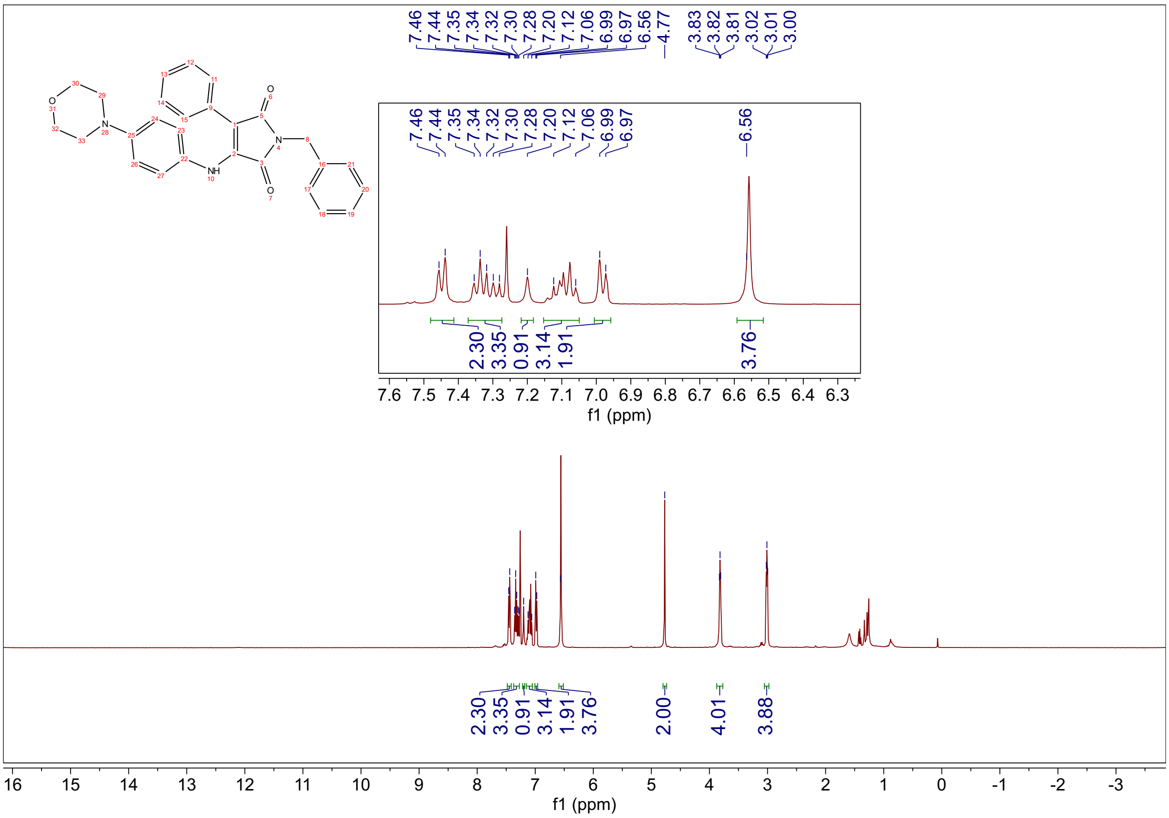
7.46
7.44
7.35
7.34
7.32
7.30
7.28
7.20
7.12
7.06
6.99
6.97
6.56
4.77
3.83
3.82
3.81
3.02
3.01
3.00
2.30
3.35
0.91
3.14
1.91
3.76
2.00
4.01
3.88
f1 (ppm)

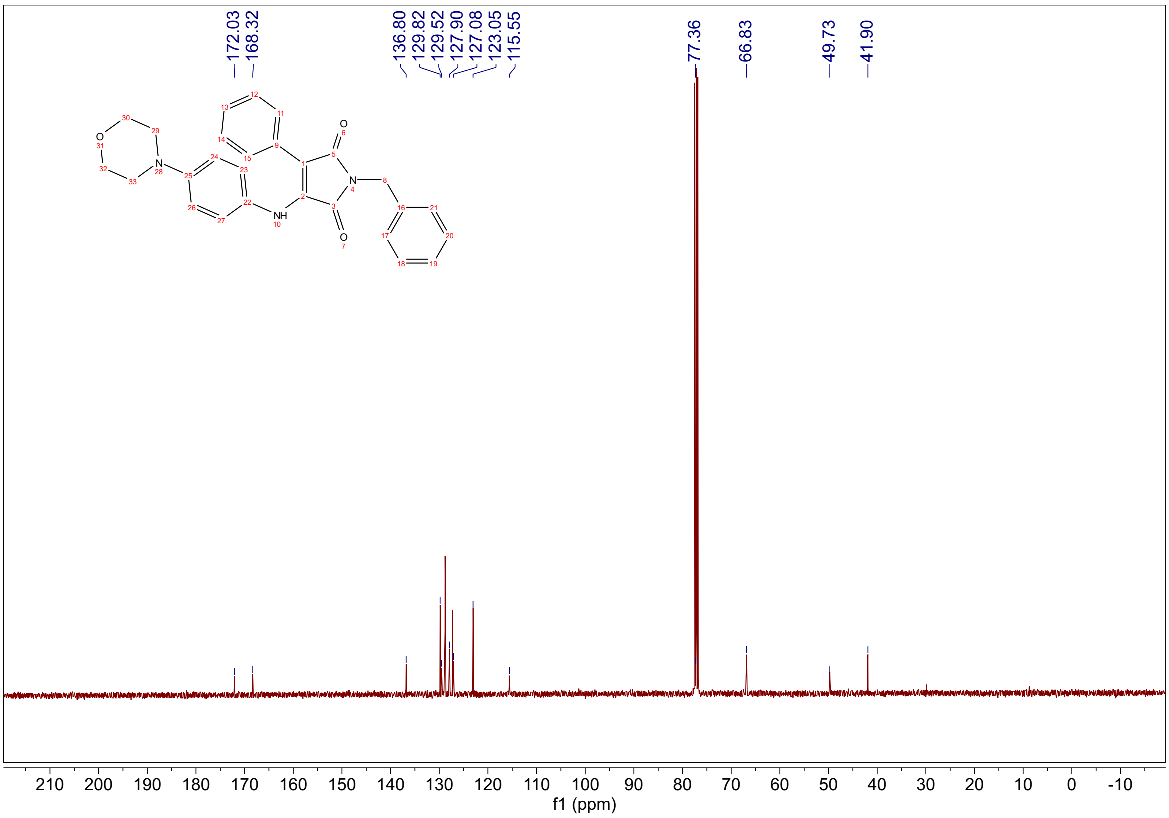
172.03
168.32
136.80
129.82
129.52
127.90
127.08
123.05
115.55
77.36
66.83
49.73
41.90
f1 (ppm)

Compound 42

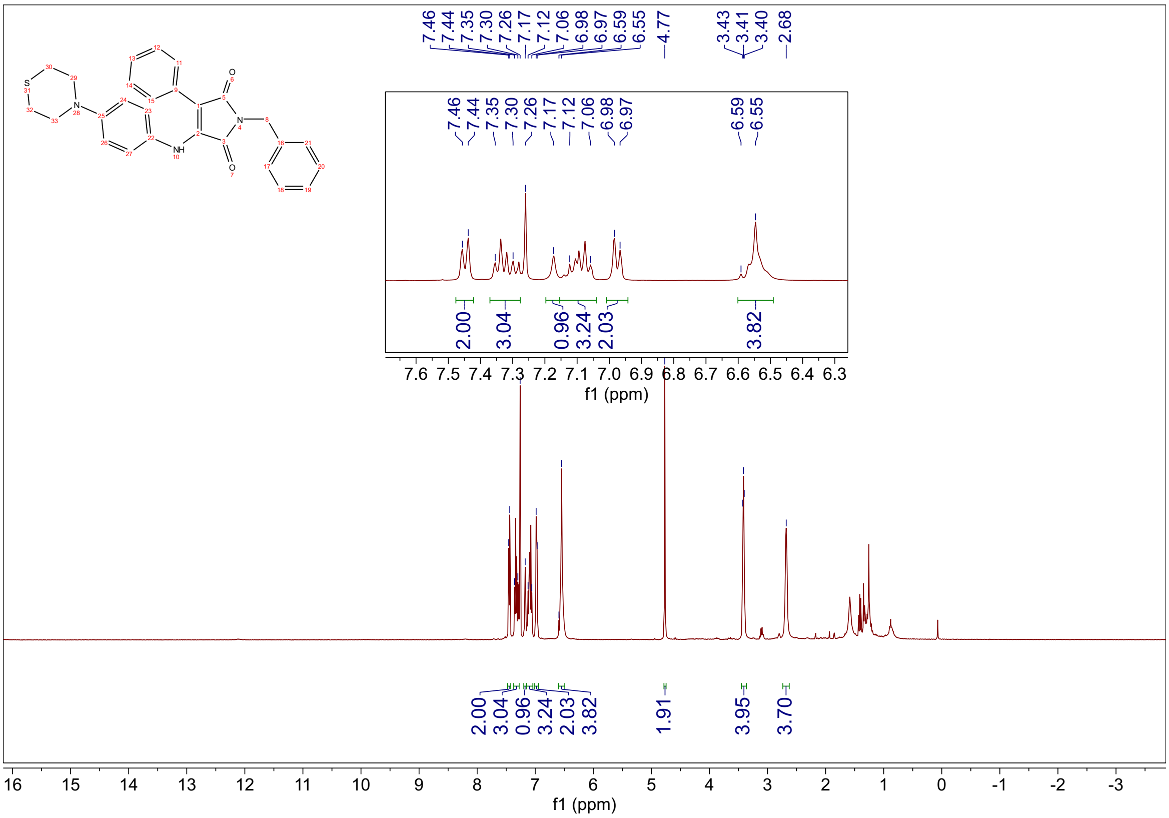
7.46
7.44
7.35
7.30
7.26
7.17
7.12
7.06
6.98
6.97
6.59
6.55
4.77
3.43
3.41
3.40
2.68
2.00
3.04
0.96
3.24
2.03
3.82
1.91
3.95
3.70
f1 (ppm)

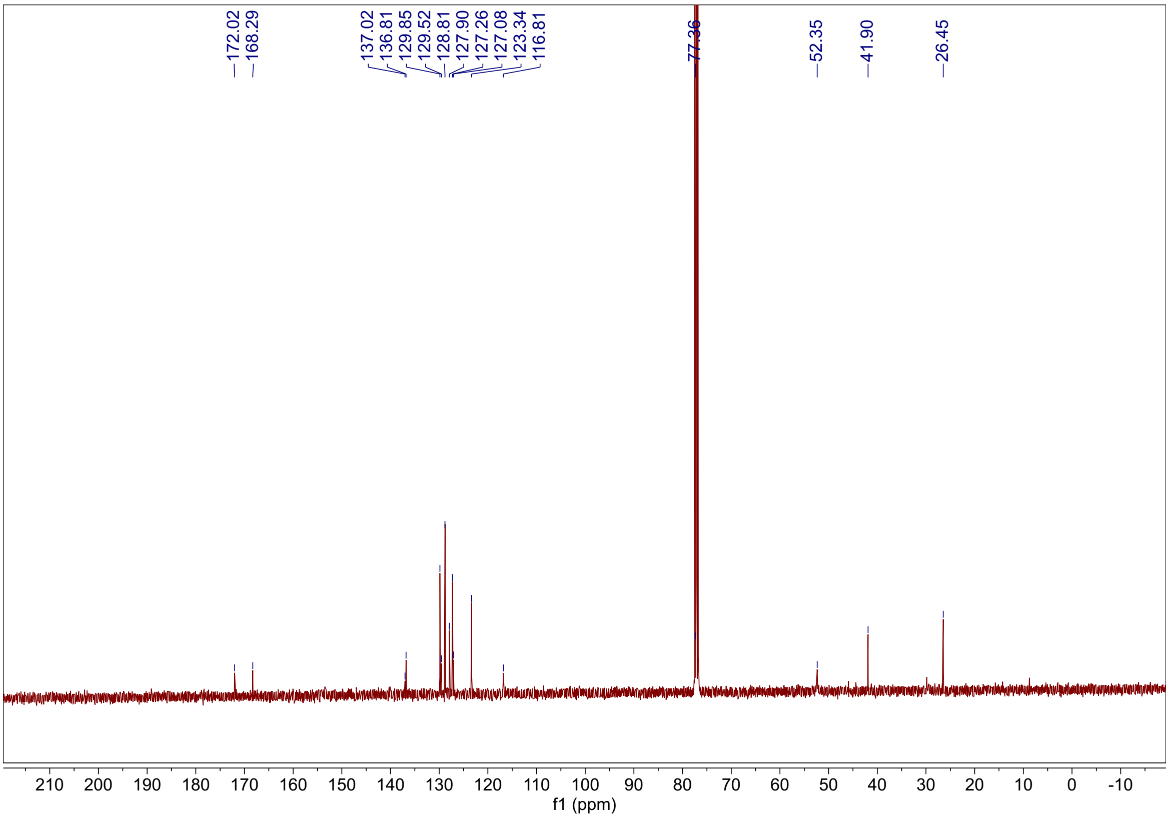
172.02
168.29
137.02
136.81
129.85
129.52
128.81
127.90
127.26
127.08
123.34
116.81
77.36
52.35
41.90
26.45
f1 (ppm)

Compound 43

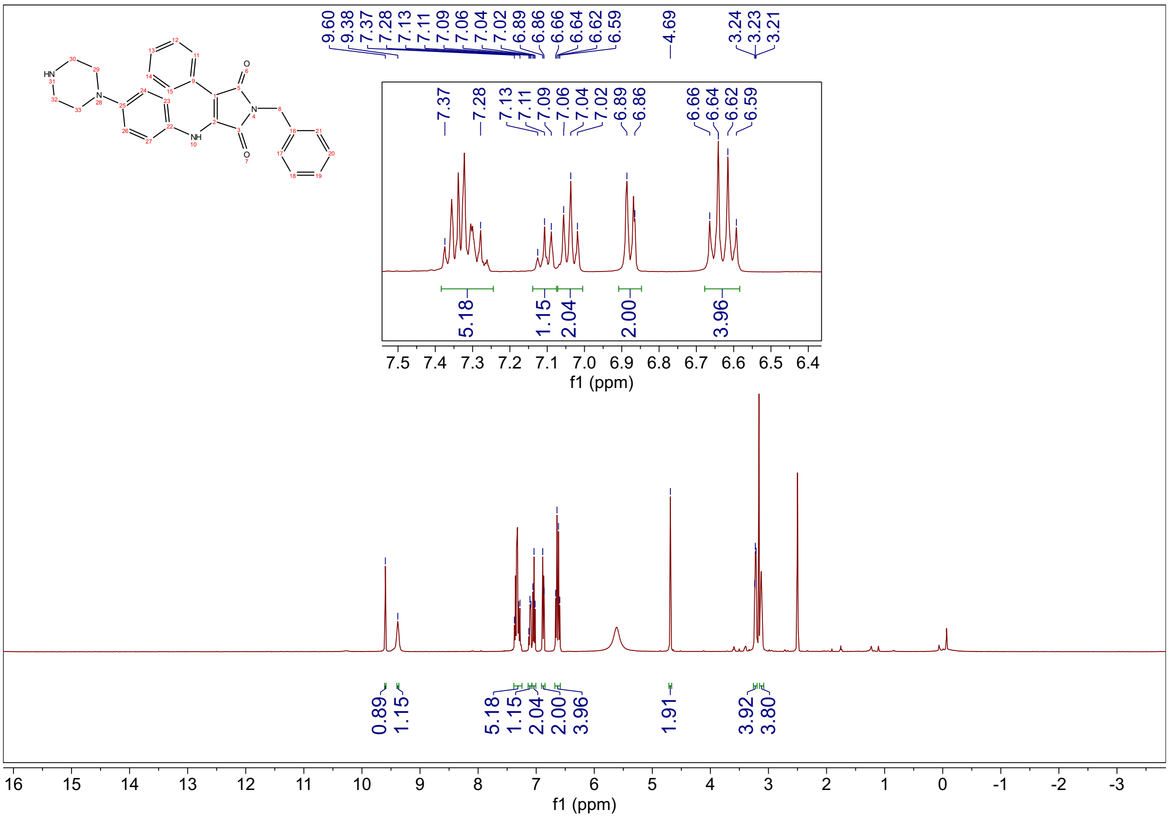
9.60
9.38
7.37
7.28
7.13
7.11
7.09
7.06
7.04
7.02
6.89
6.86
6.66
6.64
6.62
6.59
4.69
3.24
3.23
3.21
5.18
1.15
2.04
2.00
3.96
f1 (ppm)
0.89
1.15
5.18
1.15
2.04
2.00
3.96
1.91
3.92
3.80
f1 (ppm)

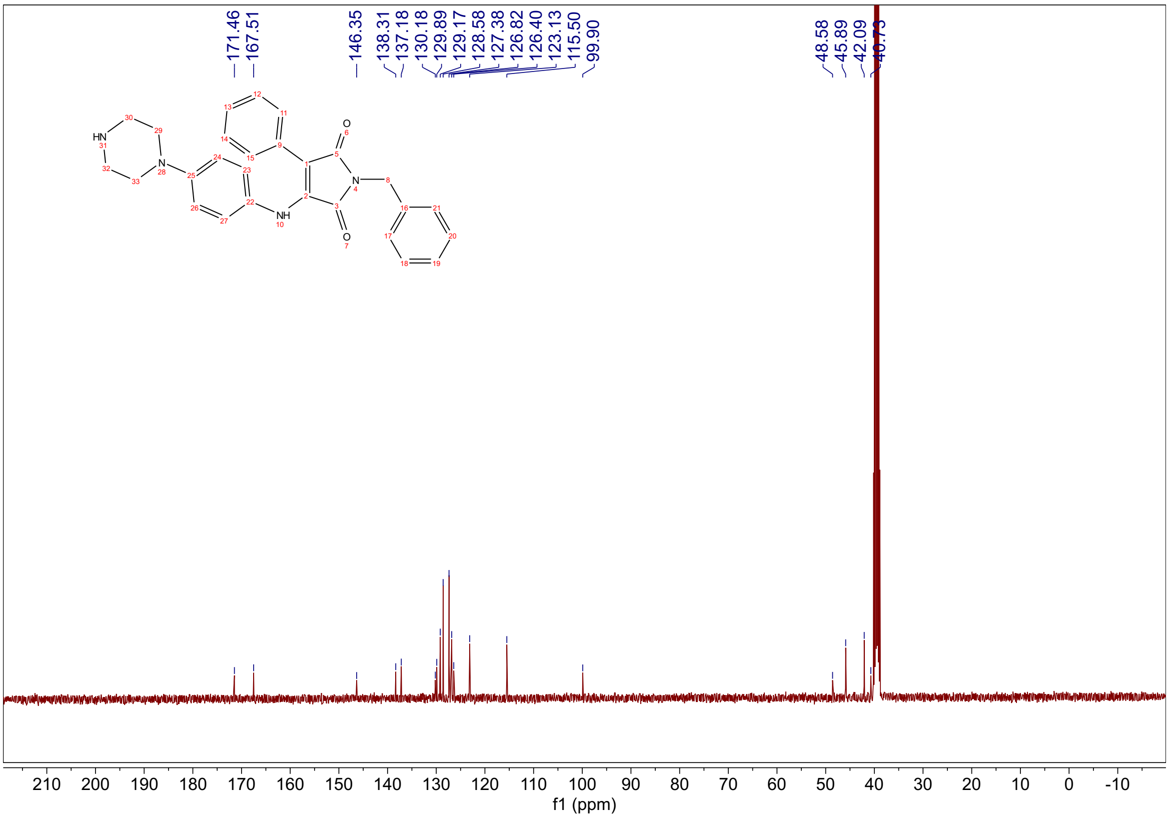
171.46
167.51
146.35
138.31
137.18
130.18
129.89
129.17
128.58
127.38
126.82
126.40
123.13
115.50
99.90
48.58
45.89
42.09
40.73
f1 (ppm)

Compound 44

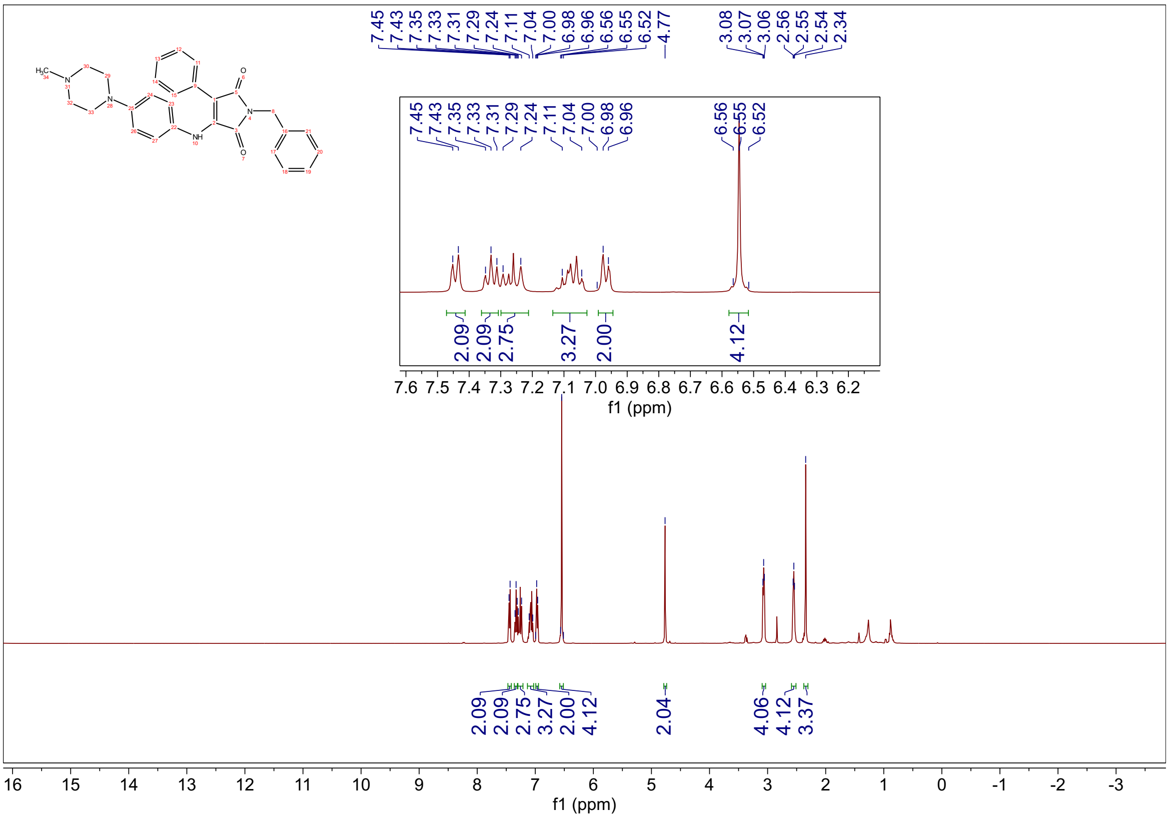
7.45
7.43
7.35
7.33
7.31
7.29
7.24
7.11
7.04
7.00
6.98
6.96
6.56
6.55
6.52
4.77
3.08
3.07
3.06
2.56
2.55
2.54
2.34
2.09
2.09
2.75
3.27
2.00
4.12
2.04
4.06
4.12
3.37
f1 (ppm)

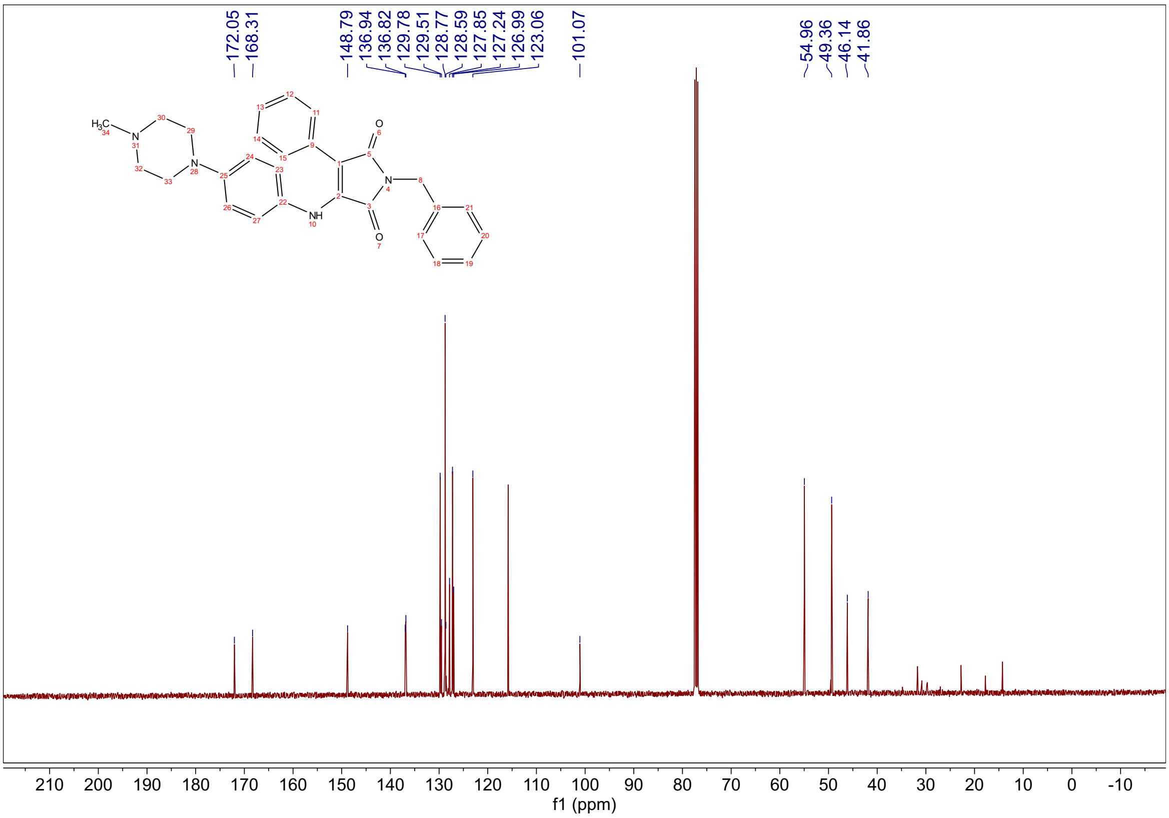
172.05
168.31
148.79
136.94
136.82
129.78
129.51
128.77
128.59
127.85
127.24
126.99
123.06
101.07
54.96
49.36
46.14
41.86
f1 (ppm)

Compound 45

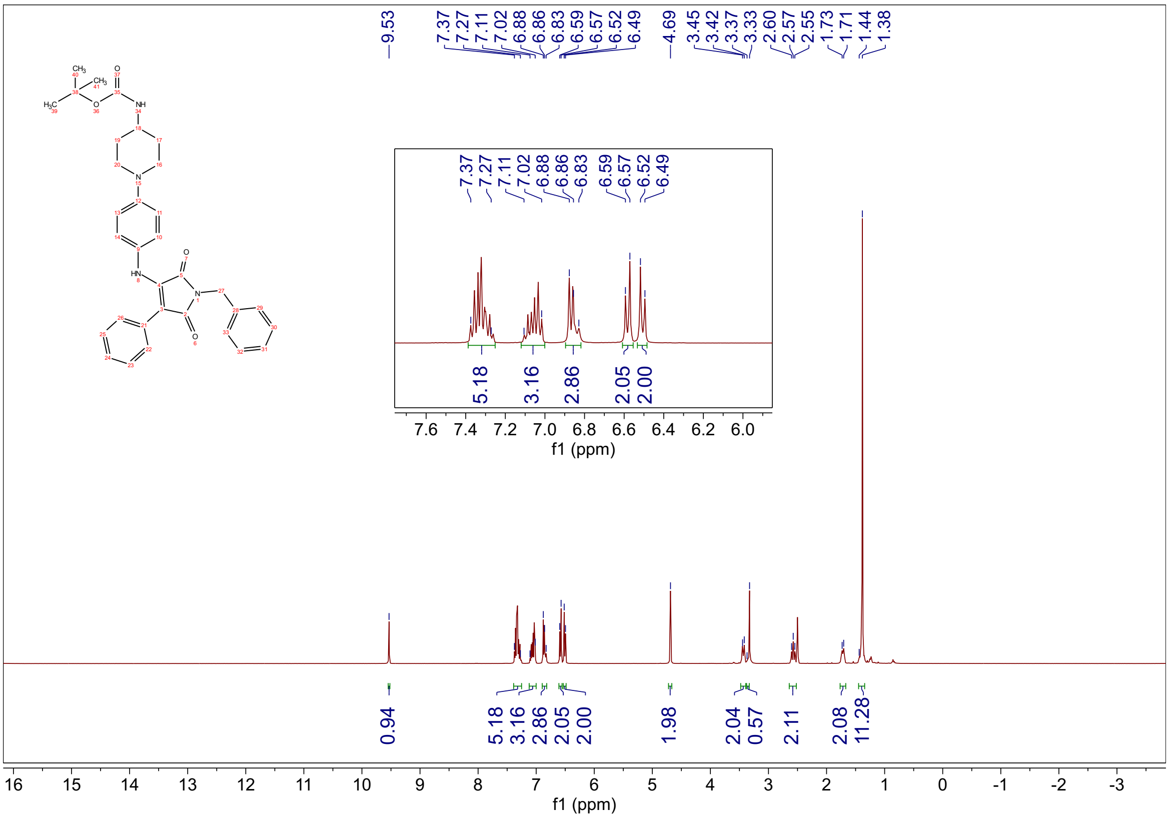

9.53
7.37 7.27 7.11 7.02 6.88 6.86 6.83 6.59 6.57 6.52 6.49
4.69
3.45 3.42 3.37 3.33 2.60 2.57 2.55 1.73 1.71 1.44 1.38
0.94
5.18 3.16 2.86 2.05 2.00
1.98
2.04 0.57
2.11
2.08 11.28
f1 (ppm)

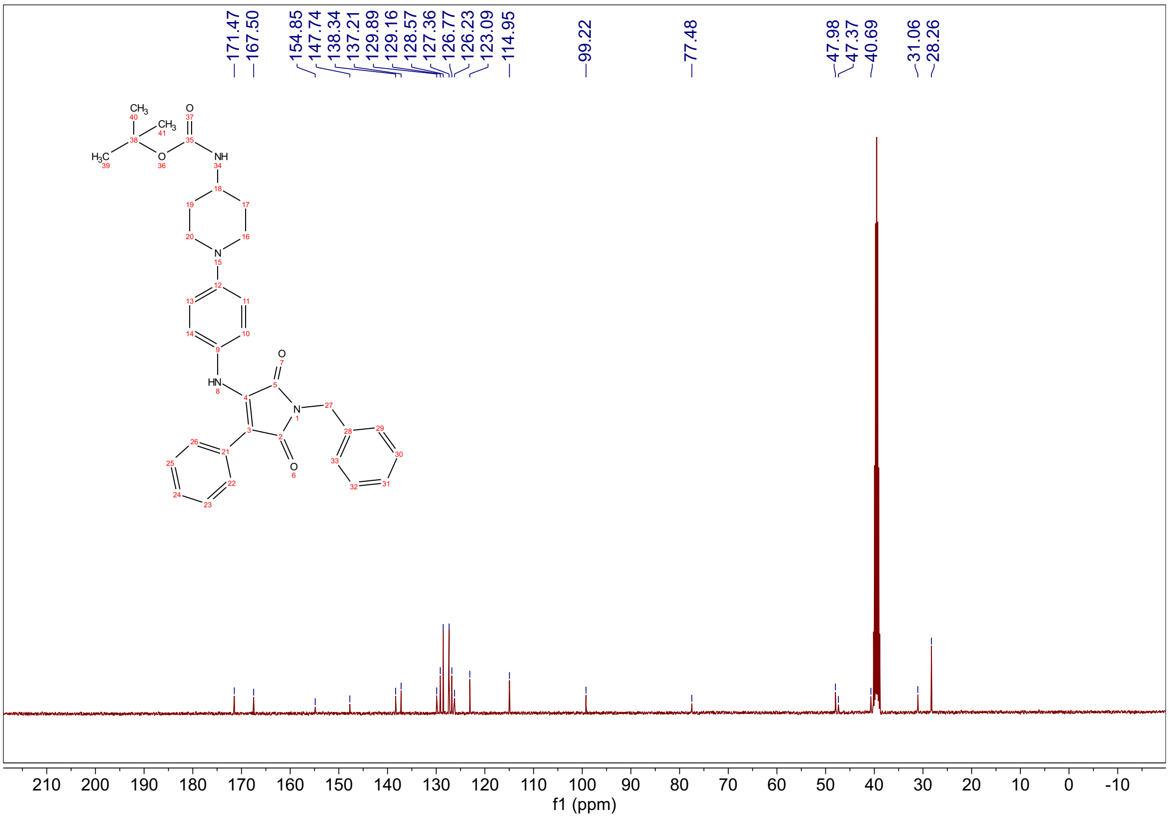

171.47 167.50
154.85 147.74 138.34 137.21 129.89 129.16 128.57 127.36 126.77 126.23 123.09 114.95
99.22
77.48
47.98 47.37 40.69
31.06 28.26
f1 (ppm)

Compound 46

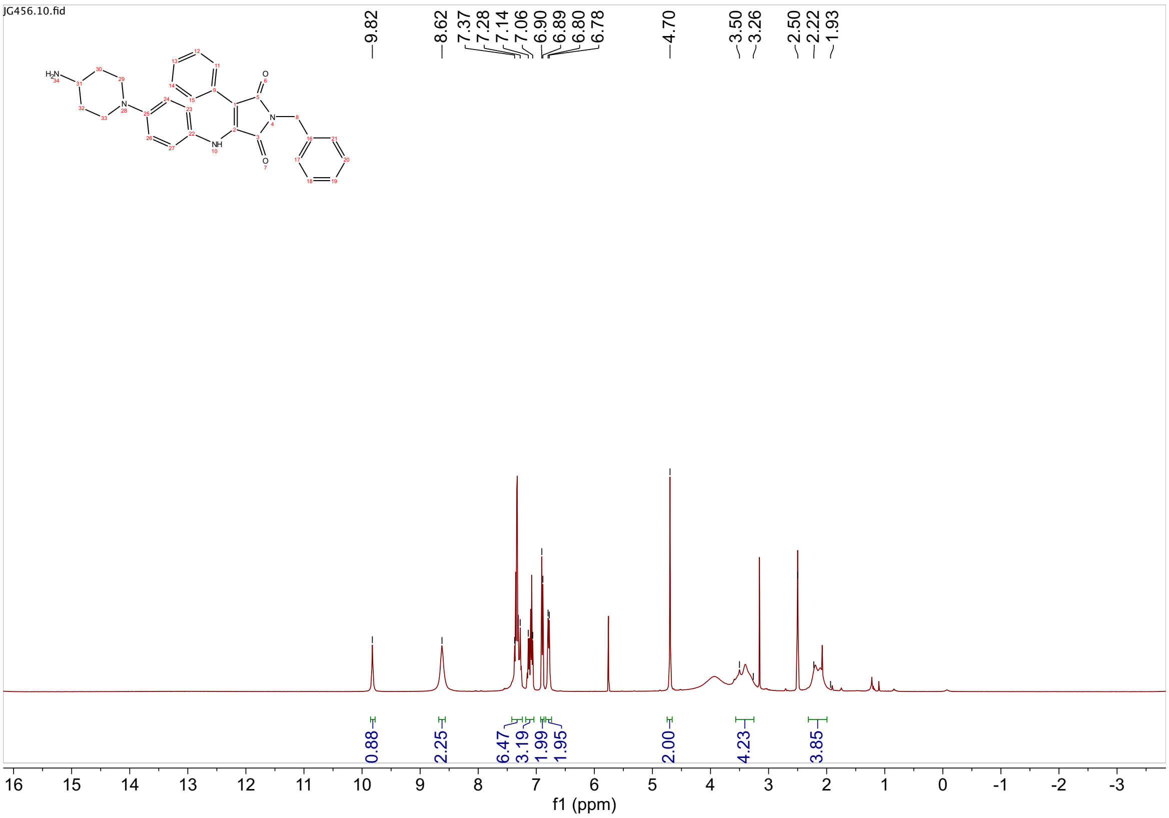
JG456.10.fid
9.82
8.62
7.37
7.28
7.14
7.06
6.90
6.89
6.80
6.78
4.70
3.50
3.26
2.50
2.22
1.93
0.88
2.25
6.47
3.19
1.99
1.95
2.00
4.23
3.85
f1 (ppm)

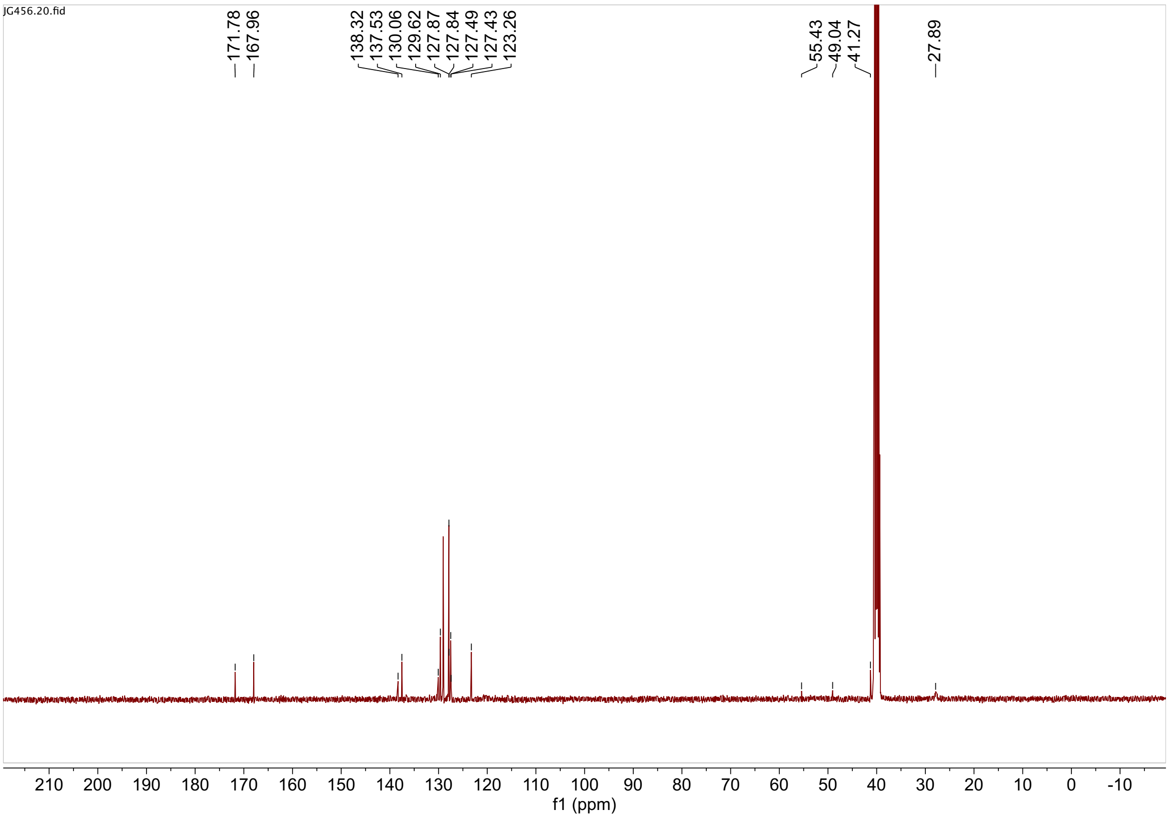
JG456.20.fid
171.78
167.96
138.32
137.53
130.06
129.62
127.87
127.84
127.49
127.43
123.26
55.43
49.04
41.27
27.89
f1 (ppm)

Compound 47

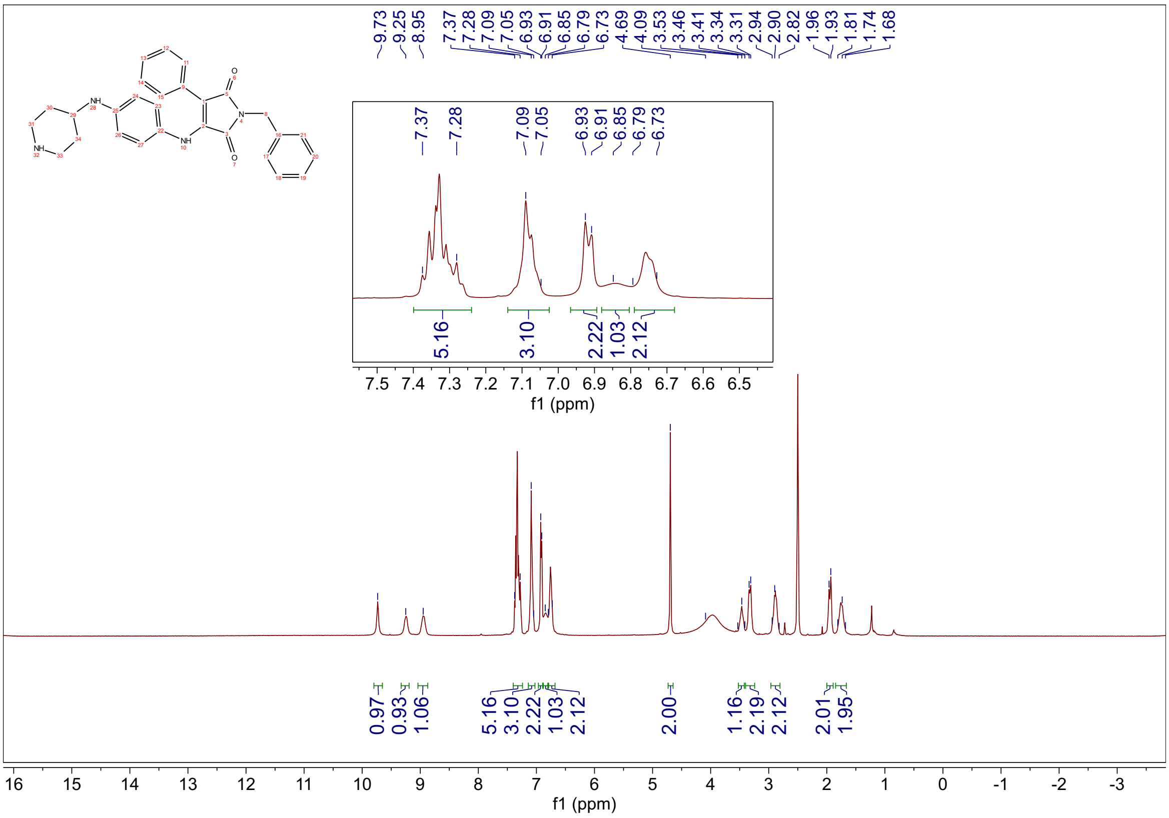

9.73
9.25
8.95
7.37
7.28
7.09
7.05
6.93
6.91
6.85
6.79
6.73
4.69
4.09
3.53
3.46
3.41
3.34
3.31
2.94
2.90
2.82
1.96
1.93
1.81
1.74
1.68
0.97
0.93
1.06
5.16
3.10
2.22
1.03
2.12
2.00
1.16
2.19
2.12
2.01
1.95
f1 (ppm)

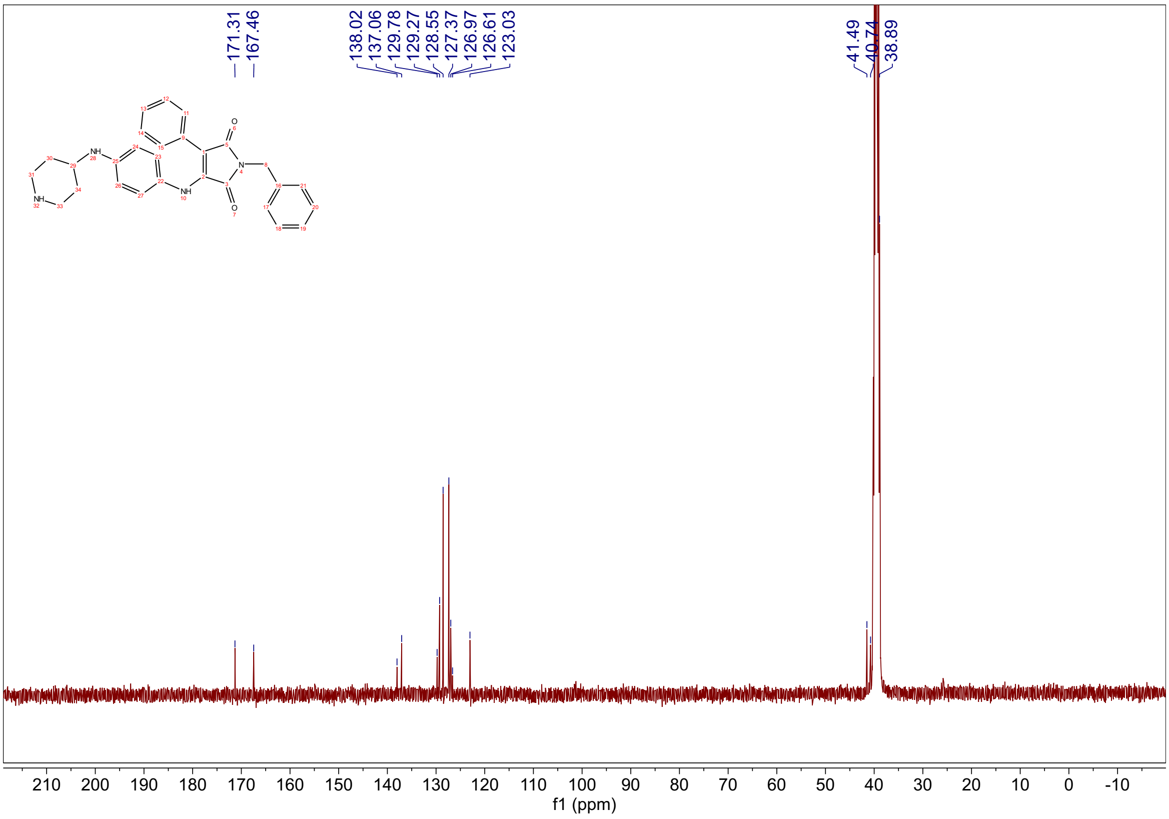

171.31
167.46
138.02
137.06
129.78
129.27
128.55
127.37
126.97
126.61
123.03
41.49
40.74
38.89
f1 (ppm)

Compound 48

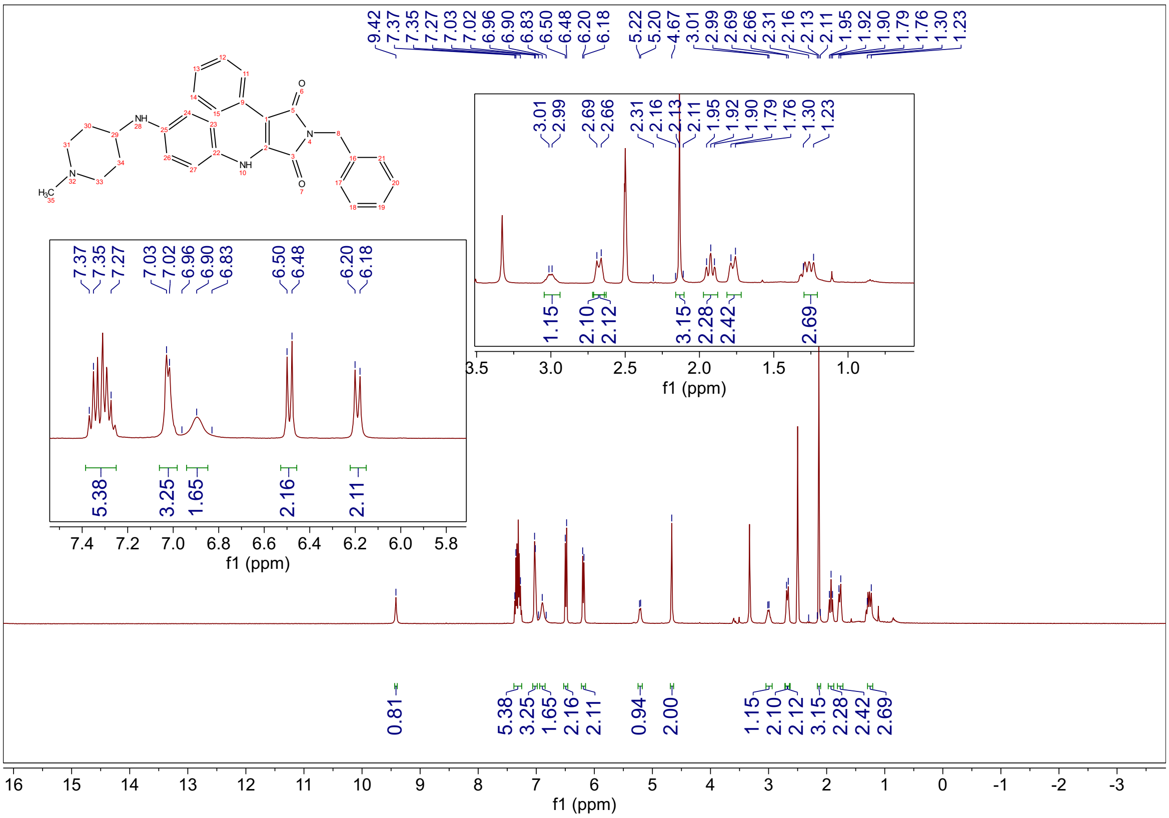
9.42 7.37 7.35 7.27 7.03 7.02 6.96 6.90 6.83 6.50 6.48 6.20 6.18 5.22 5.20 4.67 3.01 2.99 2.69 2.66 2.31 2.16 2.13 2.11 1.95 1.92 1.90 1.79 1.76 1.30 1.23
0.81 5.38 3.25 1.65 2.16 2.11 0.94 2.00 1.15 2.10 2.12 3.15 2.28 2.42 2.69
f1 (ppm)

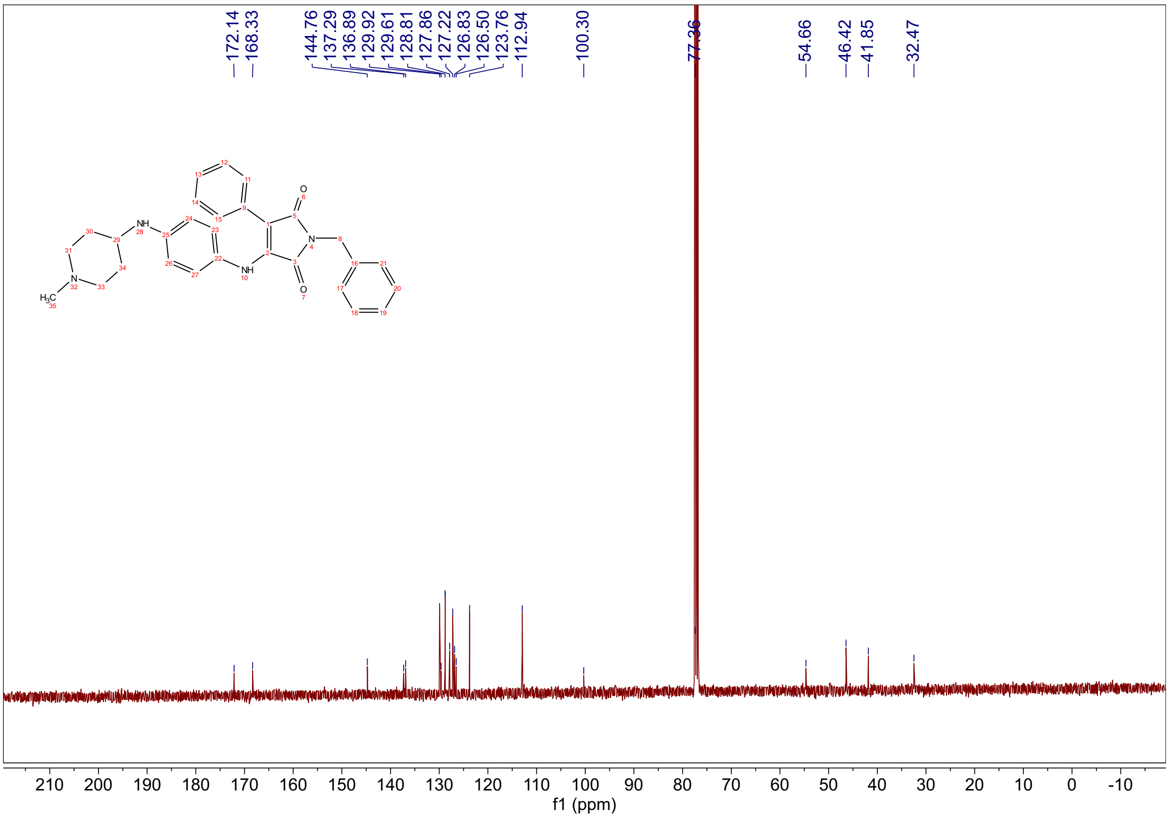
172.14 168.33 144.76 137.29 136.89 129.92 129.61 128.81 127.86 127.22 126.83 126.50 123.76 112.94 100.30 77.36 54.66 46.42 41.85 32.47
f1 (ppm)

Compound 49

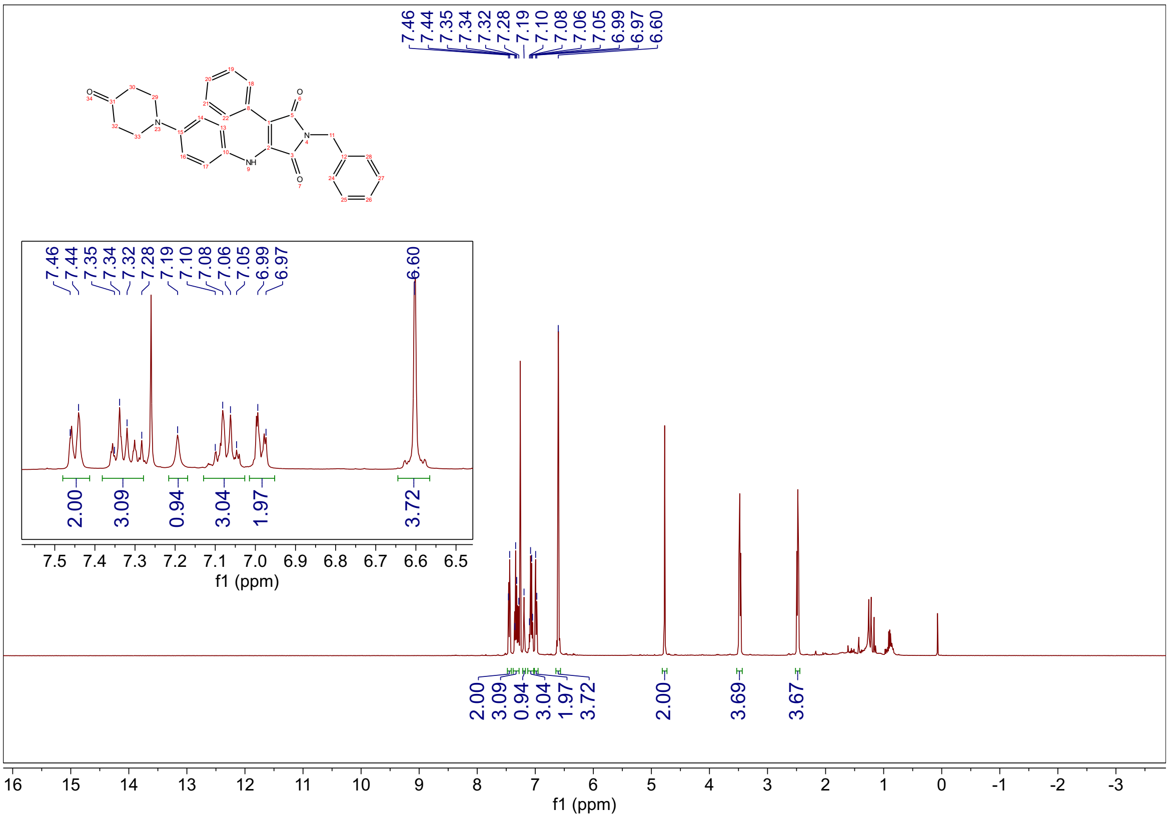

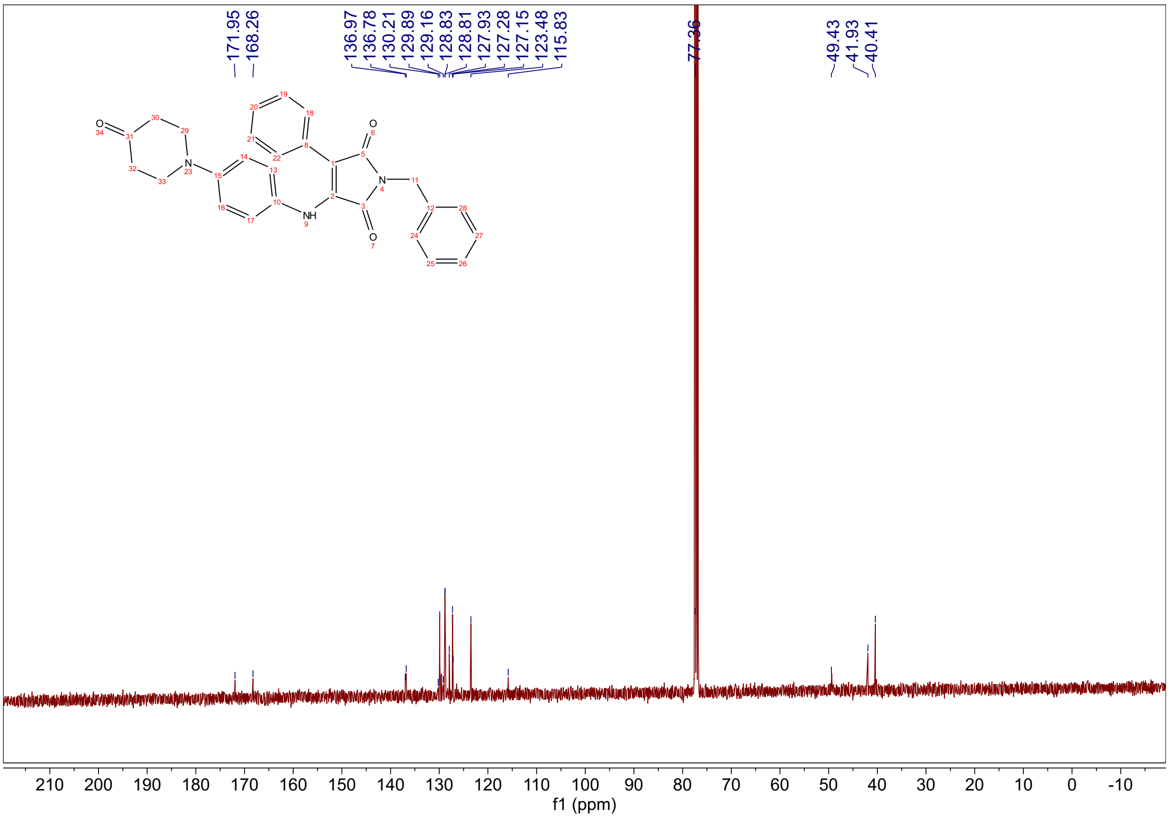

Compound 50

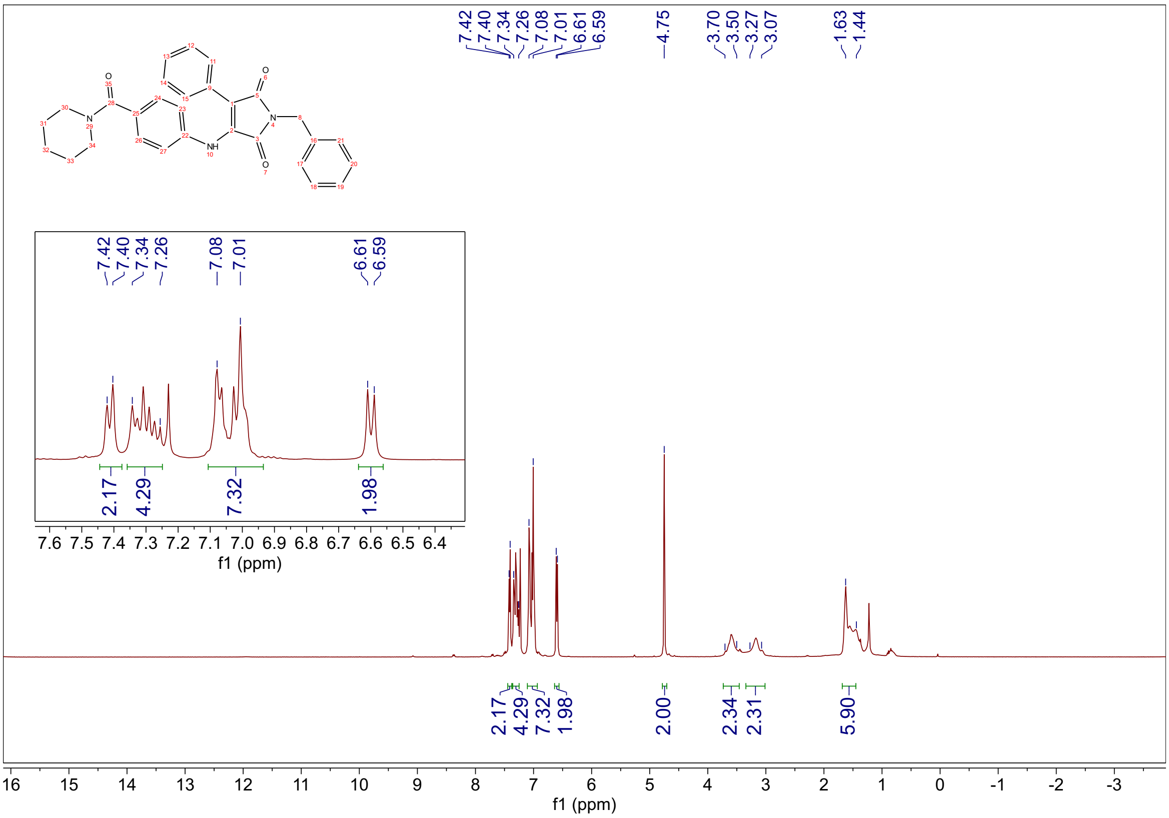

7.42
7.40
7.34
7.26
7.08
7.01
6.61
6.59
4.75
3.70
3.50
3.27
3.07
1.63
1.44
2.17
4.29
7.32
1.98
2.00
2.34
2.31
5.90
f1 (ppm)

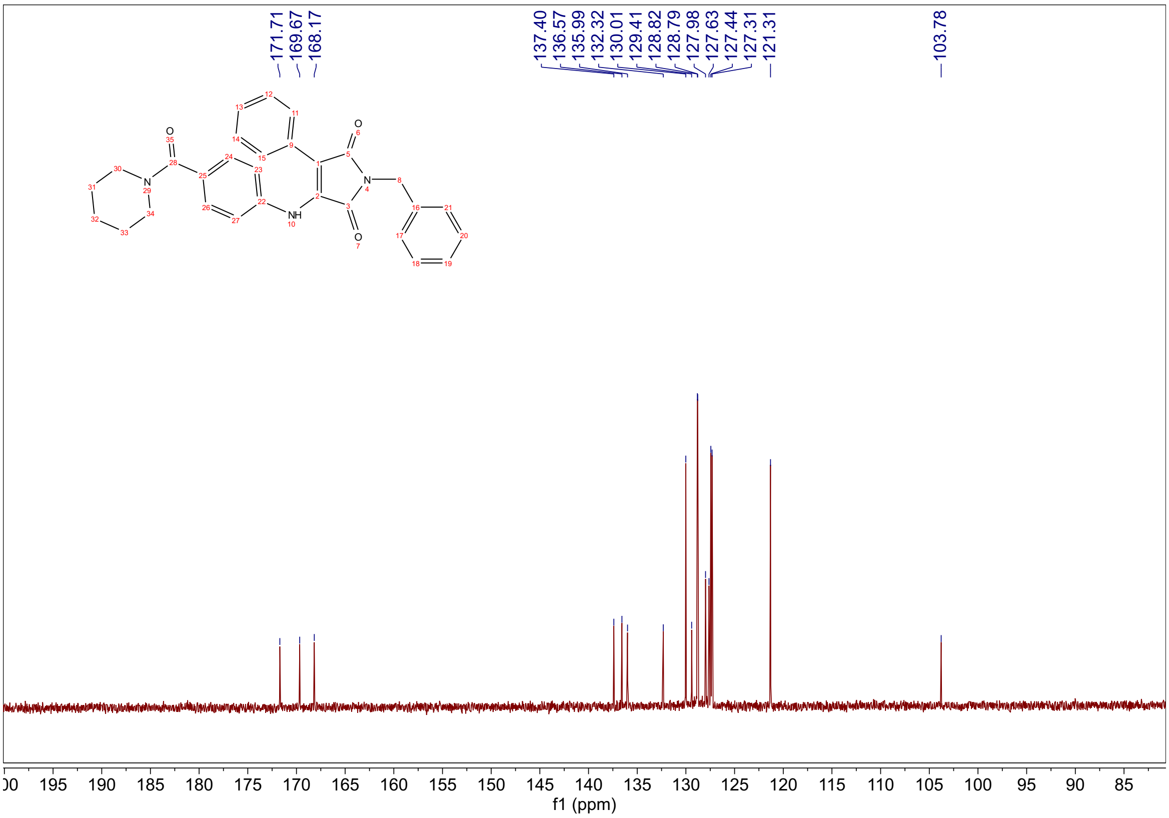

171.71
169.67
168.17
137.40
136.57
135.99
132.32
130.01
129.41
128.82
128.79
127.98
127.63
127.44
127.31
121.31
103.78
f1 (ppm)

Compound 51

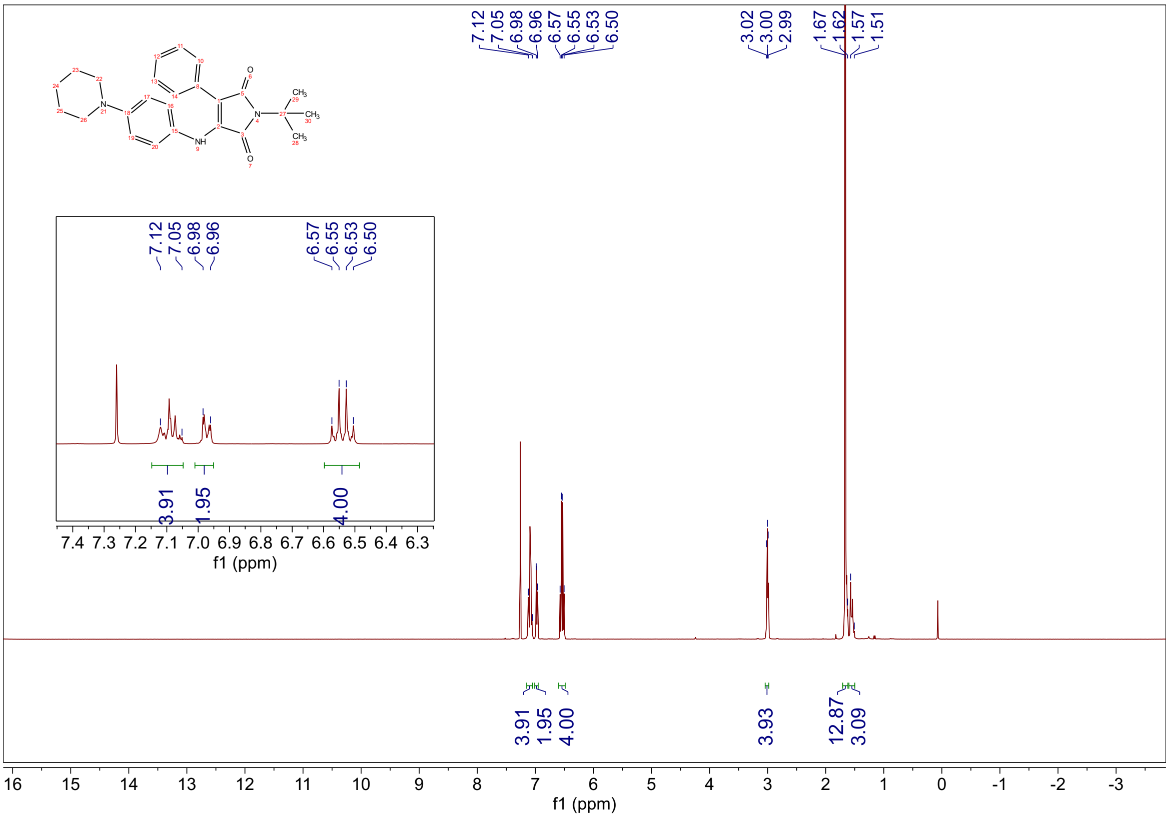

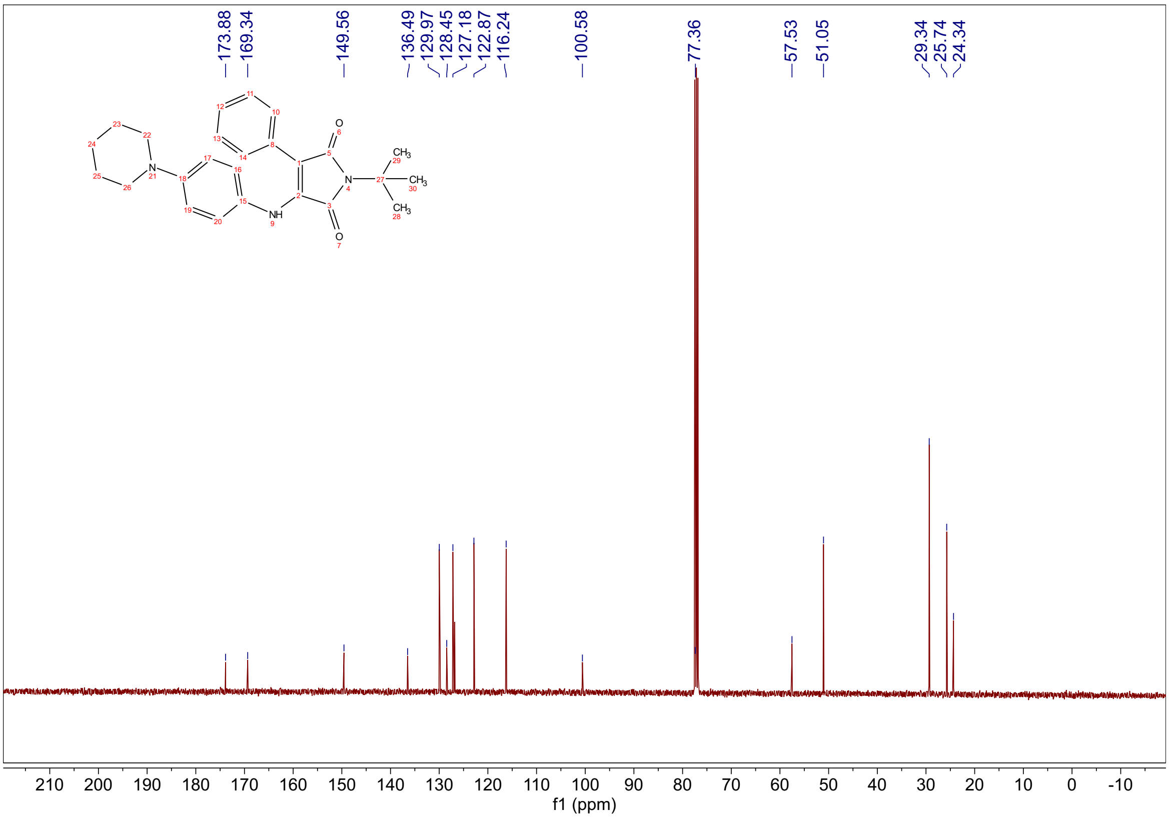

Compound 52

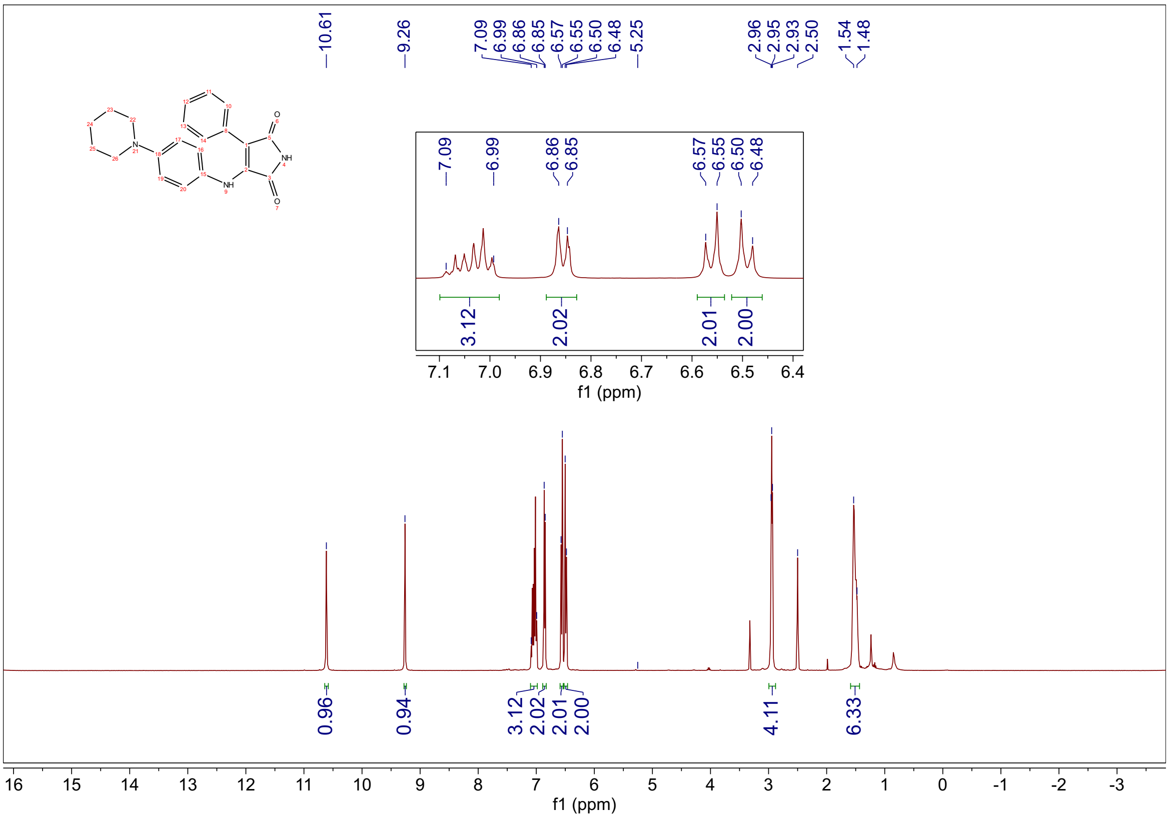
10.61
9.26
7.09
6.99
6.86
6.85
6.57
6.55
6.50
6.48
5.25
2.96
2.95
2.93
2.50
1.54
1.48
3.12
2.02
2.01
2.00
7.1 7.0 6.9 6.8 6.7 6.6 6.5 6.4
f1 (ppm)
0.96
0.94
3.12
2.02
2.01
2.00
4.11
6.33
16 15 14 13 12 11 10 9 8 7 6 5 4 3 2 1 0 -1 -2 -3
f1 (ppm)

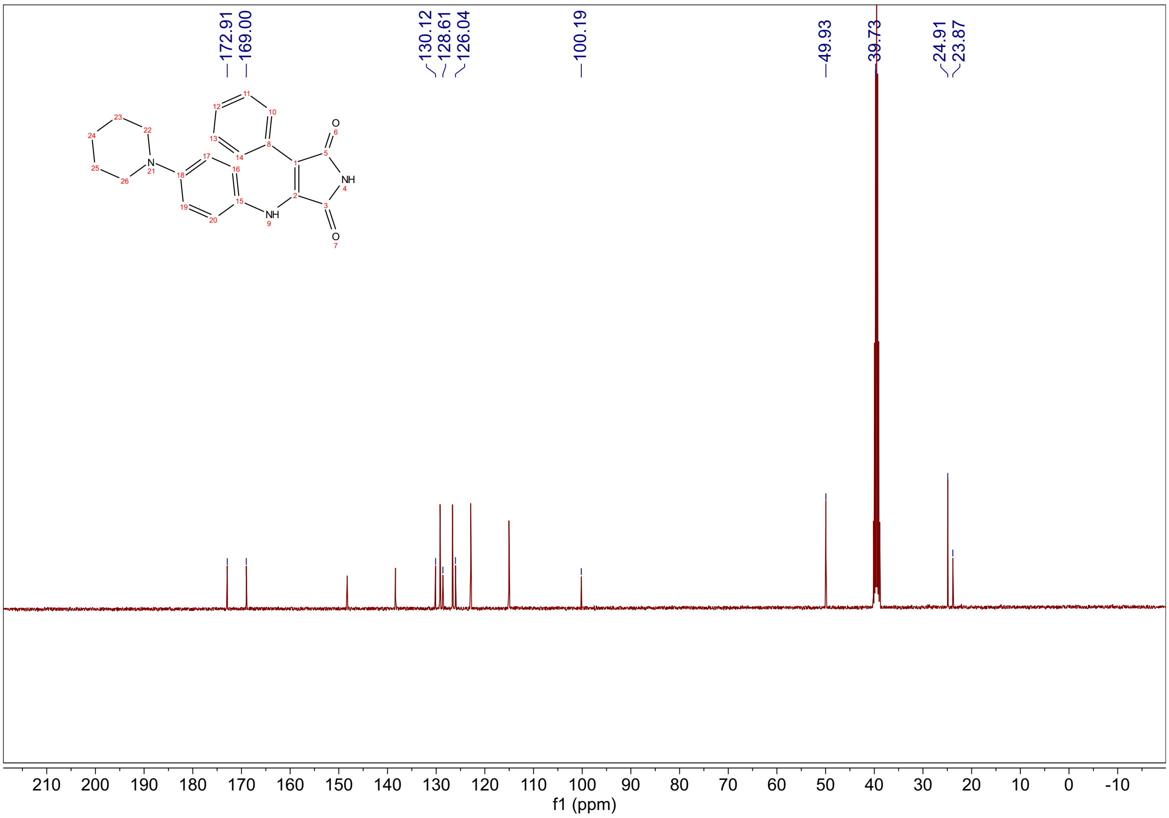
172.91
169.00
130.12
128.61
126.04
100.19
49.93
39.73
24.91
23.87
210 200 190 180 170 160 150 140 130 120 110 100 90 80 70 60 50 40 30 20 10 0 -10
f1 (ppm)

7 HR-MS measurements

	Molecular Formula	Calculated Mass $[M+H]^+$	Measured Mass
3	C28H25N3O3	385.15467	385.15539
16	C24H19FN2O3	403.14525	403.14688
17	C25H22N2O3	399.17032	399.17285
18	C25H19F3N2O3	453.14205	453.1425
19	C25H22N2O4	415.16523	415.1689
20	C28H28N2O3	441.21727	441.22135
21	C23H19N3O3	386.14992	386.1508
22	C24H26N2O3	391.20162	391.20335
23	C21H20N2O3	349.15467	349.15582
24	C21H22N2O3	351.17032	351.1718
25	C17H14N2O3	295.10772	295.10936
26	C24H19NO4	386.13868	386.14167
27	C24H20N2O3	385.15467	385.15483
28	C25H22N2O3	399.17032	399.17257
29	C23H18N2O2	377.12605 $[M+Na]^+$	377.12663
30	C23H17ClN2O2	389.10513	389.10886
31	C23H17FN2O2	373.13468	373.13836
32	C24H20N2O2	369.15975	369.16051
33	C23H18N2O3	371.13902	371.14241
34	C24H20N2O3	385.15467	385.15727
35	C25H23N3O2	398.1863	398.18523
36	C24H19N3O3	398.14992	398.14967
37	C25H19N3O3	410.14992	410.15026
38	C24H18N2O4	399.13393	399.13614
39	C25H22N2O4	415.16523	415.17132
40	C28H27N3O2	438.21760	438.21793
41	C27H25N3O3	440.19687	440.19795
42	C27H25N3O2S	456.17403	456.17653
43	C27H26N4O2	439.21285	439.21155
44	C28H28N4O2	453.2285	453.23008
45	C32H34N4O4	539.26528	539.26531
46	C28H28N4O2	453.2285	453.23044
47	C28H28N4O2	453.2285	453.22823
48	C29H30N4O2	467.24415	467.2436
49	C28H25N3O3	452.19687	452.19682
50	C29H27N3O3	466.21252	466.21351
51	C25H29N3O2	404.23325	404.23388
52	C21H21N3O2	348.17065	348.17138

8 HPLC traces of compounds 3, 16 – 53

Compound 3

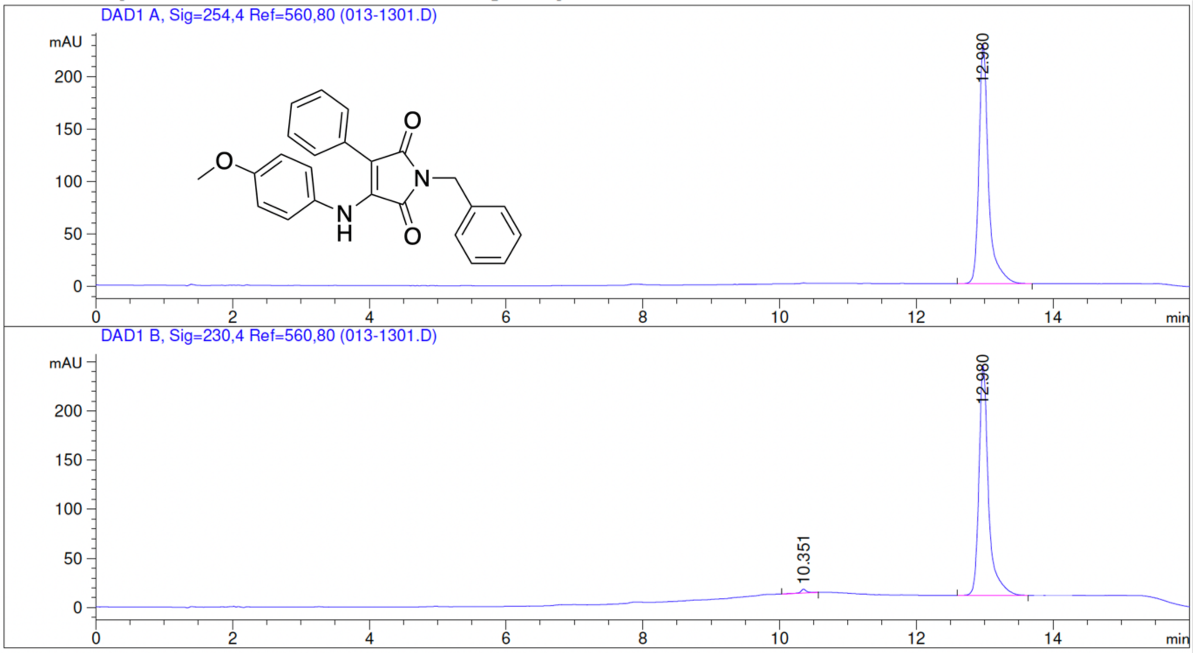

Signal 1: DAD1 A, Sig=254,4 Ref=560,80

Peak #	RetTime [min]	Type	Width [min]	Area [mAU*s]	Height [mAU]	Area %
1	12.980	BB	0.1460	2230.36060	228.24713	100.0000

Signal 2: DAD1 B, Sig=230,4 Ref=560,80

Peak #	RetTime [min]	Type	Width [min]	Area [mAU*s]	Height [mAU]	Area %
1	10.351	BB	0.0836	23.01793	3.97213	1.0013
2	12.980	BB	0.1458	2275.82739	233.38237	98.9987

Compound 16

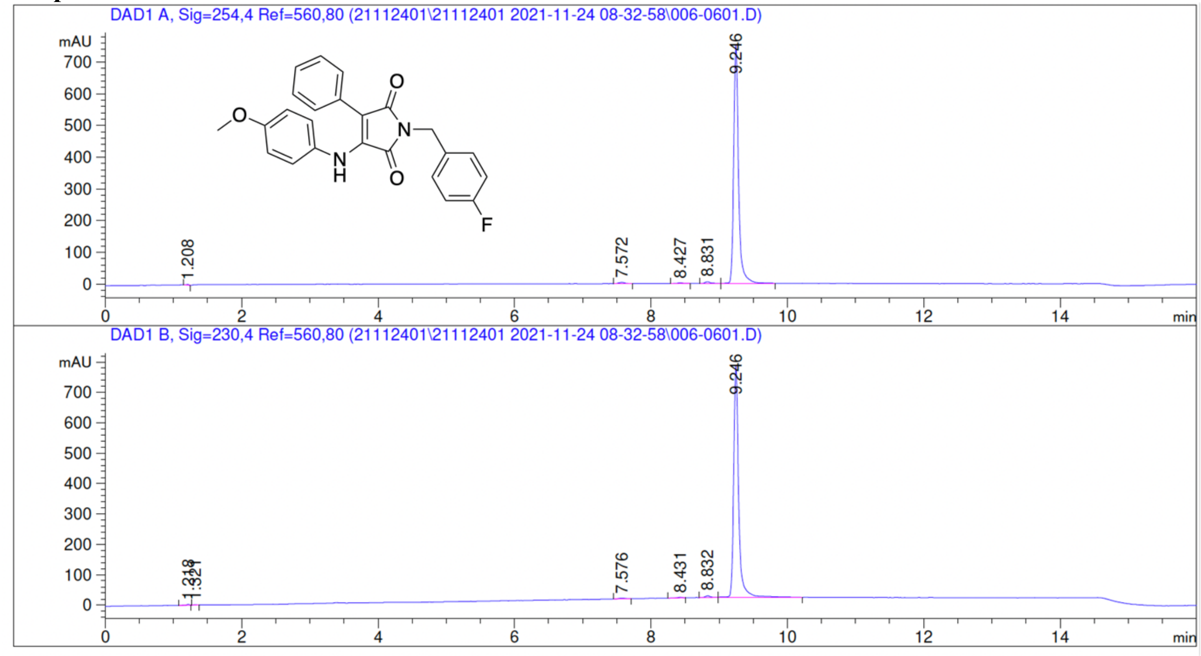

Signal 1: DAD1 A, Sig=254,4 Ref=560,80

Peak #	RetTime [min]	Type	Width [min]	Area [mAU*s]	Height [mAU]	Area %
1	1.208	BB	0.0442	6.50024	2.35602	0.1718
2	7.572	BB	0.1021	25.36325	3.88935	0.6705
3	8.427	BB	0.0763	10.98661	2.12403	0.2904
4	8.831	BB	0.0737	25.27539	5.11470	0.6682
5	9.246	BB	0.0756	3714.53540	752.70166	98.1990

Signal 2: DAD1 B, Sig=230,4 Ref=560,80

Peak #	RetTime [min]	Type	Width [min]	Area [mAU*s]	Height [mAU]	Area %
1	1.218	BB	0.0508	8.98282	2.70323	0.2252
2	1.321	BB	0.0578	4.27196	1.25190	0.1071
3	7.576	BB	0.1023	17.39272	2.52570	0.4359
4	8.431	BB	0.0754	10.27909	1.95170	0.2576
5	8.832	BV	0.0902	37.05935	5.82965	0.9289
6	9.246	VB	0.0777	3911.69019	764.45026	98.0453

Compound 17

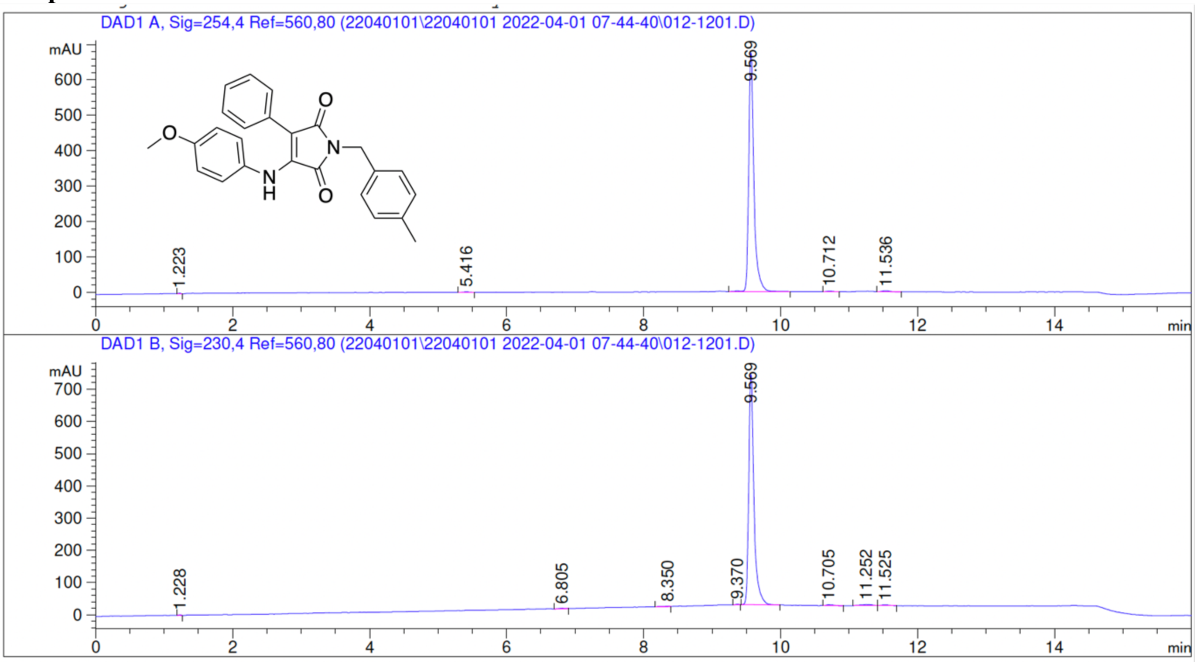

Peak #	RetTime [min]	Type	Width [min]	Area [mAU*s]	Height [mAU]	Area %
1	1.223	BB	0.0406	4.38175	1.78590	0.1216
2	5.416	BB	0.0917	8.98331	1.46146	0.2492
3	9.569	BB	0.0793	3556.72168	677.32458	98.6679
4	10.712	BB	0.0940	11.52039	1.86579	0.3196
5	11.536	BB	0.1282	23.13366	2.80615	0.6418

Peak #	RetTime [min]	Type	Width [min]	Area [mAU*s]	Height [mAU]	Area %
1	1.228	BB	0.0400	4.59242	1.91439	0.1186
2	6.805	BB	0.0807	7.81163	1.36553	0.2018
3	8.350	BB	0.0885	7.18756	1.15626	0.1857
4	9.370	BB	0.0581	4.33911	1.20217	0.1121
5	9.569	BB	0.0792	3754.57251	716.05054	96.9944
6	10.705	BB	0.1066	21.00925	2.70528	0.5427
7	11.252	BV	0.1641	46.89461	3.59804	1.2115
8	11.525	VB	0.1186	24.50776	2.78724	0.6331

Compound 18

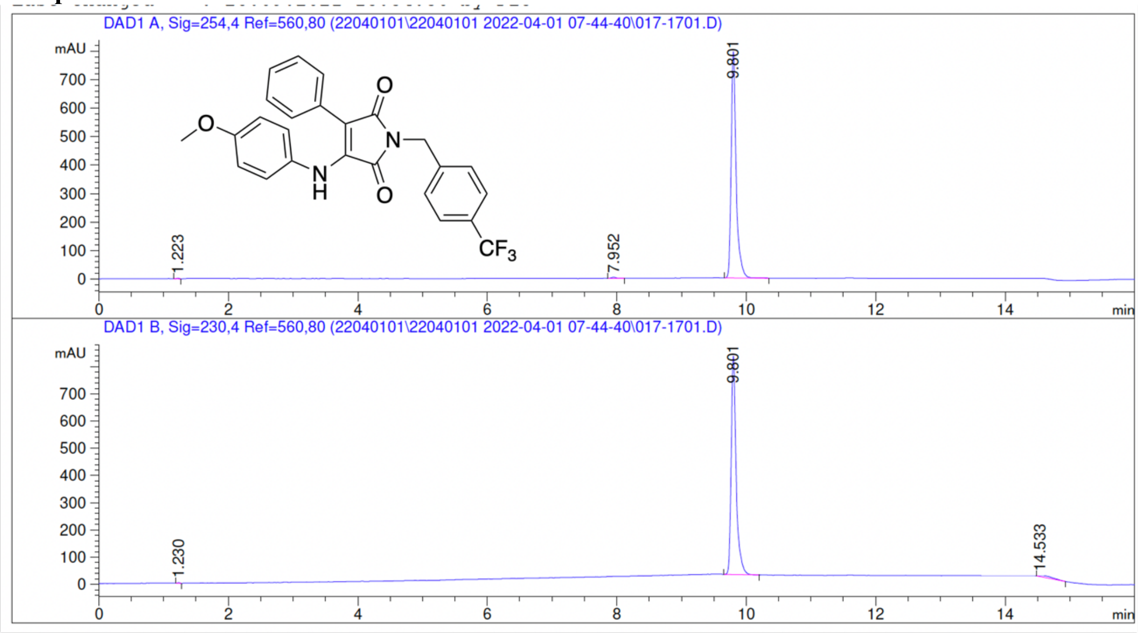

Peak #	RetTime [min]	Type	Width [min]	Area [mAU*s]	Height [mAU]	Area %
1	1.223	BB	0.0457	6.07262	2.10009	0.1425
2	7.952	BB	0.0717	17.21859	3.60578	0.4039
3	9.801	BB	0.0802	4239.55957	795.70337	99.4536

Peak #	RetTime [min]	Type	Width [min]	Area [mAU*s]	Height [mAU]	Area %
1	1.230	BB	0.0403	5.49889	2.26458	0.1252
2	9.801	BB	0.0804	4296.22119	803.47729	97.7802
3	14.533	BB	0.3280	92.03176	3.39824	2.0946

Compound 19

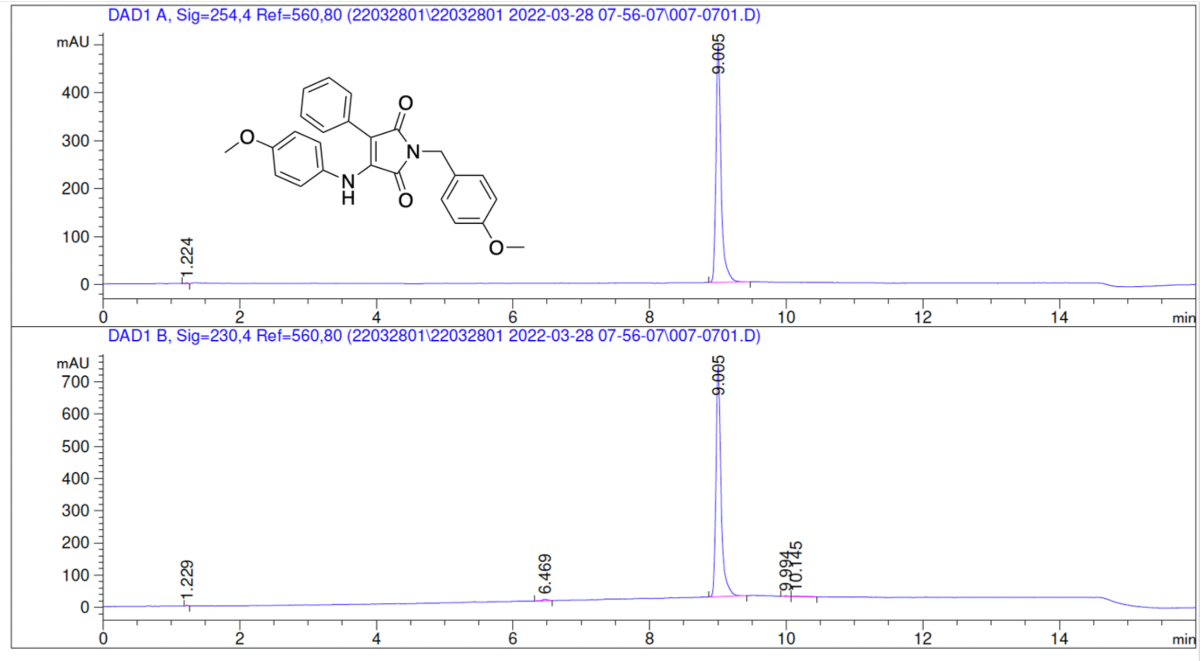

Peak #	RetTime [min]	Type	Width [min]	Area [mAU*s]	Height [mAU]	Area %
1	1.224	BB	0.0416	4.94211	1.94529	0.1895
2	9.005	BB	0.0772	2602.67822	496.29013	99.8105

Peak #	RetTime [min]	Type	Width [min]	Area [mAU*s]	Height [mAU]	Area %
1	1.229	BB	0.0401	5.50184	2.28108	0.1445
2	6.469	BB	0.0770	22.76567	4.21630	0.5981
3	9.005	BB	0.0772	3755.11523	715.94641	98.6572
4	9.994	BV	0.0781	7.28056	1.41319	0.1913
5	10.145	VB	0.1152	15.56375	1.90752	0.4089

Compound 20

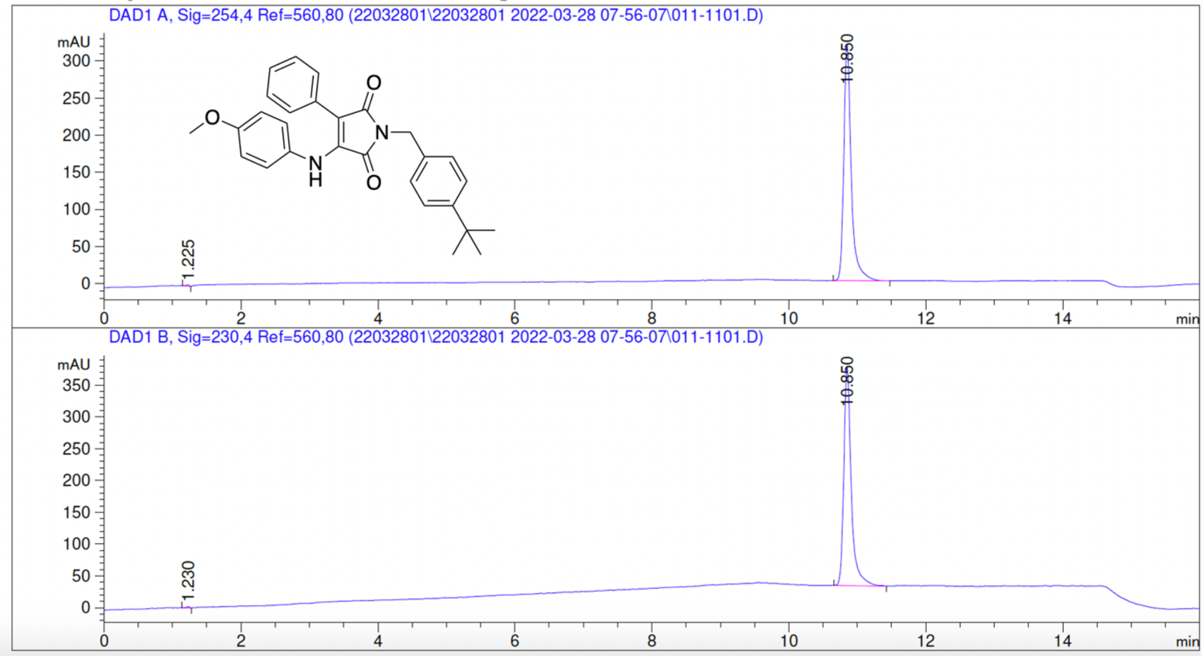

Peak #	RetTime [min]	Type	Width [min]	Area [mAU*s]	Height [mAU]	Area %
1	1.225	BB	0.0498	5.69973	1.75731	0.2351
2	10.850	BB	0.1126	2418.85864	318.52859	99.7649

Peak #	RetTime [min]	Type	Width [min]	Area [mAU*s]	Height [mAU]	Area %
1	1.230	BB	0.0512	5.86853	1.74597	0.2248
2	10.850	BB	0.1125	2604.64429	343.27380	99.7752

Compound 21

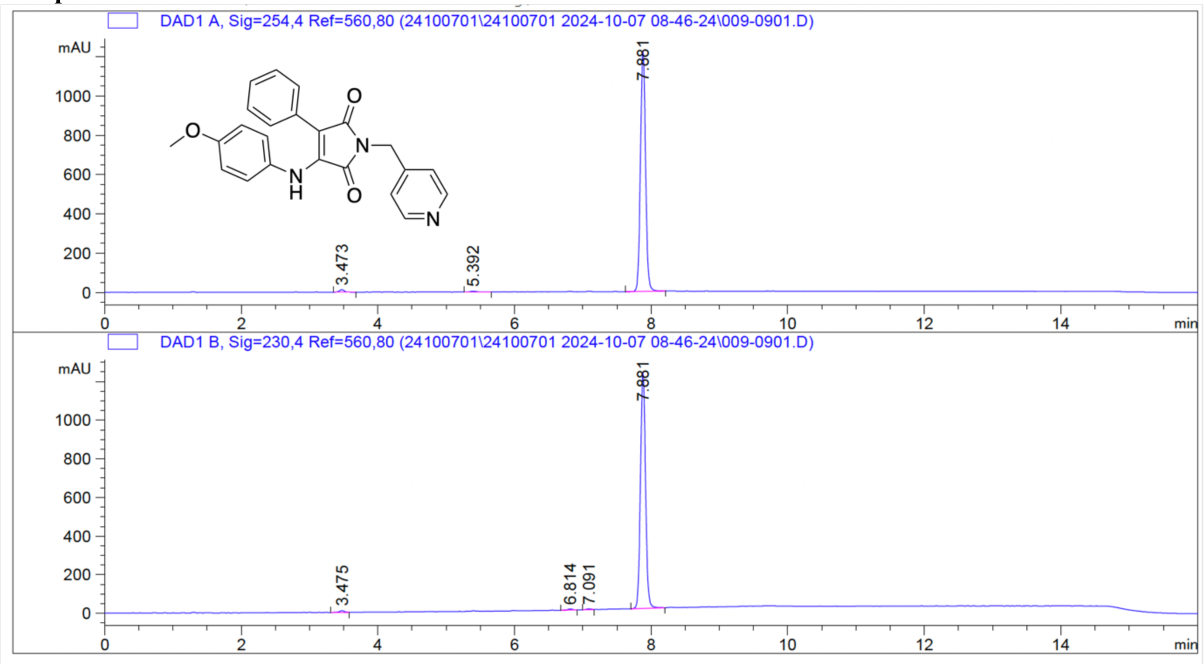

Signal 1: DAD1 A, Sig=254,4 Ref=560,80

Peak #	RetTime [min]	Type	Width [min]	Area [mAU*s]	Height [mAU]	Area %
1	3.473	BB	0.0773	66.07075	12.99332	1.0276
2	5.392	BB	0.0963	29.88431	4.34101	0.4648
3	7.881	BB	0.0803	6333.77441	1225.52820	98.5076

Signal 2: DAD1 B, Sig=230,4 Ref=560,80

Peak #	RetTime [min]	Type	Width [min]	Area [mAU*s]	Height [mAU]	Area %
1	3.475	BB	0.0780	43.24808	8.40755	0.6780
2	6.814	BB	0.0750	27.77728	5.49552	0.4355
3	7.091	BB	0.0657	18.90596	4.43947	0.2964
4	7.881	BB	0.0800	6288.96680	1223.83032	98.5902

Compound 22

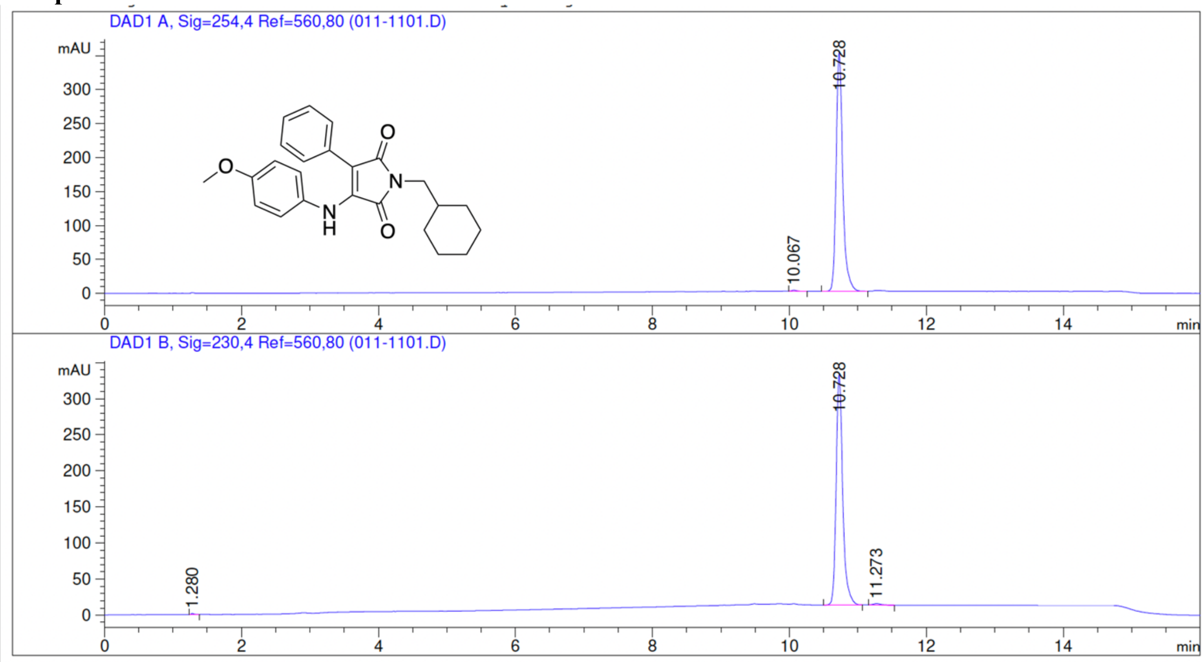

Signal 1: DAD1 A, Sig=254,4 Ref=560,80

Peak #	RetTime [min]	Type	Width [min]	Area [mAU*s]	Height [mAU]	Area %
1	10.067	BB	0.0744	6.24199	1.29091	0.2655
2	10.728	BB	0.0994	2345.08887	353.42694	99.7345

Peak #	RetTime [min]	Type	Width [min]	Area [mAU*s]	Height [mAU]	Area %
1	1.280	BB	0.0504	5.58669	1.69558	0.2604
2	10.728	BB	0.0992	2126.92505	321.29700	99.1426
3	11.273	BB	0.1093	12.80651	1.75209	0.5970

Compound 23

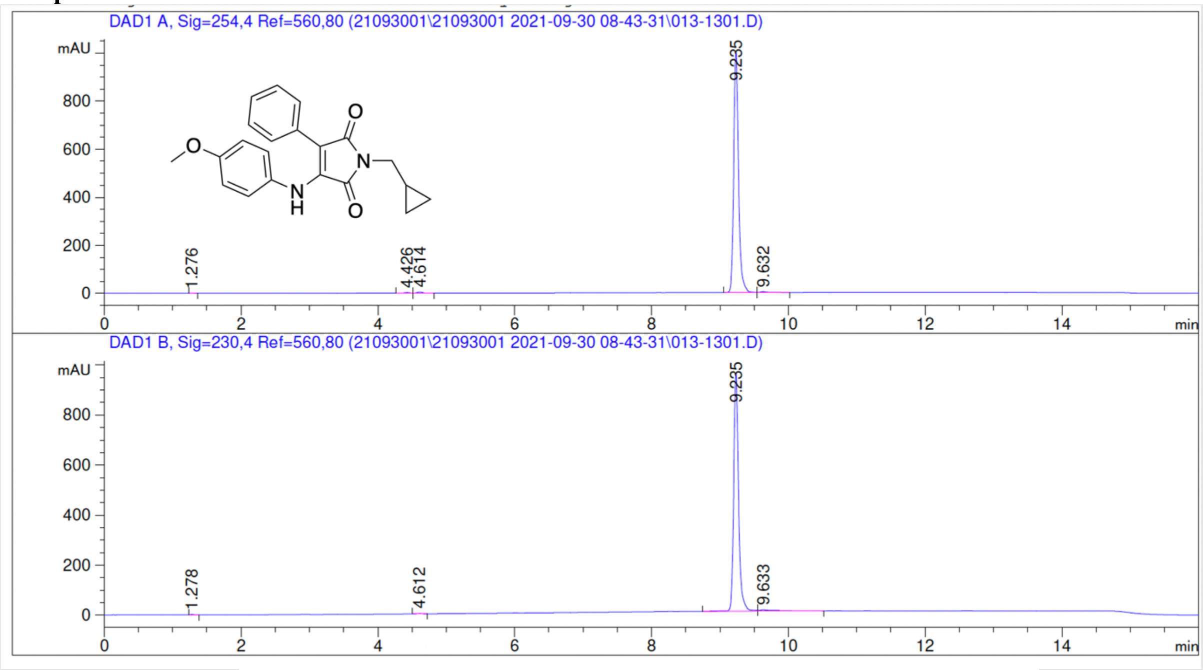

Signal 1: DAD1 A, Sig=254,4 Ref=560,80

Peak #	RetTime [min]	Type	Width [min]	Area [mAU*s]	Height [mAU]	Area %
1	1.276	BB	0.0476	4.24220	1.38849	0.0854
2	4.426	BV	0.0904	12.57963	2.14581	0.2532
3	4.614	VB	0.0781	25.69018	4.98502	0.5172
4	9.235	BV	0.0748	4895.31006	1004.85828	98.5493
5	9.632	VB	0.1082	29.55129	3.73879	0.5949

Signal 2: DAD1 B, Sig=230,4 Ref=560,80

Peak #	RetTime [min]	Type	Width [min]	Area [mAU*s]	Height [mAU]	Area %
1	1.278	BB	0.0497	6.15568	1.90400	0.1281
2	4.612	BB	0.0794	9.63850	1.89221	0.2006
3	9.235	BV	0.0754	4683.60059	952.64349	97.4939
4	9.633	VB	0.2401	104.59829	5.44611	2.1773

Compound 24

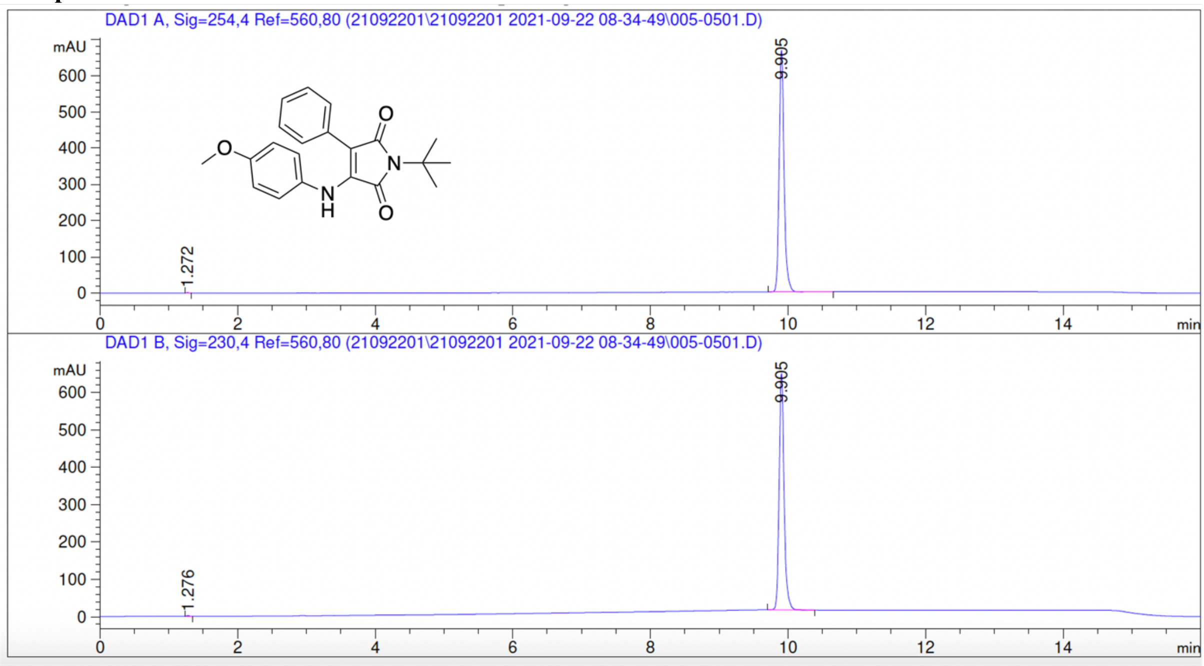

Signal 1: DAD1 A, Sig=254,4 Ref=560,80

Peak #	RetTime [min]	Type	Width [min]	Area [mAU*s]	Height [mAU]	Area %
1	1.272	BB	0.0403	4.38132	1.80570	0.1321
2	9.905	BB	0.0759	3313.15625	668.09076	99.8679

Peak #	RetTime [min]	Type	Width [min]	Area [mAU*s]	Height [mAU]	Area %
1	1.276	BB	0.0438	6.34970	2.33092	0.2015
2	9.905	BB	0.0759	3144.33716	633.81750	99.7985

Compound 25

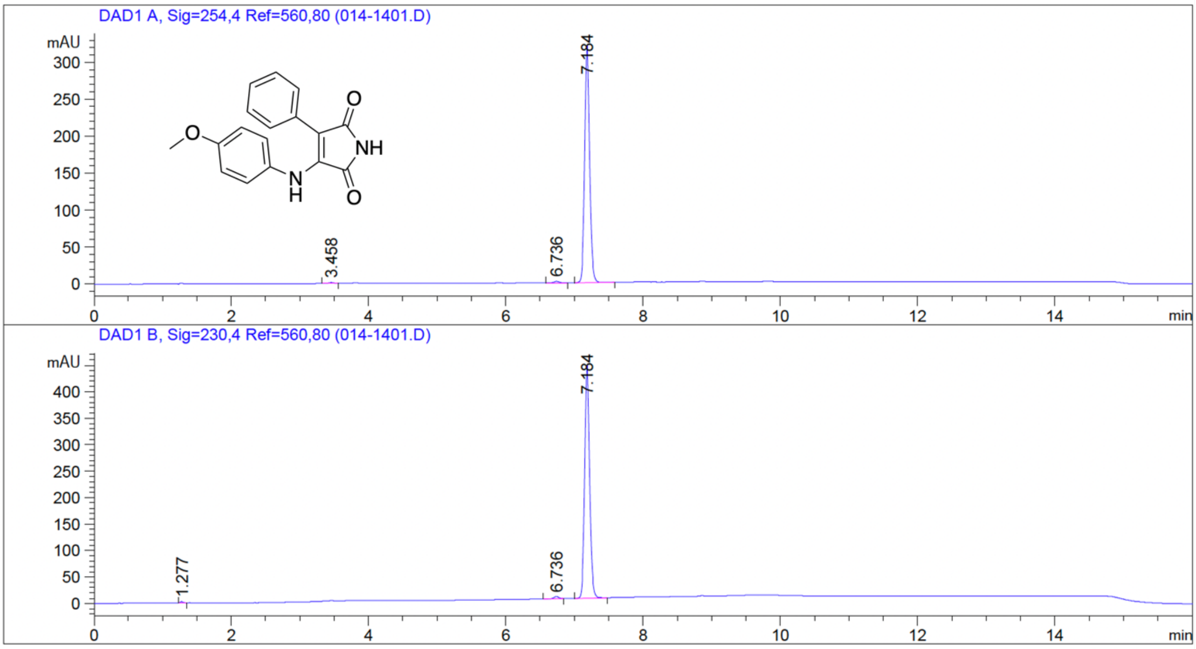

Signal 1: DAD1 A, Sig=254,4 Ref=560,80

Peak #	RetTime [min]	Type	Width [min]	Area [mAU*s]	Height [mAU]	Area %
1	3.458	BB	0.0744	6.47902	1.29345	0.3954
2	6.736	BB	0.0807	10.00199	1.92389	0.6105
3	7.184	BB	0.0788	1621.93494	321.76480	98.9941

Signal 2: DAD1 B, Sig=230,4 Ref=560,80

Peak #	RetTime [min]	Type	Width [min]	Area [mAU*s]	Height [mAU]	Area %
1	1.277	BB	0.0473	7.36642	2.43637	0.3273
2	6.736	BB	0.0754	22.11216	4.49646	0.9824
3	7.184	BB	0.0787	2221.23950	441.36618	98.6903

Compound 26

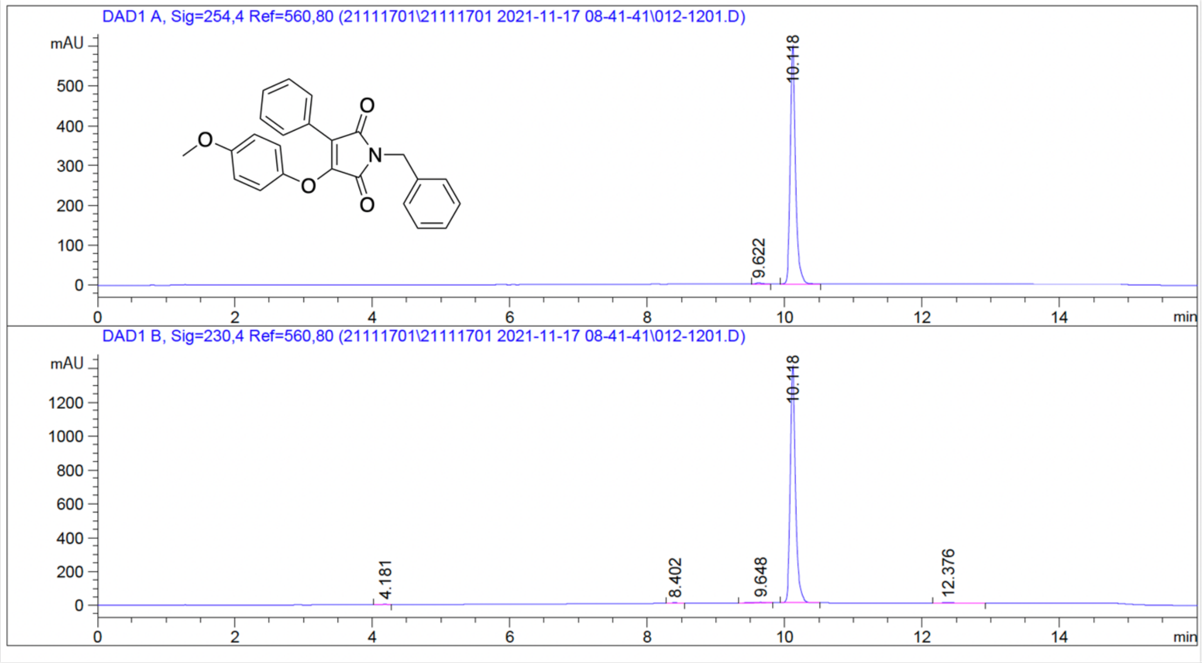

Signal 1: DAD1 A, Sig=254,4 Ref=560,80

Peak #	RetTime [min]	Type	Width [min]	Area [mAU*s]	Height [mAU]	Area %
1	9.622	BB	0.0913	13.35503	2.24697	0.4199
2	10.118	BB	0.0819	3167.47583	597.32642	99.5801

Signal 2: DAD1 B, Sig=230,4 Ref=560,80

Peak #	RetTime [min]	Type	Width [min]	Area [mAU*s]	Height [mAU]	Area %
1	4.181	BB	0.0743	5.68855	1.17956	0.0758
2	8.402	BB	0.0753	22.19486	4.51771	0.2956
3	9.648	BB	0.1382	24.72513	2.66670	0.3293
4	10.118	BB	0.0819	7425.96045	1398.50952	98.9082
5	12.376	BB	0.1679	29.36573	2.67859	0.3911

Compound 27

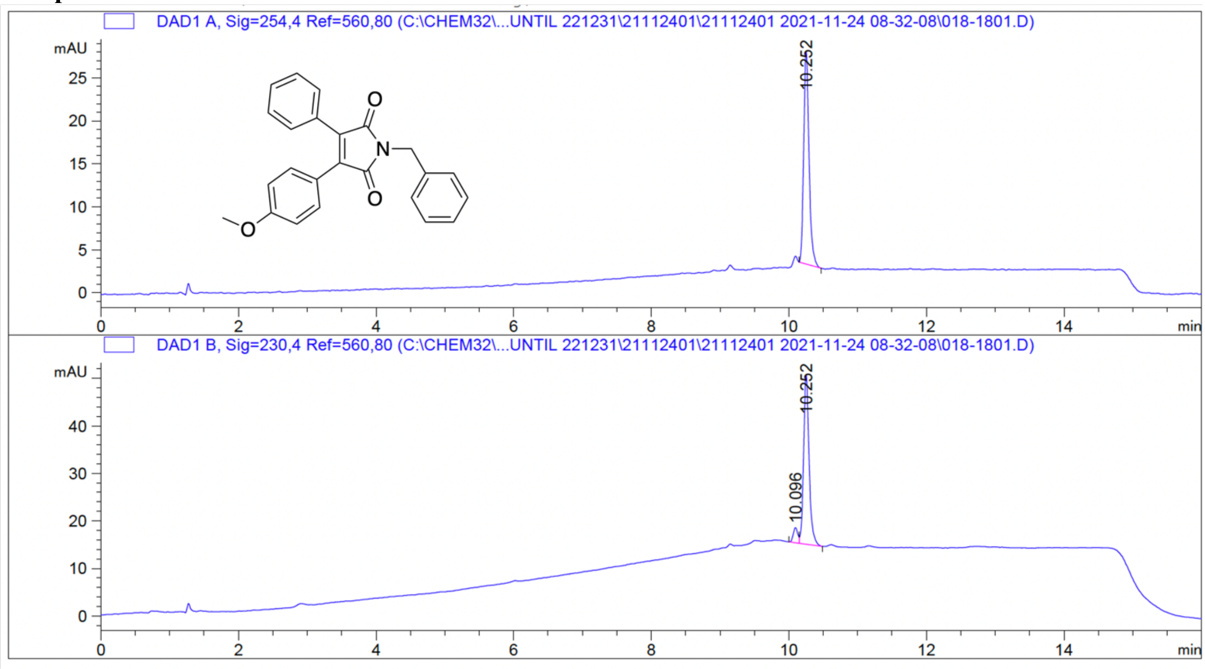

Signal 1: DAD1 A, Sig=254,4 Ref=560,80

Peak #	RetTime [min]	Type	Width [min]	Area [mAU*s]	Height [mAU]	Area %
1	10.252	BB	0.0828	133.11987	24.73200	100.0000

Peak #	RetTime [min]	Type	Width [min]	Area [mAU*s]	Height [mAU]	Area %
1	10.096	BV	0.0714	14.50785	3.17215	6.8603
2	10.252	VB	0.0846	196.96780	35.59465	93.1397

Compound 28

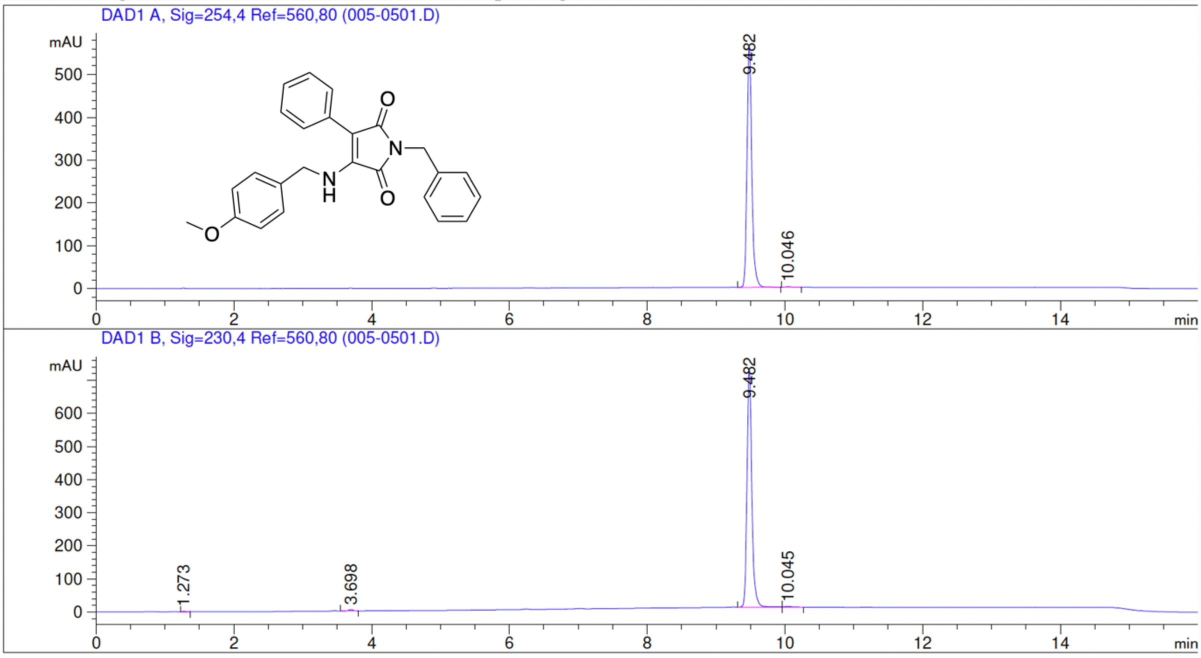

Peak #	RetTime [min]	Type	Width [min]	Area [mAU*s]	Height [mAU]	Area %
1	9.482	BB	0.0739	2718.31641	566.82501	99.7138
2	10.046	BB	0.0871	7.80306	1.31703	0.2862

Peak #	RetTime [min]	Type	Width [min]	Area [mAU*s]	Height [mAU]	Area %
1	1.273	BB	0.0482	5.73506	1.85071	0.1624
2	3.698	BB	0.0764	18.87581	3.90456	0.5345
3	9.482	BV	0.0744	3489.71606	721.35419	98.8164
4	10.045	VB	0.1046	17.18938	2.31573	0.4867

Compound 29

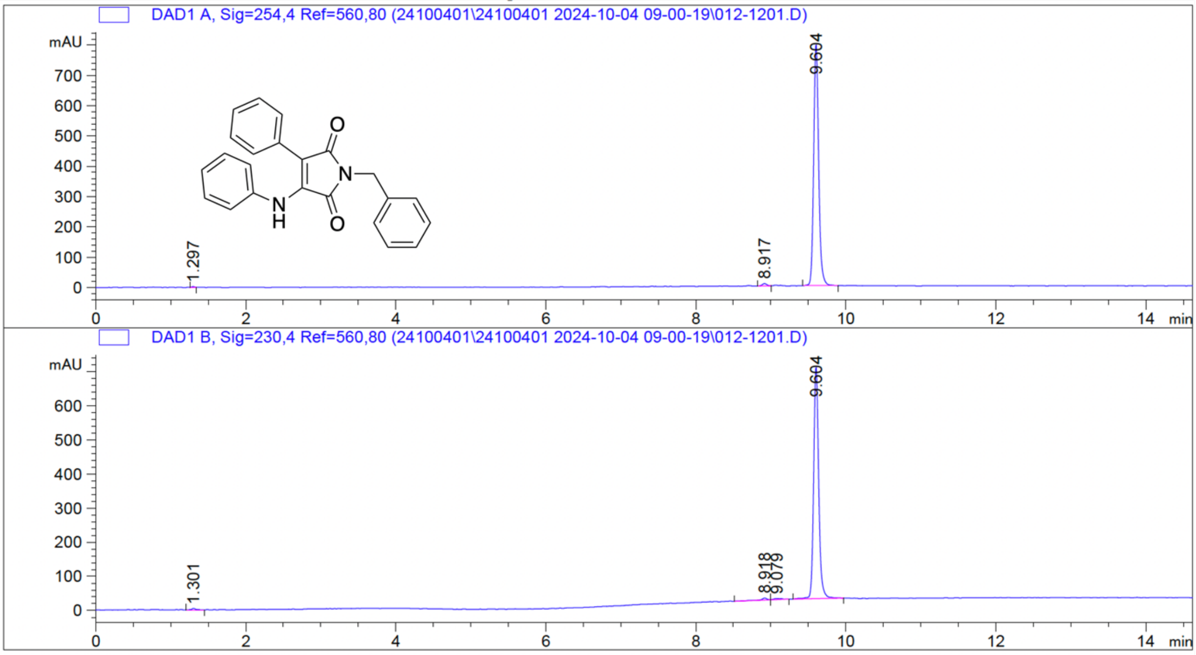

Signal 1: DAD1 A, Sig=254,4 Ref=560,80

Peak #	RetTime [min]	Type	Width [min]	Area [mAU*s]	Height [mAU]	Area %
1	1.297	BB	0.0403	6.51588	2.68351	0.1754
2	8.917	BB	0.0647	33.07352	7.92415	0.8902
3	9.604	BB	0.0697	3675.81689	799.05615	98.9345

Peak #	RetTime [min]	Type	Width [min]	Area [mAU*s]	Height [mAU]	Area %
1	1.301	BB	0.0588	16.39185	4.08329	0.5036
2	8.918	BV	0.0901	37.93097	5.96855	1.1653
3	9.079	VB	0.0960	24.05089	3.33882	0.7389
4	9.604	BB	0.0704	3176.62280	681.50513	97.5922

Compound 30

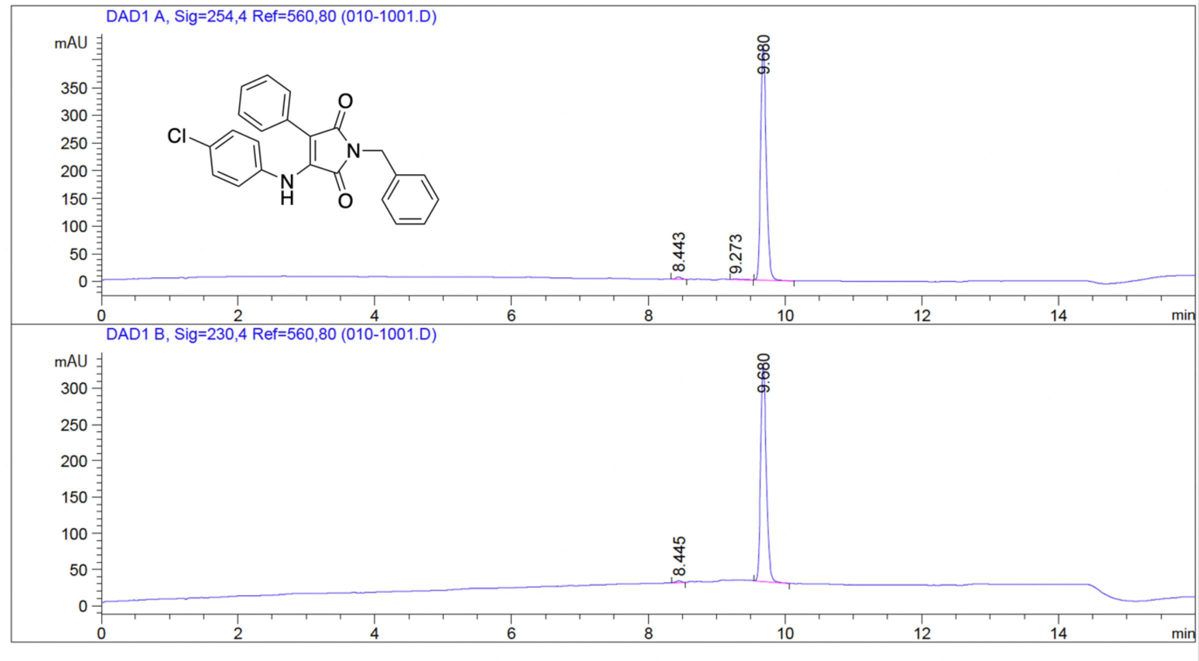

Peak #	RetTime [min]	Type	Width [min]	Area [mAU*s]	Height [mAU]	Area %
1	8.443	BB	0.0866	22.01128	3.97292	0.9001
2	9.273	BB	0.1087	9.88111	1.29970	0.4041
3	9.680	BB	0.0886	2413.43042	423.00668	98.6958

Signal 2: DAD1 B, Sig=230,4 Ref=560,80

Peak #	RetTime [min]	Type	Width [min]	Area [mAU*s]	Height [mAU]	Area %
1	8.445	BB	0.0827	12.87556	2.47385	0.7490
2	9.680	BB	0.0885	1706.05579	299.35757	99.2510

Compound 31

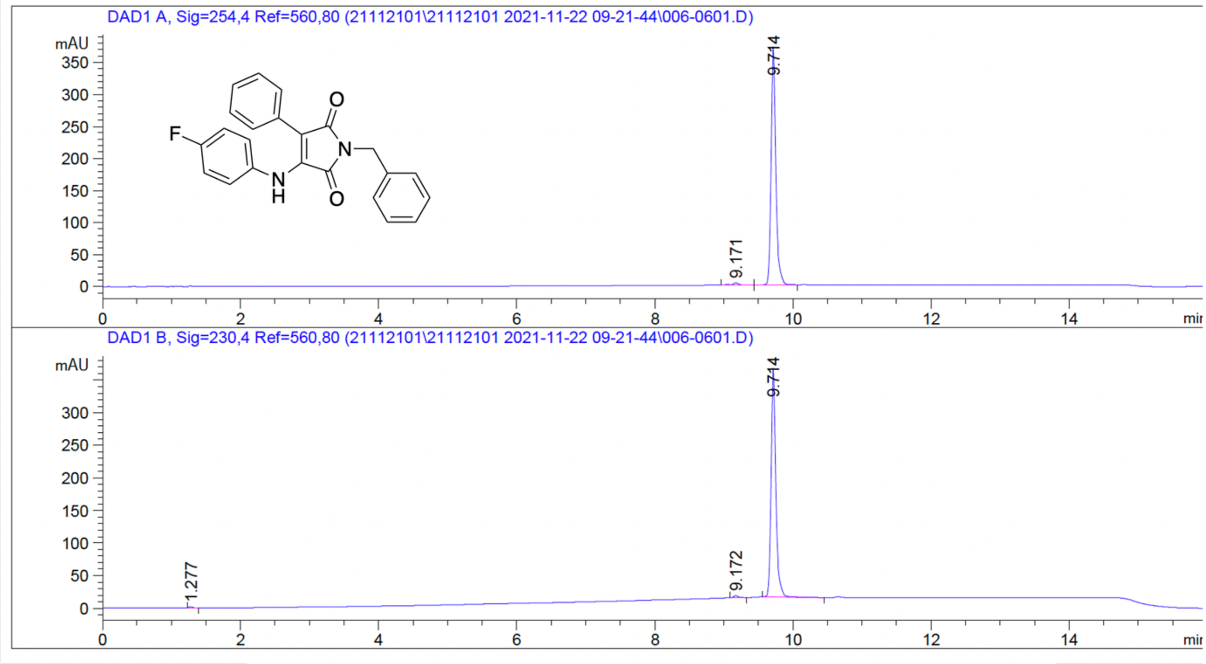

Signal 1: DAD1 A, Sig=254,4 Ref=560,80

Peak #	RetTime [min]	Type	Width [min]	Area [mAU*s]	Height [mAU]	Area %
1	9.171	BB	0.0918	23.65084	3.64024	1.3231
2	9.714	BB	0.0733	1763.90662	371.84464	98.6769

Signal 2: DAD1 B, Sig=230,4 Ref=560,80

Peak #	RetTime [min]	Type	Width [min]	Area [mAU*s]	Height [mAU]	Area %
1	1.277	BB	0.0483	5.90686	1.89674	0.3470
2	9.172	BB	0.0707	16.14310	3.44384	0.9483
3	9.714	BB	0.0738	1680.30664	351.10599	98.7047

Compound 32

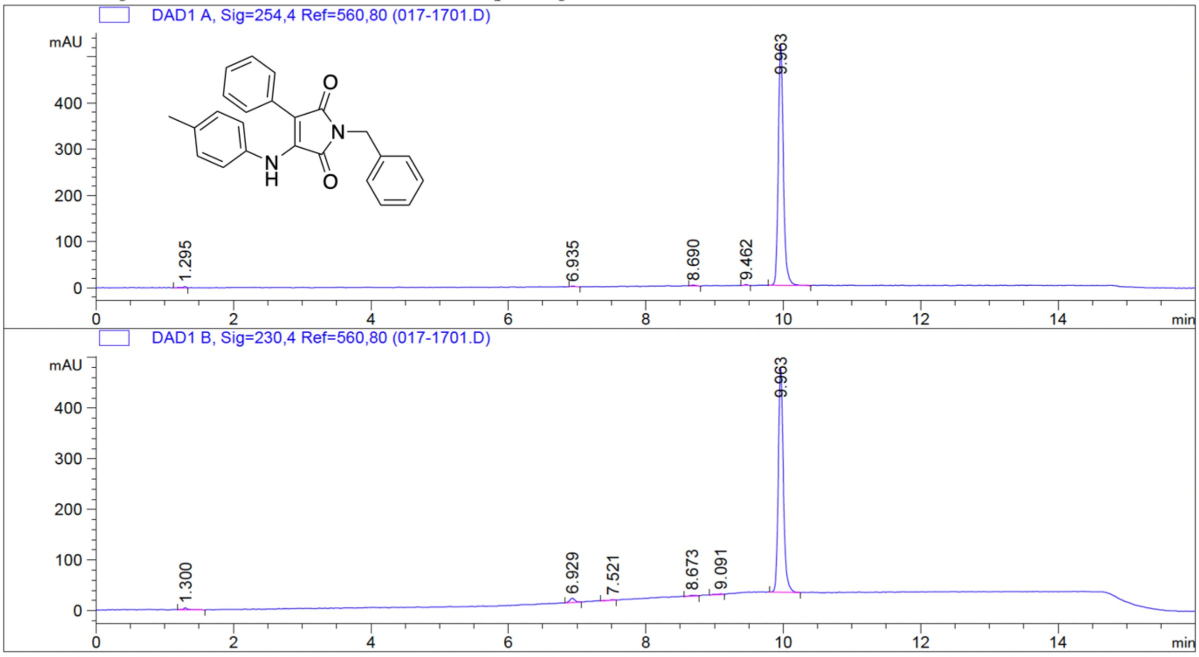

Signal 1: DAD1 A, Sig=254,4 Ref=560,80

Peak #	RetTime [min]	Type	Width [min]	Area [mAU*s]	Height [mAU]	Area %
1	1.295	BB	0.0523	9.34887	2.57844	0.3455
2	6.935	BB	0.0581	5.84869	1.47984	0.2161
3	8.690	BB	0.0705	7.87334	1.81866	0.2909
4	9.462	BB	0.0559	6.65291	1.85381	0.2458
5	9.963	BB	0.0798	2676.51685	522.21057	98.9017

Signal 2: DAD1 B, Sig=230,4 Ref=560,80

Peak #	RetTime [min]	Type	Width [min]	Area [mAU*s]	Height [mAU]	Area %
1	1.300	BB	0.0641	16.28227	3.64335	0.6924
2	6.929	BB	0.0796	43.09681	8.42966	1.8328
3	7.521	BB	0.0839	6.37782	1.03482	0.2712
4	8.673	BB	0.0863	10.81088	1.74506	0.4598
5	9.091	BB	0.0951	11.07599	1.55451	0.4710
6	9.963	BB	0.0794	2263.80151	444.41663	96.2728

Compound 33

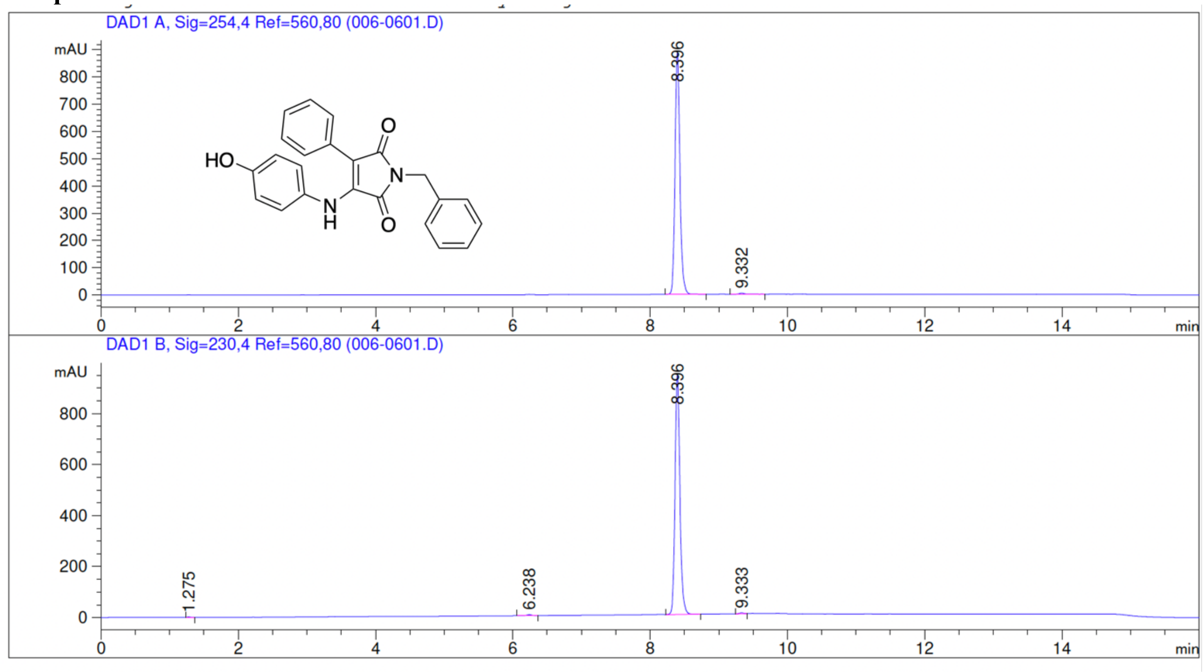

Signal 1: DAD1 A, Sig=254,4 Ref=560,80

Peak #	RetTime [min]	Type	Width [min]	Area [mAU*s]	Height [mAU]	Area %
1	8.396	BB	0.0755	4393.40430	891.74811	99.4402
2	9.332	BB	0.0834	24.73240	4.41282	0.5598

Peak #	RetTime [min]	Type	Width [min]	Area [mAU*s]	Height [mAU]	Area %
1	1.275	BB	0.0473	5.29492	1.75038	0.1132
2	6.238	BB	0.0786	18.58273	3.69855	0.3972
3	8.396	BB	0.0754	4640.40771	943.00568	99.1882
4	9.333	BB	0.0630	14.10284	3.49729	0.3014

Compound 34

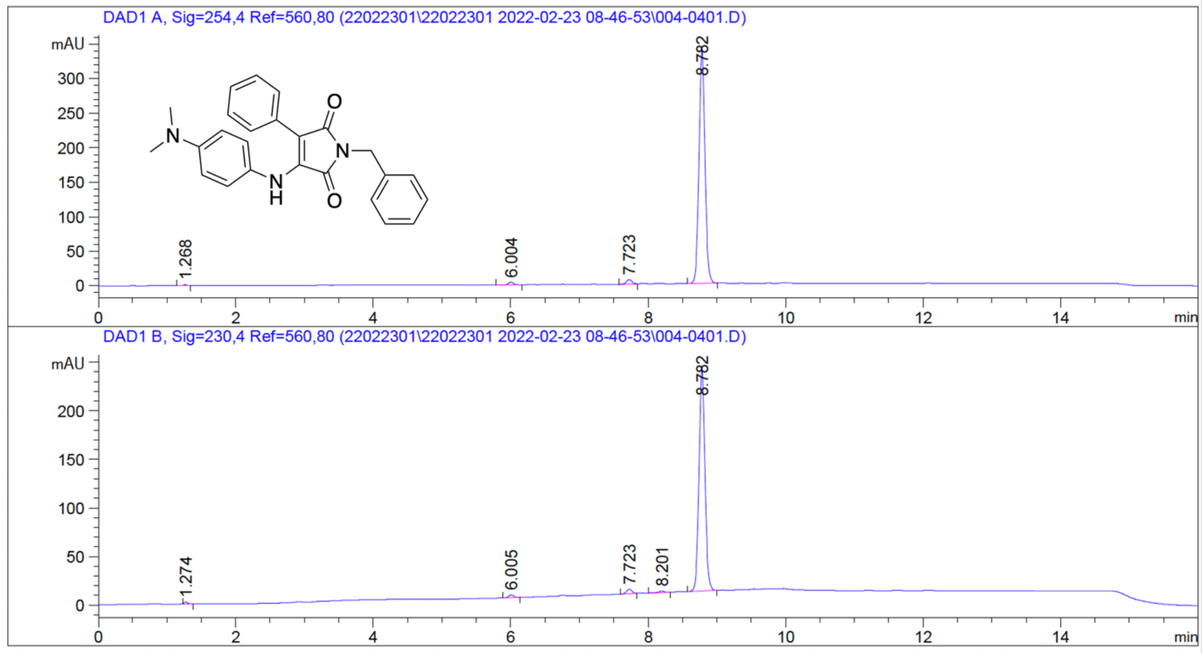

Signal 1: DAD1 A, Sig=254,4 Ref=560,80

Peak #	RetTime [min]	Type	Width [min]	Area [mAU*s]	Height [mAU]	Area %
1	1.268	BB	0.0567	5.53465	1.51134	0.2620
2	6.004	BB	0.0798	21.20585	4.14040	1.0040
3	7.723	BB	0.0898	35.27032	6.25717	1.6699
4	8.782	BB	0.0918	2050.09619	342.82758	97.0640

Signal 2: DAD1 B, Sig=230,4 Ref=560,80

Peak #	RetTime [min]	Type	Width [min]	Area [mAU*s]	Height [mAU]	Area %
1	1.274	BB	0.0459	5.28412	1.82043	0.3692
2	6.005	BB	0.0793	14.26657	2.80520	0.9967
3	7.723	BB	0.0897	25.64977	4.55544	1.7919
4	8.201	BB	0.0845	9.61475	1.68584	0.6717
5	8.782	BB	0.0916	1376.59778	230.79660	96.1706

Compound 35

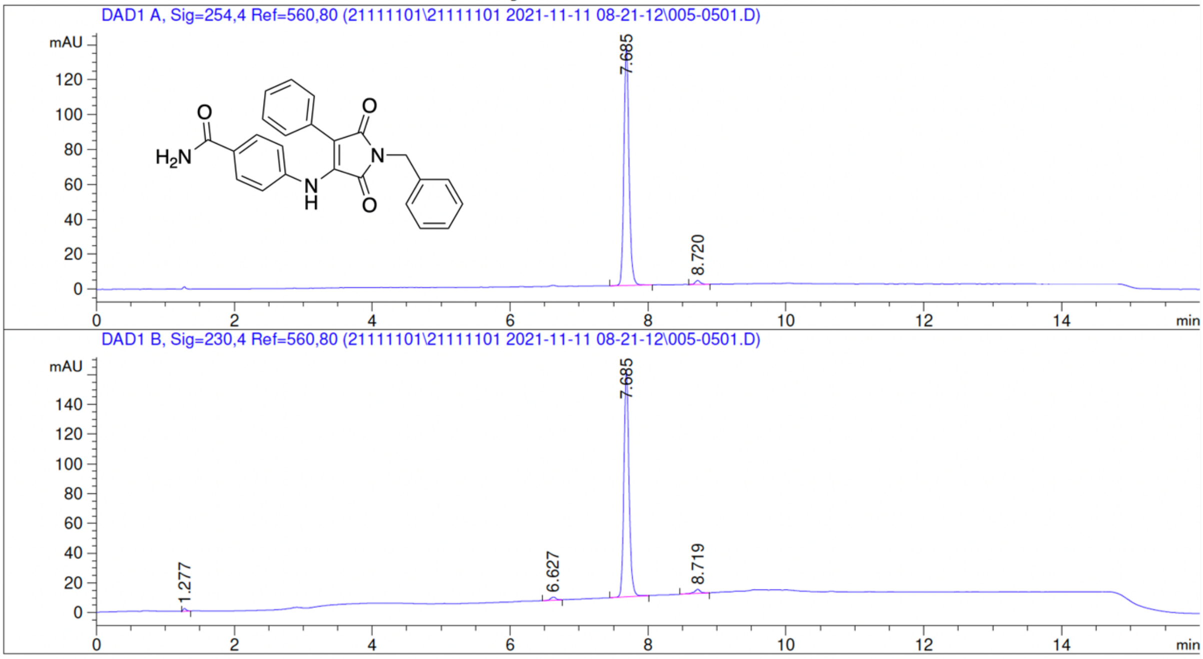

Signal 1: DAD1 A, Sig=254,4 Ref=560,80

Peak #	RetTime [min]	Type	Width [min]	Area [mAU*s]	Height [mAU]	Area %
1	7.685	BB	0.0799	706.46613	137.65070	98.3783
2	8.720	BB	0.0770	11.64552	2.30136	1.6217

Signal 2: DAD1 B, Sig=230,4 Ref=560,80

Peak #	RetTime [min]	Type	Width [min]	Area [mAU*s]	Height [mAU]	Area %
1	1.277	BB	0.0473	5.87482	1.94162	0.7174
2	6.627	BB	0.0822	11.39203	2.13699	1.3912
3	7.685	BB	0.0799	784.28253	152.81776	95.7784
4	8.719	BB	0.0912	17.30162	2.75747	2.1129

Compound 36

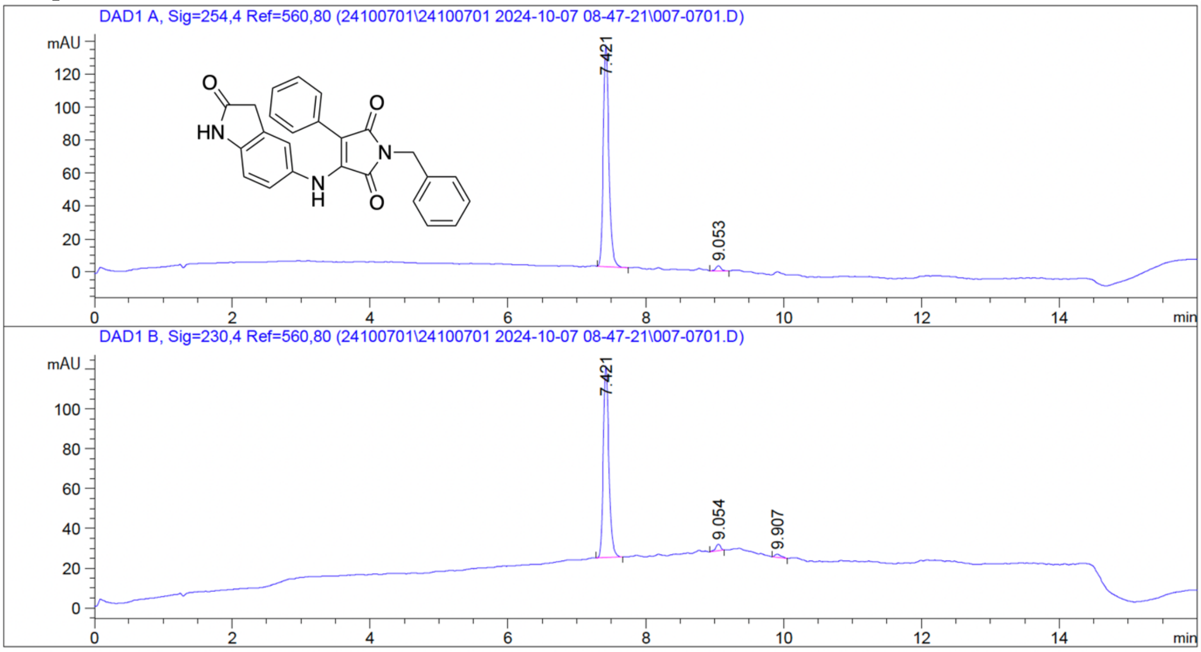

Signal 1: DAD1 A, Sig=254,4 Ref=560,80

Peak #	RetTime [min]	Type	Width [min]	Area [mAU*s]	Height [mAU]	Area %
1	7.421	BB	0.0873	749.64087	133.87224	97.9220
2	9.053	BB	0.0790	15.90807	3.04438	2.0780

Signal 2: DAD1 B, Sig=230,4 Ref=560,80

Peak #	RetTime [min]	Type	Width [min]	Area [mAU*s]	Height [mAU]	Area %
1	7.421	BB	0.0872	536.02002	95.91282	95.2921
2	9.054	BB	0.0768	16.75248	3.32127	2.9782
3	9.907	BB	0.0940	9.72977	1.53340	1.7297

Compound 37

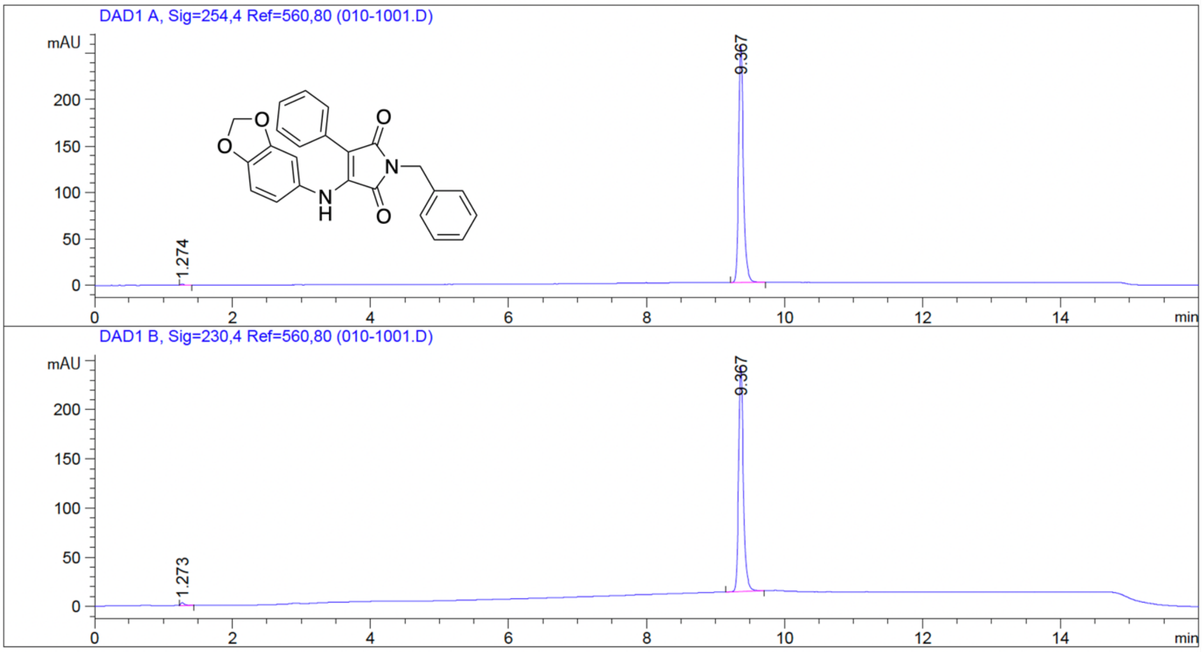

Signal 1: DAD1 A, Sig=254,4 Ref=560,80

Peak #	RetTime [min]	Type	Width [min]	Area [mAU*s]	Height [mAU]	Area %
1	1.274	BB	0.0465	4.40712	1.48846	0.3572
2	9.367	BB	0.0742	1229.36719	254.99409	99.6428

Signal 2: DAD1 B, Sig=230,4 Ref=560,80

Peak #	RetTime [min]	Type	Width [min]	Area [mAU*s]	Height [mAU]	Area %
1	1.273	BB	0.0525	10.18975	2.93774	0.9157
2	9.367	BB	0.0743	1102.65222	228.36844	99.0843

Compound 38

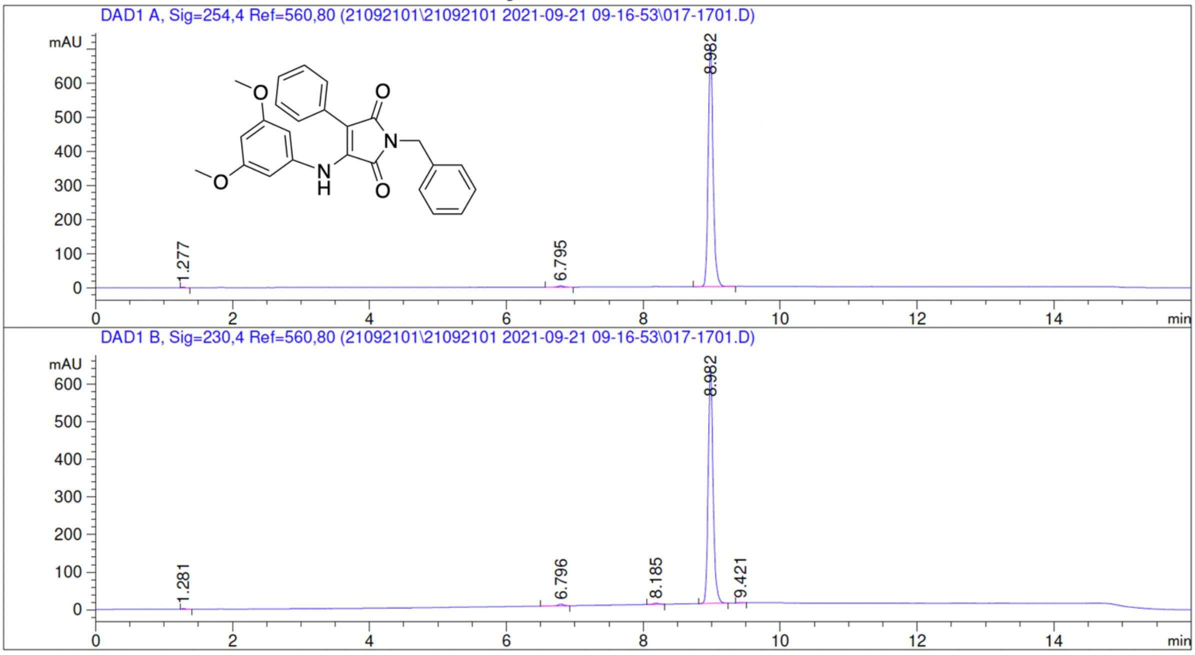

Signal 1: DAD1 A, Sig=254,4 Ref=560,80

Peak #	RetTime [min]	Type	Width [min]	Area [mAU*s]	Height [mAU]	Area %
1	1.277	BB	0.0427	4.26332	1.61901	0.1203
2	6.795	BB	0.0833	19.63149	3.50700	0.5539
3	8.982	BB	0.0759	3520.13501	708.82715	99.3258

Signal 2: DAD1 B, Sig=230,4 Ref=560,80

Peak #	RetTime [min]	Type	Width [min]	Area [mAU*s]	Height [mAU]	Area %
1	1.281	BB	0.0474	6.77048	2.23422	0.2138
2	6.796	BB	0.0848	23.95935	4.31685	0.7567
3	8.185	BB	0.0789	16.39905	3.14384	0.5179
4	8.982	BB	0.0758	3113.73120	628.04840	98.3368
5	9.421	BB	0.0632	5.53599	1.36707	0.1748

Compound 39

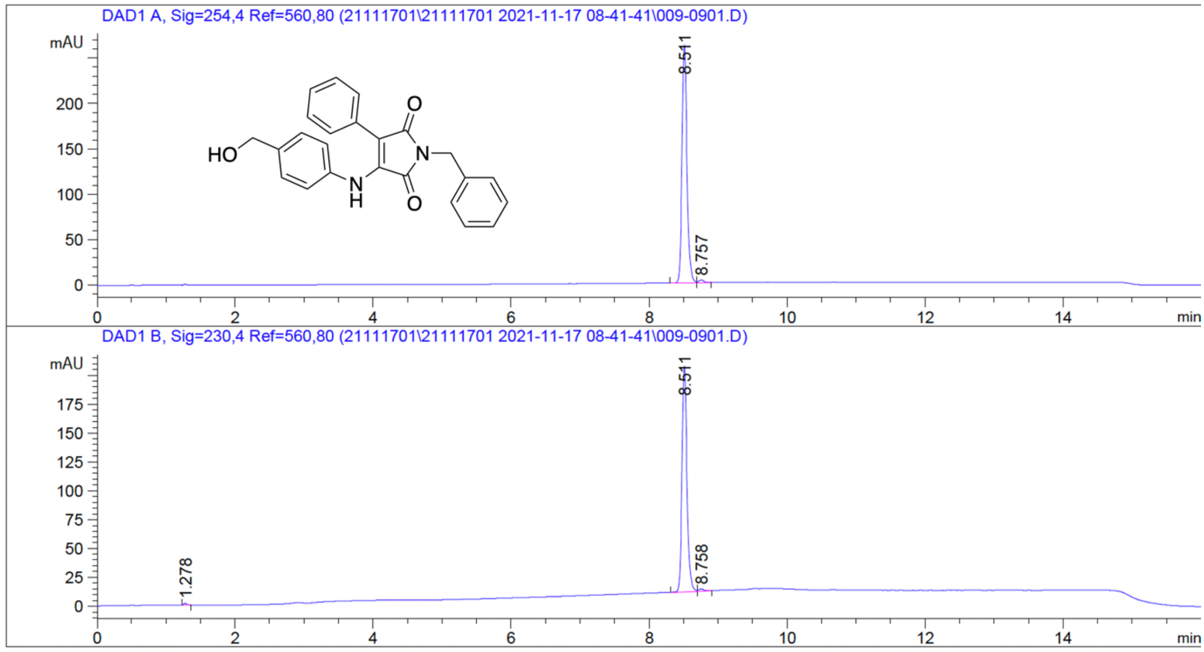

Signal 1: DAD1 A, Sig=254,4 Ref=560,80

Peak #	RetTime [min]	Type	Width [min]	Area [mAU*s]	Height [mAU]	Area %
1	8.511	BV	0.0754	1289.81116	262.05090	98.7837
2	8.757	VB	0.0790	15.88164	3.14060	1.2163

Signal 2: DAD1 B, Sig=230,4 Ref=560,80

Peak #	RetTime [min]	Type	Width [min]	Area [mAU*s]	Height [mAU]	Area %
1	1.278	BB	0.0469	5.28944	1.76948	0.5408
2	8.511	BV	0.0754	962.79303	195.60396	98.4286
3	8.758	VB	0.0792	10.08104	1.92191	1.0306

Compound 40

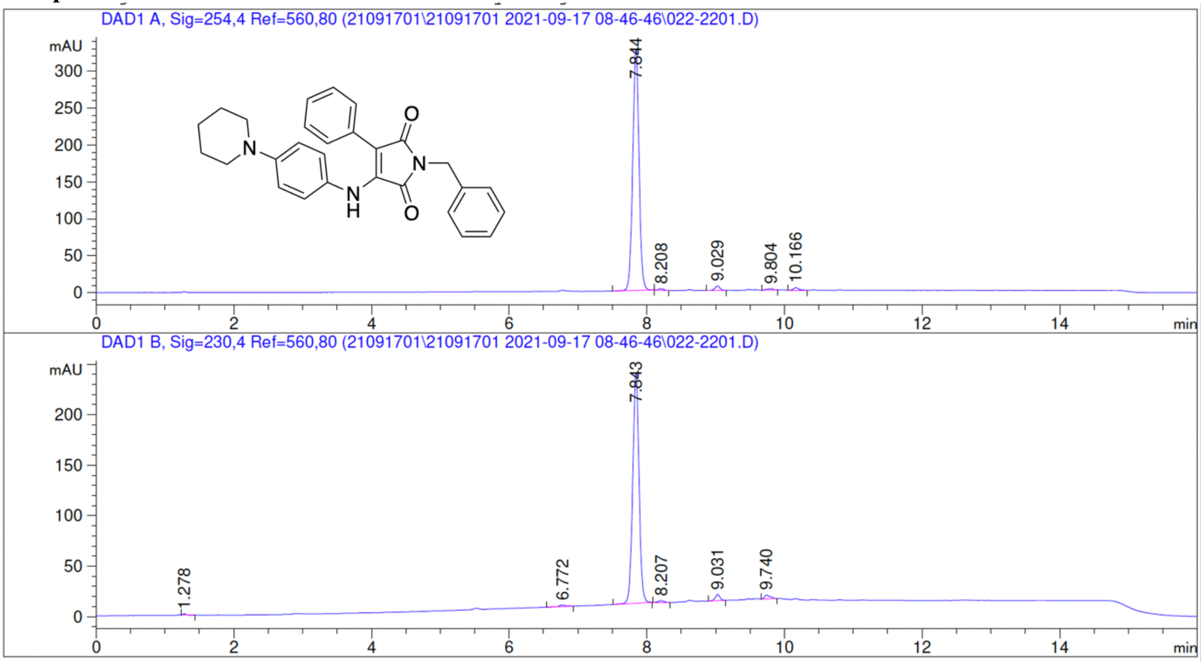

Signal 1: DAD1 A, Sig=254,4 Ref=560,80

Peak #	RetTime [min]	Type	Width [min]	Area [mAU*s]	Height [mAU]	Area %
1	7.844	BB	0.0983	2083.17700	327.18561	96.8037
2	8.208	BB	0.0807	10.65092	2.04584	0.4949
3	9.029	BB	0.0778	30.47286	5.94478	1.4161
4	9.804	BB	0.0938	10.90191	1.63527	0.5066
5	10.166	BB	0.0776	16.75768	3.27909	0.7787

Signal 2: DAD1 B, Sig=230,4 Ref=560,80

Peak #	RetTime [min]	Type	Width [min]	Area [mAU*s]	Height [mAU]	Area %
1	1.278	BB	0.0482	5.22366	1.68313	0.3403
2	6.772	BB	0.0977	11.08164	1.62303	0.7219
3	7.843	BB	0.0983	1458.48755	228.90704	95.0086
4	8.207	BB	0.0800	9.15165	1.72200	0.5962
5	9.031	BB	0.0747	29.15868	5.99577	1.8995
6	9.740	BB	0.0834	22.00712	3.80701	1.4336

Compound 41

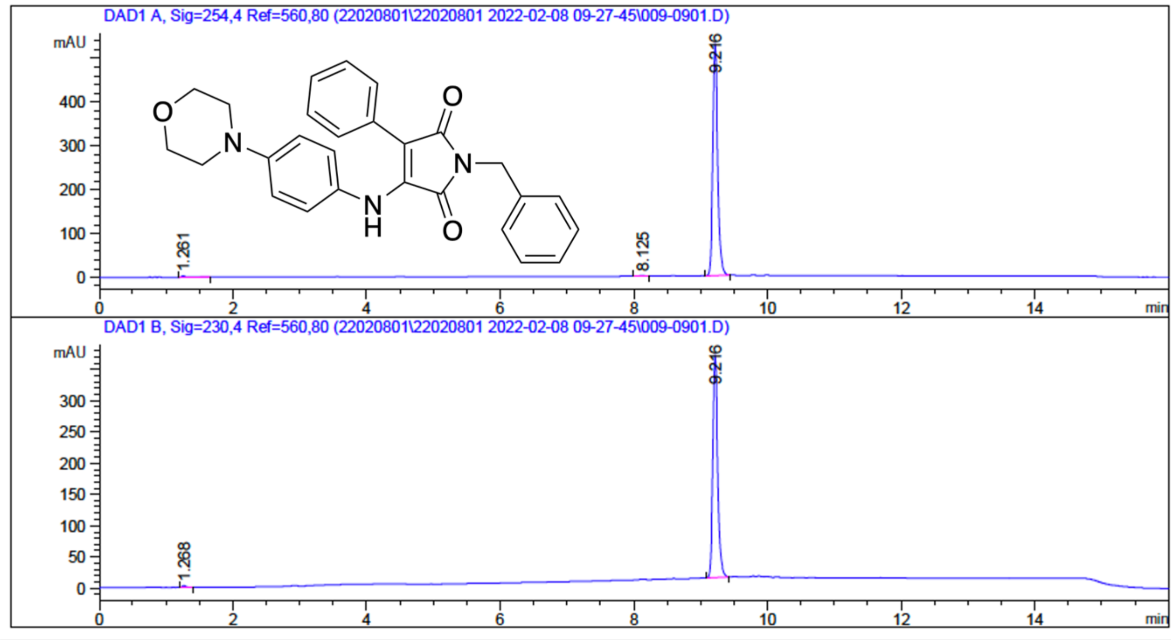

Signal 1: DAD1 A, Sig=254,4 Ref=560,80

Peak #	RetTime [min]	Type	Width [min]	Area [mAU*s]	Height [mAU]	Area %
1	1.261	BB	0.0606	13.56120	3.25252	0.5066
2	8.125	BB	0.0706	7.63802	1.63331	0.2853
3	9.216	BB	0.0767	2655.83813	528.18787	99.2081

Signal 2: DAD1 B, Sig=230,4 Ref=560,80

Peak #	RetTime [min]	Type	Width [min]	Area [mAU*s]	Height [mAU]	Area %
1	1.268	BB	0.0524	9.48866	2.73961	0.5292
2	9.216	BB	0.0765	1783.47998	355.40988	99.4708

Compound 42

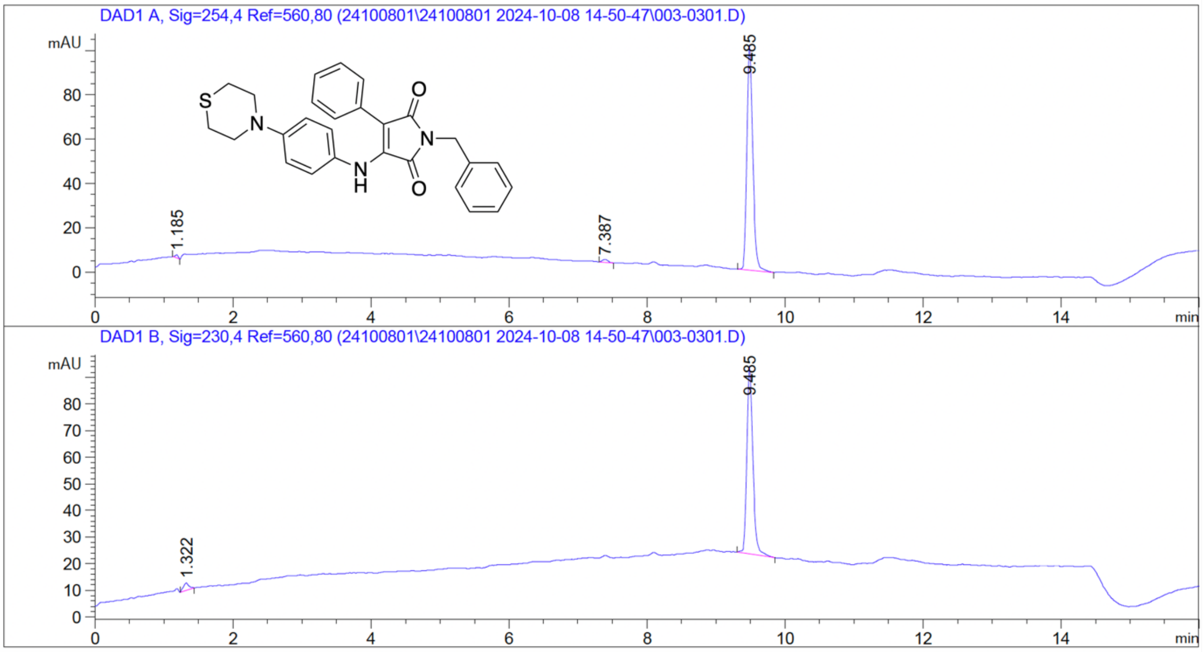

Signal 1: DAD1 A, Sig=254,4 Ref=560,80

Peak #	RetTime [min]	Type	Width [min]	Area [mAU*s]	Height [mAU]	Area %
1	1.185	BB	0.0476	4.52461	1.48422	0.7318
2	7.387	BB	0.0936	7.46031	1.32705	1.2066
3	9.485	BB	0.0917	606.29443	101.53003	98.0616

Signal 2: DAD1 B, Sig=230,4 Ref=560,80

Peak #	RetTime [min]	Type	Width [min]	Area [mAU*s]	Height [mAU]	Area %
1	1.322	BB	0.0789	15.25736	2.83161	3.4259
2	9.485	BB	0.0934	430.09204	70.31952	96.5741

Compound 43

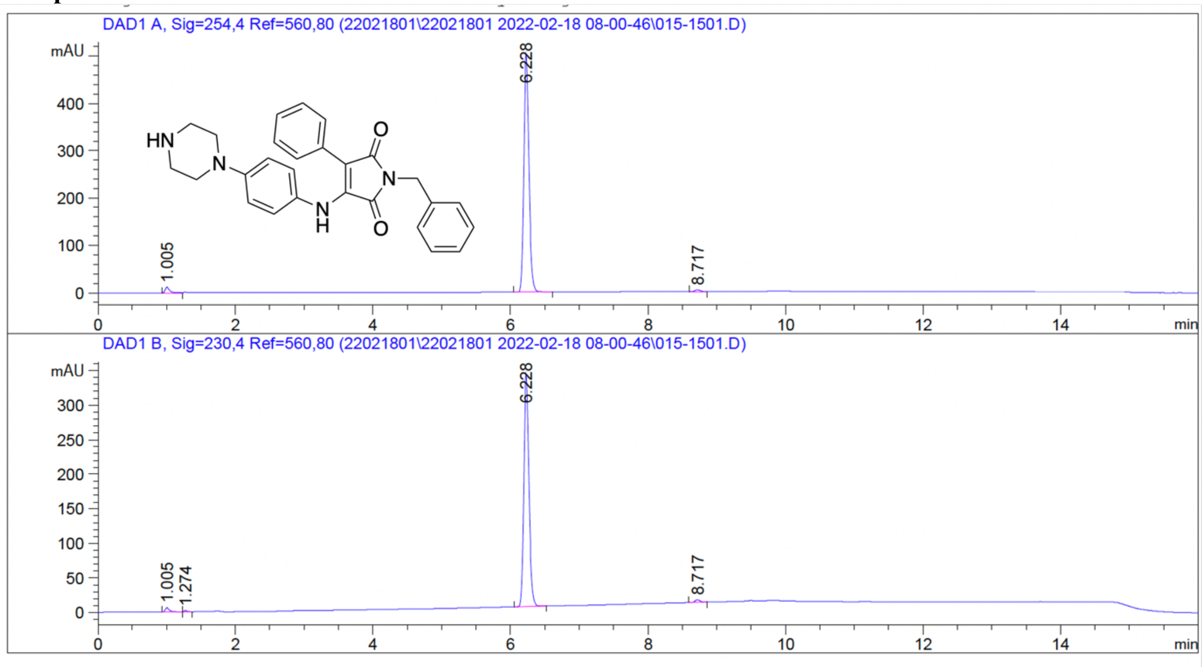

Signal 1: DAD1 A, Sig=254,4 Ref=560,80

Peak #	RetTime [min]	Type	Width [min]	Area [mAU*s]	Height [mAU]	Area %
1	1.005	BB	0.0606	52.06165	12.49851	1.9688
2	6.228	BB	0.0798	2575.48804	502.81793	97.3969
3	8.717	BB	0.0767	16.77351	3.33264	0.6343

Signal 2: DAD1 B, Sig=230,4 Ref=560,80

Peak #	RetTime [min]	Type	Width [min]	Area [mAU*s]	Height [mAU]	Area %
1	1.005	BB	0.0613	25.62370	6.06261	1.4444
2	1.274	BB	0.0474	5.72106	1.88763	0.3225
3	6.228	BB	0.0797	1723.03601	336.78241	97.1263
4	8.717	BB	0.0768	19.63469	3.89494	1.1068

Compound 44

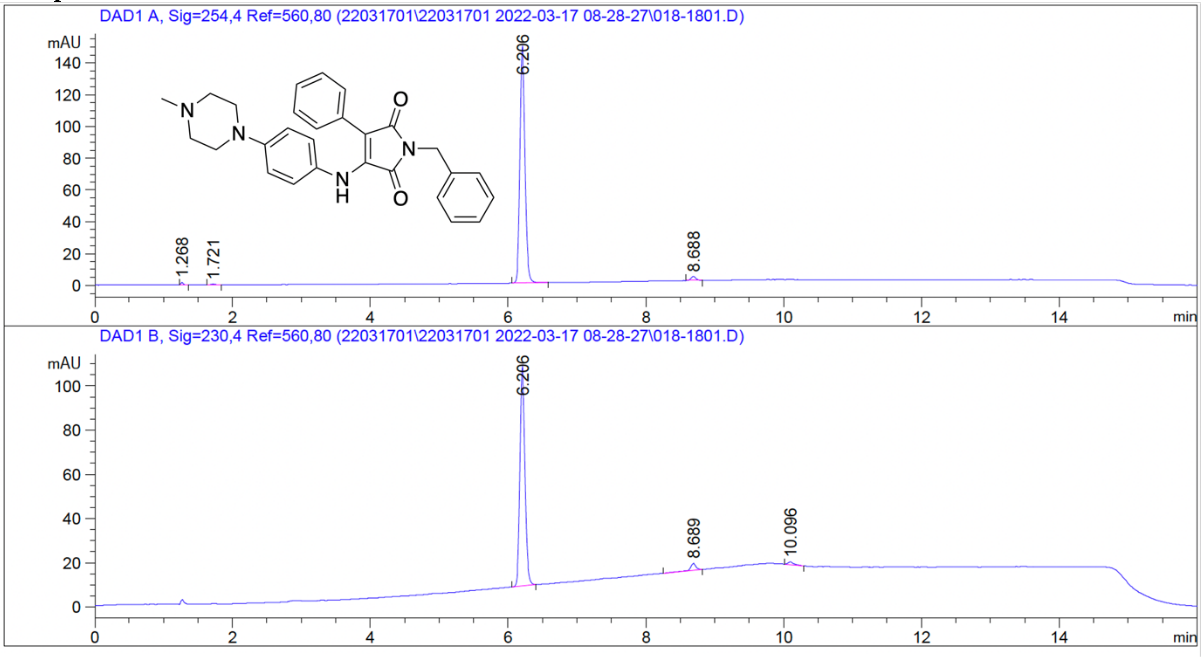

Signal 1: DAD1 A, Sig=254,4 Ref=560,80

Peak #	RetTime [min]	Type	Width [min]	Area [mAU*s]	Height [mAU]	Area %
1	1.268	BB	0.0426	3.93171	1.50068	0.5022
2	1.721	BB	0.0672	2.40266	5.26499e-1	0.3069
3	6.206	BB	0.0777	763.57544	149.29169	97.5316
4	8.688	BB	0.0744	12.99035	2.68526	1.6593

Signal 2: DAD1 B, Sig=230,4 Ref=560,80

Peak #	RetTime [min]	Type	Width [min]	Area [mAU*s]	Height [mAU]	Area %
1	6.206	BB	0.0774	505.02255	99.27365	95.2799
2	8.689	BB	0.0829	17.21009	3.09215	3.2469
3	10.096	BB	0.0850	7.80821	1.32095	1.4731

Compound 45

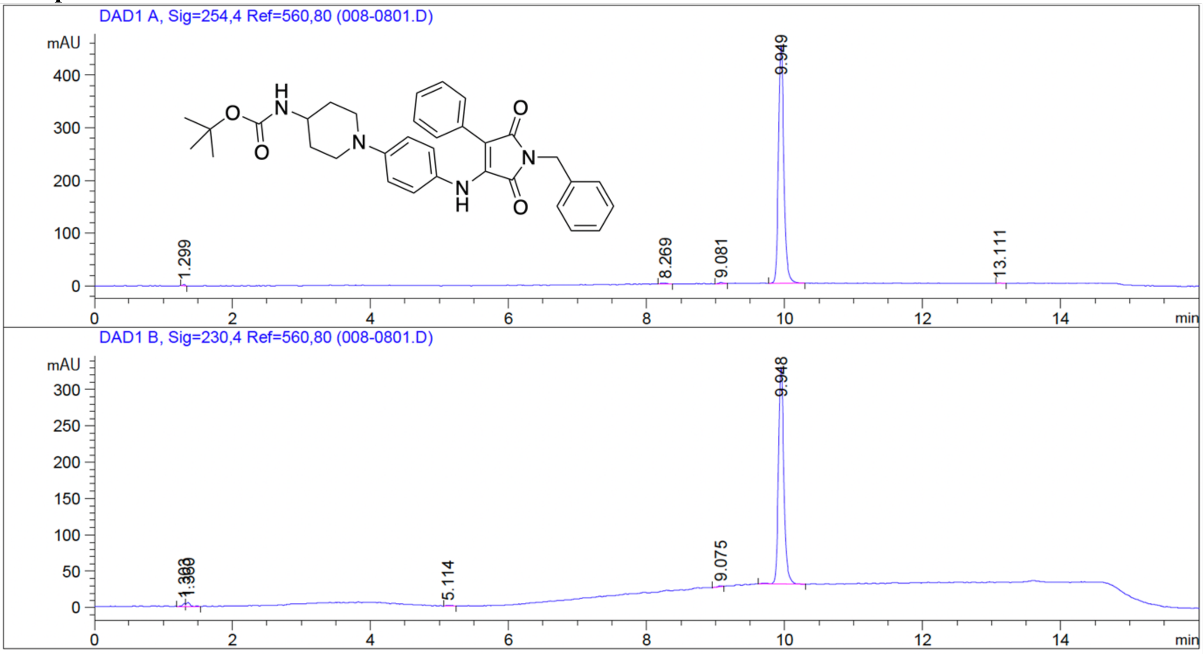

Signal 1: DAD1 A, Sig=254,4 Ref=560,80

Peak #	RetTime [min]	Type	Width [min]	Area [mAU*s]	Height [mAU]	Area %
1	1.299	BB	0.0408	5.84936	2.36875	0.2366
2	8.269	BB	0.0997	11.52878	1.82565	0.4664
3	9.081	BB	0.0718	10.91668	2.36788	0.4417
4	9.949	BB	0.0831	2438.15967	450.92560	98.6401
5	13.111	BB	0.0673	5.31795	1.12198	0.2151

Signal 2: DAD1 B, Sig=230,4 Ref=560,80

Peak #	RetTime [min]	Type	Width [min]	Area [mAU*s]	Height [mAU]	Area %
1	1.303	BV	0.0456	10.66629	3.70658	0.6443
2	1.360	VB	0.0528	18.93938	5.16609	1.1440
3	5.114	BB	0.0893	7.06188	1.12341	0.4266
4	9.075	BB	0.0575	6.09957	1.56515	0.3684
5	9.948	BB	0.0832	1612.78931	297.77420	97.4168

Compound 46

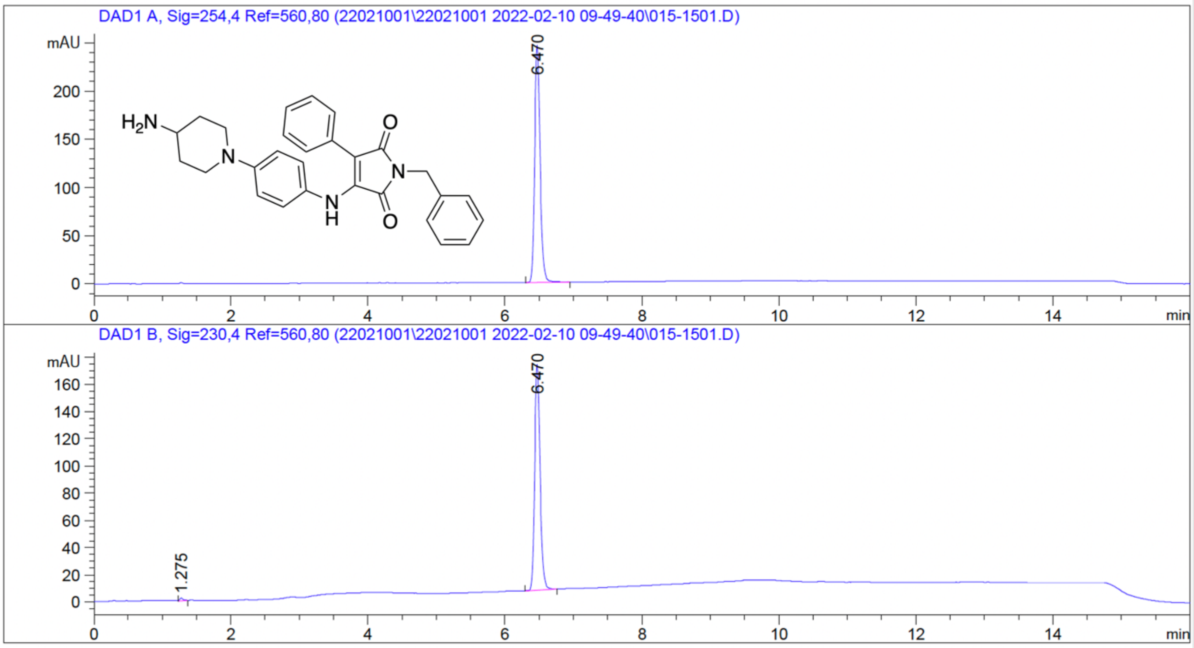

Signal 1: DAD1 A, Sig=254,4 Ref=560,80

Peak #	RetTime [min]	Type	Width [min]	Area [mAU*s]	Height [mAU]	Area %
1	6.470	BB	0.0839	1348.57043	246.21469	100.0000

Signal 2: DAD1 B, Sig=230,4 Ref=560,80

Peak #	RetTime [min]	Type	Width [min]	Area [mAU*s]	Height [mAU]	Area %
1	1.275	BB	0.0493	6.16887	1.93078	0.6772
2	6.470	BB	0.0835	904.77039	166.29216	99.3228

Compound 47

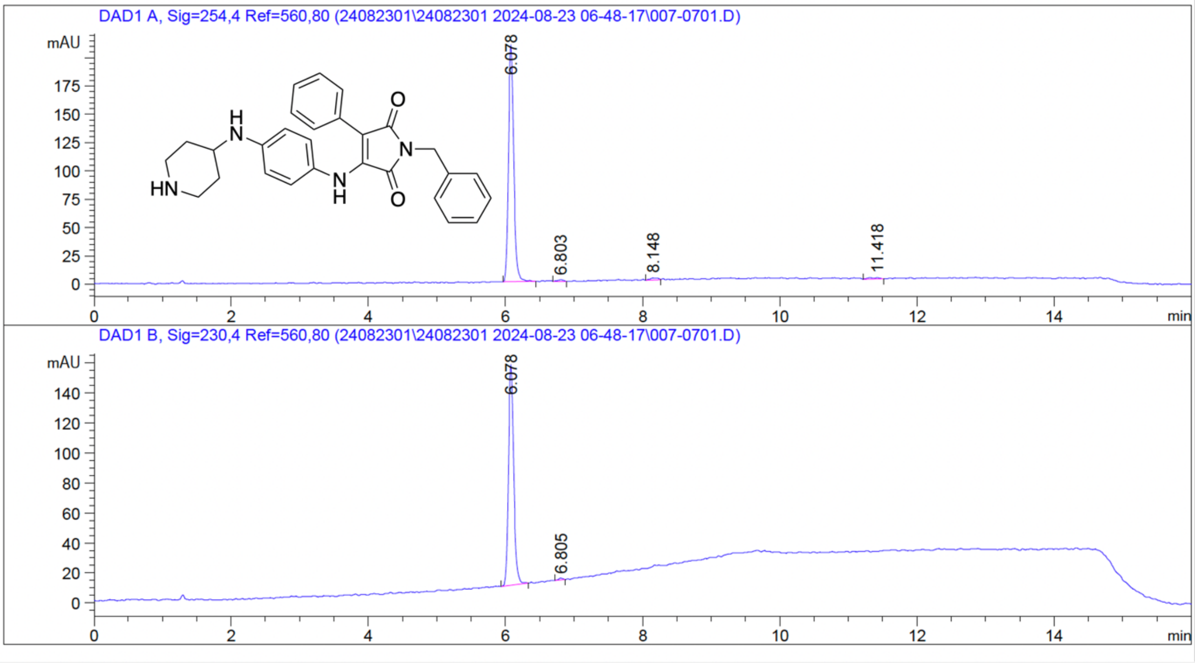

Signal 1: DAD1 A, Sig=254,4 Ref=560,80

Peak #	RetTime [min]	Type	Width [min]	Area [mAU*s]	Height [mAU]	Area %
1	6.078	BB	0.0820	1106.49036	208.36246	96.9396
2	6.803	BB	0.0857	8.42990	1.33482	0.7385
3	8.148	VV	0.1070	14.79848	1.85725	1.2965
4	11.418	BB	0.1302	11.70387	1.15540	1.0254

Signal 2: DAD1 B, Sig=230,4 Ref=560,80

Peak #	RetTime [min]	Type	Width [min]	Area [mAU*s]	Height [mAU]	Area %
1	6.078	BB	0.0817	775.44214	146.56863	99.4027
2	6.805	BB	0.0574	4.65979	1.19873	0.5973

Compound 48

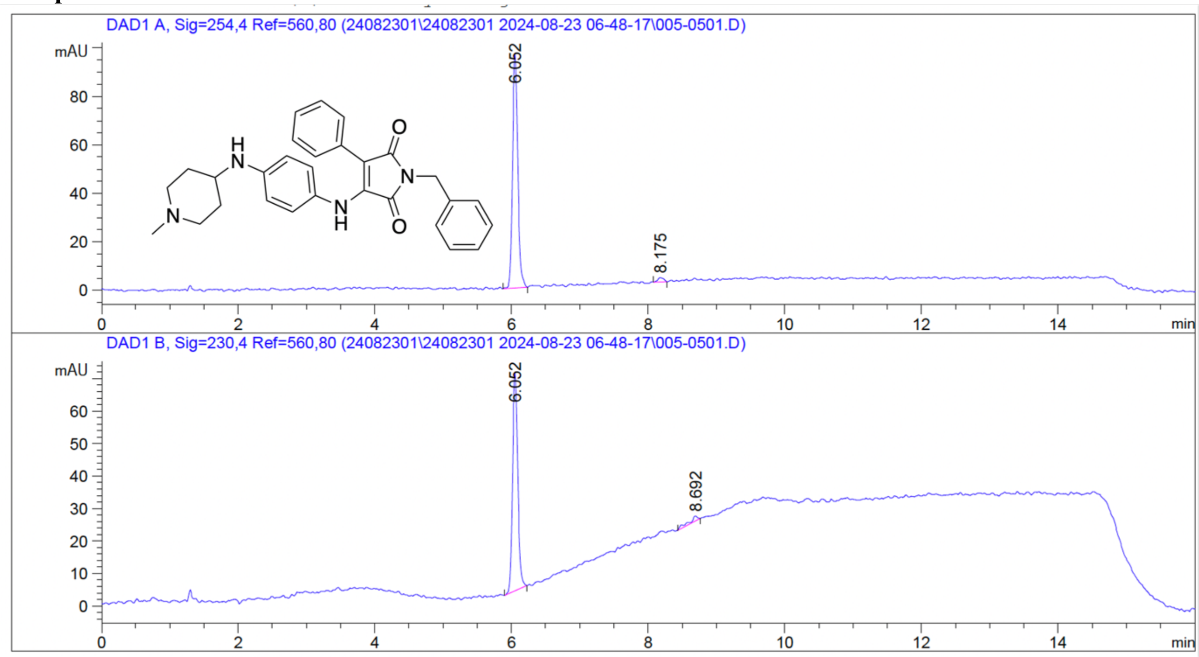

Signal 1: DAD1 A, Sig=254,4 Ref=560,80

Peak #	RetTime [min]	Type	Width [min]	Area [mAU*s]	Height [mAU]	Area %
1	6.052	BB	0.0822	514.01074	96.34083	98.0985
2	8.175	BB	0.0807	9.96339	1.74186	1.9015

Signal 2: DAD1 B, Sig=230,4 Ref=560,80

Peak #	RetTime [min]	Type	Width [min]	Area [mAU*s]	Height [mAU]	Area %
1	6.052	BB	0.0817	354.34845	67.02138	96.4786
2	8.692	BB	0.1178	12.93352	1.48198	3.5214

Compound 49

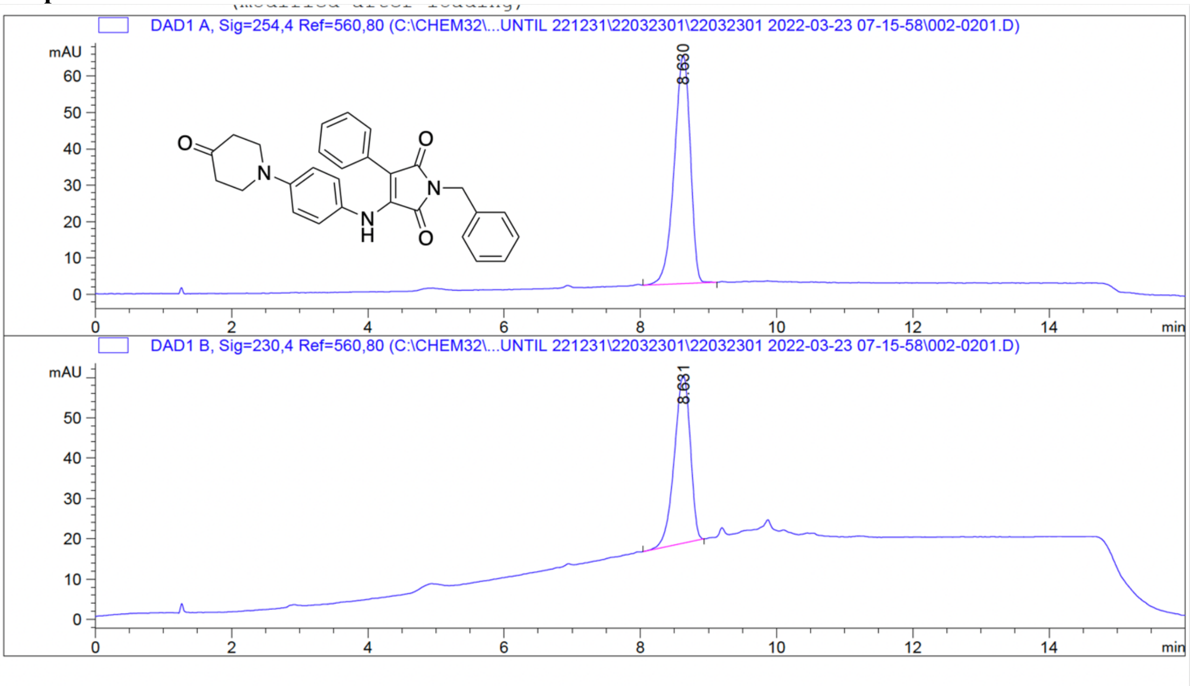

Signal 1: DAD1 A, Sig=254,4 Ref=560,80

Peak #	RetTime [min]	Type	Width [min]	Area [mAU*s]	Height [mAU]	Area %
1	8.630	BB	0.2549	1027.28101	62.78840	100.0000

Peak #	RetTime [min]	Type	Width [min]	Area [mAU*s]	Height [mAU]	Area %
1	8.631	BB	0.2579	676.94629	41.58319	100.0000

Compound 50

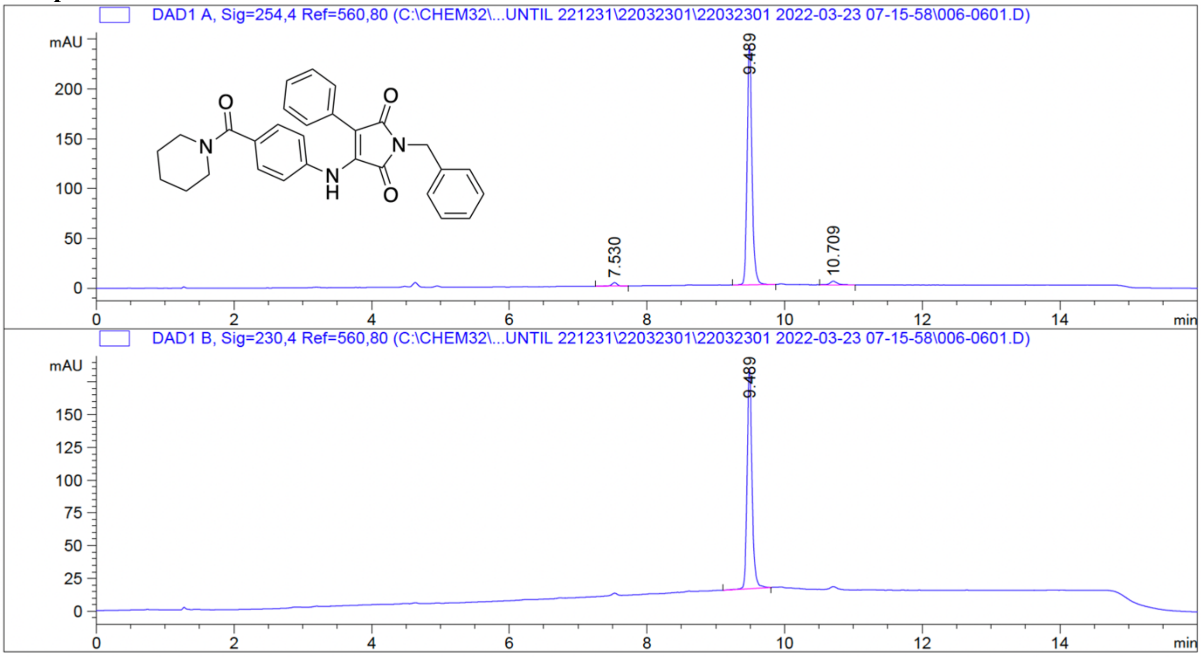

Signal 1: DAD1 A, Sig=254,4 Ref=560,80

Peak #	RetTime [min]	Type	Width [min]	Area [mAU*s]	Height [mAU]	Area %
1	7.530	BB	0.0894	20.37556	3.42584	1.6553
2	9.489	BB	0.0752	1184.02808	241.58331	96.1888
3	10.709	BB	0.1095	26.53809	3.62173	2.1559

Signal 2: DAD1 B, Sig=230,4 Ref=560,80

Peak #	RetTime [min]	Type	Width [min]	Area [mAU*s]	Height [mAU]	Area %
1	9.489	BB	0.0756	832.91229	168.77924	100.0000

Compound 51

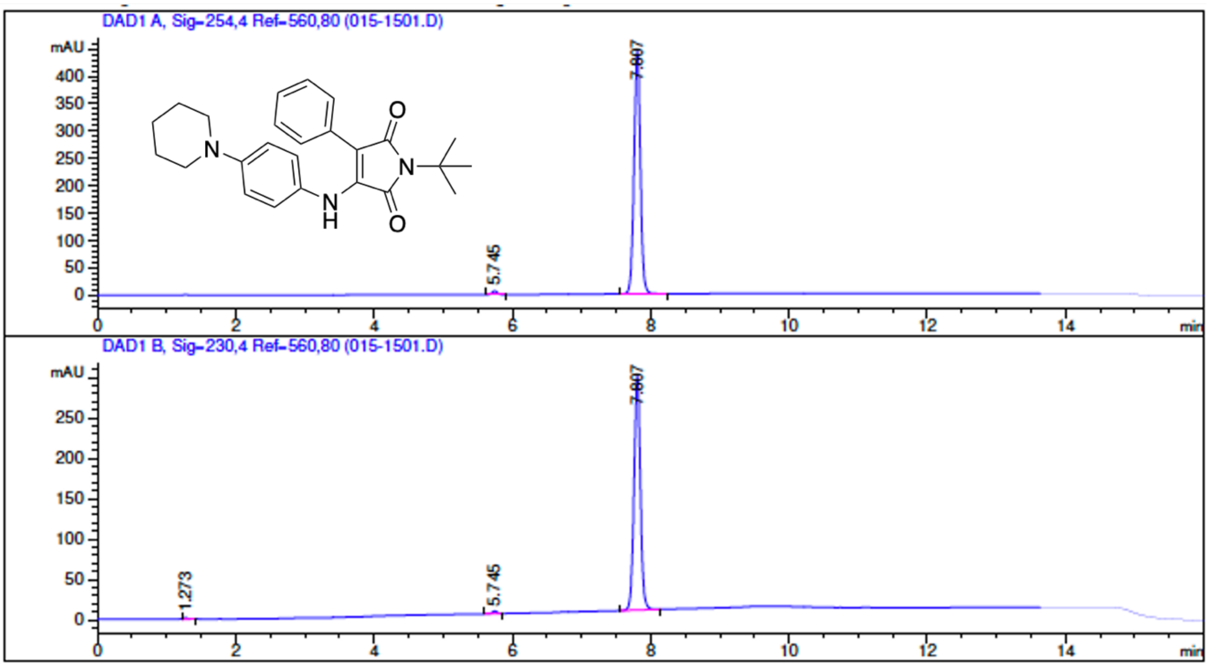

Signal 1: DAD1 A, Sig=254,4 Ref=560,80

Peak #	RetTime [min]	Type	Width [min]	Area [mAU*s]	Height [mAU]	Area %
1	5.745	BB	0.0789	32.70832	6.48417	1.1349
2	7.807	BB	0.0988	2849.34131	444.22339	98.8651

Peak #	RetTime [min]	Type	Width [min]	Area [mAU*s]	Height [mAU]	Area %
1	1.273	BB	0.0505	6.14412	1.86082	0.3260
2	5.745	BB	0.0779	15.66260	3.15662	0.8311
3	7.807	BB	0.0990	1862.83533	289.92218	98.8429

Compound 52

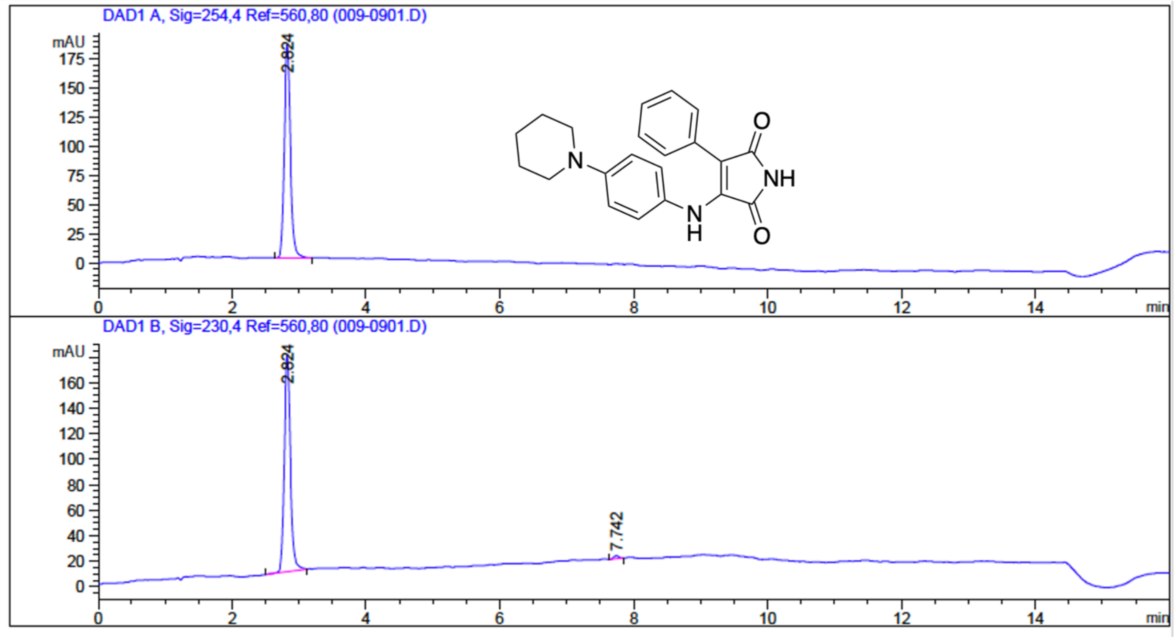

Signal 2: DAD1 B, Sig=230,4 Ref=560,80

Peak #	RetTime [min]	Type	Width [min]	Area [mAU*s]	Height [mAU]	Area %
1	2.824	BB	0.0976	1070.02209	169.71819	98.8159
2	7.742	BB	0.0848	12.82199	2.38174	1.1841

Signal 1: DAD1 A, Sig=254,4 Ref=560,80

Peak #	RetTime [min]	Type	Width [min]	Area [mAU*s]	Height [mAU]	Area %
1	2.824	BB	0.0971	1145.50793	182.91136	100.0000
